# How Does the Powder Mixture of Ibuprofen and Caffeine Attenuate the Solubility of Ibuprofen? Comparative Study for the Xanthine Derivatives to Recognize Their Intermolecular Interactions Using Fourier-Transform Infrared (FTIR) Spectra, Differential Scanning Calorimetry (DSC), and X-ray Powder Diffractometry (XRPD)

**DOI:** 10.1021/acs.molpharmaceut.4c00429

**Published:** 2024-08-07

**Authors:** Shoya Suenaga, Hikaru Kataoka, Kanji Hasegawa, Ryotaro Koga, Chihiro Tsunoda, Wataru Kuwashima, Tomohiro Tsuchida, Satoru Goto

**Affiliations:** Faculty of Pharmaceutical Sciences, Tokyo University of Science, 2641 Yamazaki, Noda, Chiba, Japan 278-8510

**Keywords:** attenuated total reflection-Fourier-transform infrared (ATR-FTIR) spectrometry, X-ray powder diffraction (XRPD), differential scanning calorimetry (DSC), singular value decomposition (SVD), trajectory analysis

## Abstract

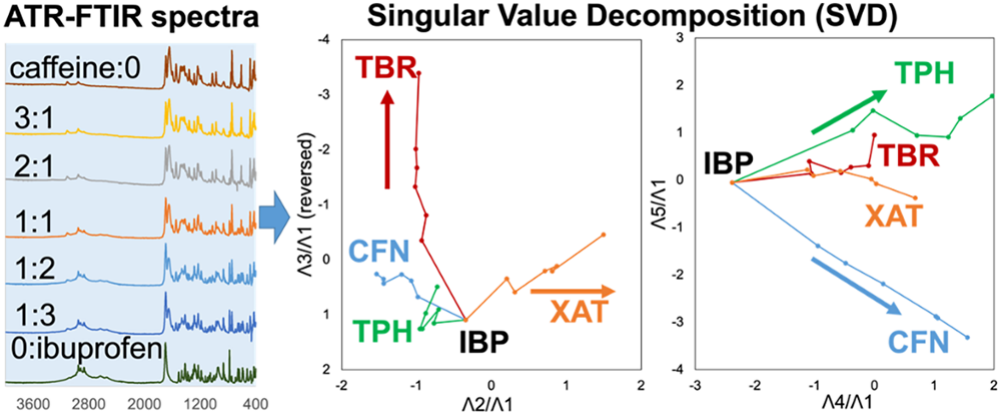

Molecular interactions between active pharmaceutical ingredients (APIs) and xanthine (XAT) derivatives were analyzed using singular value decomposition (SVD). XAT derivatives were mixed with equimolar amounts of ibuprofen (IBP) and diclofenac (DCF), and their dissolution behaviors were measured using high-performance liquid chromatography. The solubility of IBP decreased in mixtures with caffeine (CFN) and theophylline (TPH), whereas that of DCF increased in mixtures with CFN and TPH. No significant differences were observed between the mixtures of theobromine (TBR) or XAT with IBP and DCF. Mixtures with various molar ratios were analyzed using differential scanning calorimetry, X-ray powder diffraction, and Fourier-transform infrared spectroscopy to further explore these interactions. The results were subjected to SVD. This analysis provides valuable insights into the differences in interaction strength and predicted interaction sites between XAT derivatives and APIs based on the combinations that form mixtures. The results also showed the impact of the XAT derivatives on the dissolution behavior of IBP and DCF. Although IBP and DCF were found to form intermolecular interactions with CFN and TPH, these effects resulted in a reduction of the solubility of IBP and an increase in the solubility of DCF. The current approach has the potential to predict various interactions that may occur in different combinations, thereby contributing to a better understanding of the impact of health supplements on pharmaceuticals.

## 1 Introduction

**Chart 1:**
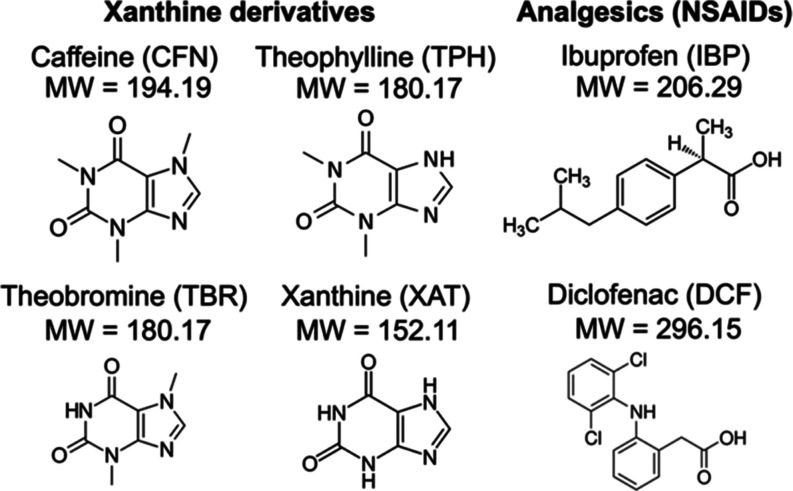
Chemical Structures of the Xanthine Derivatives and Analgesics (NSAIDs)

The potential for the consumption of supplements has increased because of the high variety of available supplements.^1,2^ Recent exposure to the caffeine (CFN) in coffee (*Coffea arabica*), theophylline (TPH) in tea (*Camellia sinensis*), and theobromine (TBR) in cacao (*Theobroma cacao*), all of which are xanthine (XAT)-derivative alkaloids that stimulate the central nervous system and can lead to addiction (Chart 1).^3,4^ Although the benefits of the consumption of these compounds have been shown, for example, the consumption of chocolate improves cognitive function and is statistically correlated with the total number of Nobel laureates per capita,^5−7^ the excessive consumption of these goods can have negative effects. Incidents of acute poisoning have been documented, particularly with caffeinated energy drinks (LD_50_ = 1 mmol/kg, distribution volume *V*_d_ = 0.7 L/kg).^3,8−10^

These topics are relevant in a number of fields including food and nutrition science, forensic medicine, health and environmental chemistry, and animal biochemistry. In pharmacology, uncontrolled intake of these common ingredients exposes the consumer to potential synergistic and antagonistic effects with medications.^11,12^ XAT derivatives are known to metabolize through the cytochrome P450 type 1A2 (CYP1A2) enzyme.^13,14^ The substrates and competitors of CYP1A2 include active pharmaceutical ingredients (APIs) of tricyclic antidepressants (amitriptyline and clomipramine), atypical antipsychotics (olanzapine and clozapine), the local anesthetic (LA) ropivacaine, the sleep-inducing drug melatonin, the serotonin-selective receptor inhibitor (SSRI) fluvoxamine, the anticoagulant warfarin, and progesterone.^15,16^ Moreover, CYP1A2 contains inhibitors such as fluoroquinolone (an antibiotic), verapamil (Ca^2+^-inhibitor), and flavanone naringenin in grapefruit juice. Administration of the proton-pump inhibitors, omeprazole, antiepileptic carbamazepine, antituberculous rifampicin, and CFN can induce the genetic expression of CYP1A2.^17^ Therefore, XAT derivatives may alter or intensify the effects of the APIs and phytochemicals.

This study focused on the physicochemical intermolecular interactions between the APIs and the XAT derivatives in diets and beverages. These interactions were considered without the constrictions of pharmacology, pharmacokinetics, or pharmacodynamics mentioned above (legal or clinical guidelines).

CFN (water solubility 110 mmol/L, log *P* = −0.07, p*K*_a_ 0.6)^18^ is a 1,3,7-trimethylated XAT derivative that forms two anhydrous crystalline polymorphisms: stable phase II (form β) at ambient temperature (with a phase transition at 426 K upon heating) and high-temperature phase I (form α), with a melting point (*T*_m_) of 512 K. The transition from phase I to phase II is complex and partial during supercooling to 400 K.^19−23^ CFN easily sublimes above 443 K, with a sublimation enthalpy of 100 kJ/mol.^23^ The monohydrate crystal of CFN contains 4/9 water molecules, which forms the anhydrous form II after dehydration.^20,21,24^ This central nervous system stimulant poses a challenge in crystal nucleation and phase transitions and has intrigued analytical and computational chemists for approximately 30 years.^20,25^ CFN is found at concentrations ranging between 0.17 to 0.33 mmol in 160 mL of drip coffee and green tea, approximately 1 mmol in 100 mL of machine-extracted coffee, and 1–2 mmol in 1 L of energy drinks.^1−4,26^ Because CFN is an API, a daily dose of 150 mg (equivalent to 0.75 mmol) elicits awakening, antipyretic, analgesic, cardiotropic, and diuretic effects.^26^ In senior citizens and pregnant women, reductions in total body water content and valid fat amounts could enhance the action of CFN, posing severe risks due to intermolecular interactions with medications, diets, and beverages.^1−3,10,26^

TPH (water solubility 40 mmol/L, log *P* = 0.00, p*K*_a_ 8.55)^18^ is a pyrimidinedione-condensed imidazole derivative that exists in two anhydrous crystalline forms, I and II (*T*_m_ values of 546 and 542 K, respectively), with identical diffractogram patterns.^20,27^ A monohydrate crystal (form M) leads to the metastable form III owing to dehydration at 353 K, whereas anhydrous forms IV and V have been identified using solvent-mediated transformation and supercritical antisolvent methods.^28−31^ TPH is therapeutically used at a daily dose of 2.2 mmol for bronchoasthma, chronic bronchitis, chronic obstructive pulmonary disease (COPD), and other conditions owing to its more potent broncho-relaxation effect than that of CFN.^32^ TPH can cause adverse effects, such as convulsions, and is prone to poisoning symptoms due to its narrow therapeutic range (0.06–0.11 mmol/L in the blood), necessitating frequent therapeutic drug monitoring.^33^ Aminophylline (neophylline and phyllocontin) is a compounding drug of TPH. Combination therapy with isoproterenol, erythromycin, cimetidine, phenytoin, and new quinolone antibiotics can lead to drug interactions, decreasing TPH clearance (increasing its concentration in blood); however, high-fat diets enhance TPH release (*V*_d_ = 0.5 L/kg).

TBR (water solubility 2 mmol/L, log *P* = −0.78, p*K*_a_ 10.05) is a TPH regioisomer that is composed of a 6-membered dicarboxyimide portion (CO–NH–CO). Polymorphs of TBR have not been reported; additionally, the anhydrous form of this compound remains stable even after 24 h in an aqueous slurry.^34^ The consumption of TBR by canines and felines can result in diarrhea, vomiting, and cramps because of their slow metabolic rates; therefore, the maximum standard of TBR in feed in the EU was set at 300 mg/kg of livestock mass.^35,36^ In racehorses, TBR intake constitutes illegal doping of intentional excitement.^37,38^ TBR induces genetic mutations in canines and rodents; however, no adverse effects have been reported in humans. In the 20th century, TBR was used as an API for syphilis, cardiovascular disease, and diuresis; however, this has been discontinued. TBR, akin to allopurinol and febuxostat, is being assessed as a potential agent for preventing uric acid crystallization in nephrolithiasis and hyperuricemia.^39^

XAT (water solubility 0.5 mmol/L, log *P* = −0.73, p*K*a 7.52) is an endogenous purine metabolic intermediate oxidized to uric acid by XAT dehydrogenase and XAT oxidase. The crystalline structure of pure XAT requires further investigation.^40^ The aqueous insolubility of XAT and TBR compared with that of CFN and TPH can be attributed to several factors. The log *P* values of these XAT derivatives were not greater than zero, indicating their hydrophilicity. The topological polar surfaces^41^ of CFN, TPH, TBR, and XAT were 58.4, 69.3, 67.2, and 86.9 Å^2^, respectively. The partition coefficients and the solvent-accessible interfaces could not determine the aqueous insolubility of XAT and TBR. The *T*_m_ values of XAT and TBR were 640 and 573 K, respectively, which were higher than those of ambient-stable CFN (512 K) and TPH (546 K). According to Richards’ rule, the higher *T*_m_ values of XAT and TBR suggest a larger fusion enthalpy, making their crystalline solids thermodynamically stable and biased toward crystallization after dissolution.^42^ Schröder-Van Laar’s law, which is equivalent to Van’t Hoff’s reaction isobar, indicates a linear relationship between solubility and reciprocal temperature. This implies that the dissolution enthalpy for XAT and TBR was significant, which corresponded to their high fusion enthalpy.^43^ The formation of a crystalline lattice would occur under the regulation of polar substituents rather than hydrophilic solvation. Additionally, the imidazole N7–H of XAT and TPH provides diverse intermolecular interactions according to Etter’s rule; this favors the formation of molecular compounds.^28^

Given the potent agrypnode activity of these XAT derivatives, their intermolecular interactions with any APIs can be potent or perilous. As the balance between disease inhibition and central nervous stimulation varies among species, including humans and experimental animals, the individual activity of administered ingredients should be acknowledged. Therefore, this study aims to draw attention to the unexpected ingestion of XAT derivatives during the administration of APIs.

Nonsteroidal anti-inflammatory analgesics (NSAIDs), (S)-(+)-ibuprofen (IBP), and diclofenac (DCF) were selected as model APIs because of their limited specificity for CYP1A2 and the lack of clinical/pharmacodynamic interactions with XAT derivatives.^15−17^ As its enantiomeric conversion progresses intravitally, IBP is used clinically as a racemic mixture (*T*_m_ 348–351 K).^44^ However, the physicochemical purity of the enantiomeric IBP (*T*_m_ 325 K) is essential for assessing the intermolecular interactions and thermodynamic properties of XAT derivatives. NSAIDs inhibit cyclooxygenase (COX), synthesize autacoids, and induce inflammation and pain. They provide symptomatic anti-inflammatory and analgesic treatments, preventing the overuse or overdose of opiates, ketamine, and fentanyl for pain relief.^45^ The inhibition of COX1 by IBP can stimulate gastrointestinal mucous membranes, mitigated by antacids, such as prophylactics, albeit with potential bioavailability loss.^44−46^ DCF, which has low COX1 selectivity, reduces this adverse effect.^46^ COX2 inhibitors directly target inflammation but pose renal failure risks (DCF exhibits the worst effect). This has been shown in Asian raptors exposed to bioconcentrated COX2 inhibitors.^46^ Hypersensitivity reactions to NSAIDs (aspirin-induced asthma) vary among individuals and depend on drug responsiveness.^46^ A scientific consensus on the potential association between DCF and worsened COVID-19 infections is lacking.^47^ Despite their advantages and disadvantages, many individuals use NSAIDs (predominantly DCF and IBP), making them valuable model APIs.

## 2 Materials and Methods

### 2.1 Materials

CFN (CAS RN 58-08-2) was supplied by Fujifilm Wako Pure Chemicals (Osaka, Japan). TPH (58-55-9), TBR (83-67-0), XAT (69-89-6), IBP (15687-27-1), DCF (15307-86-5), DCF sodium salt (15307-79-6), LCMS grade methanol, high-performance liquid chromatography (HPLC) grade 1-octanol, and deuterated solvents (D_2_O, methanol-*d*_4_, and DMSO-*d*_6_) were obtained from Tokyo Chemical Industry (Tokyo, Japan). All the other materials and solvents were of analytical grade. The aqueous-phase solvents were prepared by mixing 100 mM KH_2_PO_4_ and 100 mM Na_2_HPO_4_ (100 mM P_i_ buffer). The pH was adjusted before fixing the prescribed concentrations of the acid components of the buffer.

### 2.2 HPLC Measurement of API Concentration in the Sample Solution

The sample solution was filtered with a membrane filter (Minisart RC 4 with 0.22 μm pore size; Sartorius, Göttingen, Germany). The fractionation of the API or XAT derivative in the filtrate was performed via HPLC (Shimadzu Co., Kyoto, Japan) using a mobile phase of 25 mM citric acid buffer (pH 3.0):methanol of 3:7 at a flow rate of 1 mL/min. A reversed-phase column (Capcell Pak C18; Shiseido; 5 μm, 4.6 mmφ × 250 mm) was mounted at a temperature of 313 K. The quantities of IBP, DCF, CFN, TPH, TBR, and XAT were determined by monitoring the absorbance at a wavelength of 275 nm.

### 2.3 The Solubility and Dissociation Rates of Sample Powders

To assess the apparent solubility of pure APIs in solutions of XAT derivatives, “pre-dissolution procedures” were conducted. Solutions containing 0, 10, 20, and 50 mM CFN or 0, 5, 10, 15, and 20 mM TPH in 100 mM phosphate buffer (pH 6.8) were prepared; TBR and XAT were excluded. To determine the apparent solubility of API mixtures with CFN, TPH, TBR, or XAT, “co-dissolution procedures” were performed using 100 mM phosphate buffer (pH 6.8). The API samples, adjusted to exceed saturation, were added to 5 mL of the dissolving medium and shaken in a water bath at 298 K for a specific duration. The concentration of API in the supernatant was measured using HPLC. The solubility concentrations of all APIs were determined from each API’s standard curves. The *R*-square values of all standard curves are over 0.995. The detection limit of IBP, which is the highest among the samples, is 0.4666 mM, and the quantitation limit of TPH, which is also the highest among the samples, is 2.645 mM.

The dissolution process of the pure APIs or API mixtures assumably corresponded to a linear correlation or a saturation curve. The API samples were homogenized using an agate mortar and pestle to align the particle sizes without sieving, considering the potential contamination of superfine powders. The immediate dissolution of the adhering powders in the solution at *t* = 0 was assumed; additionally, their concentrations were treated as the initial concentration (*C* = *C*_0_) if a sink condition was applicable owing to the higher solubility of the API.

For curves resembling a saturation curve, nonlinear curve fitting using the Noyes–Whitney integral expression (eq 1) was applied:^48,49^
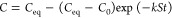
1where *C*_eq_ is the apparent solubility of the API, *k* is the dissolution rate constant, and *S* is the surface area of the sample particles. The determination of *S* was challenging, and excess powder in the equilibrium solution likely resulted in a uniform *S*. Therefore, regardless of the differences between the APIs and mixtures, a constant *kS* was used as the apparent dissolution rate constant. Curve-fitting procedures employed the Solver module of Microsoft Excel 2016 with the implemented GRG nonlinear option. The recrystallized precipitates were verified by X-ray powder diffraction (XRPD) and differential scanning calorimetry (DSC); no cocrystals or other generated solids were observed.

### 2.4 Thermal Analysis of Pure APIs and their Mixtures with XAT Derivatives

DSC was performed using a DSC8230 instrument (Rigaku Co., Tokyo, Japan) with a sample mass of 10.0 mg. Aluminum oxide was used as reference regent in all measurement. The sample was placed in an aluminum pan and sealed. Temperature scanning ranged between 303–543 K, 303–573 K, 303–633 K, and 303–603 K for CAF, TPH, TBR, and XAT, respectively. Scanning was performed at a rate of 10.0 K/min under a nitrogen gas flow rate of 30 mL/min.

The *T*_m_ was determined using Thermo Plus 2 software (Rigaku Co., Tokyo, Japan) by identifying the temperature at the maximum gradient on the left side of the peak curve, which was obtained from the intersection of the baseline extension and the tangent line. If the obtained curve displayed a simple endothermic peak, the area enclosed by the endothermic curve and the baseline was converted to the total melting enthalpy (Δ_fus_*H*) of the target component using compensation with the instrument coefficient. Additionally, the total melting entropy (Δ_fus_*S*) of the component was simultaneously approximated by dividing Δ_fus_*H* by *T*_m_ according to Clausius’ classical definition.

### 2.5 XRPD of Pure APIs and their Mixtures with XAT Derivatives

XRPD pattern measurements were conducted using a RINT 2000 instrument (Rigaku Co., Tokyo, Japan) equipped with a Cu Kα radiation source operating at 40 kV and 40 mA. Monochromatic radiation was produced by filtering the X-rays through a Ni filter, and measurements were performed using the parallel-beam method within a 2θ range from 5 to 40°; scanning was performed at a rate of 0.02° per step. Each spectrum represented an average of five scans, and the scanning sequences were performed in triplicate or more. Samples were prepared by crushing them in an agate mortar and pestle; thereafter, the powders were mixed.

DSC-XRPD, which involves simultaneous measurements of X-ray diffractometry and DSC, was conducted using an RINT 2000 instrument at a scanning rate of 10 K/min under a nitrogen gas flow rate of 40 mL/min. This technique utilized the thermal scanning stage unit, XRD-DSCII.^50^

The diffractograms of the single-crystal structures of the drug were validated against reproduced references generated from three-dimensional (3D) coordinates using the Reflex Module of Powder Diffraction on Biovia/Accelrys Materials Studio 2023 (Dassault Systems). The Miller indices of the prominent peaks were calculated using 3D crystalline coordinates retrieved from the Cambridge Crystallographic Data Centre (CCDC).

### 2.6 Attenuated Total Reflection-Fourier Transform Infrared (ATR-FTIR) Spectrometry

ATR-FTIR spectra were recorded using an FTIR spectrometer (PerkinElmer Co., Massachusetts) equipped with a universal attenuated total reflectance accessory. The samples were measured at wavelengths ranging from 4000 to 400 cm^–1^. A force of 100 N was applied to the sample at standard temperature. The spectra averaged 16 or more scans taken at 0.5 cm^–1^ resolution. The experiment was repeated at least twice for verification.

### 2.7 Singular Value Decomposition (SVD) Computation for Spectrometric Data and pH Profiles

The *i*-th spectrum  of the sample represents a *m*-dimensional vertical vector measured at a specific wavelength. The wavelength range spans from 250–500 nm (for example, for MLX) with an interval of 0.5 nm, resulting in *m* = 501. Matrix *M*, defined in eq 2, consists of a horizontal sequence of obtained spectral vectors (with dimensions *m* × *n* = 501 × 11).
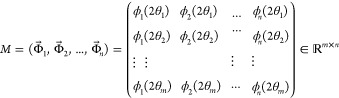
2

*M* and *M*^*t*^ represent the real and transposed matrices, respectively. The products *M^t^M* and *MM*^*t*^ formed orthogonal matrices. The matrices describing *M* can be transformed into eq 3:
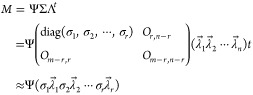
3

The matrix ∑ comprises the diagonal elements {σ_*i*_|1 ≤ *i* ≤ *r*} (positive real values ordered in descending order) representing singular values denoting dispersion. The *i*-th column of the orthogonal matrix Λ is the coefficient vector corresponding to the singular value σ_*i*_, and vector *λ⃗*_*i*_ is a specific singular vector. The rows of matrix Ψ are considered basis function vectors—the principal component vector *ω⃗*_*i*_ results from the product of *λ⃗*_*i*_ and the corresponding σ_*i*_.
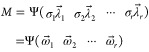
4

Matrix Ψ comprises rows that are basis function vectors. The SVD was applied to a 501 × 11 spectral data matrix. The dimensionality was determined based on the logarithm of the singular value against the index, establishing the minimum dimensionality required to replicate the vector space of the document spectrum. The minimum value of the dimensionality is almost negligible when the singular value is lower than a specified threshold (for example, several hundredths of the highest singular value). The chosen dimensionality, *r*, enables the principal components to approximately reproduce the vector space, including the documental spectrum as the *j*-th feature vector () composed of the *i*-th elements *x*_*i*,*j*_ as described in eq 5:^51−65^
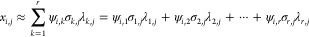
5

### 2.8 Nuclear Magnetic Resonance (NMR) Spectroscopy

^1^H NMR measurements were conducted using a 400 MHz NMR spectrometer (JNM-ECZ 400 S, Japan Electronics Co., Ltd., Tokyo, Japan). Sample solutions were prepared using D_2_O as the protic solvent and dimethyl sulfoxide-*d*_6_ (DMSO-*d*_6_) as the aprotic solvent, with sample concentration exceeding 1.5 w/v%. The chemical shifts of the samples were calibrated using the internal tetramethylsilane (TMS) signal as the zero point, and the solvent signals (4.800 ppm for D_2_O and 2.500 ppm for DMSO-*d*_6_) served as reference points. When sodium salts were required, the neutral NSAID species in D_2_O were dissolved in equimolar amounts of aqueous NaOH and ethanol-*d*_6_. Thereafter, the NSAID species were dried under reduced pressure in a rotary evaporator and heated to dryness below the *T*_m_ of the sodium salts. The formation of sodium salts was confirmed by measuring the *T*_m_ using DSC (DSC8230, Rigaku Co., Ltd., Tokyo, Japan).

^1^H–^1^H homonuclear correlation spectroscopy (COSY) involved scanning electromagnetic radiation pulses through hydrogen nuclei, eliciting responses from resonant hydrogen atoms with geminal, vicinal, or long-range coupling. The diagonal signal corresponded to the hydrogen response to the scanned radio waves at a specific frequency, whereas cross peaks that did not align with the diagonal signal revealed adjacent hydrogens. ^1^H–^1^H homonuclear Overhauser effect spectroscopy (NOESY) identified signals arising from hydrogen atoms in close spatial proximity, providing through-space correlations via spin–lattice relaxation. For optimal spectral assignment by NOESY, the mixing time should fall between half of *T*_1_ and *T*_1_, with increasing longitudinal relaxation time enhancing the sensitivity of NOESY. This can be achieved by selecting a low-viscosity solvent (such as acetone-*d*_6_) and removing the dissolved oxygen from the sample.

Diffusion-ordered spectroscopy (DOSY) was performed using the pulse-field gradient method at 303 K with a bipolar longitudinal eddy current delay (BPPLED) pulse sequence. The relaxation time was optimized to 7–14 s, with the duration of the pulsed field gradient and diffusion time adjusted to obtain a residual signal with a maximum field strength of 10%. The Stokes radius of the solute was calculated from the diffusion coefficient obtained using the Nernst and Einstein equation.
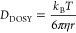
6where *T* represents the temperature (303 K), η is the viscosity (2.00 mPa s for DMSO-*d*_6_, and 1.25 mPa s for D_2_O), and *k*_B_ is the Boltzmann constant (1.381 × 10^–23^ J K^–1^).^51^

## 3 Results and Discussion

### 3.1 Dissolution Curves of the APIs into the Buffers Containing the XAT Derivatives (Pre-Dissolving)

Previous studies on the solubility of IBP (p*K*_a_ 4.45)^18^ have reported solubilities of approximately 40 mM in a 100 mM phosphate buffer (pH 7.18) and 20 mM in a 25 mM phosphate buffer (pH 6.88).^51−53^ These solubilities were represented by the equation 0.074 × (1 + 10^pH-p*K*a^), according to the Henderson–Hasselbalch equation. Mixtures of IBP with the basic drug lidocaine (LDC) reduced IBP solubility without pH fluctuations; the extent of modification depended on the experimental conditions—pH, temperature, and buffer composition.^51,52^ Similarly, DCF (p*K*_a_ 3.99)^18^ solubility in 100 mM phosphate buffer (pH 6.45) and 50 mM MES buffer (pH 5.5) were approximately 0.7 and 0.1 mM, respectively.^53−55^ These values corresponded to the equation—0.0027 × (1 + 10^pH-p*K*a^)—according to the Henderson–Hasselbalch equation. Addition of 10 mM LDC to the dissolution buffer increased the solubility of DCF within the buffering capacity range.^52^

**Figure 1:**
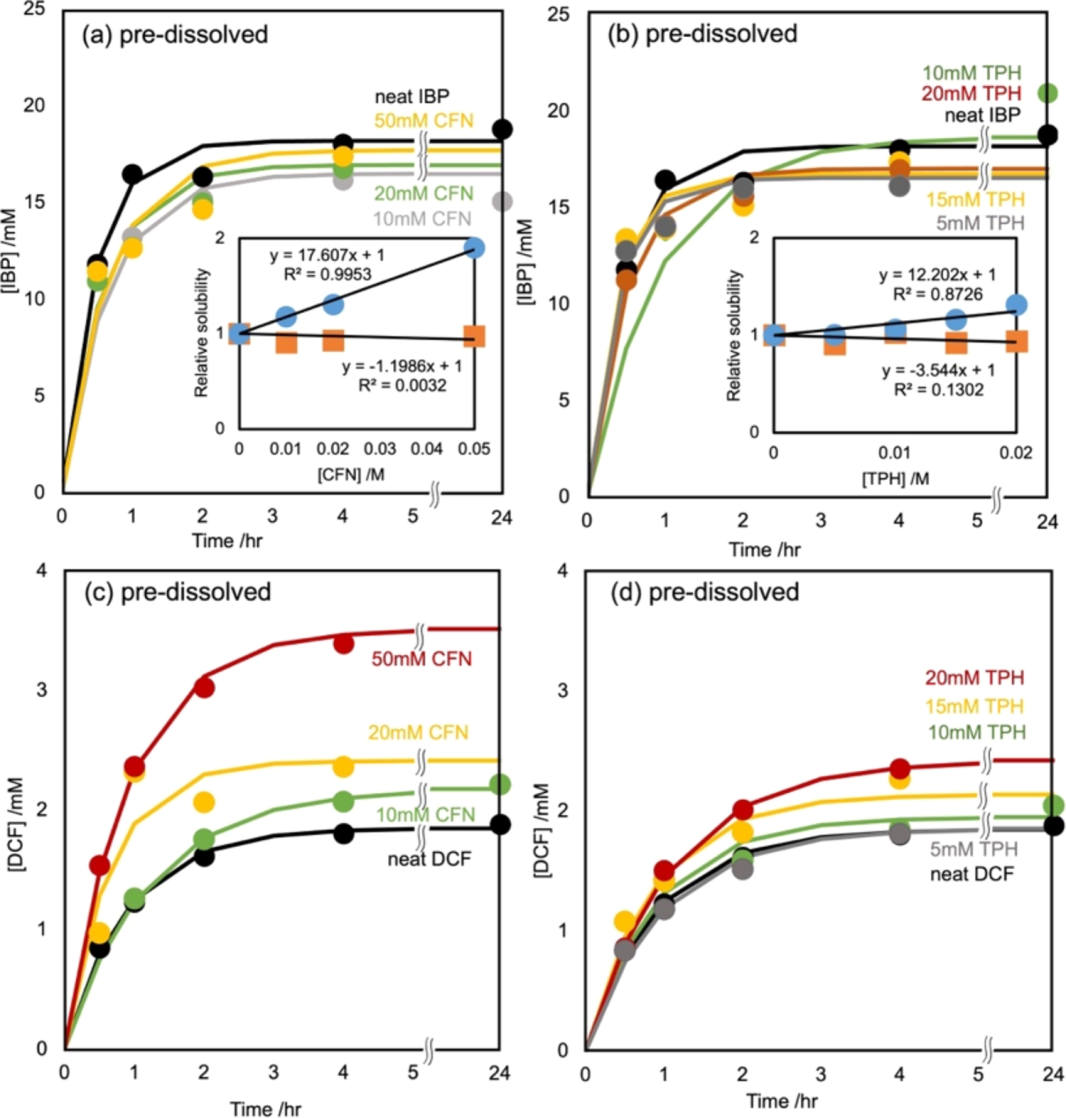
Effects of the predissolved CFN (a) and TPH (b) on IBP dissolution and those of the predissolved CFN (c) and TPH (d) on DCF dissolution. Dissolution curves were fitted using the Noyes–Whitney equation with the dissolution rate constant term *kS*, in which the solid surface area parameter *S* was assumed to be invariable in dissolution to the equilibrium. The insets in (a) and (b) show the relative solubility of IBP (squares) and DCF (circles) as a function of the initial concentrations of CFN and TPH. The predissolved CFN and TPH did not influence the saturated concentration of IBP.

In this study, the solubility of IBP and DCF in the presence and absence of CFN and TPH was assessed. CFN and TPH were used as replacements for LDC. Figure 1a,b shows the effect of predissolved CFN and TPH in 100 mM phosphate buffer solutions (pH 6.8) on IBP dissolution. The saturation curves of IBP in the presence and absence of CFN and TPH were not significantly different. Furthermore, pH remained constant throughout the dissolution process. Analysis of the obtained dissolution curves facilitated the estimation of the saturated concentration of IBP using curve fitting eq 1. The resulting IBP saturated concentrations, irrespective of CFN and TPH, were 17.22 ± 0.61 and 17.34 ± 0.86 mM. This aligned with the solubility equation previously described.

The saturated concentrations of the dissolution curves of DCF in the predissolved CFN and TPH solutions were higher than those of the curve in the absence of CFN and TPH (Figure 1c,d). The pH was constant during DCF dissolution. Based on eq 1, the solubilities of DCF in the absence of CFN and TPH were 1.759 ± 0.044 and 1.686 ± 0.053 mM, respectively. The insets of Figure 1a,b show the relative solubilities of IBP and DCF with respect to the saturated concentrations of CFN and TPH. CFN and TPH did not influence IBP solubility but enhanced DCF solubility, indicating that CFN and TPH act as hydrotropes (solubilizers) for DCF but are unrelated to IBP. In addition, the solubility of indomethacin (INM) was enhanced by the addition of CFN to the dissolving medium (data not published).

### 3.2 Dissolution Curves of the API Mixtures with the XAT Derivatives into the Buffer (Co-Dissolving)

A previous study investigating the interaction between DCF and basic additives reported that the presence or absence of cimetidine (CIM) in the solution had an insignificant influence on the solubility of DCF (0.829 mM) and INM (0.625 mM) during the predissolution procedure. However, the simultaneous addition of CIM crystal powder with DCF or INM increased the solubility of DCF (3.39 mM) and INM (3.34 mM) during the codissolution procedure. Similar effects were observed with imidazole and arginine. Conversely, famotidine (FAM) delayed the dissolution of DCF; however, the final DCF solubility was not affected by the presence of FAM in the equilibrated solution, despite the effective interaction kinetics between DCF and FAM.

**Figure 2:**
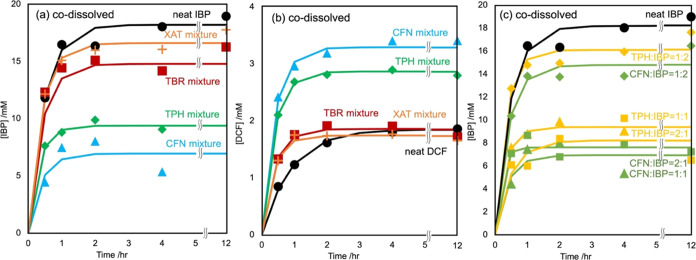
Effects of the codissolved CFN (triangles), TPH (diamonds), TBR (squares), and XAT (crosses) in the IBP or DCF equimolar mixtures in the IBP (a) and DCF (b) dissolutions. Dissolution curves were fitted using the Noyes–Whitney equation with the dissolution rate constant term *kS*, in which the solid surface area parameter *S* was assumed to be invariable in dissolution to the equilibrium. The codissolved CFN and TPH decreased and increased the saturated concentrations of IBP and DCF, respectively. TBR and XAT exhibited limited influence on their solubility. (c) Effects of CFN and TPH codissolved proportions in the IBP mixtures on the IBP dissolutions. The codissolved CFN and TPH at the equimolar and double proportions attenuated the saturated concentrations of IBP; however, those at the half equivalences were ineffective. An equivalent or higher amount of CFN and TPH restricted the release of IBP from its solid phase.

In this study, the effects of the simultaneous addition of CFN and TPH on the solubility of IBP and DCF during the codissolution procedure were assessed. Equimolar mixtures of IBP or DCF with CFN, TPH, TBR, or XAT were added to 5 mL of 100 mM phosphate buffer (pH 6.8). Figure 2a shows the IBP dissolution curve of its mixtures with the XAT derivatives measured under the same conditions as the predissolution procedures. Compared with the results observed in the predissolving procedures, mixing CFN and TPH decreased the solubility of IBP.

Varying the proportions of mixed CFN and TPH showed that mixing twice the amount of IBP with CFN and TPH resulted in a similar behavior similar to that of the dissolution of IBP alone (Figure 2c). Conversely, mixing half the amount of IBP with CFN and TPH resulted in dissolution behavior identical to that of an equimolar mixture. This suggests that IBP forms equimolar complexes with CFN and TPH, which controls the amount released into the aqueous solution. TBR and XAT did not significantly affect the IBP dissolution curve.

Figure 2b shows the dissolution curves of DCF in mixtures with XAT derivatives. Mixing with CFN or TPH increased the saturated concentration of DCF, which was consistent with the results obtained in the predissolving procedures. CFN and TPH enhanced DCF dissolution as hydrotropes. These results indicate that a significant delay in the dissolution rate was not observed; therefore, CFN and TPH are unlikely to inhibit DCF dissolution. The dissolution curves in the presence and absence of TBR and XAT did not significantly differ for saturated concentrations of IBP and DCF.

These findings suggest that the solubility of additives (CFN and TPH) plays a crucial role in the hydrotropic effects or solubility attenuation. However, comparative experiments for TBR and XAT were not feasible because of their poor aqueous solubilities.

**Figure 3:**
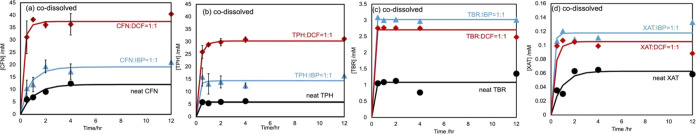
(a, b) Effects of IBP and DCF on the solubility of the codissolved CFN (a) and TPH (b) in their equimolar IBP or DCF mixtures. Dissolution curves were fitted using the Noyes–Whitney equation with the dissolution rate constant term *kS*, in which the solid surface area parameter *S* was assumed to be invariable in dissolution to the equilibrium. Adding IBP into their equimolar CFN and TPH mixtures increased the saturation concentrations of the XAT derivatives. The effect of DCF was two or three times greater than that of IBP. (c, d) Effects of IBP and DCF on the codissolved TBR (c) and XAT (d) solubility in their equimolar IBP or DCF mixtures. Dissolution curves were fitted as described in the caption of Figure S1. Adding IBP and DCF into their equimolar TBR and XAT mixtures induced the saturation conditions of the XAT derivatives. The solubility of pure TBR was measured as 1 mM. However, the results suggested that the coadministration of TBR and NSAIDs increased the solubility of TBR by 3-fold. IBP and DCF increased the solubility of XAT by 2-fold; however, the absolute concentration level and biological influences were negligible.

The dissolution curves of XAT derivatives in equimolar mixtures of IBP and DCF were examined. An increase in the saturated concentration of each XAT derivative when mixed with the APIs was observed (Figure 3). Although TBR and XAT were not significantly involved in the solubility of IBP and DCF, their effects were not expected owing to their poor solubility. In contrast, DCF solubility was enhanced by predissolving CFN and TPH in the buffer and by mixing CFN and TPH with solid DCF. This suggests that intermolecular interactions between DCF and CFN or TPH occur in solution or during dissolution.

Although predissolving CFN or TPH in the buffer retained IBP solubility, mixing them into the solid hindered the release of IBP from the crystal. Therefore, the attenuation of IBP solubility caused by mixing CFN or TPH was not likely due to the formation of equimolar and insoluble complexes of IBP with XAT derivatives after dissolution. However, CFN and TPH selectively regulate solute release from IBP crystals.

### 3.3 DSC Curves of the Pure APIs and their Mixture with the XAT Derivatives

**Figure 4:**
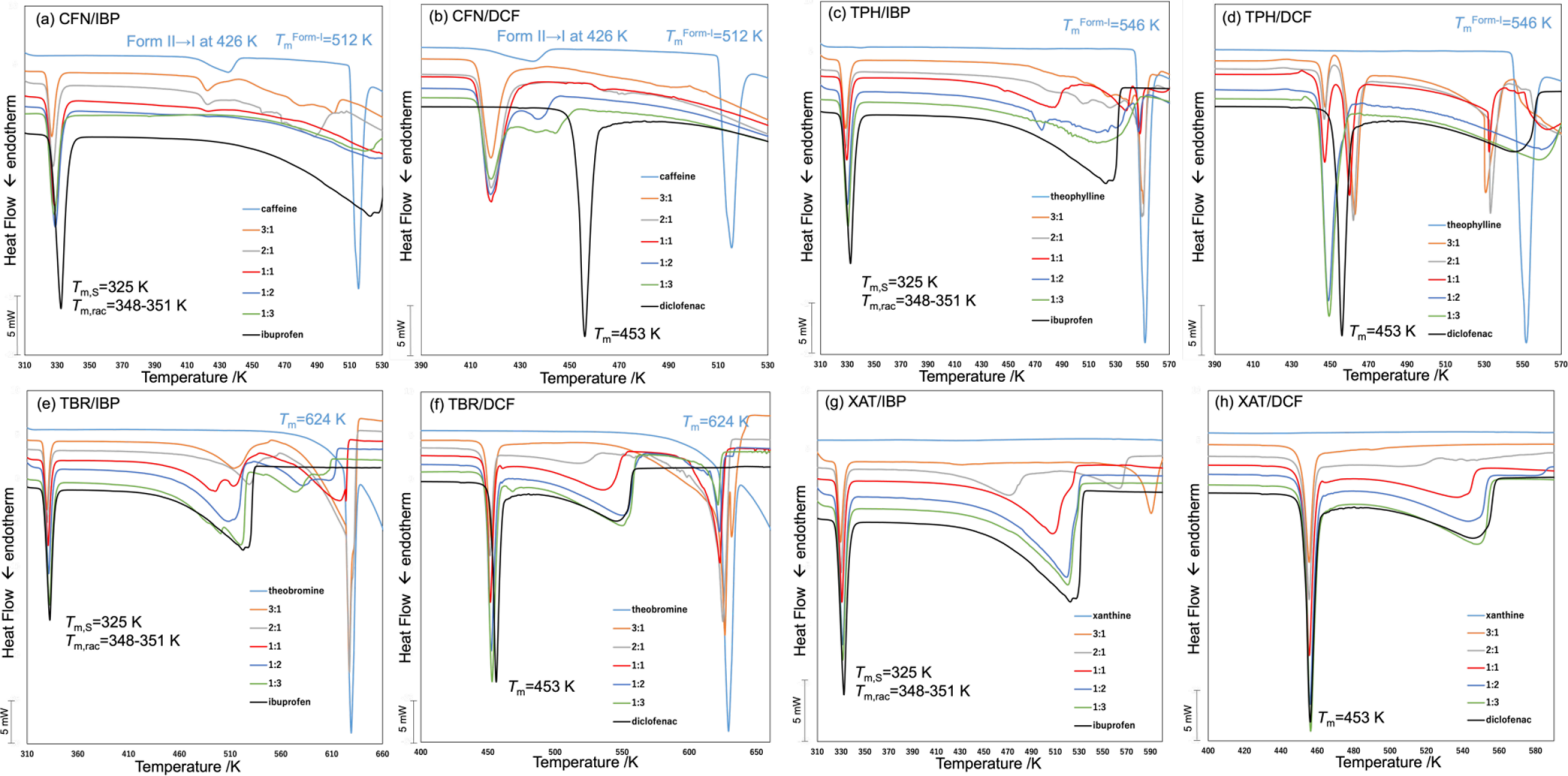
DSC curves of the CFN/IBP (a), CFN/DCF (b), TPH/IBP (c), TPH/DCF (d), TBR/IBP (e), TBR/DCF (f), XAT/IBP (g), and XAT/DCF (h) mixtures at various molar ratios. Pure XAT derivatives (sky blue), pure APIs (black), and their mixtures at ratios of 3:1 (orange), 2:1 (gray), 1:1 (red), 1:2 (blue), and 1:3 (green). IBP produced an endothermal signal at 52 °C, indicating the sample was an enantiomer (a, c, e, g). Polymorphs of CFN anhydrous crystals contained Form I (melting point = 237 °C) and Form II (phase transform to Form I at 160 °C for intact crystals and at 153 °C for ground powder) (a, b). Polymorphs of TPH anhydrate crystals contained Form I (melting point = 273 °C) and Form II (melting point = 269 °C) (c, d). The *T*_m_ of the pure TBR was 315 °C (e, f), whereas the pure XAT was not verified under these experimental conditions (g, h). Shifts were not observed in the endothermic signals of IBP (e, g) and DCF (h) in the mixtures at these molar ratios; however, nonzero shifts were observed in the DCF mixtures of pure DCF (f).

Thermal analyses of the mixtures and pure crystals were conducted using DSC to evaluate the intermolecular interactions between the APIs and XAT derivatives. Figure 4 shows the curves of the mixtures of CFN (a and b), TPH (c and d), TBR (e and f), and XAT (g and h) with APIs at mole fractions of 1:0, 3:1, 2:1, 1:1, 1:2, 1:3, and 0:1; the ratios of 1:0 and 0:1 corresponded to the pure XAT derivative and pure API, respectively. The expected starting *T*_m_ of the IBP enantiomer was approximately 325 K; however, greater discernibility of relative temperatures was observed at the top of the peak, particularly at equal scanning rates.

The top of the IBP fusion peak was observed at 332 K and a scanning rate of 10 K/min (Figure 4a). Regarding the pure CFN, an endothermic peak corresponding to the form II/I phase transition signal was detected at 433 K, whereas the peak for the form I fusion signal was observed at 510 K. The top of the IBP melting signal in mixtures containing IBP was reduced by 4–7 K depending on the mole fraction of CFN. This attenuation of the IBP melting peak was sensitive to the mole fraction of CFN, with equimolar or CFN-higher mole fraction mixtures consistently exhibiting an intersection of the left-side tangent line of the endothermic peak on the baseline extension. The top of the form II/I phase transition and form I fusion CFN signals decreased to 421 and 487 K, respectively, until an equimolar mixture was reached; the corresponding endothermic signals were less pronounced in the mixtures that contained lower CFN fractions. An equimolar mixture exhibited the most efficient interaction between IBP and CFN.

The top of the peaks (and their left intersections) of the equimolar and CFN-higher mole fraction mixtures of DCF were reduced by more than 37 K (Figure 4b). The intermediate peaks observed in the mixtures containing higher DCF mole fractions showed that there was diversity in the DCF crystal phase. The diversity exhibited in the curves of the DCF/TPH mixtures will be discussed in the following section. The phase-transition and melting peaks of CFN shifted to lower temperatures because of the blend, suggesting that the DCF crystal was accessible to the CFN crystal.

Similar trends were observed in the curves of the IBP mixtures with TPH, whereas more complex peaks emerged in the curves of the DCF mixtures with TPH (Figure 4c,d). The *T*_m_ of TPH decreased by approximately 11 K in an equimolar mixture with IBP. However, an endothermal signal at a temperature higher than the *T*_m_ of pure DCF was simultaneously observed in the curves of equivalent or excess mole fractions of TPH in the TPH/DCF mixtures. An exothermal signal was observed between the peaks at 445 and 459 K, indicating that the stable HD2 form of DCF melted and promptly transformed into a high-*T*_m_ polymorph (or cocrystal with TPH).^56^

The top of the IBP and DCF peaks decreased by 2–5 K depending on the mole fraction of TBR or XAT in the mixtures; however, the left intersections of these peaks remained constant (Figures 4e–h, and S1). Mixing DCF and TBR resulted in a *T*_m_ value that marginally differed from that of pure. However, the *T*_m_ values of IBP and DCF were assumably not affected by TBR or XAT. These results are consistent with those of the codissolution experiments: CFN and TPH enhanced DCF solubility and reduced IBP solubility, whereas TBR and XAT had negligible effects on the dissolution of IBP and DCF.

### 3.4 DSC-XRPD Experiments for the API Equimolar Mixtures with CFN or TPH

**Figure 5:**
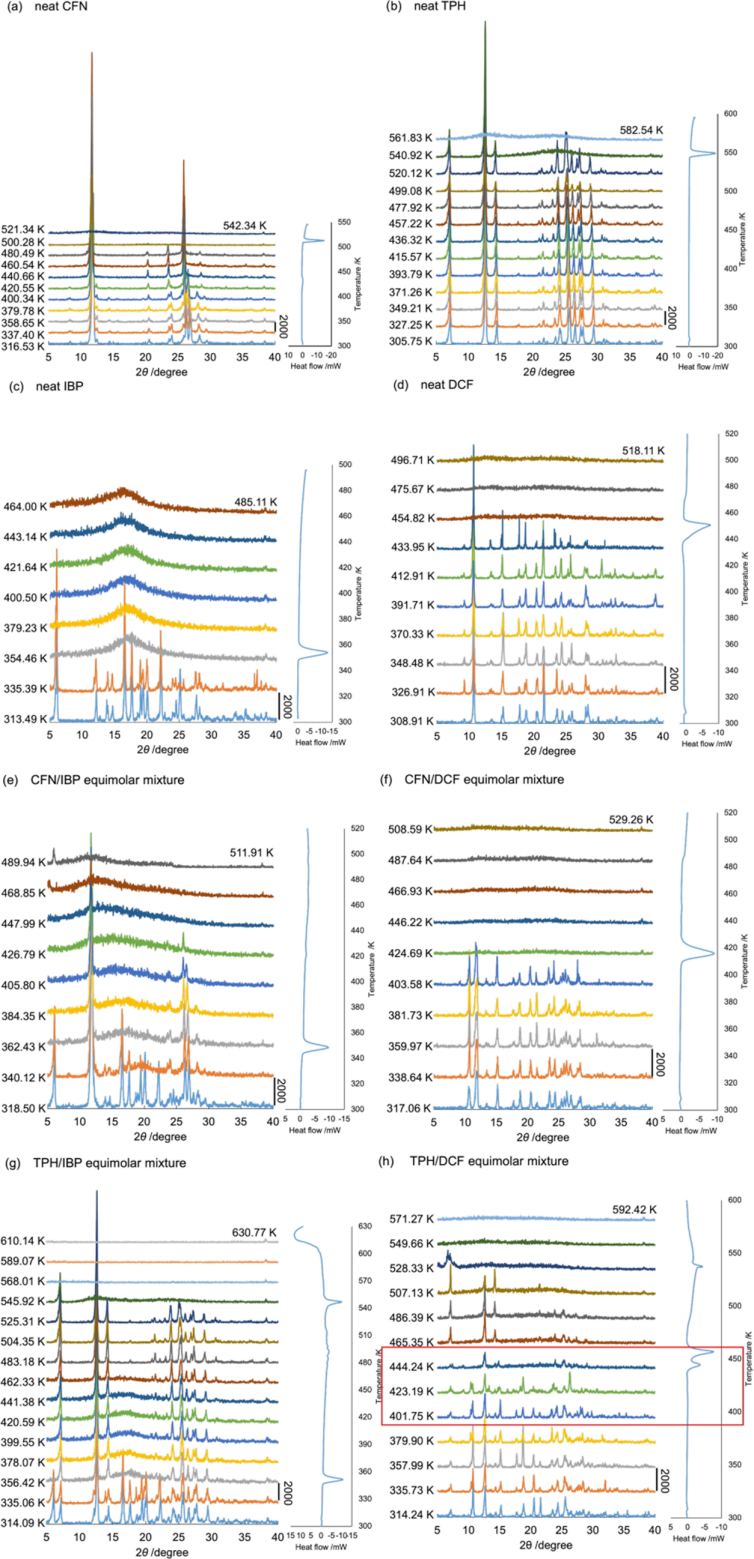
DSC-XRPD diffractograms of pure CAF (a), TPH (b), IBP (c), and DCF (d), as well as those of CFN/IBP (e), CFN/DCF (f), TPH/IBP (g), and TPH/DCF (h) equimolar mixtures. Temperature scanning was performed at a rate of 10 K/min.

DSC-XRPD experiments were continuously conducted on mixtures of APIs with XAT derivatives. Figure 5 shows the temperature-scanning diffractograms of pure CFN (a), TPH (b), IBP (c), and DCF (d) and their equimolar mixtures. Signals at 12.5, 19.8, 24.4, 26.5, 27.9, and 29.1° were not observed; however, signals at 23.6 and 28.5° were observed at a higher temperature than that of the form II/I transformation (426 K) (Figure 5a). Subsequently, a halo pattern of CFN was observed at a higher temperature than the *T*_m_ of form I (512 K). Conversely, simple signals of TPH and IBP were confirmed at their respective *T*_m_ values. The diffractograms of pure DCF were consistent with the pattern of polymorph HD2 and transformed into a halo pattern at the *T*_m_.^57^ The signal heights at 17.7, 23.5, and 27.9° exhibited different patterns depending on the rising temperature, suggesting that DCF switched its crystalline habits in response to each temperature level. Figure 5e shows the DSC-XRPD diffractograms of the CFN/IBP equimolar mixture, where the diffraction signals of IBP and CFN were not observed at 358 and 420 K, respectively. Figure 5g shows similar results for the TPH/IBP equimolar mixture.

The signal pattern of the CFN/DCF equimolar mixture changed to a pattern containing amplified signals at the two-by-theta values of 10.7 and 21.5° after reaching a temperature of 408 K (Figure 5f); the CFN signals were absent at this stage. These two signals were assigned to the HD2-form signals of DCF, which differ from the diffractions of the other polymorphs, HD1 and HD3.^57^

A previous study used various solvents for recrystallizing DCF to prepare crystals with lower (in 2-propanol) and higher (in acetonitrile) *T*_m_ than the purchased pure DCF and crystals prepared in diethyl ether, acetone, chloroform, and ethanol.^57^ However, the XRPD signals of these crystals were identical to those of the HD2 crystals, suggesting that they did not exhibit new crystalline habits with similar *T*_m_ values. Lai et al. reported different HD2-polymorphic crystals as DCF1 (*T*_m_ 454 K) and DCF2 (*T*_m_ 449 K) on their notation.^66^ Although DCF may involve diverse habits with different *T*_m_ values of polymorph HD2, they did not compare and verify their individual habits using single-crystal diffraction. The exothermal signal between the 445 and 459 K peaks (Figure 4d) indicates a requirement for an unidentified “phase” transition between these habits.

Figure 5h shows the results of DSC-XRPD analysis conducted at temperatures ranging from 445 to 510 K; the presence of diffractograms was primarily attributed to TPH. Compared with the lower-temperature patterns, variations in the diffraction patterns were observed at temperatures between 424 and 445 K; additionally, minor signals were detected at 16.2 and 23.7°. These signals were not consistent with those reported for other polymorphs such as HD1 and HD3 (Figure S2). DCF demonstrated the ability to adopt suitable habits/states under different thermodynamic and chemical conditions when used alone or in combination with CFN or TPH. Additionally, TPH exhibited two polymorphs with identical diffractograms, potentially leading to distinctions in the DSC-XRPD patterns between the CFN/DCF and TPH/DCF mixtures within the 424–445 K temperature range.

Moreover, TBR and XAT exhibited negligible interactions in the DSC-XRPD experiments when mixed with IBP and DCF.

### 3.5 ATR-FTIR Spectra of the Pure APIs and their Mixtures with the XAT Derivatives

To investigate the intermolecular interactions between the XAT derivatives and APIs, ATR-FTIR spectra of the powder mixtures prepared under various conditions were analyzed. The FTIR spectra of the pure XAT derivatives (Figure S3) exhibited characteristic stretching vibrational signals, such as purine C8–H of CFN, TPH, and TBR, and amido-H of TPH, TBR (amide–II band), and XAT (containing amide–II band) within the 3200–2300 cm^–1^ range. The dicarboxyimide C2=O and amide–I C6=O stretching vibrational signals were observed at 1645, 1661, 1661, and 1645 cm^–1^ for CFN, TPH, TBR, and XAT, respectively. Distinctive signals for XAT derivatives were detected between 1000–400 cm^–1^ that were attributed to deforming vibration modes (involving wagging, rocking, and twisting) around the N–H moieties because of fewer methyl substituents in the XAT derivatives.

The FTIR spectra of pure IBP and DCF exhibited characteristic stretching vibration modes for the terminal methyl and branching C–H bonds of IBP at 2953, 2955, and 2870 cm^–1^, as well as a COOH signal at 1700 cm^–1^.^67^ Detailed information on middle-infrared signals of DCF was limited.^68^ Normal mode analysis of the thermal vibrations using molecular orbital computations provided simulated spectra corresponding to the observed signals. The signals at 3323 and 1690 cm^–1^ corresponded to the simulated signals for N–H bond stretching (3259 cm^–1^) and the COOH group (1722 cm^–1^), respectively. Additionally, the signals at 1287 and 1247 cm^–1^ were induced by the deformation vibrations of the acetic acid moiety (1304 and 1273 cm^–1^), and the signals at 1545 and 1390 cm^–1^ were attributed to the 2,6-dichloroaniline moiety (1578 and 1452 cm^–1^). The triplet pattern at 1578 cm^–1^ was characteristic and isolated but weak (the strong ν_C=N_ signal of the XAT derivatives will overlap on the triplet pattern). The dipole moment of the vibrational modes of the triplet pattern (those of ν_C–Cl_ and δ_C–Cl_ with significant inertia moment were lower than expected) was likely reduced; additionally, its detectability was lower in the FTIR spectrum than its detectability in the Raman spectrum. These observations suggested characteristic vibration modes in the phenylacetic acid moiety of DCF, with fewer detectable signals in the 2,6-dichloroaniline moiety in the FTIR spectrum.

Mixtures of XAT derivatives with IBP or DCF were prepared at various molar ratios and allowed to stand undisturbed at room temperature in the dark for up to 7 days. The ATR-FTIR spectra of these mixtures, depicted in Figure S5, exhibited gradual changes depending on the molar ratio of the XAT derivatives to the APIs. Despite expectations from the dissolution experiments, the spectra of the mixtures of CFN and TPH did not qualitatively exhibit absorption bands like those of TBR and XAT. Additionally, the differences between identical spectra of the sample lots were not negligible. Consequently, quantitatively distinguishing the extent to which the spectra deviated from the combination of the spectra of mixed components was challenging.

### 3.6 SVD Computation of FTIR Spectra to Analyze the Effects of the XAT Derivatives on the APIs

The analysis employed SVD and eigenvalue decomposition (EVD), also known as principal component analysis (PCA), to extract spectral similarities and represent the given spectra as a linear combination of these patterns. The EVD prioritizes major similarities and extracts orthogonal compositions that represent subsequent similarities.^69^ These high-order compositions are linearly independent of the lower ranks. Given that spectrometric data often exhibit co-occurrence (dependence) in individual signals due to intertwined absorptions (or emissions) from structural components, the SVD method efficiently accounts for this co-occurrence between similarity patterns.^59^ SVD has been successfully applied to derivative spectra of various spectroscopic techniques, such as FTIR derivative spectra,^58^ UV–visible spectra,^59,60^ far-UV circular dichroism (CD) spectra,^61^ powder X-ray diffractograms,^54−56,62^ electron spin resonance (ESR),^63^ fluorescence spectra,^64,65^ and curves,^56^ to quantify specific contributions of experimental conditions.

In this study, FTIR spectra of IBP mixtures containing XAT derivatives were analyzed using the SVD method. Raw spectra were used instead of derivative spectra (suitable for recognizing spectral patterns^58^) to maintain a rational stoichiometric intensity (depending on the molar ratios) according to the Beer–Lambert equation for IR absorption. The results of the SVD analysis of the IBP mixtures with XAT derivatives are illustrated in Figure S6, where 25 spectra (averages of two measurements for six mixtures per XAT derivative and one pure IBP; Figure S5) were used. The cumulative sum of the variances at the fifth obtained singular value reached 91.0%. Figure S6b shows the basis functions from the first to sixth compositions. Each basis function (ψi) represents one of the given FTIR spectra. The first composition was similar to the spectrum of pure IBP, indicating that it contains average/common information for the entire data set, primarily representing the spectrum of IBP owing to its high contribution.

Figure S6d,e shows the positive pattern of the second composition basis function (ψ2) and the negative patterns of the third composition basis function (ψ3), which align with the measured spectra of TBR and XAT, respectively. Figure S6f,g shows the negative patterns of the fourth and fifth composition basis functions (ψ4 and ψ5), containing signals from the measured spectra of CFN and TPH, respectively. These successful assignments facilitated broad classifications of the differences in the intermolecular interactions between the XAT derivatives and IBP.

The SVD computations yield singular vectors (λ) as coefficients corresponding to basis functions in their linear combinations, with components in λ indicating the contribution of each basis function relative to the molar fraction of the mixture of XAT derivatives. Components λ2 to λ5 were divided by λ1 to emphasize the properties featured in individual XAT derivatives. Figure S6i–l shows the significant continuous changes in relative components λ4/λ1 and λ5/λ1 that were based on the mole fractions of TPH and CFN, respectively. Furthermore, λ2/λ1 decreased as the TBR mole fraction increased, whereas λ3/λ1 varied with a change in the mole fraction of XAT. These profiles were consistent with previous assignments using basis functions, indicating that the second and third components involved the XAT and TBR proportions, respectively; however, the fourth and fifth components contained information about the CFN and TPH proportions.

**Figure 6:**
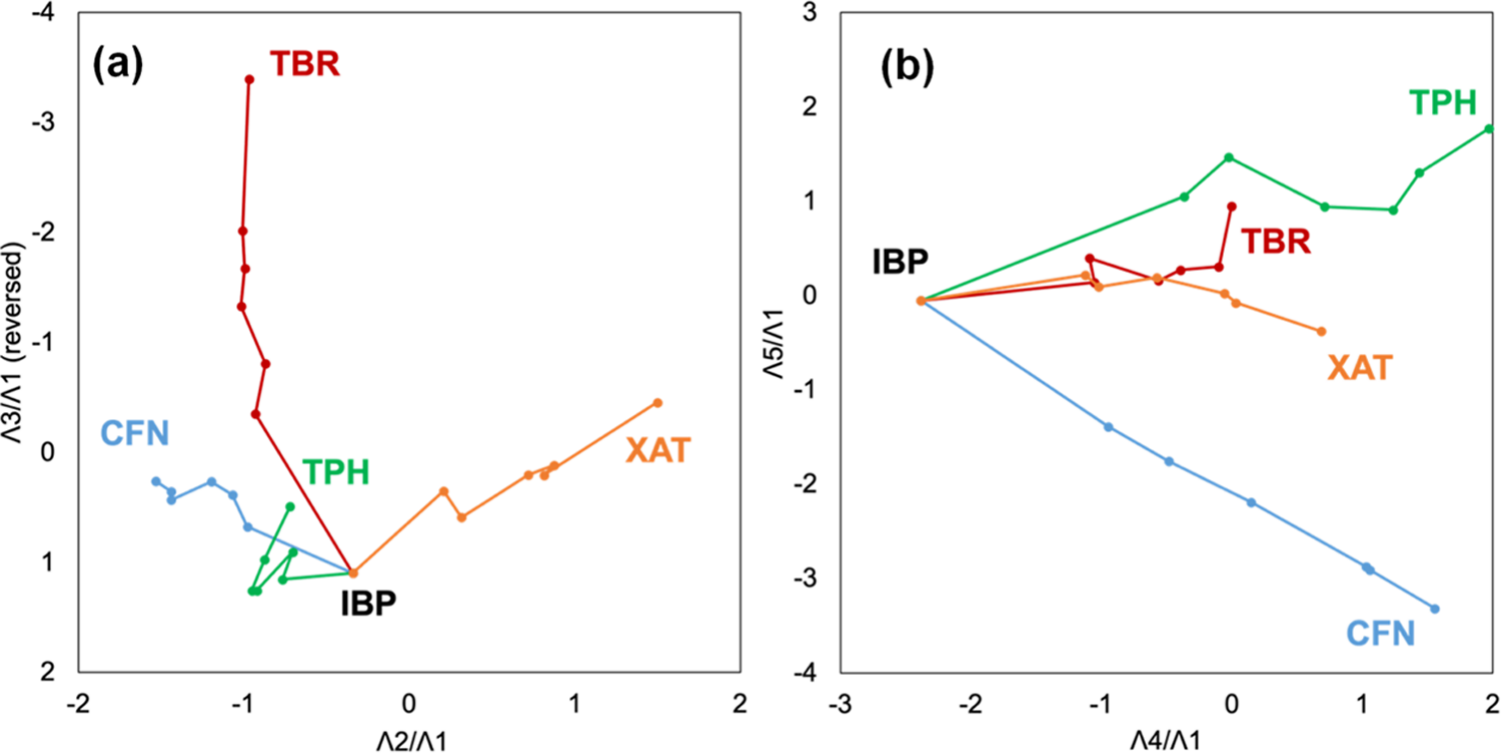
Trajectory of the IBP mixtures containing CFN (blue), TPH (green), TBR (brown), and XAT (orange). The trajectory in the (λ2/λ1)–(λ3/λ1) diagram (a) and the (λ4/λ1)–(λ5/λ1) diagram (b).

To demonstrate the trajectory of singular vectors for IBP mixtures with XAT derivatives, these trajectories were projected on (λ2/λ1)–(λ3/λ1) or (λ4/λ1)–(λ5/λ1) diagrams (Figure 6).^60−62^ Spatial awareness of the orientations of these trajectories were improved by including the animated movies of the 3D projections of the hyper-dimensional trajectories on the (λ2/λ1)–(λ3/λ1)- (λ4/λ1), and (λ2/λ1)–(λ3/λ1)- (λ5/λ1) spaces in the Supporting Information. The branch point in the trajectories indicates the vector of pure IBP. Additionally, spectral changes in the IBP mixtures were indicative of the stretch or tangle of the trajectories toward pure CFN, TPH, TBR, and XAT. Mixtures with TBR and XAT exhibited more distinct and continuous changes compared with those of CFN and TPH in the (λ2/λ1)–(λ3/λ1) diagram.

Trajectories for the IBP mixtures with CFN and TPH were tangled; however, they expanded along the (λ4/λ1)-axis and separated along the (λ5/λ1)-axis. Because the λ_i_ components were normalized, their absolute intensity on these projections was inconsequential; the corresponding singular values represented the trajectory weight. Because the second and third singular composition values were prominent, the spectral changes in the IBP mixtures with TBR and XAT were significant and sensitive to their mole fractions, suggesting no independent intermolecular interactions between IBP and TBR or XAT—their FTIR spectra changed only in proportion to the molar fractions of TBR and XAT. The SVD computations of CFN and TPH divided the mixing effects into minor signal changes, which shared their partial contributions.

**Figure 7:**
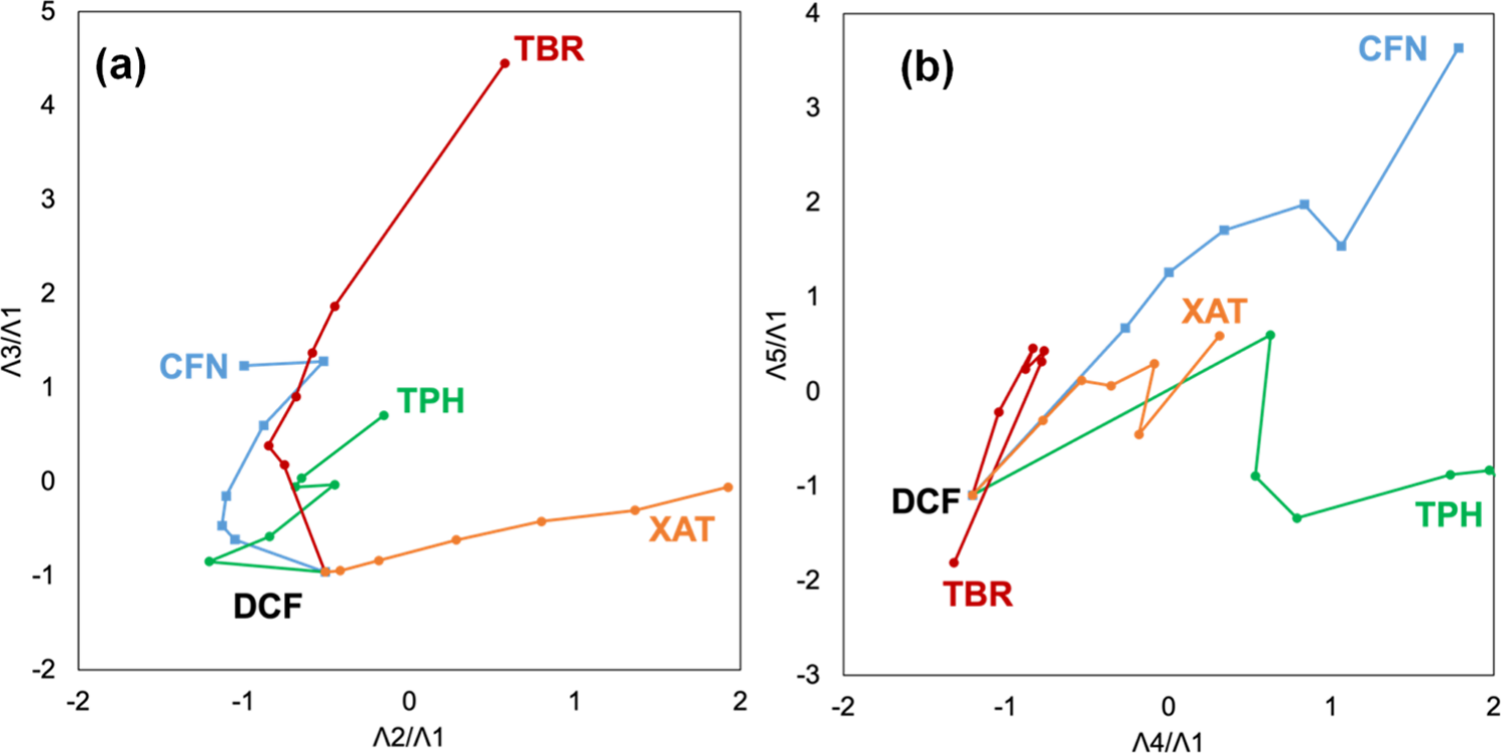
Trajectory of the DCF mixtures containing CFN (blue), TPH (green), TBR (brown), and XAT (orange). The trajectory in the (λ2/λ1)–(λ3/λ1) diagram (a) and the (λ4/λ1)–(λ5/λ1) diagram (b).

Following a similar analytical approach, DCF mixtures with XAT derivatives were analyzed, yielding comparable results (Figure S7). CFN and TPH, which significantly contributed to λ4, were effectively differentiated along the intrinsic hyper-dimensional direction by aligning onto the (λ4/λ1)-axis in both two-dimensional mappings (Figure 7). Regarding the DCF mixtures with XAT derivatives, singular vectors with higher singular values represented a combination of pure TBR or XAT spectra, whereas those with lower singular values were indicative of complex interactions of DCF with CFN or TPH.

This section describes the dependency of the spectral perturbations of IBP and DCF mixtures on the molar ratios of each XAT derivative. Despite certain proportions of the API and XAT derivatives exhibiting characteristic spectral properties, SVD analysis showed distinct compositions in the basis functions (from sixth to tenth) for the four different XAT derivatives. However, ATR-FTIR spectra of XAT derivatives were similar to the region between 1700–1100 cm^–1^ (Figure S3).

The spectral patterns of TPH and TBR were similar between 3200–2300 cm^–1^ and the fingerprint region (1100–400 cm^–1^). Further examination to discern departures from the linear correlation between the API and each XAT derivative is expected to show spectral deviations due to intermolecular interactions. However, the lack of diversity in the XAT derivative spectra resulted in contamination of these deviations. Identifying concerted spectral changes in API mixtures was challenging owing to the eccentric distribution of similar XAT derivative spectra.

### 3.7 Analyzing the Effects of the APIs on the XAT Derivatives Using SVD Computation

The spectra of the IBP and DCF mixtures with single XAT derivatives at various molar ratios were subjected to SVD computations utilizing the data sets from Figure S5a–h. The IBP and DCF spectra were adequately distinguished (Figure S6); therefore, the interactions with the XAT derivatives yielded no standard signal changes excluding the 1700/1690 cm^–1^ signal of the COOH stretching vibration modes.

The singular values and base vectors obtained from SVD analysis for the CFN/IBP and CFN/DCF mixtures are shown in Figure S8a,b. The top five singular values (cumulative sum of variance of 97.6%) were separated from lower plots. The negative (ψ1(−)) and positive (ψ2(+)) patterns of basis function contained signals observed in pure CFN spectra (Figure S8e–f,h). The spectra of pure DCF and IBP were assigned to ψ2(−) and ψ3(−), respectively (Figure S8g,i). The spectra of pure DCF and the CFN/DCF mixtures aligned with ψ4(−), whereas ψ5(+) corresponded to the CFN mixture (Figure S8j–m).

The components of the singular vectors of the CFN mole fraction in CFN/IBP or CFN/DCF mixtures are shown in Figure S8n,o. Within these components, λ3 and λ2 increased depending on the CFN mole fraction in the CFN/IBP and CFN/DCF mixtures, respectively. λ5 and λ4 increased in the CFN/IBP and CFN/DCF mixtures when the CFN mole fraction was 0.5 and lower than 0.5, respectively.

Figure S8p shows the (λ2/λ1)–(λ3/λ1) diagram, where intensities of (λ2/λ1) and (λ3/λ1) correspond to DCF and IBP mole fractions in the CFN/DCF and CFN/IBP mixtures, respectively. The linear combination spectra with the first and second basis functions of the SVD computation for the CFN/DCF mixtures to the measured CFN and DCF spectra are shown in Figure S8r,r’. Figure S8s,s’ shows the reconstructions with the first, second, and third basis functions. Two or three compositions can represent the CFN/DCF mixtures. Figure S8p–u’ shows reconstructions of CFN/IBP mixtures requiring three basis functions, highlighting the extraction of ψ4 and ψ5 as spectral changes caused by the intermolecular interaction between CFN and IBP; this did not depend on the linear combinations of intact spectra of pure CFN and IBP.

**Figure 8:**
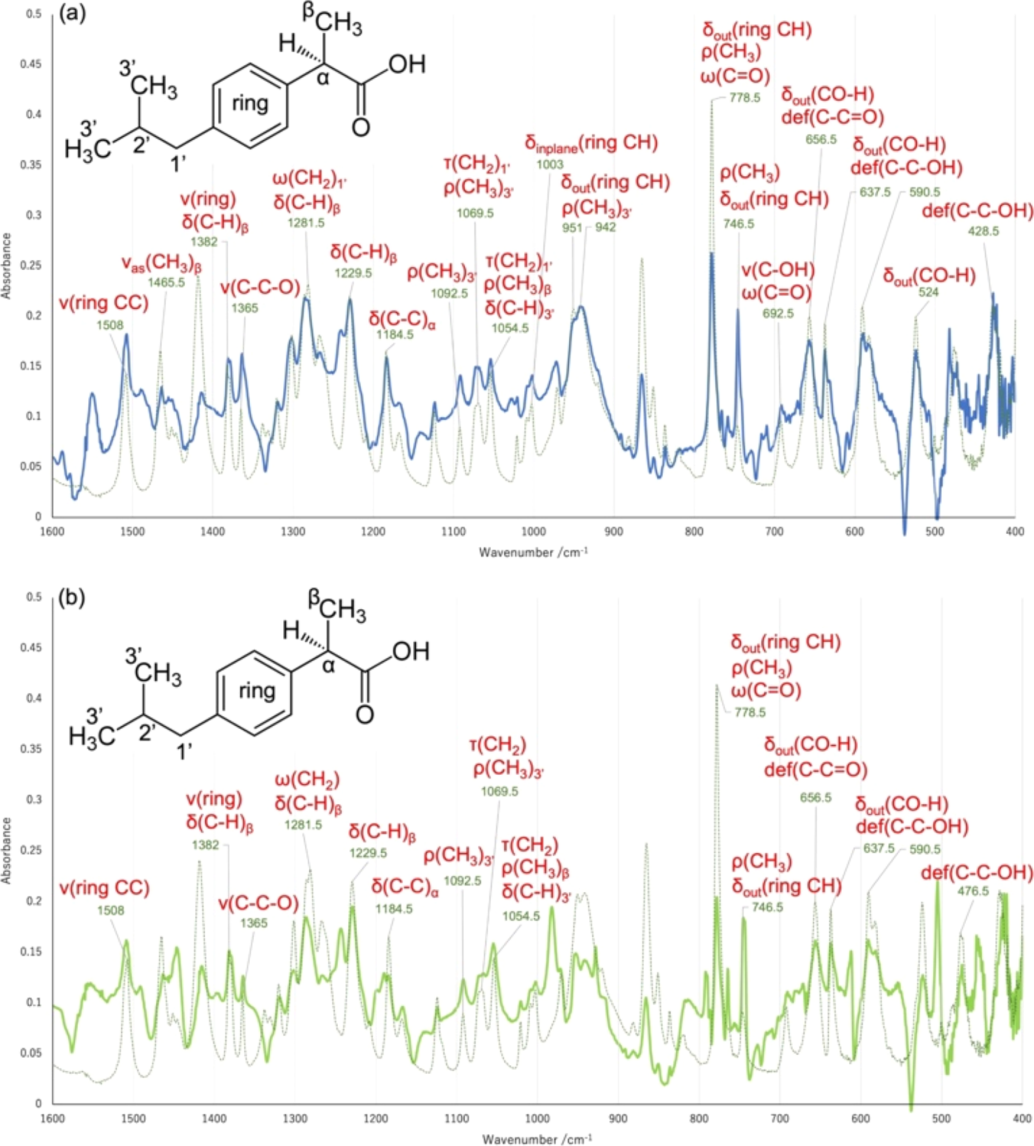
ATR-FTIR spectra were extracted as specific signals in the IBP/CFN (a) and IBP/TPH (b) mixtures. Vibration modes were represented as ν: stretching, δ: bending, ω: wagging, ρ: rocking, and τ: twisting.

Figure 8a shows the basis function, ψ5, with assignments of FTIR signals corresponding to vibration modes of moieties that were presumed to be altered by the addition of a specified mole fraction (0.5 or less) of CFN. Because the signals in the fingerprint region were considered unreliable, the signals were primarily located in the phenylpropionate moiety, excluding the COOH group.^70−72^

For the CFN/DCF mixtures, the spectral signal extraction using SVD computations was inefficient. ψ2 combined the spectra of CFN for the positive half and DCF for the negative half, whereas ψ4 represented spectra of pure DCF and CFN/DCF mixtures. Component λ4 periodically closed to λ2, indicating two components were involved in reconstructing trajectories of DCF mixtures toward pure CFN. This suggests that the decomposed basis functions do not simply specify the spectral composition of the CFN/DCF equimolar mixture. As shown in Figure 4b, an intermediate complex with an exothermal signal within 40 of the DCF *T*_m_ was found in the DCF-rich mixtures. The thermal heterogeneity in the crystalline habits of pure DCF and the CFN/DCF mixture suggested that the state of DCF and its intermolecular interactions were not uniform and switched depending on the CFN-to-DCF molar ratio. Conventional SVD analysis was considered unsuitable for unexpected and discontinuous transitions that lack regularity, thus eliminating noise factors.

The signal at 1452 cm^–1^, which was assigned to the deformation vibration of 2,6-dichloroaniline moiety, increased in the CFN/DCF equimolar mixture (Figure S8s’). This suggests that the intermolecular interactions between DCF and CFN were located in the 2,6-dichloroaniline moiety, with small perturbations of the DCF spectral patterns absorbed within the first to third compositions of SVD computation.

Figure S9 shows the spectra of the TPH/IBP and TPH/DCF mixtures that were similarly treated. The six highest singular values, with a cumulative sum of variance of 97.7%, were separated from the lower plots. The spectra of the pure IBP and TPH/IBP mixture were similar to the basis functions of ψ4 and ψ6. The similarity of the basis function ψ5 to the spectra of pure DCF and TPH/DCF mixture was lower than those of ψ4 and ψ6.

Changes in components of λ2 and λ3 were antiparallel to mole fractions of TPH in the TPH/DCF mixtures and parallel to those in TPH/IBP mixtures. λ6 was enhanced in the TPH/IBP mixture with high IBP content. Figure S9r shows the (λ2/λ1)–(λ3/λ1) diagram, where the trajectory of the TPH/DCF mixtures aligned along the (λ2/λ1)-axis. The trajectory of the TPH/IBP mixtures initially followed the trajectory of the TPH/DCF mixtures; however, this subsequently changed toward a perpendicular (λ3/λ1)-axis direction.

Figure 8b shows the basis function, ψ5, with assignments of FTIR signals that correspond to vibrational modes of moieties. Similar to the CFN/IBP mixtures, the signals were primarily located in the phenylpropionate moiety, excluding the COOH group. For TPH/DCF mixtures, component λ5 showed no correlation to the mole fraction of TPH (Figure S9q). The corresponding basis function, ψ5, resembled a collection of discontinuous peaks, excluding its fingerprint region in the 1000–400 cm^–1^ range.

For the TBR/IBP or TBR/DCF mixtures, basis functions ψ1, ψ2, and ψ3 traced measured spectra of pure TBR, DCF, and IBP (Figure S10d,g,i). However, ψ5 did not consist of the spectra of pure TBR, IBP, DCF, and their mixtures; ψ4 also exhibited limited similarity. The λ4 components in the singular vectors were correlated to λ3 (Figure S10p); this likely represented ψ3 and not the TBR/IBP mixtures. In Figure S10q, λ4 represented the TBR/DCF mixtures; however, ψ4 did not correspond to the spectrum of the TBR/DCF equimolar mixture. These results indicate that the spectral changes only corresponded to the proportions of TBR to IBP and TBR to DCF. This was consistent with the following finding from the previous section: no intermolecular interactions in TBR/IBP or TBR/DCF were identified. Figure S10r,s showed trajectories of components λ2/λ1 and λ3/λ1 throughout the tracing of the smooth transformation. The linear combinations of the first, second, and third basis functions representing the spectra of the TBR/IBP and TBR/DCF mixtures are shown in Figure S10t–w.

Similar conclusions were drawn for the aforementioned TBR, XAT/IBP, and XAT/DCF mixtures (Figure S11).

### 3.8 NMR Study for the TPH/DCF Mixtures

A few components were correlated with the mole fraction of TPH in the TPH/DCF mixtures. Figures 4d and 5h show the TPH/DCF mixtures that produce twin endothermic signals, suggesting that their intermolecular interactions are not continuous and may vary in equimolar or TPH-rich mixtures. Isolating the interaction signals from the FTIR spectra was not possible. However, the signal at 1452 cm^–1^ intensified in the TPH/DCF mixture with a molar ratio of 1:3 (Figure S9u). This suggests that the intermolecular interactions of DCF with CFN or TPH were not easily identifiable in the FTIR spectra, despite the differences between the DSC curves of pure DCF and the TPH/DCF mixtures (Figure 4b,d).

**Figure 9:**
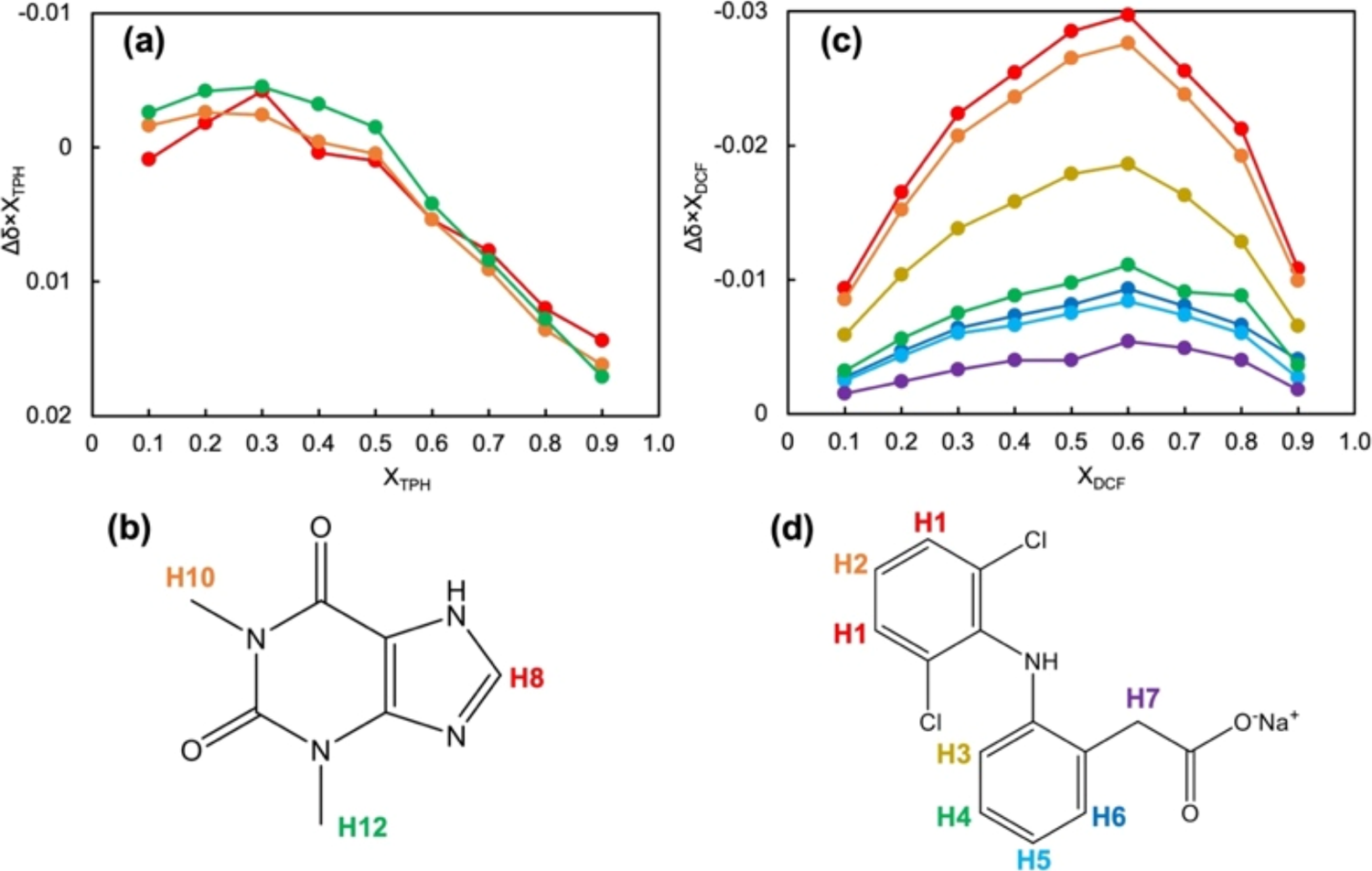
Job’s plot obtained by ^1^H NMR measurements of TPH and DCF sodium salt in various mole fractions. (a) Job’s plot of TPH. (b) Numbering of each hydrogen in the TPH. (c) Job’s plot of DCF sodium salt. (d) Numbering of each hydrogen in the DCF sodium salt. The plot colors in (a) and (c) correspond to the numbering colors in (c) and (d). Refer to Figures S12 and S13 for the signal assignments and the chemical shifts.

To confirm the intermolecular interactions between TPH and DCF, 400 MHz ^1^H NMR spectra of the TPH/DCF mixtures were analyzed in D_2_O. Improving the measurement accuracy required samples with high concentrations, which enhanced the solubility of DCF sodium salt and TPH. Regarding the THP/DCF mixtures, the chemical shift values of the TPH signals decreased(Figure S12), whereas those of the DCF signals increased depending on the mole fraction of the DCF sodium salt (Figure S13). Because the control chemical shifts changed with the concentration of the molecules, the changes were adjusted for differences between the chemical shifts and the control chemical shifts. Figure 9 shows Job’s plots depicting the difference in the chemical shift and the mole fraction as a function of the mole fractions of TPH and DCF sodium salt. These plots indicate the stoichiometric intensity of the intermolecular interaction between TPH and the DCF sodium salt, resulting in an optimal molar ratio of 1:2 in the TPH/DCF complex. The most significant chemical shift variations in the DCF sodium salt were observed in the hydrogen atoms of the 2,6-dichloroaniline moiety, indicating that this is the site for the intermolecular interaction of DCF with TPH. Figure S14 shows the DOSY spectrum, verifying the formation of the TPH/DCF complex in D_2_O with the following diffusion coefficient: *D*_DOSY_ = 0.485 m^2^ s^–1^.^51^ These results corroborate the speculation derived from the SVD computation of the FTIR spectra of TPH/DCF mixtures in the crystals. They confirmed that TPH approaches the 2,6-dichloroaniline moiety of DCF in solution and acts as a hydrotrope.

## 4 Conclusions

Assessing the solubility and interactions of APIs, IBP, and DCF in the presence of various XAT derivatives showed that CFN and TPH enhanced the solubility of DCF but had no impact on IBP solubility. DCF and IBP acted as solubilizers; however, DCF had a greater effect. When equimolar mixtures of IBP or DCF with CFN, TPH, TBR, or XAT were introduced into a phosphate buffer solution, CFN and TPH decreased IBP solubility and increased DCF solubility. DSC thermal analysis indicated shifts in the peak temperatures, suggesting interactions between the APIs and XAT derivatives. Changes in the diffraction patterns were observed using XRPD analysis, indicating alterations in the crystalline structures. Analysis of the ATR-FTIR spectra of the mixtures showed no qualitative changes compared with those of the pure XAT derivatives.

SVD was used to analyze the FTIR spectra of the mixtures of XAT derivatives with IBP or DCF. Excluding the COOH stretching and vibration modes, significant changes in the singular vector components depended on the mole fraction of CFN in the CFN/IBP mixtures, particularly in the phenylpropionic acid moieties. Spectral signal extraction using SVD computations for the TPH/IBP and TPH/DCF mixtures yielded similar results for the IBP spectra. However, for the CFN/DCF and TPH/DCF mixtures, the SVD analysis proved inefficient owing to discontinuous transitions. NMR analysis of the TPH/DCF mixtures indicated intermolecular interactions between the 2,6-dichloroaniline moiety of DCF and TPH. SVD analyses of the FTIR spectra conducted for the TBR/IBP and TBR/DCF mixtures indicated the absence of intermolecular interactions.

Although IBP and DCF were shown to form intermolecular interactions with CFN and TPH, these effects caused IBP solubility to decrease and DCF solubility to increase.

## Supporting Information Available

Curves of the IBP and DCF mixtures with CFN, TPH, TBR, and XAT (Figure S1); DSC-XRPD diffractograms of DCF polymorphs, TPH, TPH/DCF (Figure S2); ATR-FTIR spectra of CFN, TPH, TBR, and XAT (Figure S3); ATR-FTIR spectra of IBP and DCF, and assignment of the FTIR signal onto simulated peaks (Figure S4); ATR-FTIR spectra of IBP and DCF mixtures with CFN, TPH, TBR, and XAT at various molar ratios (Figure S5); the results of SVD computation for the IBP mixtures with XAT derivatives (Figure S6); the results of SVD computation for the DCF mixtures with XAT derivatives (Figure S7); the trajectories of the first to fifth singular vectors of the mixtures for CFN/IBP and CFN/DCF (Figure S8); the trajectories of the first to fifth singular vectors of the mixtures for TPH/IBP and TPH/DCF (Figure S9); the trajectories of the first to fifth singular vectors of the mixtures for TBR/IBP and TBR/DCF (Figure S10); the trajectories of the first to fifth singular vectors of the mixtures for XAT/IBP and XAT/DCF (Figure S11); signal assignment for TPH on ^1^H NMR spectrum, and the chemical shift of TPH in TPH/DCF mixtures (Figure S12); signal assignment for DCF on ^1^H NMR spectrum and the chemical shift of DCF in TPH/DCF mixtures (Figure S13); DOSY spectrum of DCF in THP/DCF solution (Figure S14); the SEM images of IBP, DCF, CFN, TPH, and mixtures (Figure S15) (PDF)
neat_compounds_FTIR (XLSX)
Caffeine_FTIR (XLSX)
Theophyline_FTIR (XLSX)
Theobromine_FTIR (XLSX)
Xanthine_FTIR (XLSX)

## Abbreviations

CFN: caffeine
TPH: theophylline
TBR: theobromine
XAT: xanthine
CYP1A2: cytochrome P450 type 1A2
API: active pharmaceutical ingredient
LA: local anesthetics
SSRI: serotonin-selective receptor inhibitor
*T*_m_: melting points
NSAID: nonsteroidal anti-inflammatory drug
DCF: diclofenac
IBP: (*S*)-(+)-ibuprofen
LDC: lidocaine
FAM: famotidine
CIM: cimetidine
XRPD: X-ray powder diffraction
DSC: differential scanning calorimetry
ATR-FTIR: attenuated total reflection-Fourier transform infrared, *P*, *P*_oct_
*K*_OW_: the intrinsic partition coefficient between the 1-octanol and aqueous phases
*D*: the apparent partition coefficient, namely distribution coefficient
LD_50_: 50% lethal dose
*V*_d_: distribution volume
