# Supplementary material for: How Does the Powder Mixture of Ibuprofen and Caffeine Attenuate the Solubility of Ibuprofen? Comparative Study for the Xanthine Derivatives to Recognize Their Intermolecular Interactions Using Fourier-Transform Infrared (FTIR) Spectra, Differential Scanning Calorimetry (DSC), and X-ray Powder Diffractometry (XRPD)
Source: Mol Pharm. 2024 Aug 7;21(9):4524–40. doi: 10.1021/acs.molpharmaceut.4c00429 (PMC11487632; doi:10.1021/acs.molpharmaceut.4c00429)
Supplement: Supplementary file 1 — mp4c00429_si_001.pdf [file mp4c00429_si_001.pdf]

How does the powder mixture of ibuprofen and caffeine attenuate the solubility of ibuprofen? Comparative study for the xanthine derivatives to recognize their intermolecular interactions using Fourier-transform infrared (FTIR) spectra, differential scanning calorimetry (DSC), and X-ray powder diffractometry (XRPD).

Shoya Suenaga¶, Hikaru Kataoka¶, Kanji Hasegawa, Ryotaro Koga,
Chihiro Tsunoda, Wataru Kuwashima, Tomohiro Tsuchida, and Satoru Goto*

Supplemental Materials

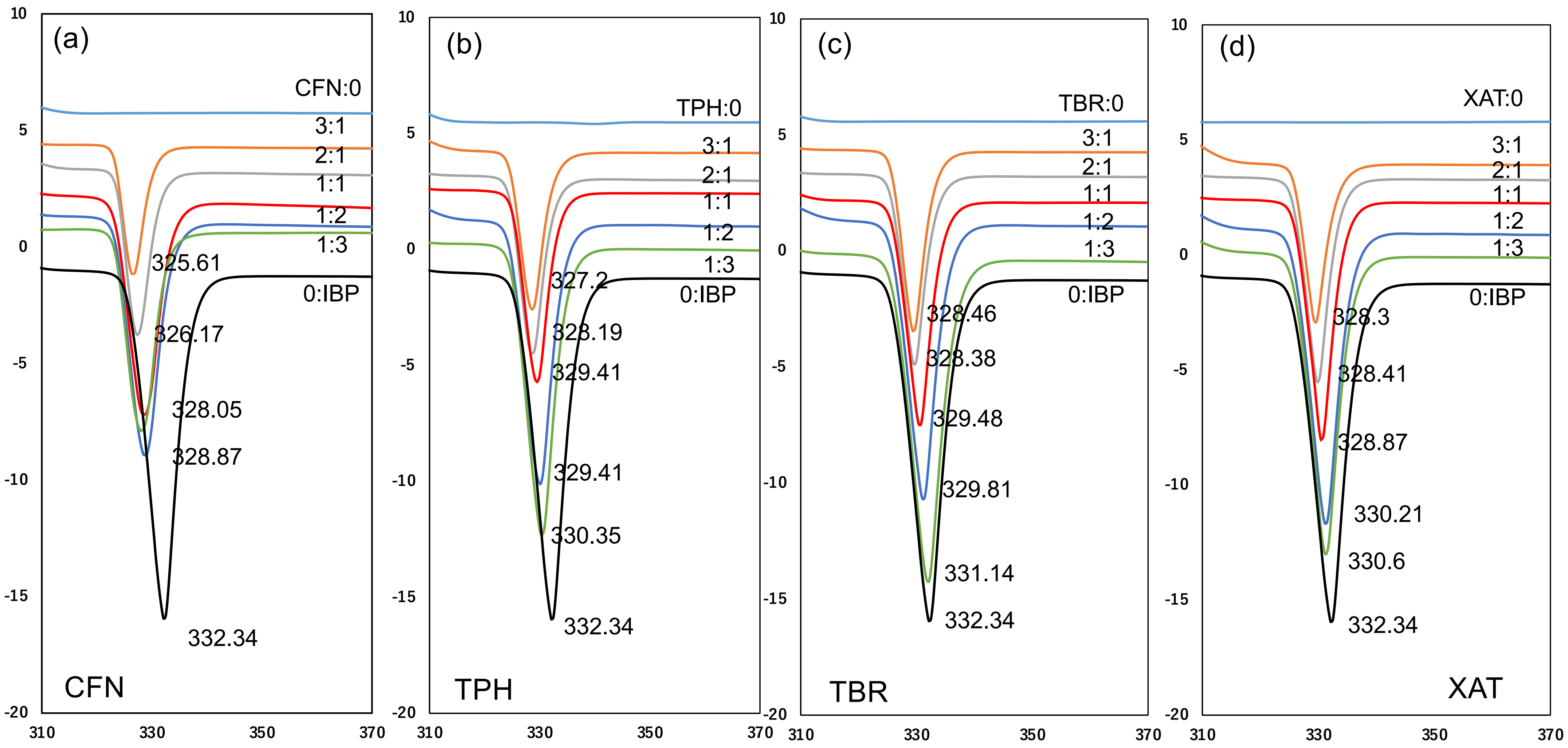

Figure S1. The endothermic signal of IBP melting in the DSC thermograms of the IBP mixtures with CFN (a), TPH (b), TBR (c), and XAT (d), which were expanded from the thermograms in Figure 4. Numbers expressed the temperatures of the peak tops. The intersections of the left-side tangent lines of peaks on the baseline extensions shifted in the mixtures of CFN/IBP and TPH/IBP. However, those in the mixtures of TBR/IBP and XAT/IBP were invariable, indicating no intermolecular interactions.

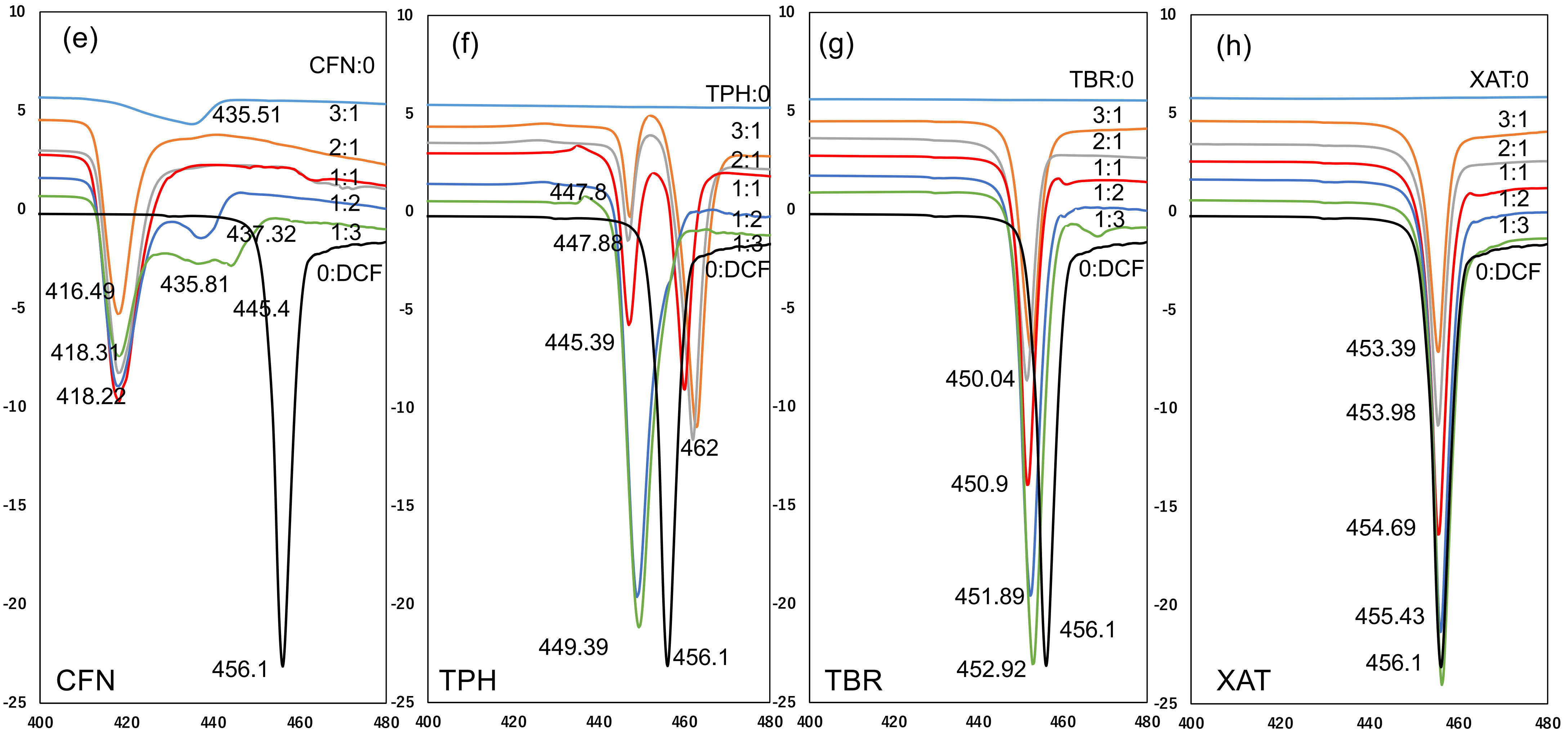

Figure S1. The endothermic signal of DCF melting in the DSC thermograms of the DCF mixtures with CFN (e), TPH (f), TBR (g), and XAT (h), which were expanded from the thermograms in Figure 4. Numbers expressed the temperatures of the peak tops. The intersections of the left-side tangent lines of peaks on the baseline extensions shifted in the mixtures of CFN/DCF, TPH/DCF, and TBR/DCF. However, those in the mixtures of XAT/DCF were invariable, indicating no intermolecular interactions.

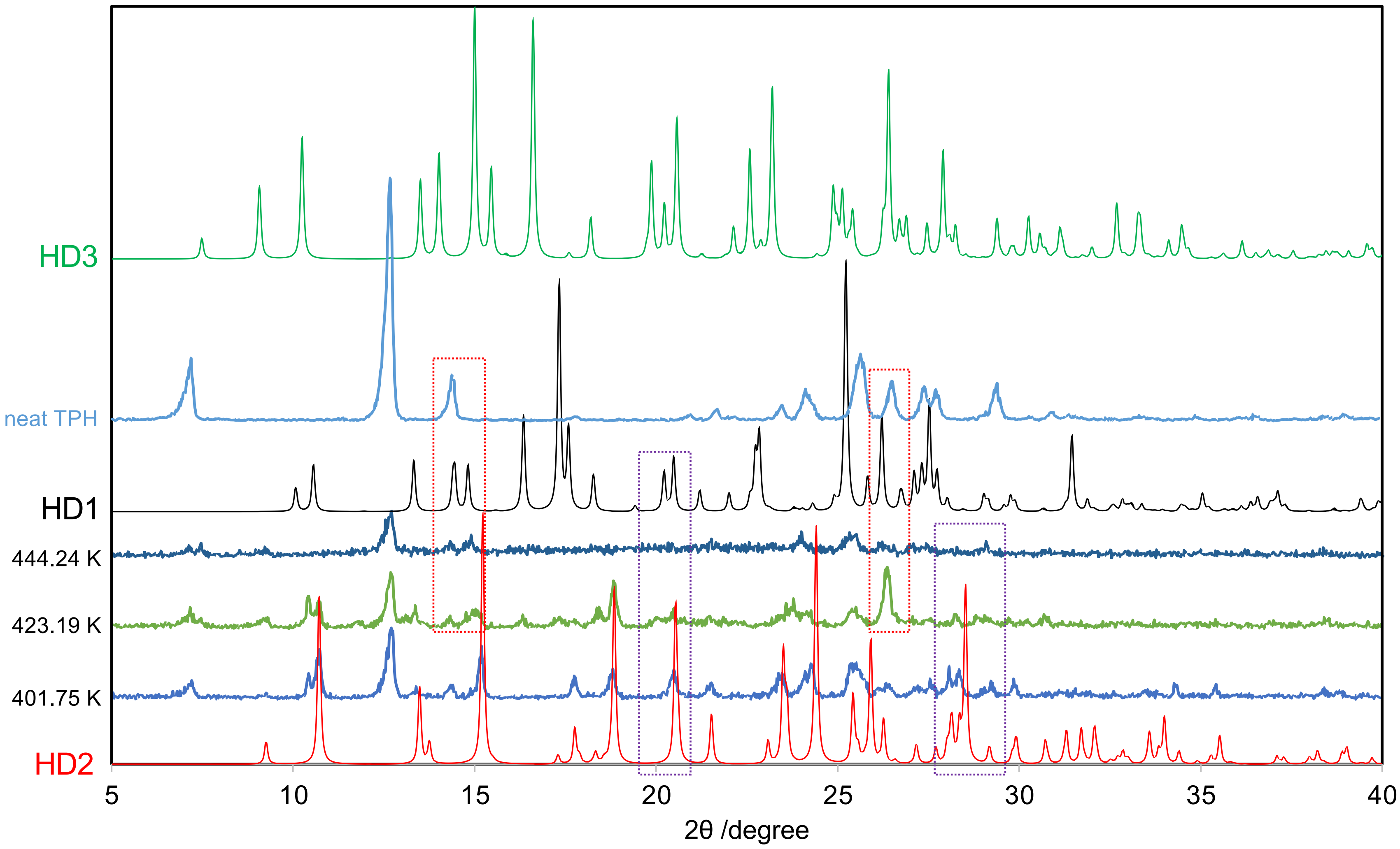

Figure S2. DSC-XRPD diffractograms of the neat TPH (sky blue), the TPH/DCF equimolar mixture at 401.75 K (cyan), 423.19 K (moss green), and 444.24 K (indigo) from Figure 5(h), and the simulated diffractograms of DCF with its polymorphs of HD1 (black), HD2 (red), and HD3 (green).

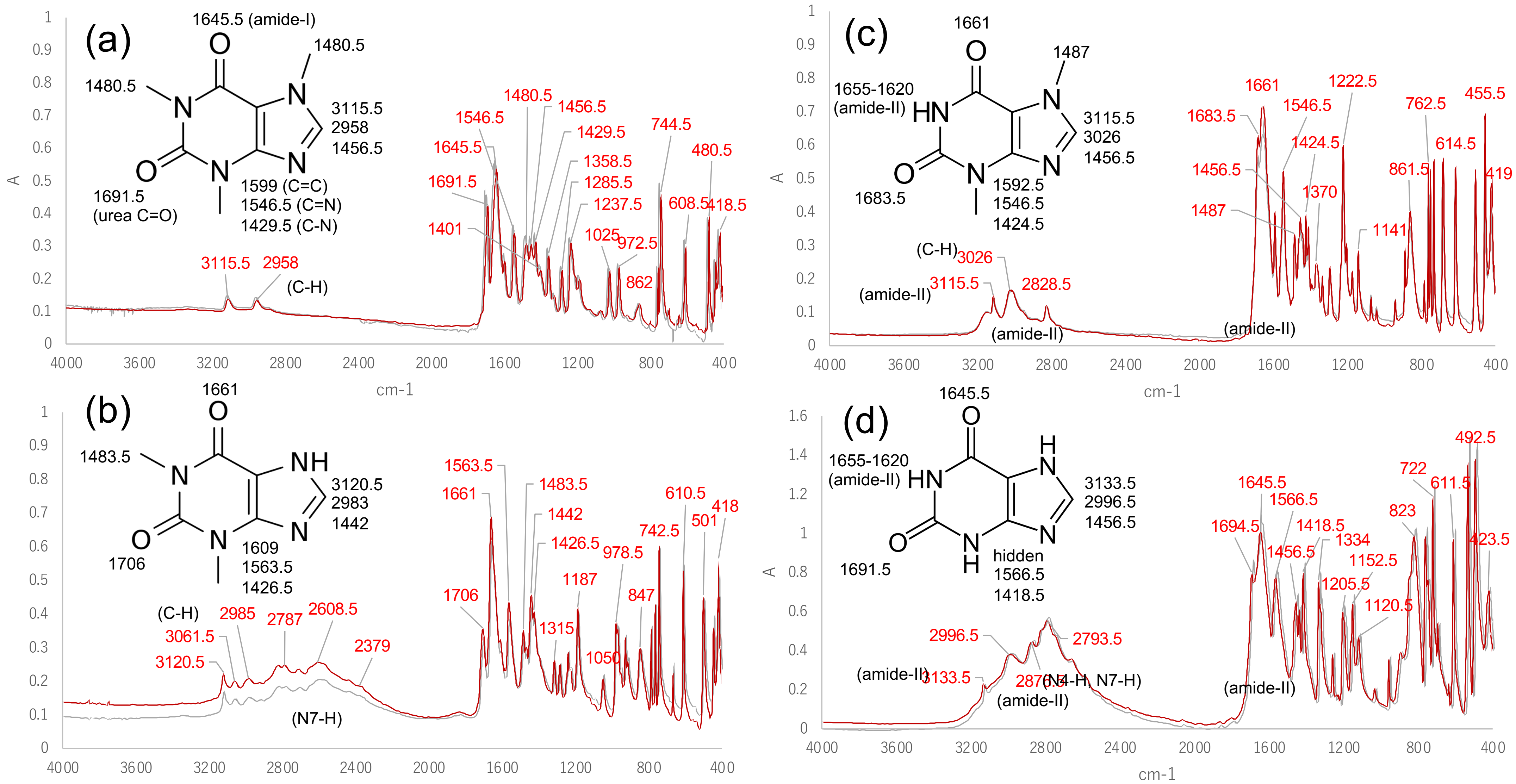

Figure S3. The ATR-FTIR spectra of CFN (a), TPH (b), TBR (c), and XAT (d). Bordeaux lines show spectra of neat crystals, and gray lines show their solids standing for 7 days. Numbers adjacent to noticeable peaks express the corresponding wave numbers. It was considered no particular changes in the significant peaks.

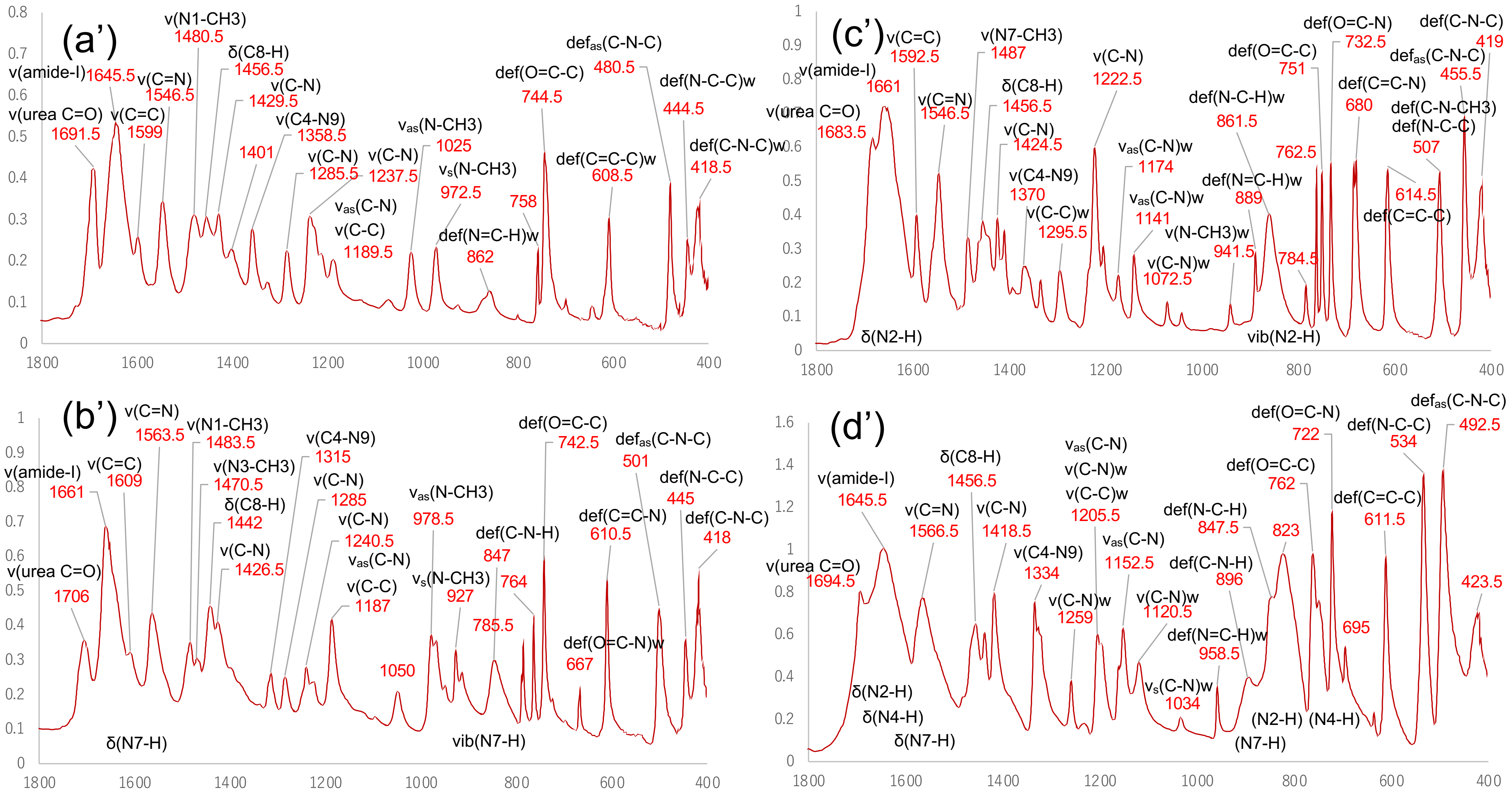

Figure S3. Expansions of the FTIR spectra of CFN (a'), TPH (b'), TBR (c'), and XAT (d') into the wavenumber range of 1800-400 cm^{-1}. Numbers adjacent to noticeable peaks express the corresponding wave numbers. Their assignments to the moieties were appended.

Table S1. The signal assignments for the normal modes of the structural vibrations in the ATR-FTIR spectra of the neat XAT derivatives.

	CFN	TPH	TBR	XAT
Purine C8-H stretching	3112, 2958	3121, 2985	3116, 3026	3134, 2997
Amide-II (N2-H stretching)	-	-	2829	2877
N3-H, N7-H stretching	-	2787, 2609	-	2794, …
Urea C2=O stretching: Falk 1989	1692	1706	1684	1695
Amide-I C6=O stretching: Falk 1989	1646	1661	1661	1646
Amide-II N1-H bending (scissoring)	-	-	1655-1620	1655-1620
Purine C4=C5 stretching: Cohen 1975	1599	1609	1592	n.d.
Purine C8=N9 stretching: Cohen 1975	1547	1564	1547	1567
substitution CH3 bending (1453)	1480	1484	1487	-
Purine C8-H bending: Cohen 1975	1457	1442	1457	1457
Purine C5-N7 stretching: Silverstein	1430	1427	1425	1419
Purine C4-N9 stretching	1359	1315	1370	1334
Purine C-N, C-O vibration: Cohen 1975	1289,1238, 1190	1285,1241, 1187	1295,1223, 1174	1259,1206, 1153

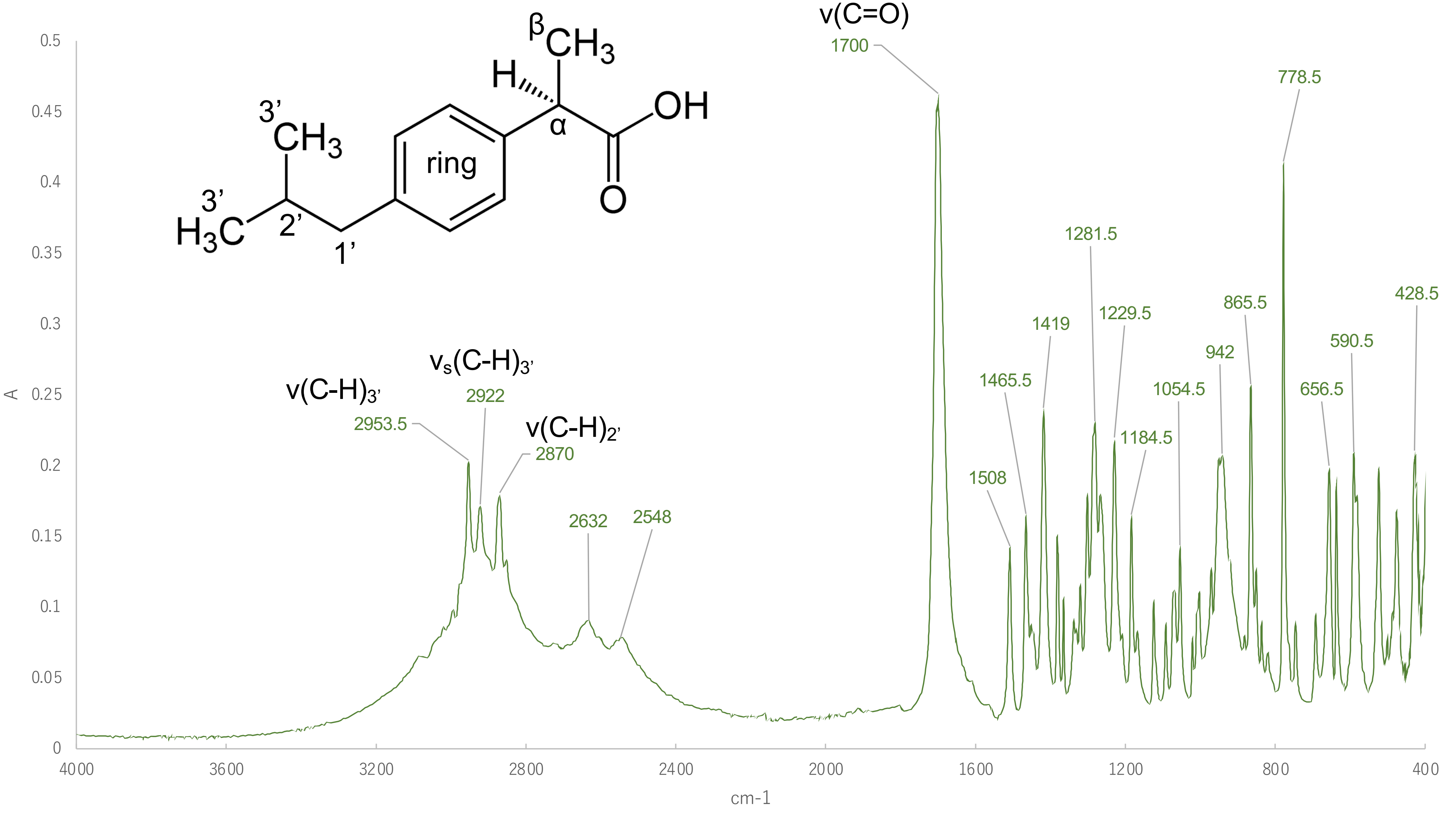

Figure S4. The ATR-FTIR spectra of IBP.

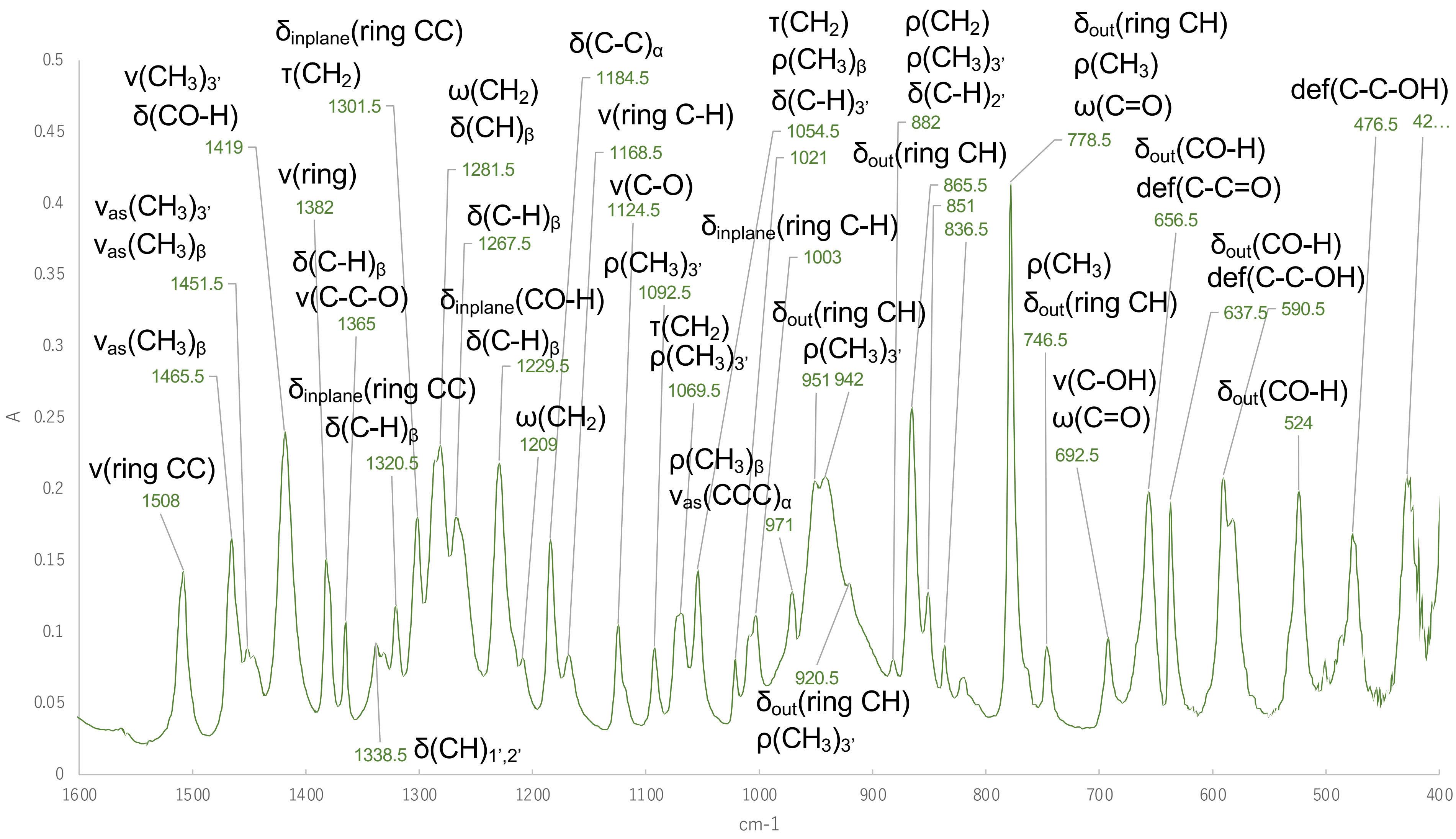

Figure S4. The ATR-FTIR spectra of IBP. Vibration modes were represented as v: stretching, δ: bending, ω: wagging, ρ: rocking, and τ: twisting.

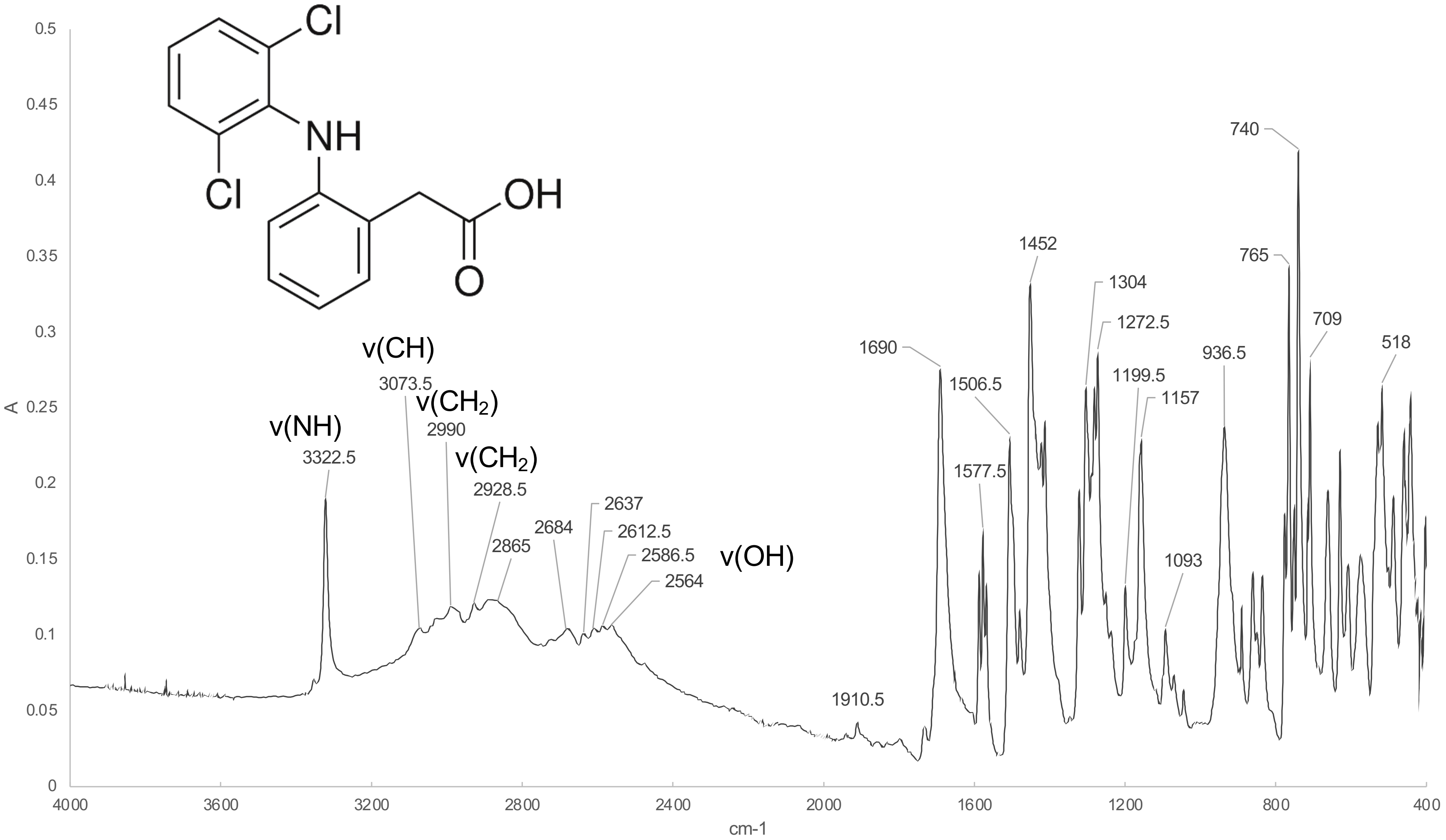

Figure S4. The ATR-FTIR spectra of DCF.

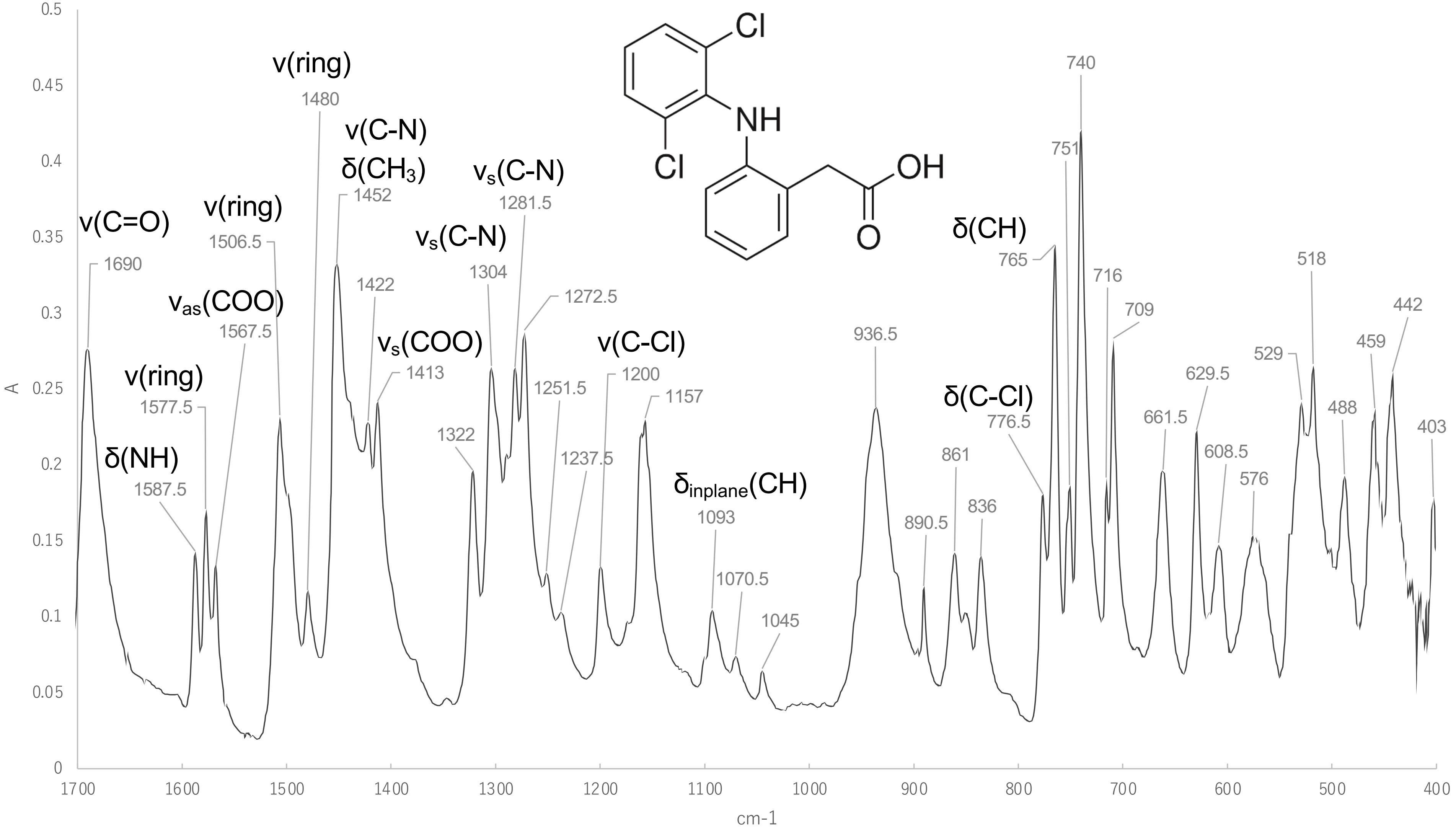

Figure S4. The ATR-FTIR spectra of DCF. Vibration modes were represented as ν: stretching, δ: bending, ω: wagging, ρ: rocking, and τ: twisting.

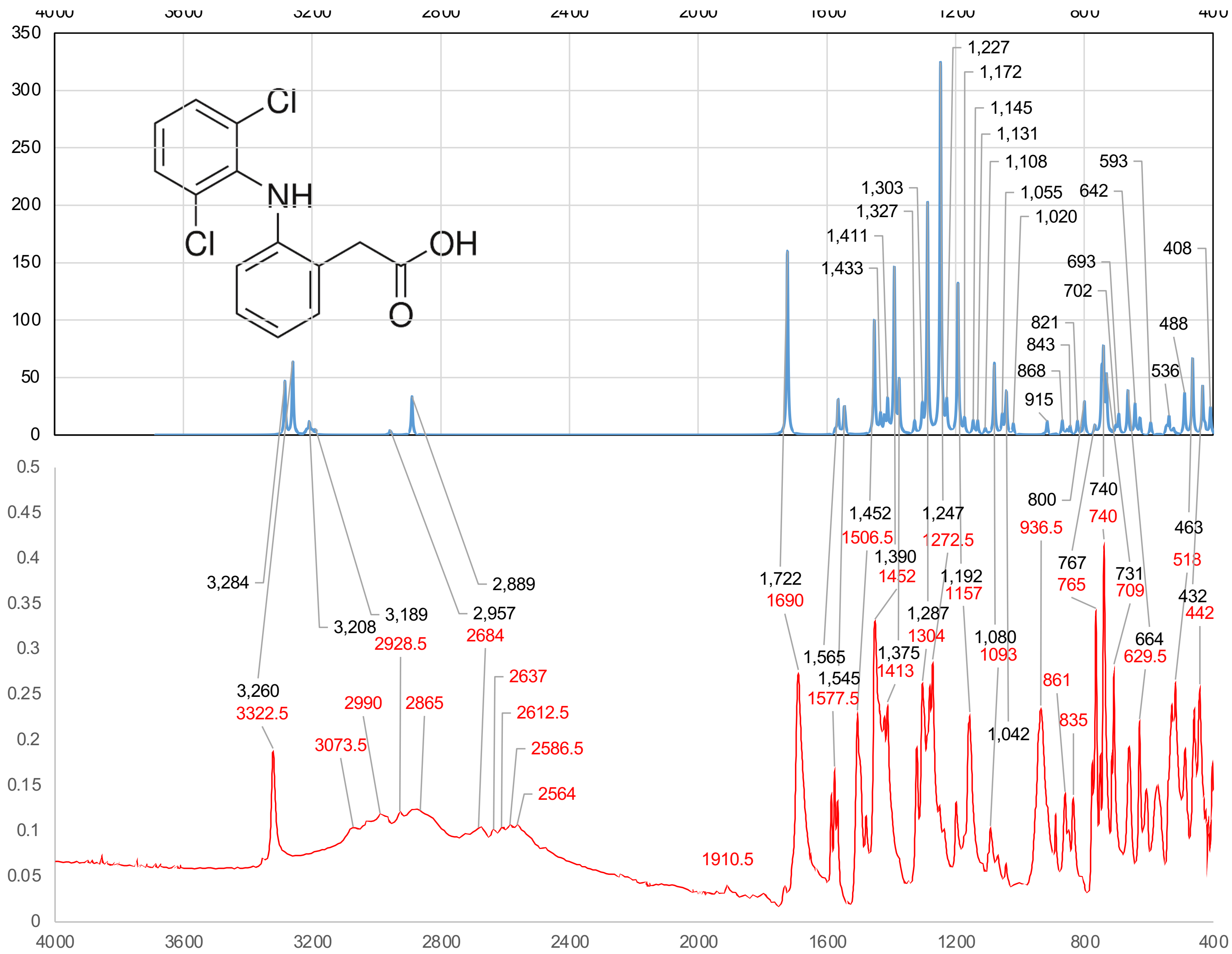

Figure S4. The ATR-FTIR spectra of DCF. Assignment the FTIR signals onto the simulated peaks in the normal mode analysis.

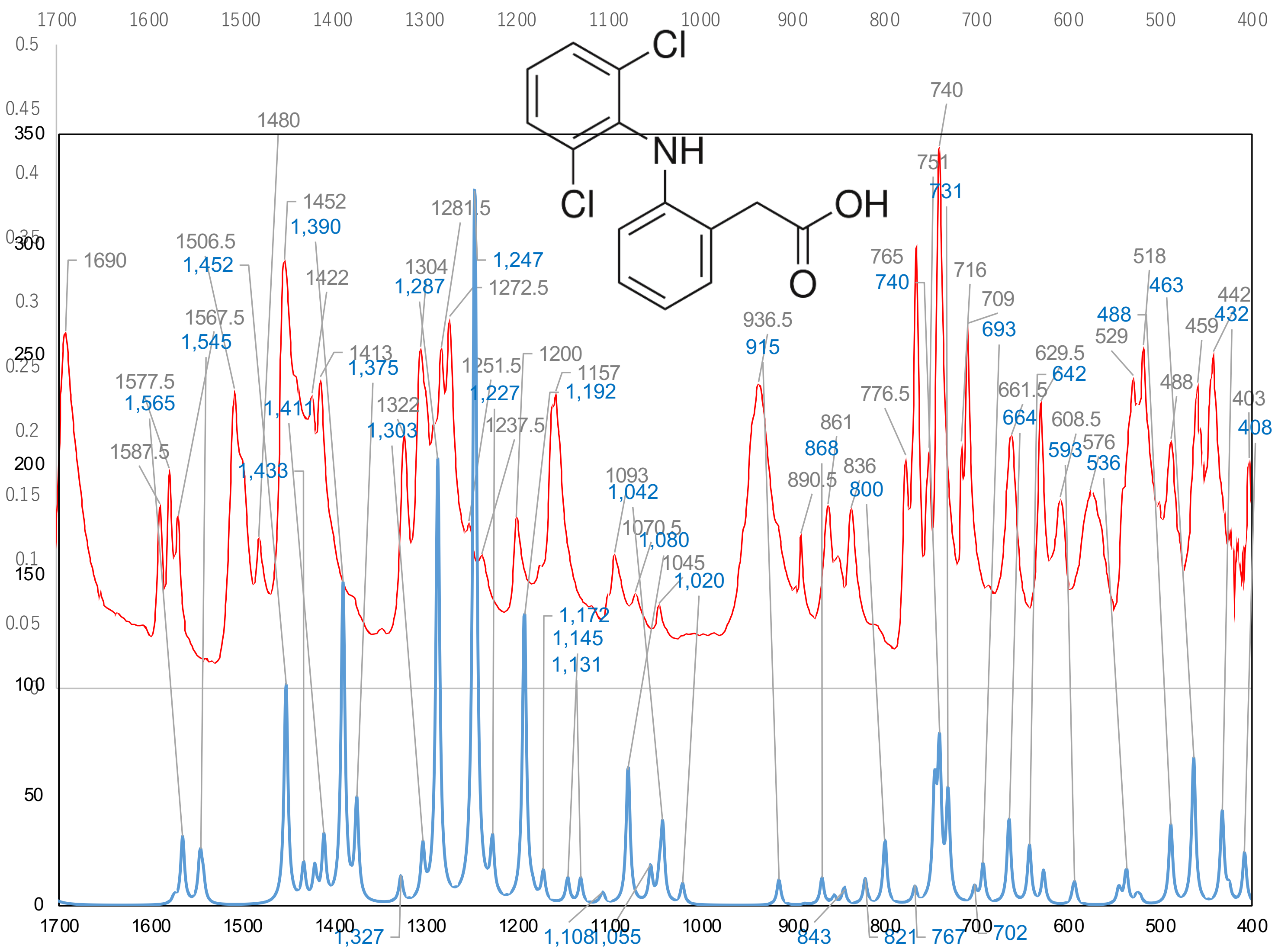

Figure S4. The ATR-FTIR spectra of DCF. Assignment the FTIR signals onto the simulated peaks in the normal mode analysis.

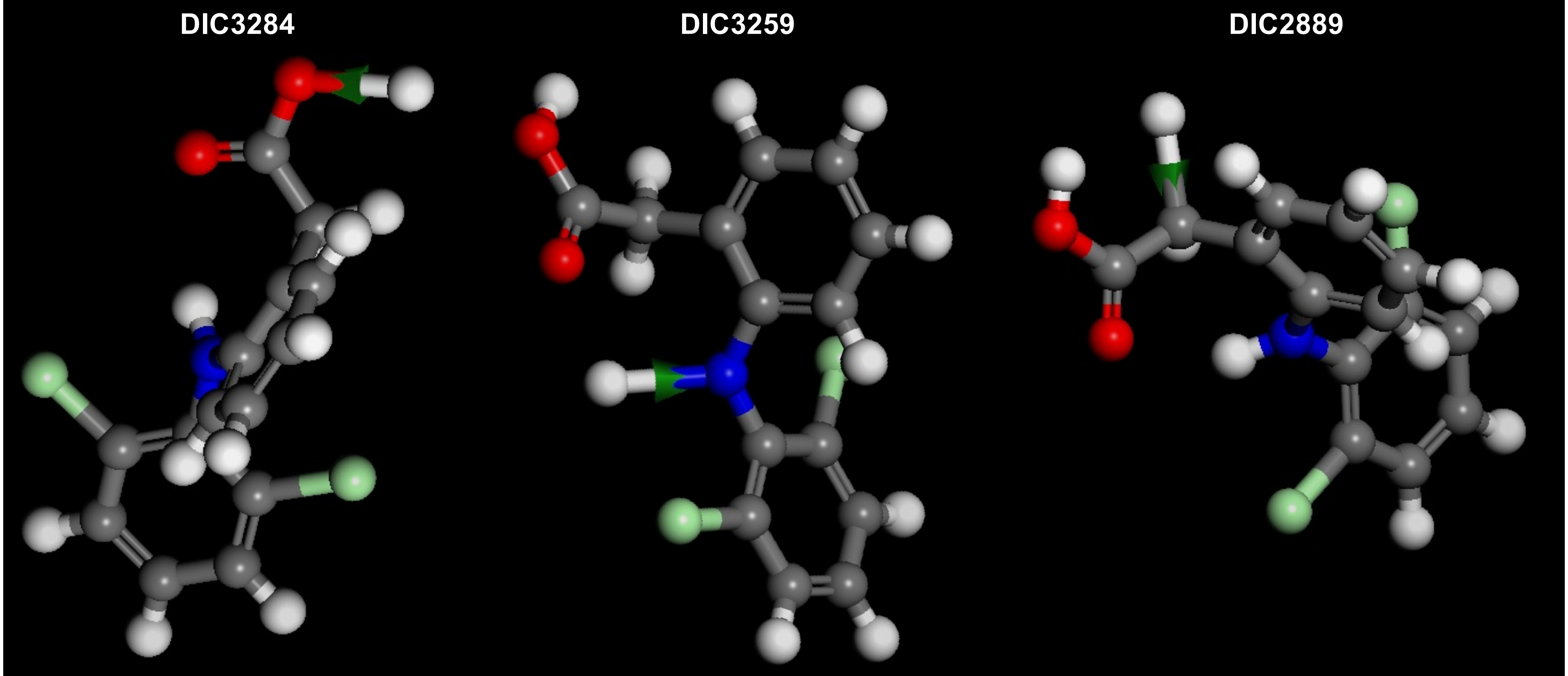

Figure S4. The vibration modes of the assigned peaks at the wavenumbers of 3284, 3259, and 2889 cm^{-1}.

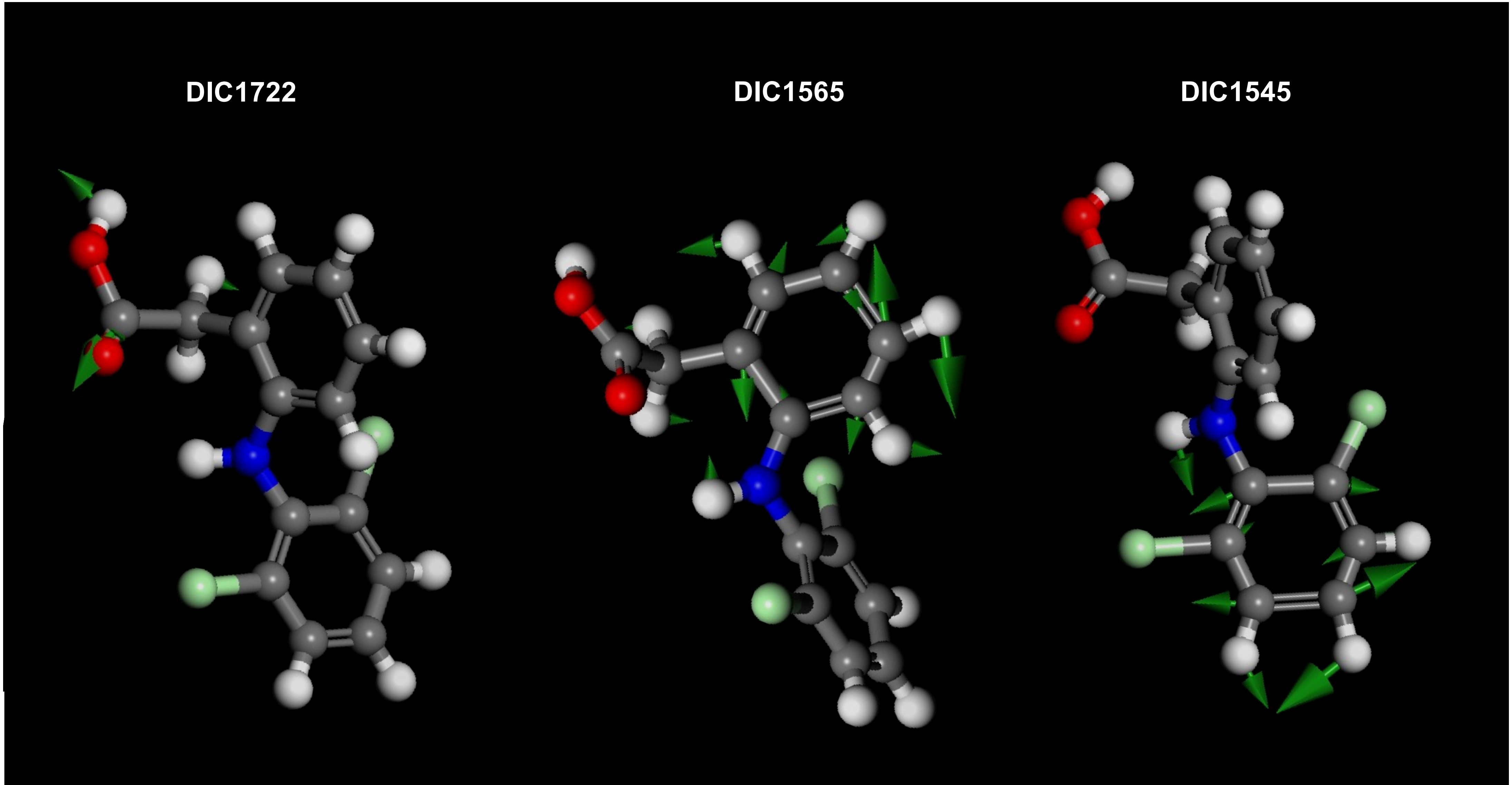

Figure S4. The vibration modes of the assigned peaks at the wavenumbers of 1722, 1565, and 1545 cm^{-1}.

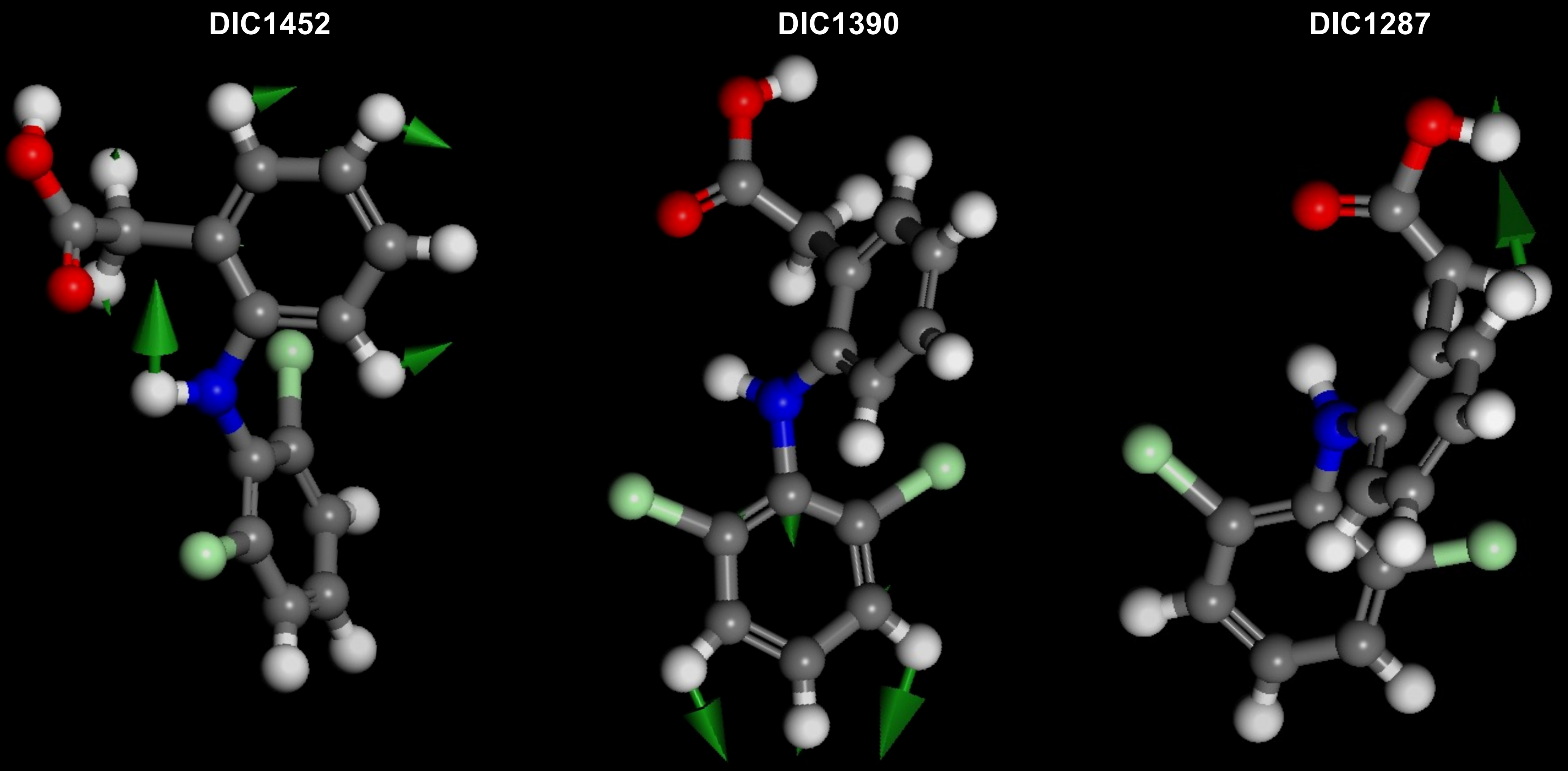

Figure S4. The vibration modes of the assigned peaks at the wavenumbers of 1452, 1390, and 1287 cm^{-1}.

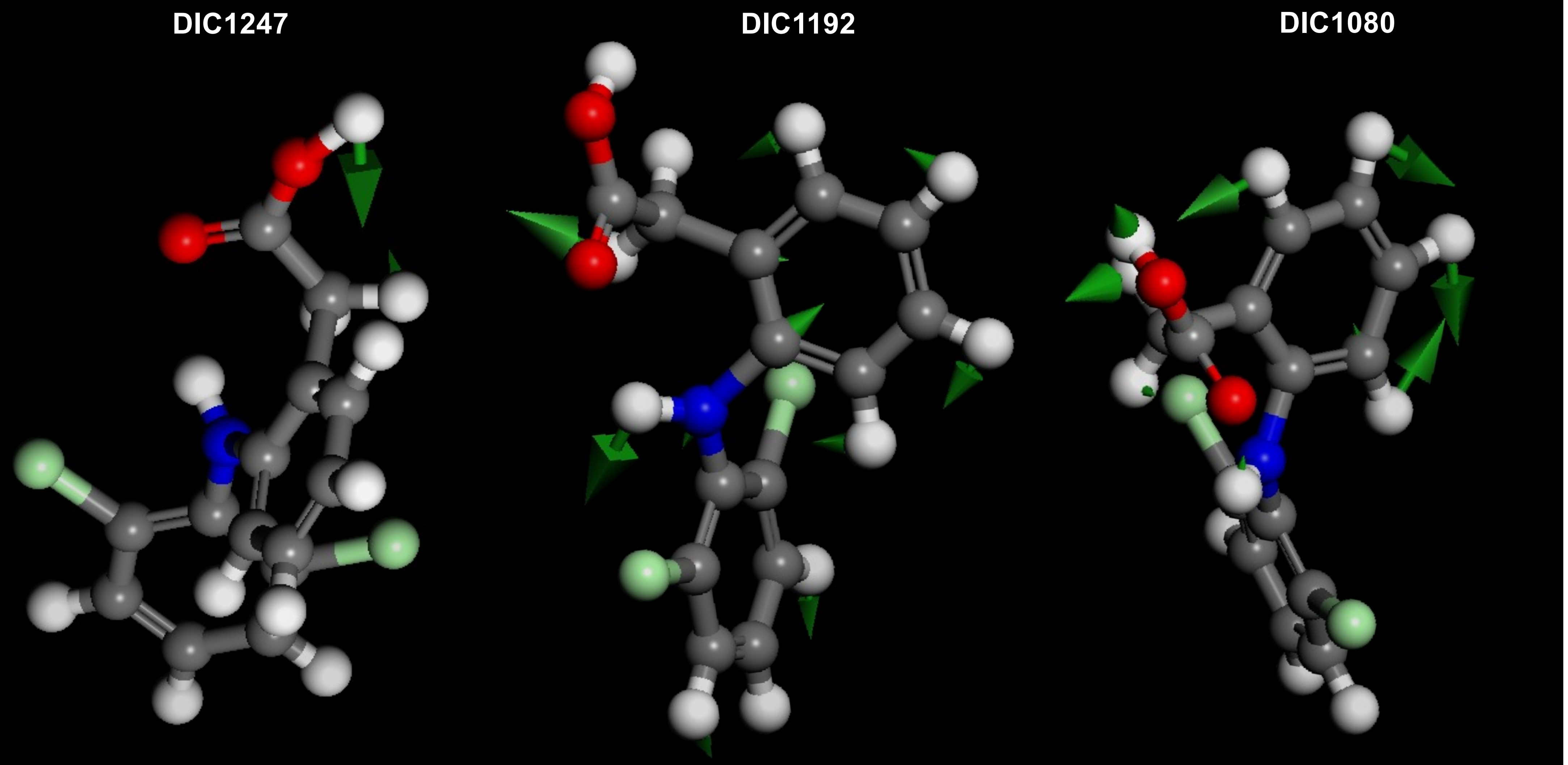

Figure S4. The vibration modes of the assigned peaks at the wavenumbers of 1247, 1192, and 1080 cm^{-1}.

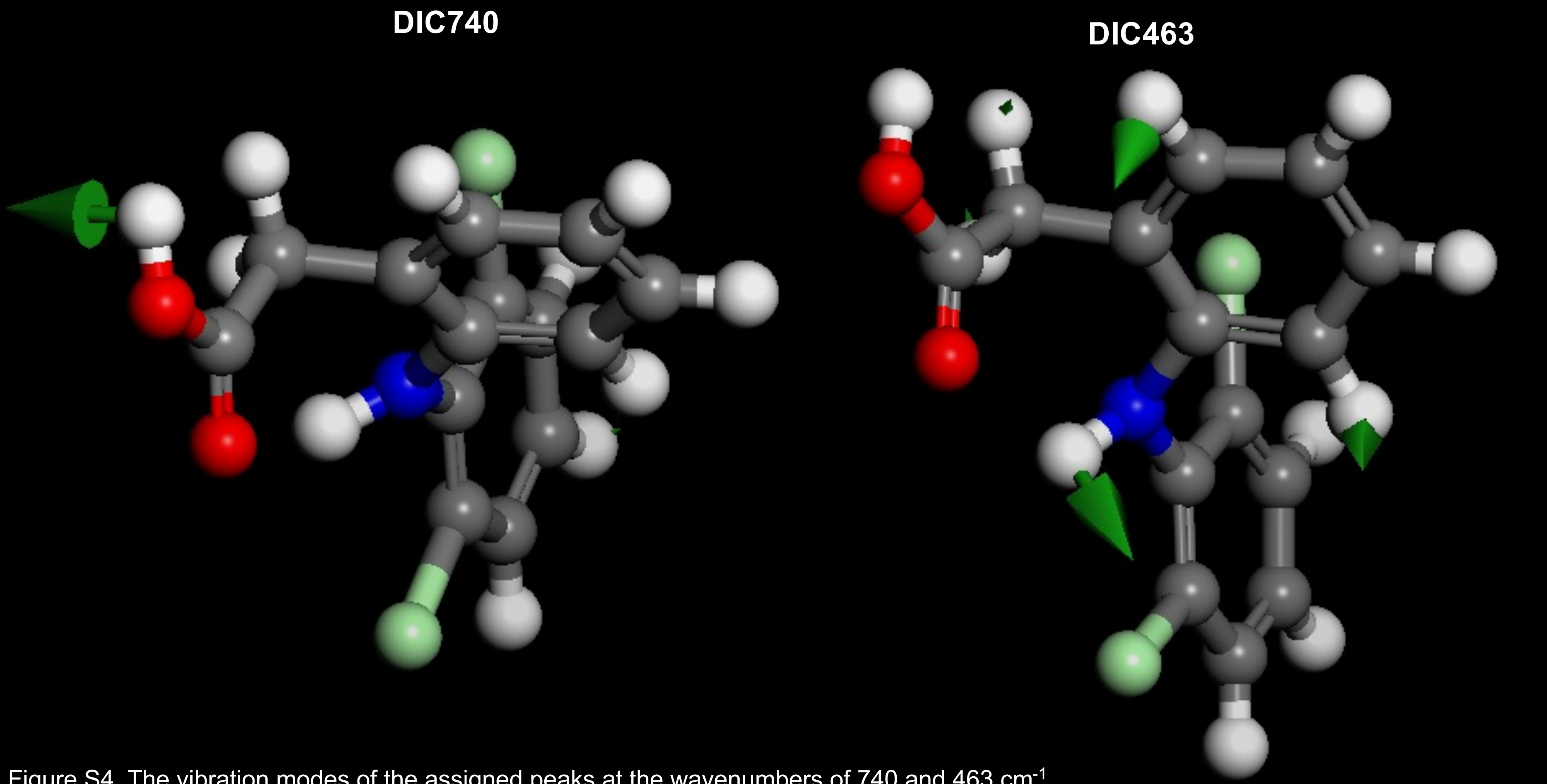

Figure S4. The vibration modes of the assigned peaks at the wavenumbers of 740 and 463 cm^{-1}.

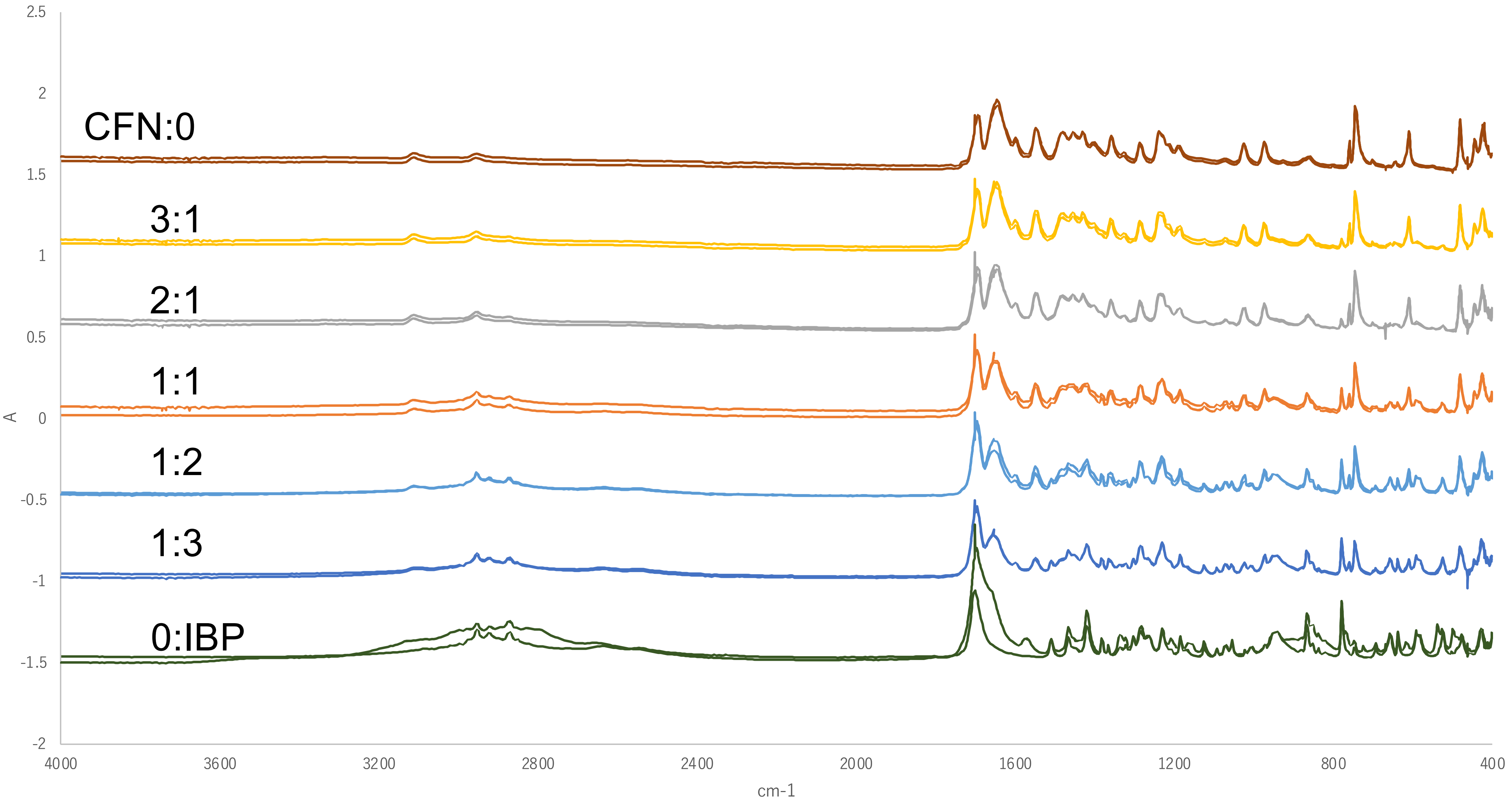

Figure S5. (a) Crystalline FTIR spectra of mixtures of IBP with CFN at various molar ratios. Neat CFN (brown), neat IBP (black), and their mixtures at the ratios of 3:1 (yellow), 2:1 (gray), 1:1 (orange), 1:2 (sky blue), and 1:3 (blue) were measured twice.

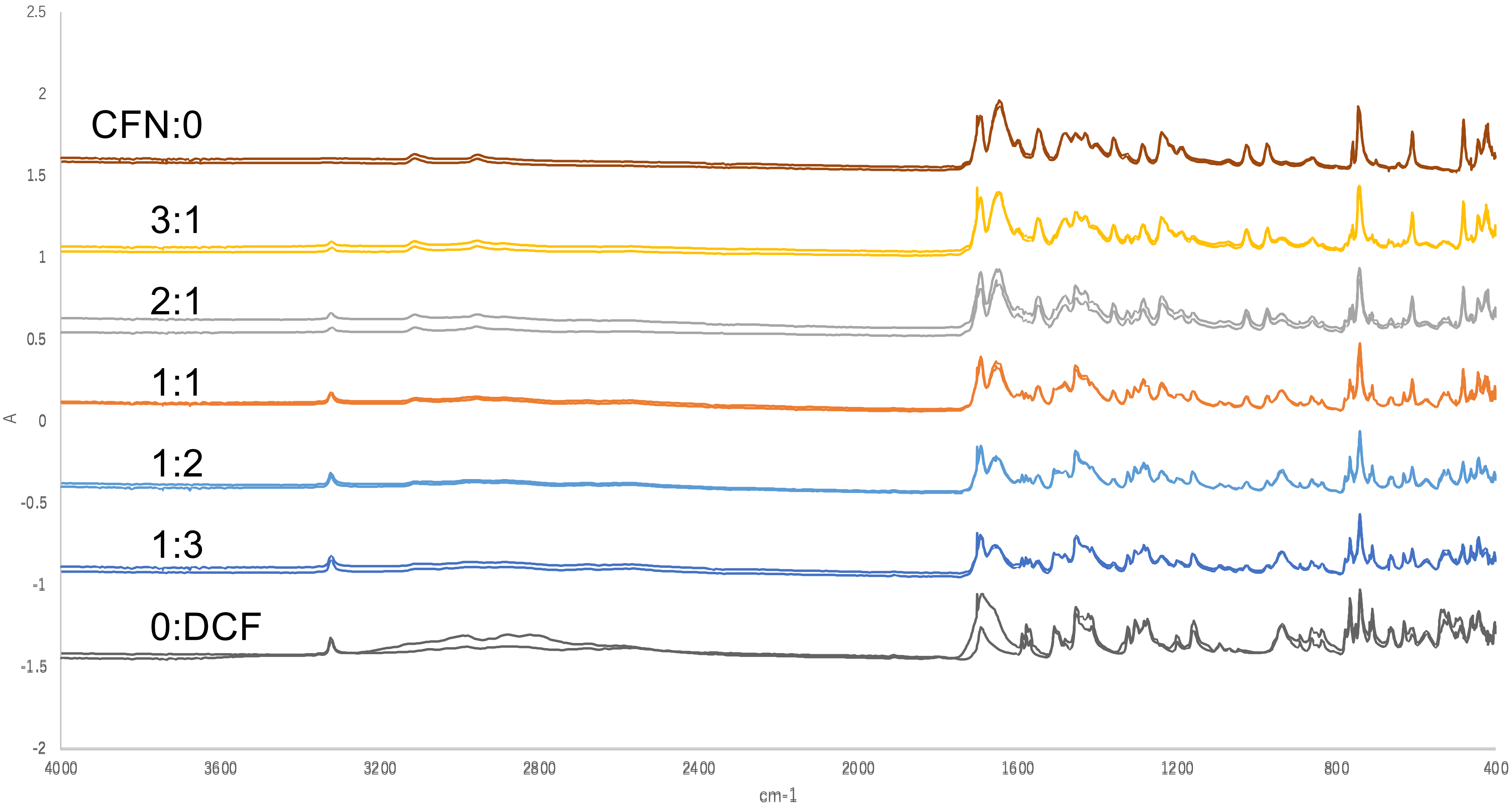

Figure S5. (b) Crystalline FTIR spectra of mixtures of DCF with CFN at various molar ratios. Neat CFN (brown), neat DCF (black), and their mixtures at the ratios of 3:1 (yellow), 2:1 (gray), 1:1 (orange), 1:2 (sky blue), and 1:3 (blue) were measured twice.

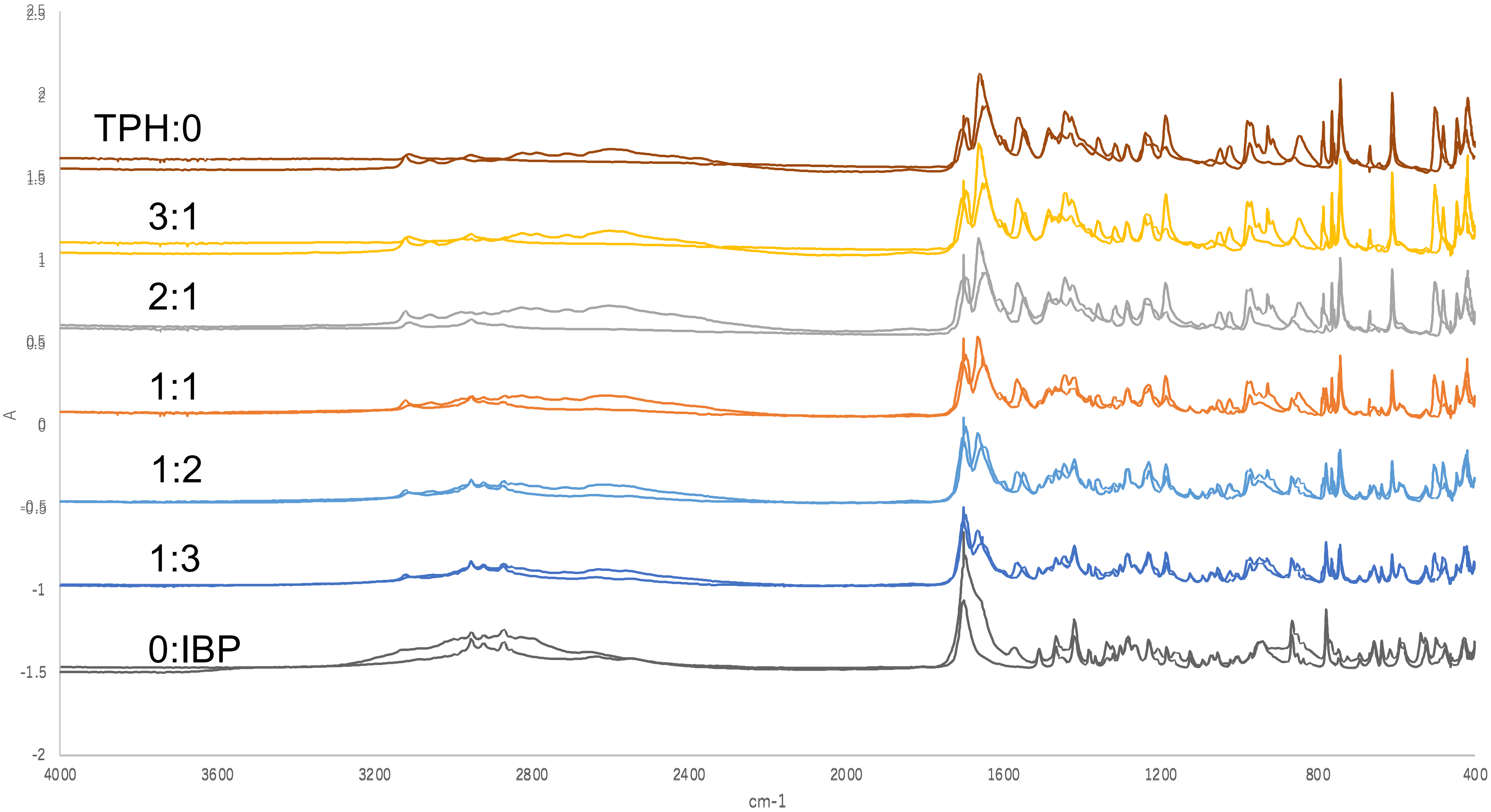

Figure S5. (c) Crystalline FTIR spectra of mixtures of IBP with TPH at various molar ratios. Neat TPH (brown), neat IBP (black), and their mixtures at the ratios of 3:1 (yellow), 2:1 (gray), 1:1 (orange), 1:2 (sky blue), and 1:3 (blue) were measured twice.

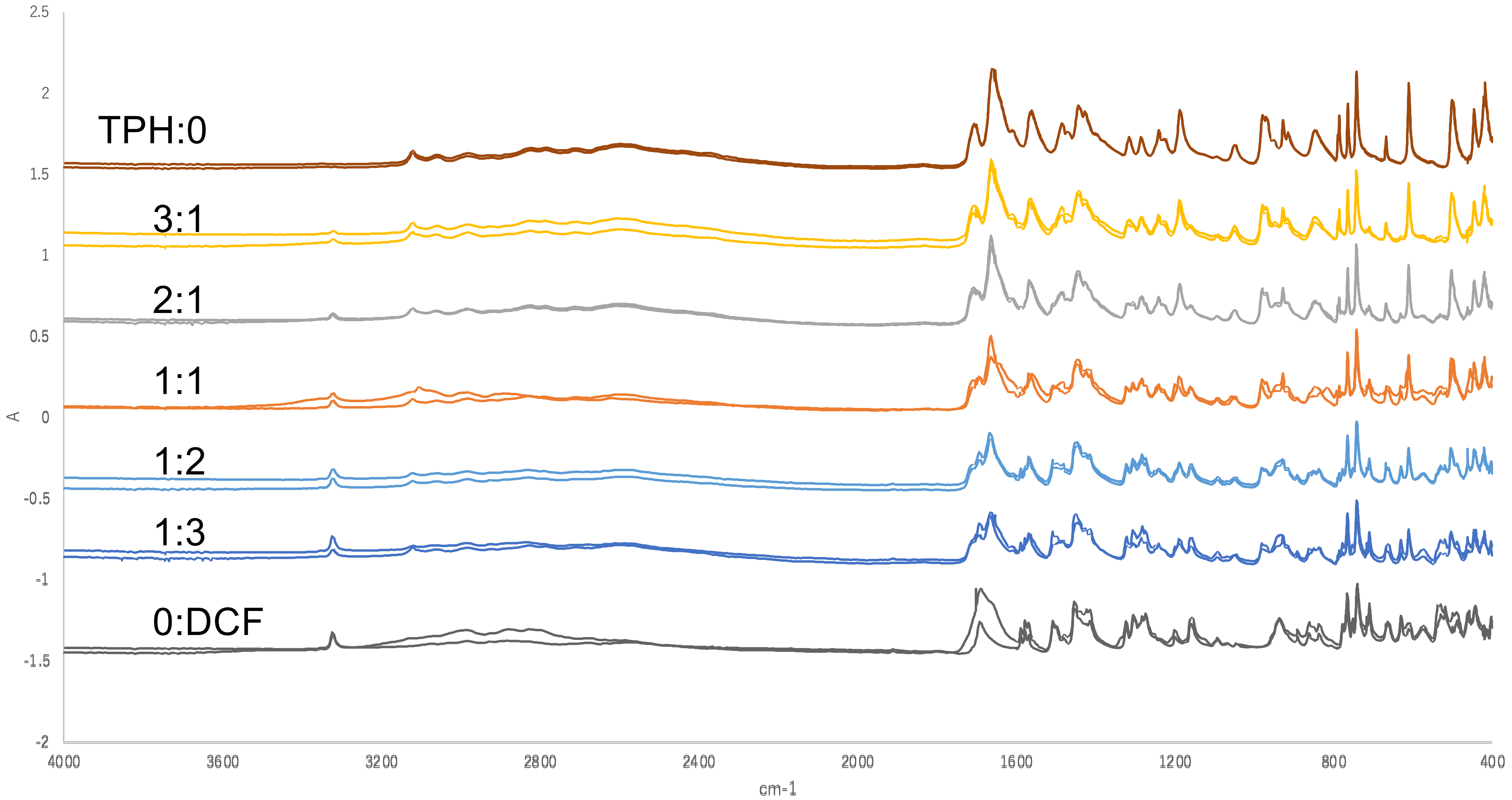

Figure S5. (d) Crystalline FTIR spectra of mixtures of DCF with TPH at various molar ratios. Neat TPH (brown), neat DCF (black), and their mixtures at the ratios of 3:1 (yellow), 2:1 (gray), 1:1 (orange), 1:2 (sky blue), and 1:3 (blue) were measured twice.

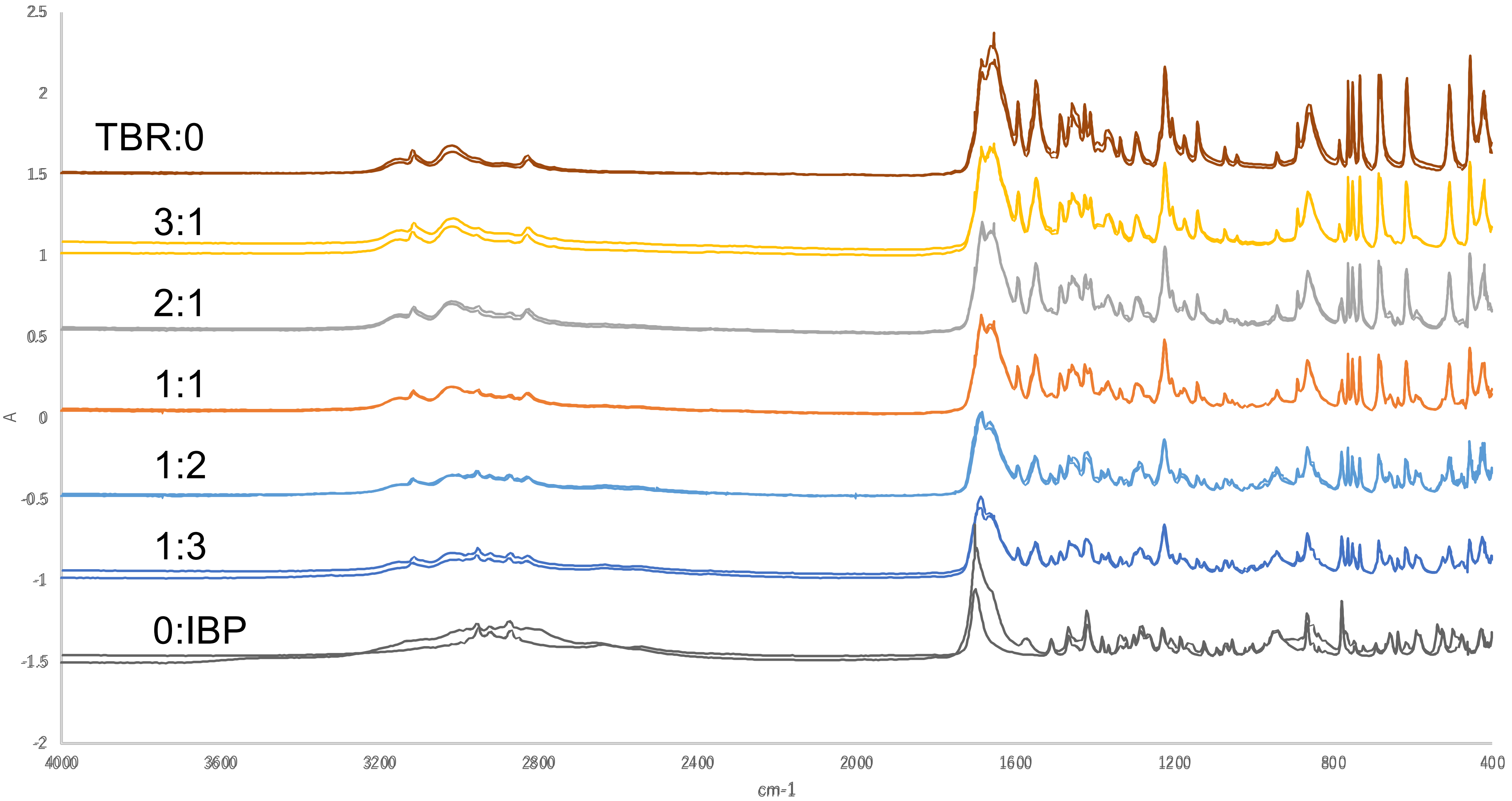

Figure S5. (e) Crystalline FTIR spectra of mixtures of IBP with TBR at various molar ratios. Neat TBR (brown), neat IBP (black), and their mixtures at the ratios of 3:1 (yellow), 2:1 (gray), 1:1 (orange), 1:2 (sky blue), and 1:3 (blue) were measured twice.

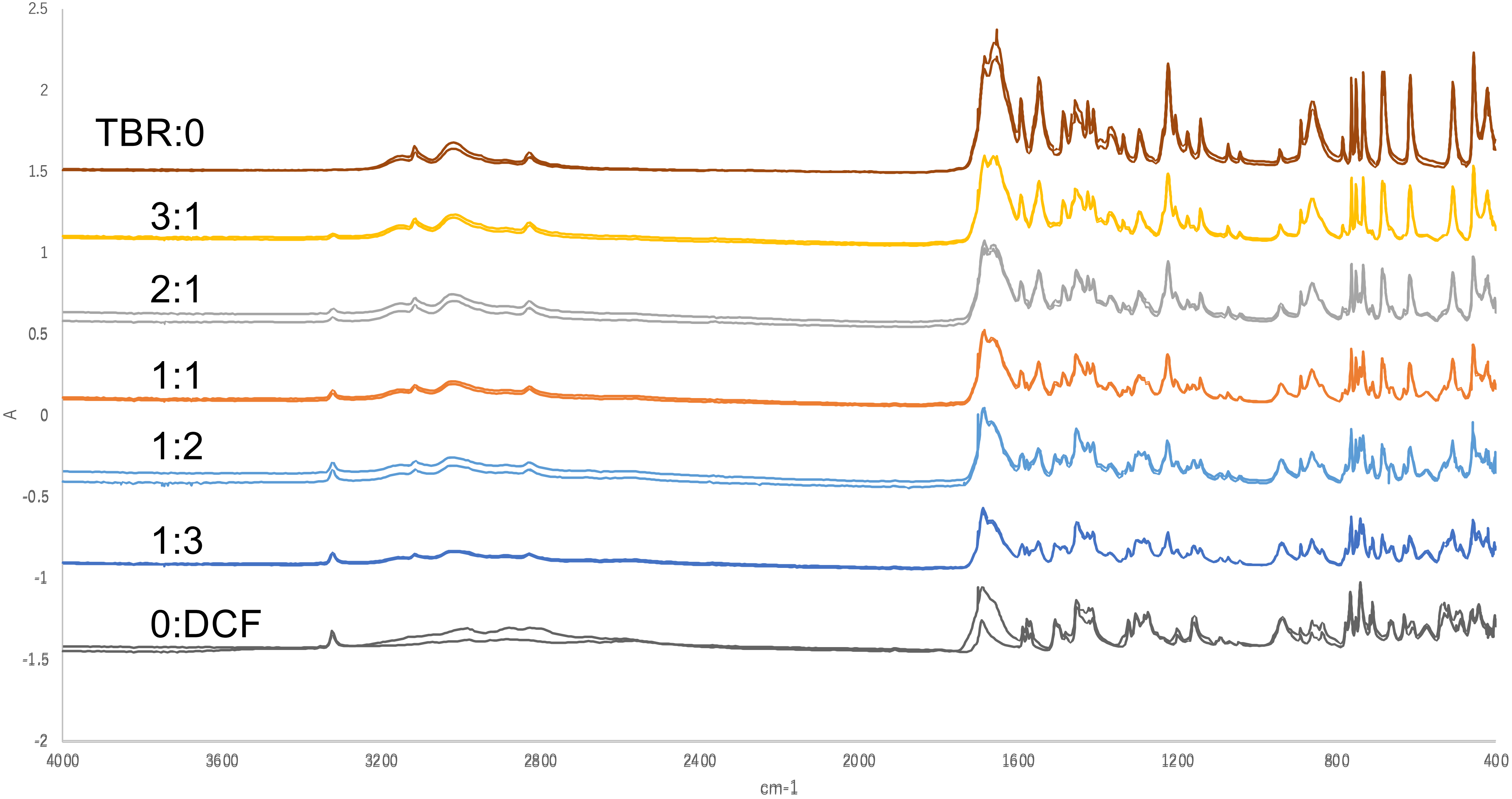

Figure S5. (f) Crystalline FTIR spectra of mixtures of DCF with TBR at various molar ratios. Neat TBR (brown), neat DCF (black), and their mixtures at the ratios of 3:1 (yellow), 2:1 (gray), 1:1 (orange), 1:2 (sky blue), and 1:3 (blue) were measured twice.

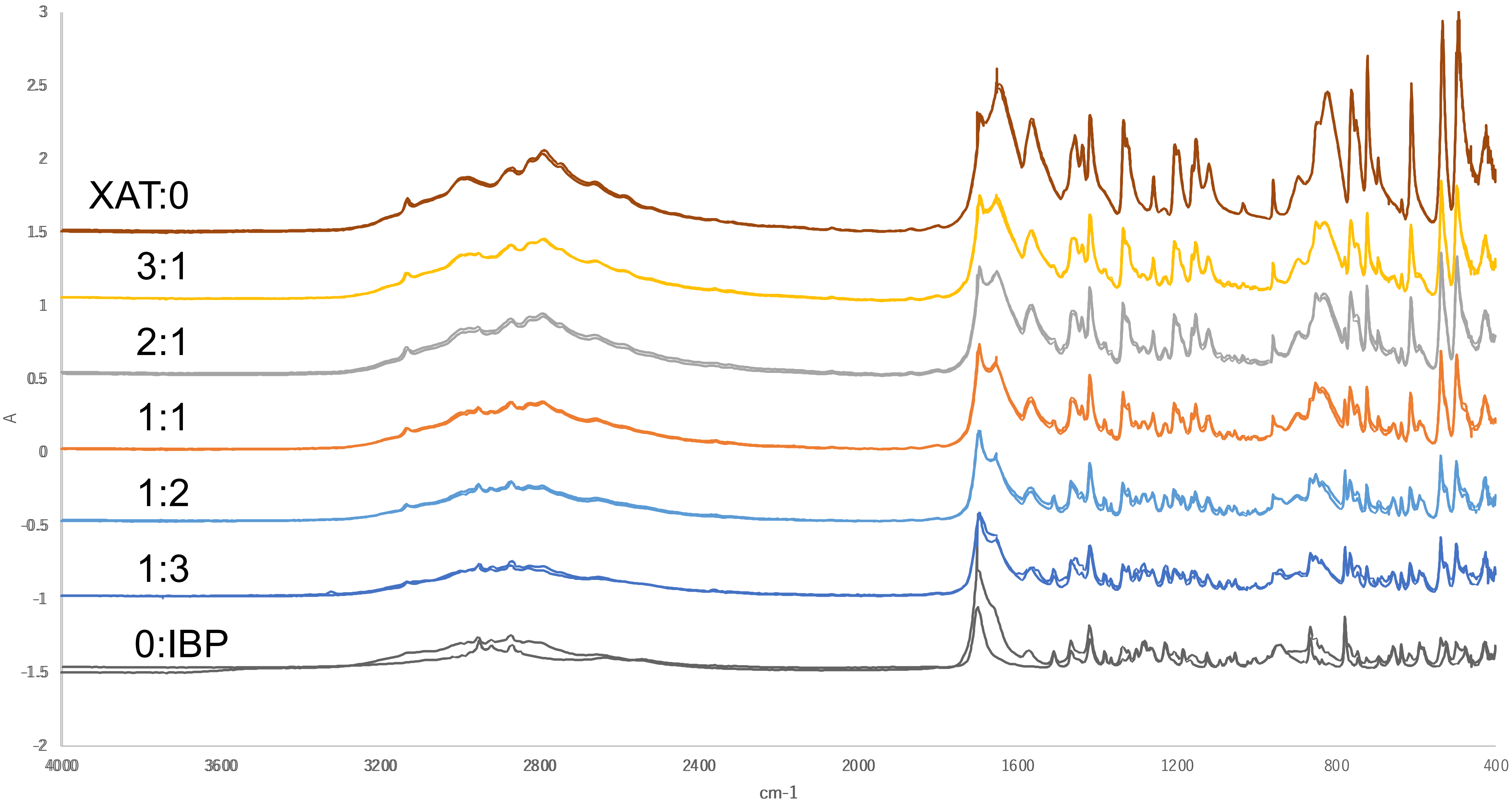

Figure S5. (g) Crystalline FTIR spectra of mixtures of IBP with XAT at various molar ratios. Neat XAT (brown), neat IBP (black), and their mixtures at the ratios of 3:1 (yellow), 2:1 (gray), 1:1 (orange), 1:2 (sky blue), and 1:3 (blue) were measured twice.

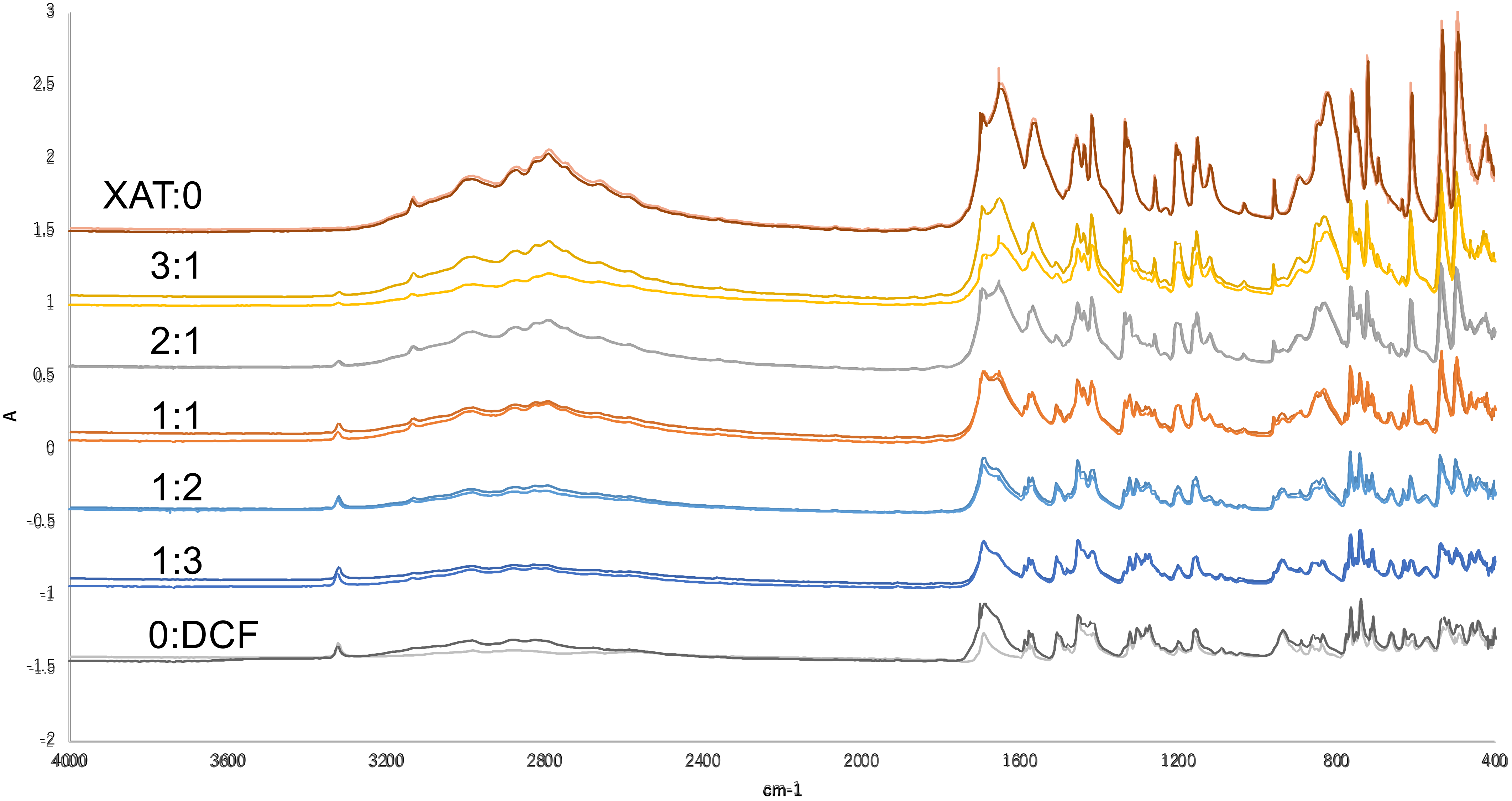

Figure S5. (h) Crystalline FTIR spectra of mixtures of DCF with XAT at various molar ratios. Neat XAT (brown), neat DCF (black), and their mixtures at the ratios of 3:1 (yellow), 2:1 (gray), 1:1 (orange), 1:2 (sky blue), and 1:3 (blue) were measured twice.

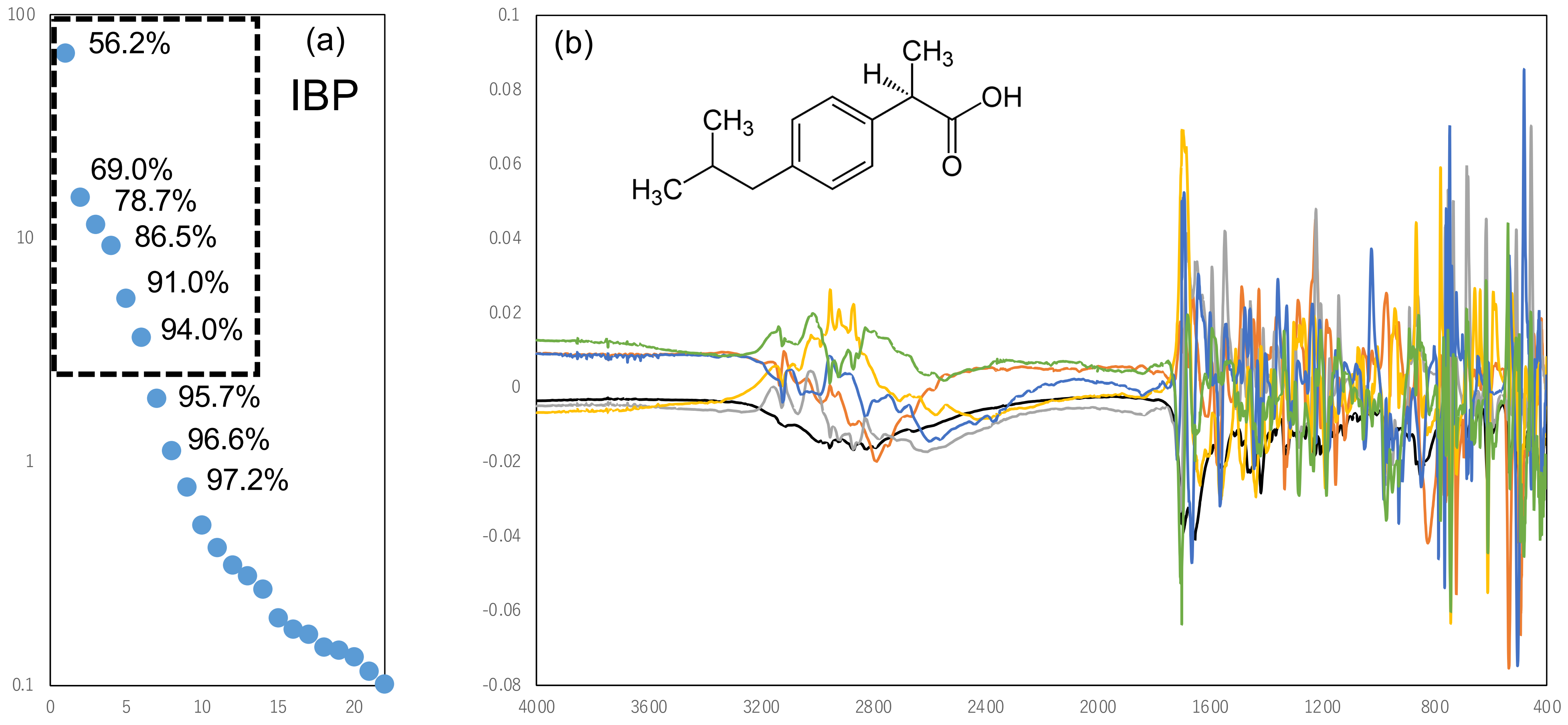

Figure S6. Summarized results of SVD computation for the IBP mixtures with the XAT derivatives: (a) the singular values and their corresponding cumulative sum of variances and (b) the first to sixth basis functions (94%). Curves in descending order were represented by colors of black, orange, gray, yellow, blue, and green.

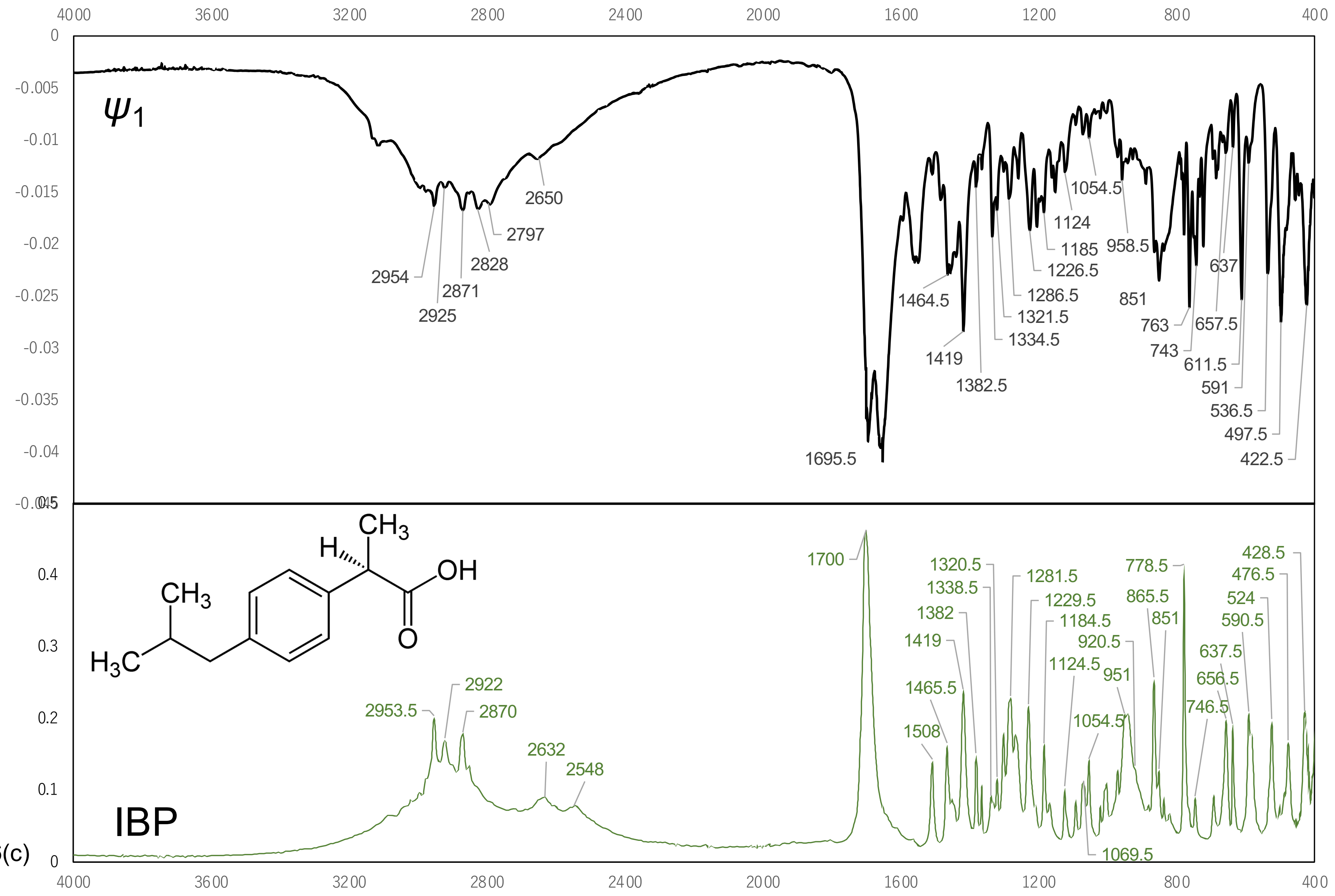

ψ1
IBP
1695.5
1700
2954
2925
2871
2828
2797
2650
2953.5
2922
2870
2632
2548

Figure S6(c)

Figure S6(d)

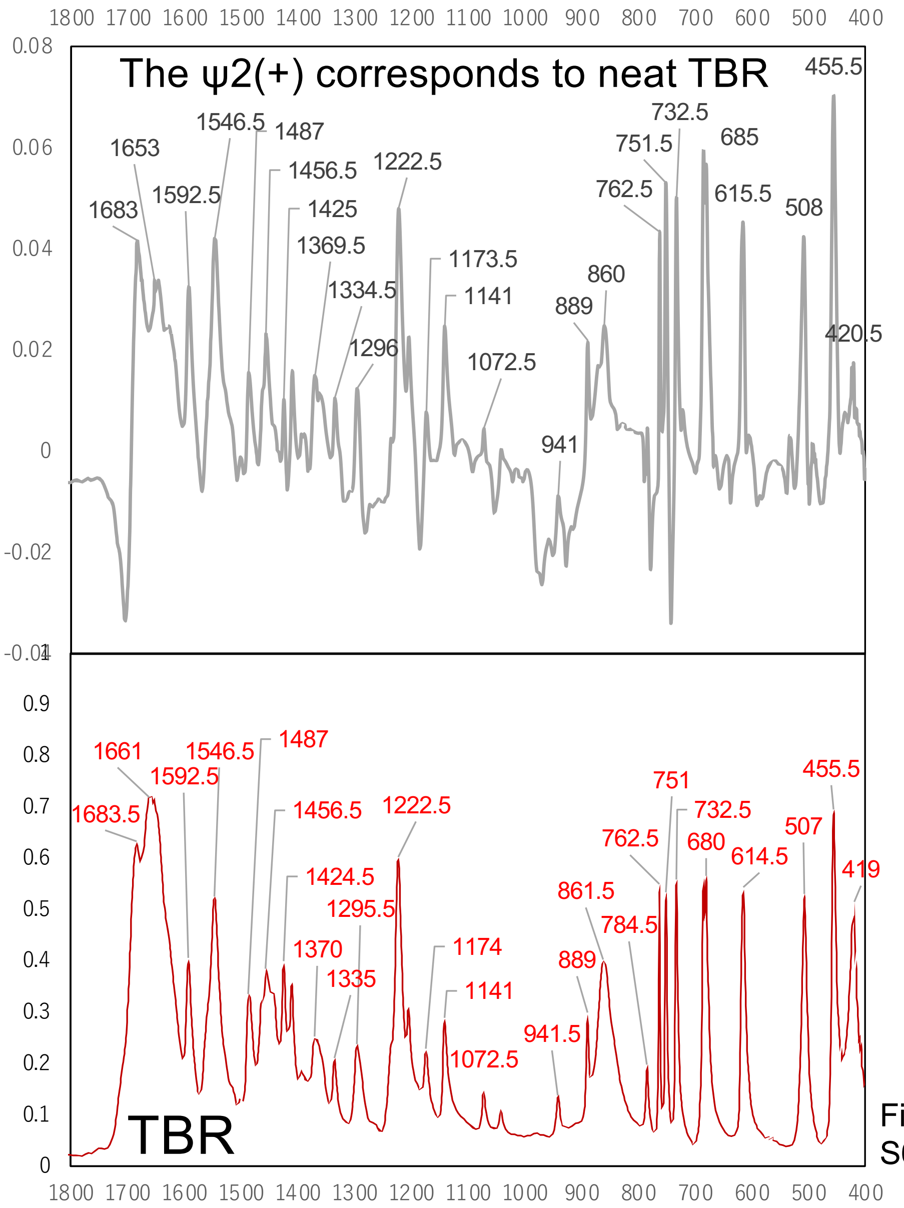

Figure S6(e)

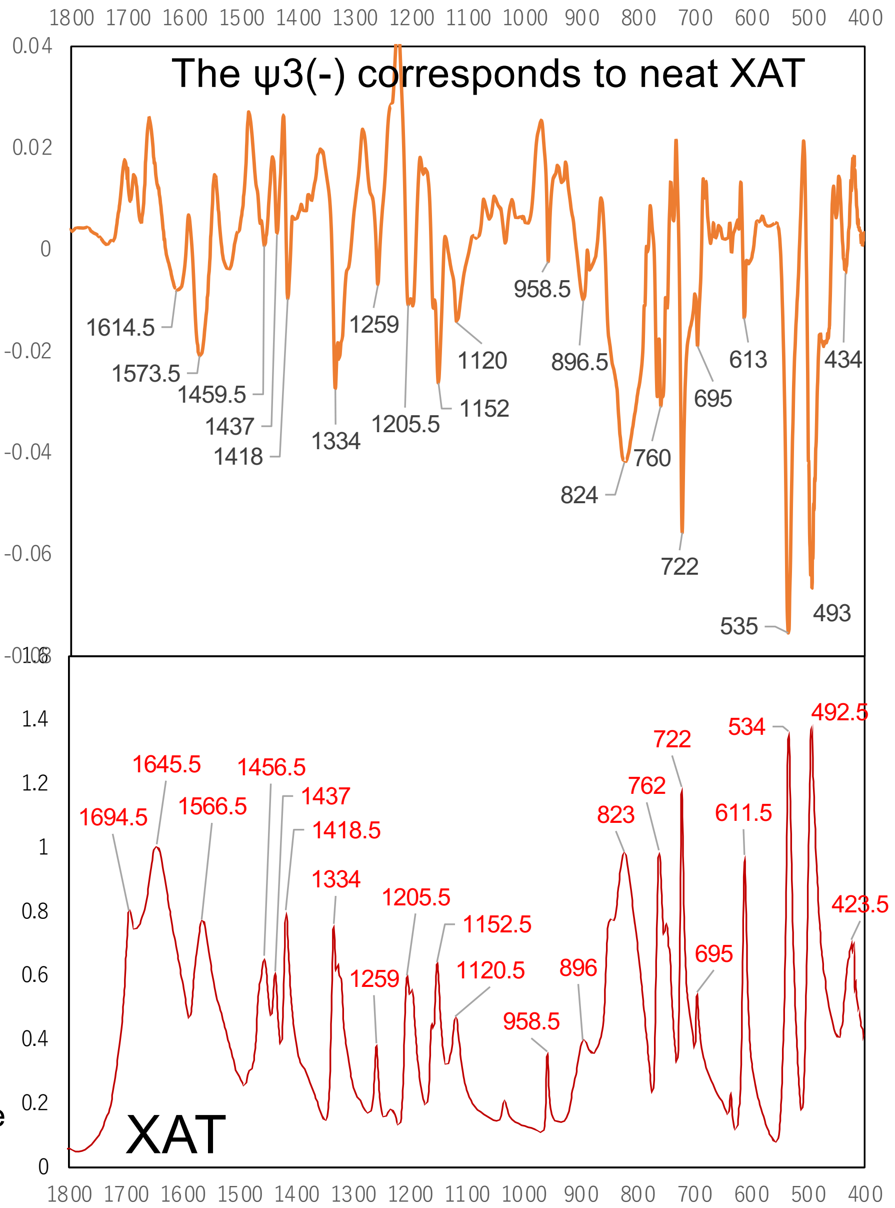

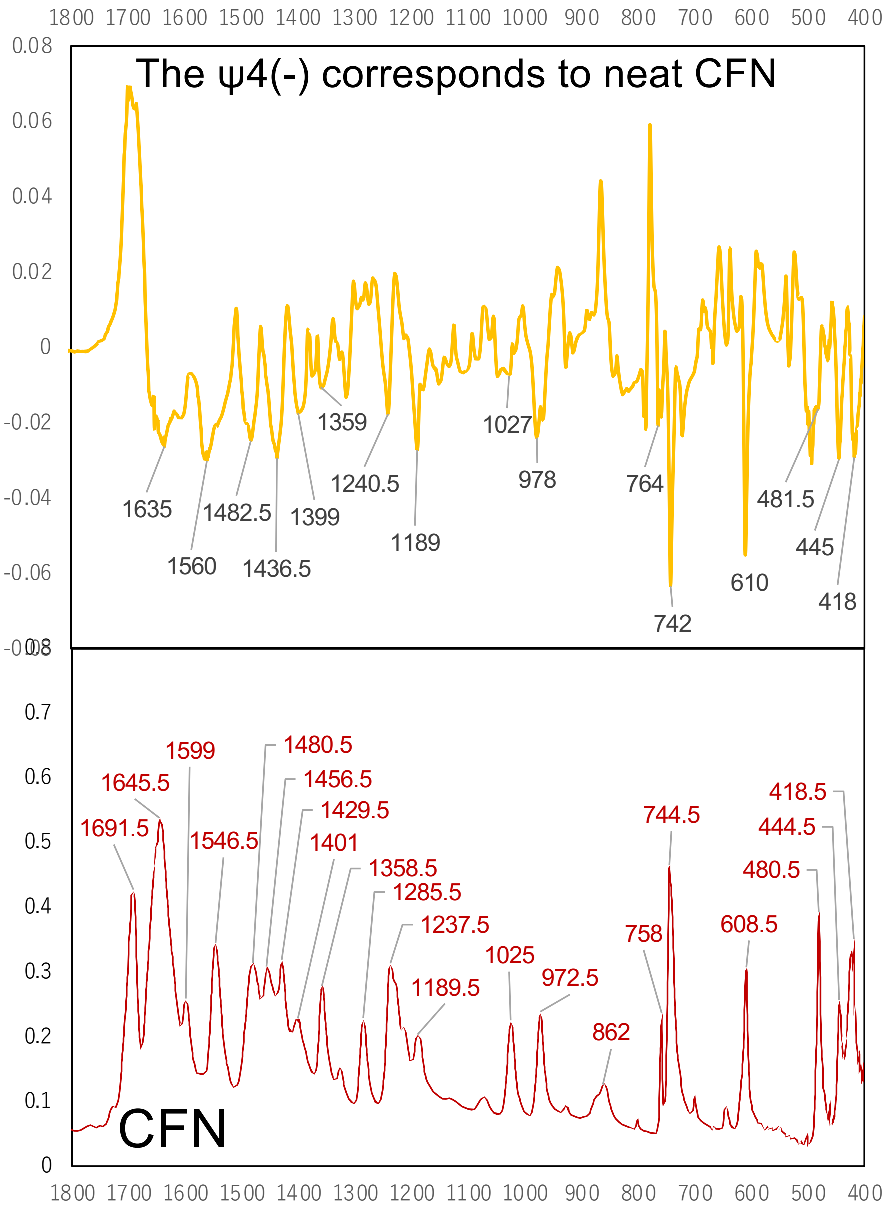

Figure S6(f)

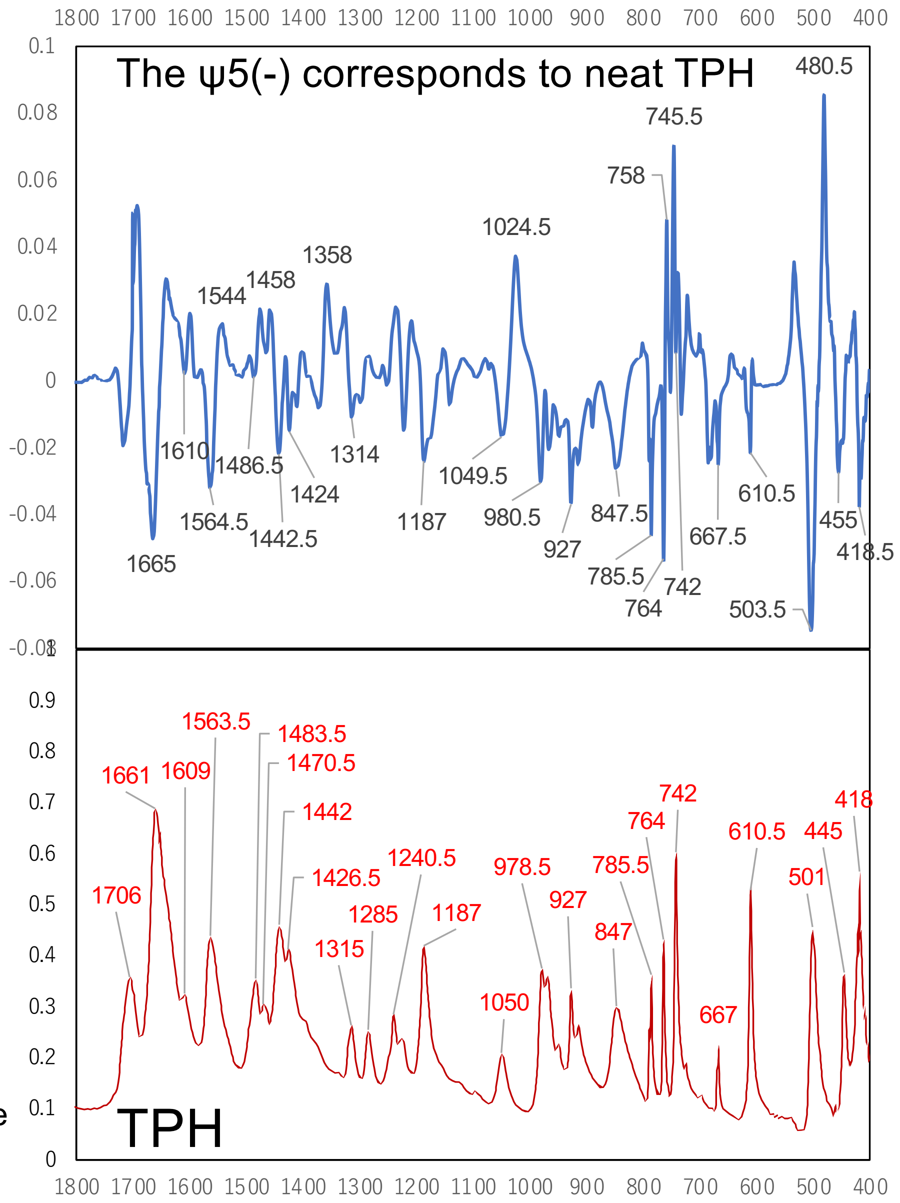

Figure S6(g)

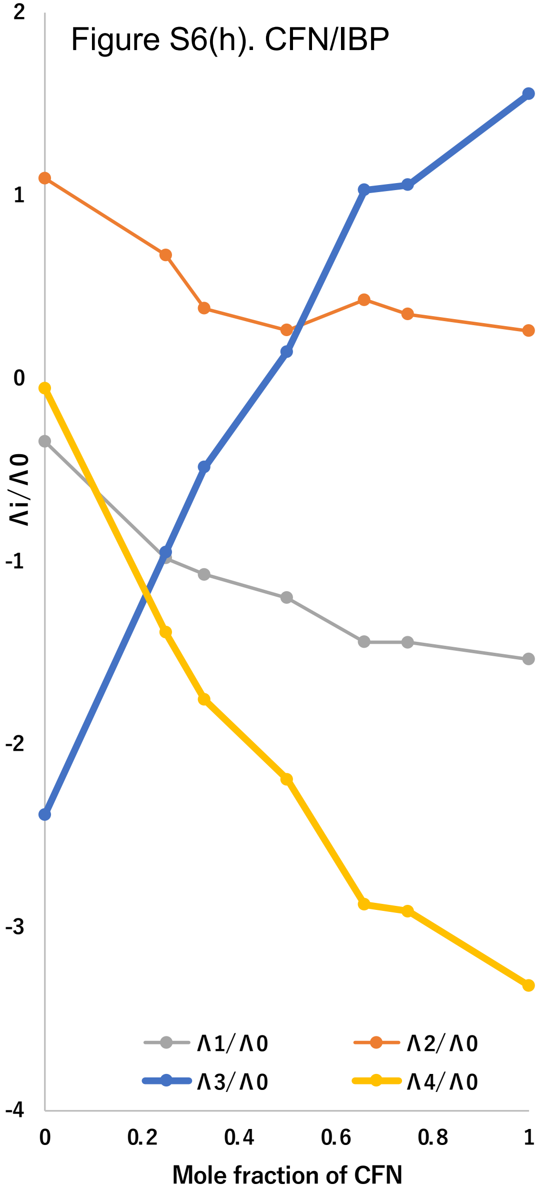
Figure S6(h). CFN/IBP
Λi/Λ0
2
1
0
-1
-2
-3
-4
0
0.2
0.4
0.6
0.8
1
Mole fraction of CFN
Λ1/Λ0
Λ2/Λ0
Λ3/Λ0
Λ4/Λ0

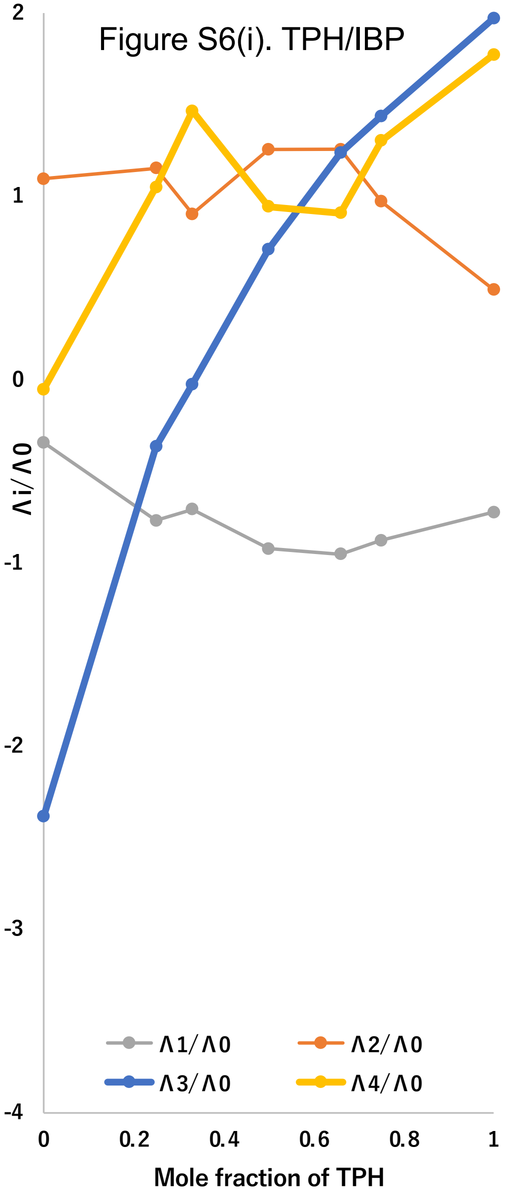
Figure S6(i). TPH/IBP
Λi/Λ0
2
1
0
-1
-2
-3
-4
0
0.2
0.4
0.6
0.8
1
Mole fraction of TPH
Λ1/Λ0
Λ2/Λ0
Λ3/Λ0
Λ4/Λ0

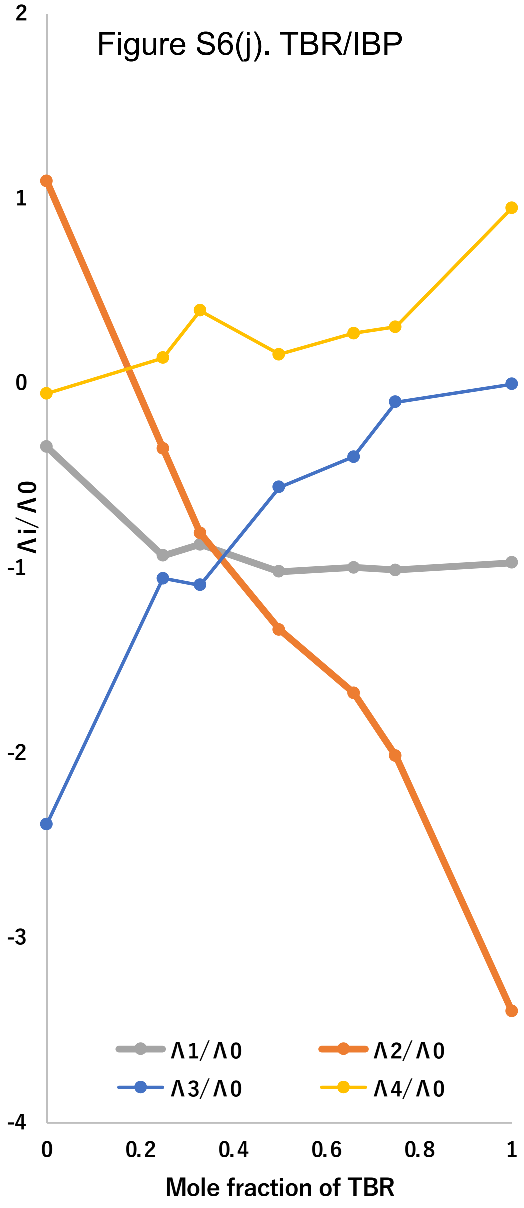
Figure S6(j). TBR/IBP
Λi/Λ0
2
1
0
-1
-2
-3
-4
0
0.2
0.4
0.6
0.8
1
Mole fraction of TBR
Λ1/Λ0
Λ2/Λ0
Λ3/Λ0
Λ4/Λ0

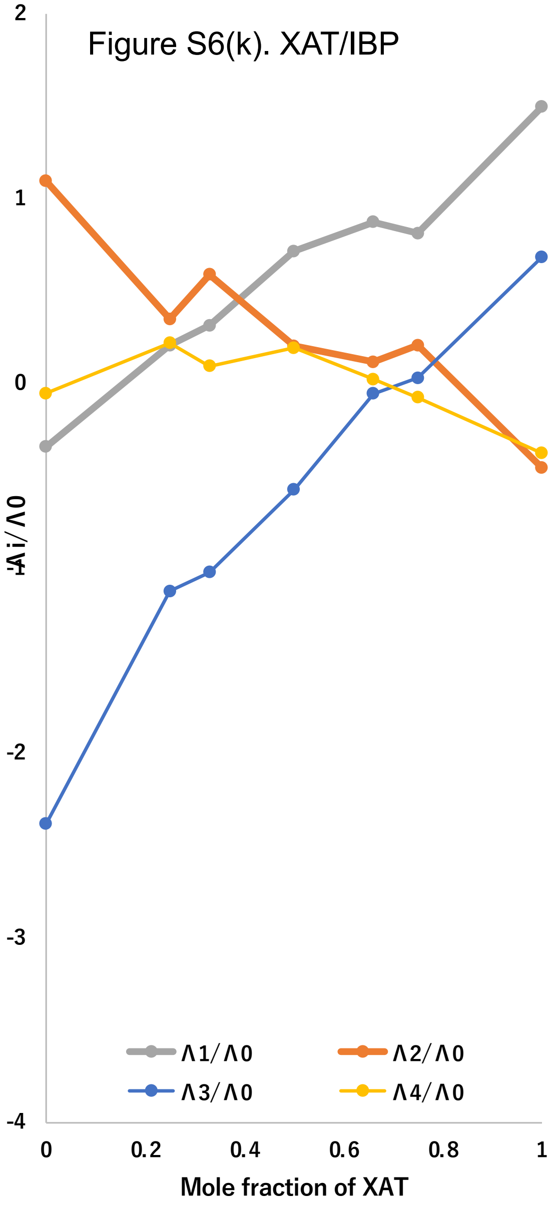
Figure S6(k). XAT/IBP
Λi/Λ0
2
1
0
-1
-2
-3
-4
0
0.2
0.4
0.6
0.8
1
Mole fraction of XAT
Λ1/Λ0
Λ2/Λ0
Λ3/Λ0
Λ4/Λ0

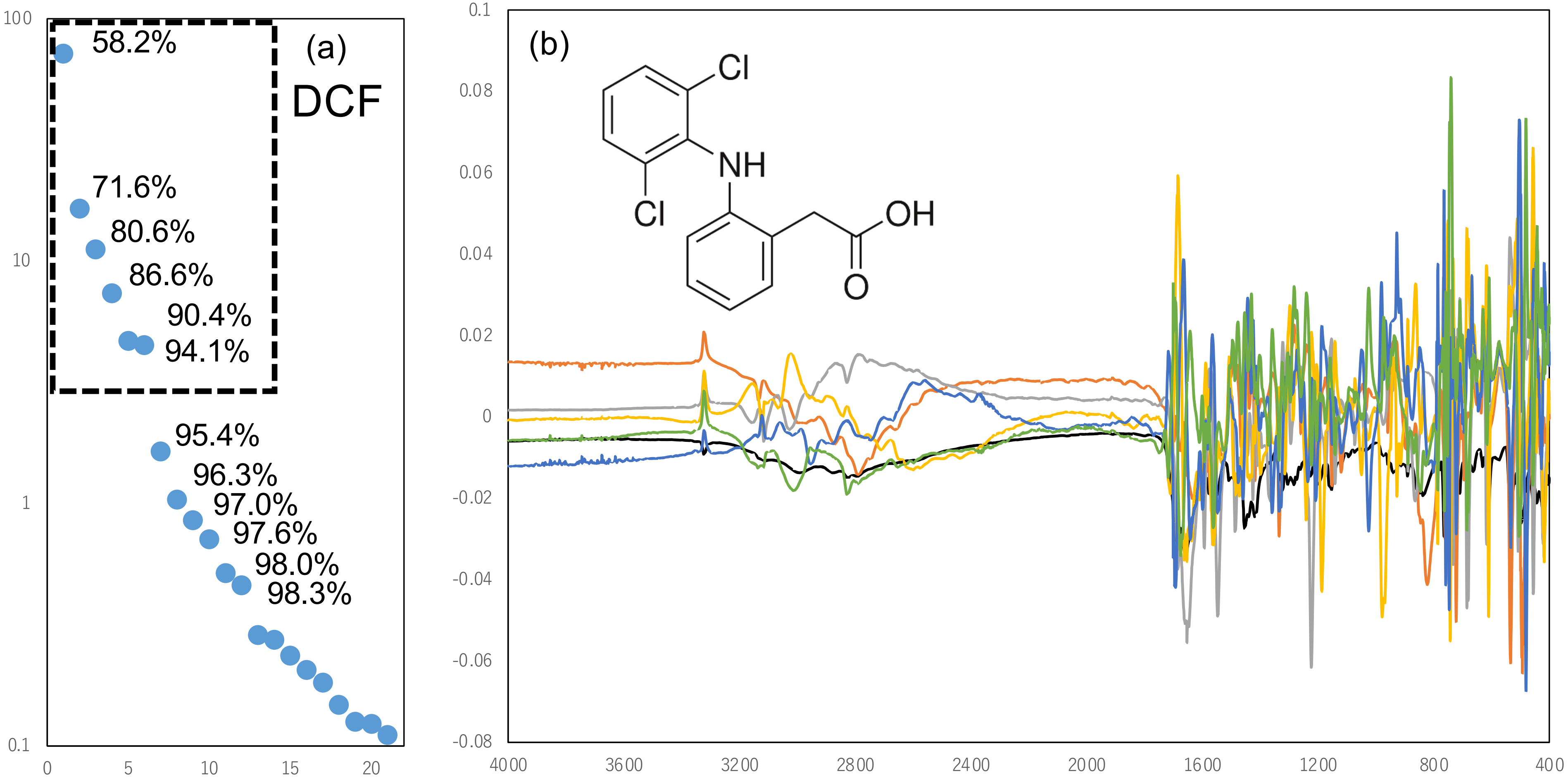

Figure S7. Summarized results of SVD computation for the DCF mixtures with the XAT derivatives: (a) the singular values and their corresponding cumulative sum of variances and (b) the first to sixth basis functions (94%). Curves in descending order were represented by colors of black, orange, gray, yellow, blue, and green.

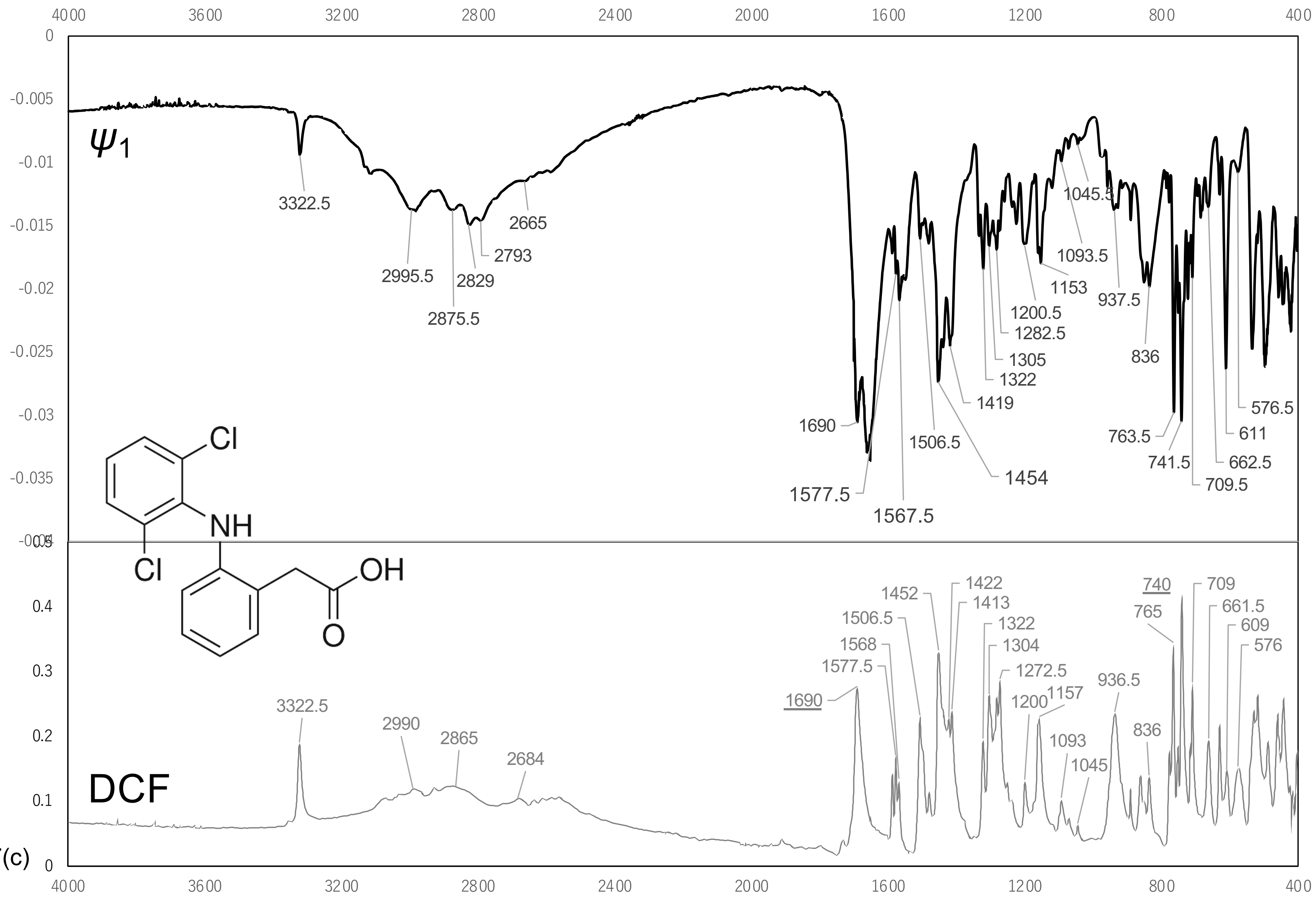

ψ1
DCF
Cl
NH
Cl
OH
O
3322.5
2995.5
2875.5
2829
2793
2665
1690
1577.5
1567.5
1506.5
1454
1419
1322
1305
1282.5
1200.5
1153
1093.5
1045.5
937.5
836
763.5
741.5
709.5
662.5
611
576.5
2990
2865
2684
1568
1452
1422
1413
1304
1272.5
1200
1157
1093
1045
936.5
765
740
709
661.5
609
576

Figure S7(c)

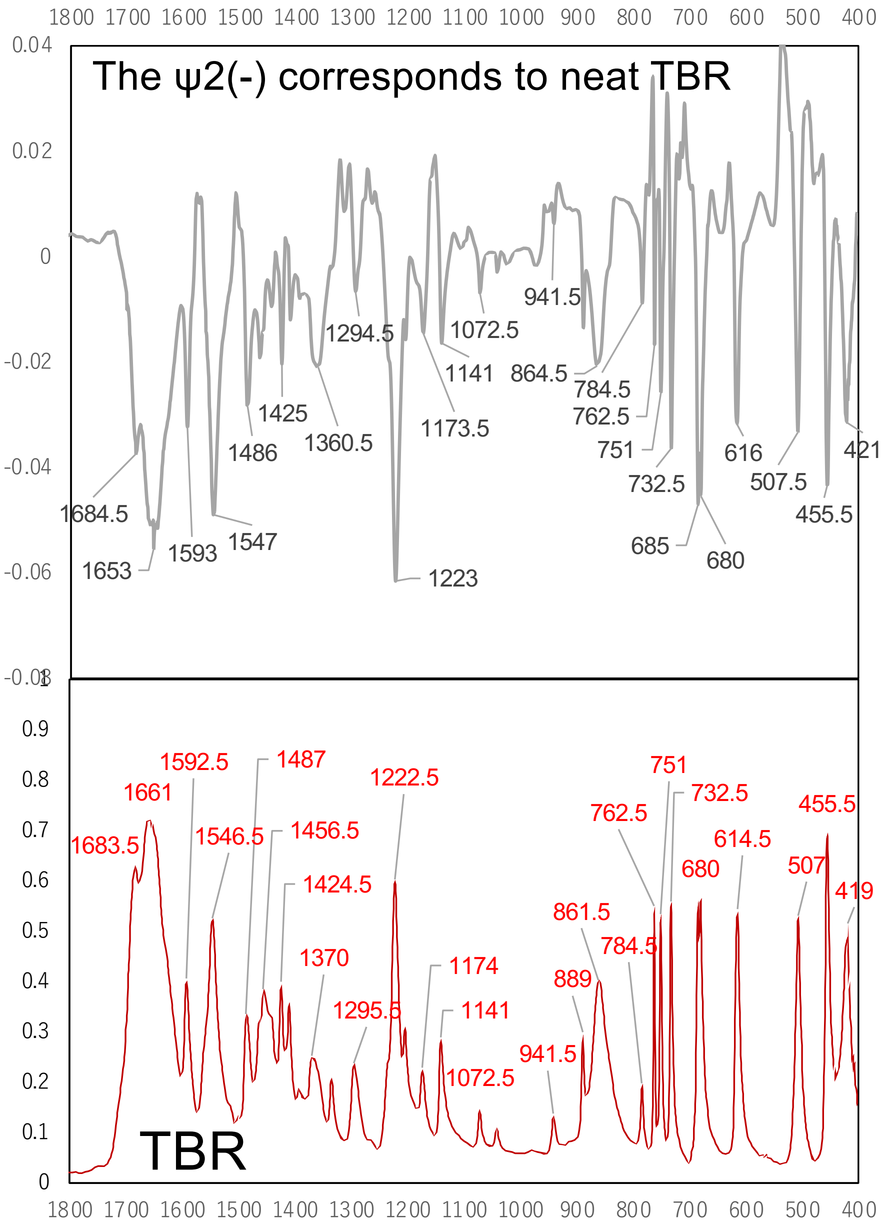

Figure S7(d)

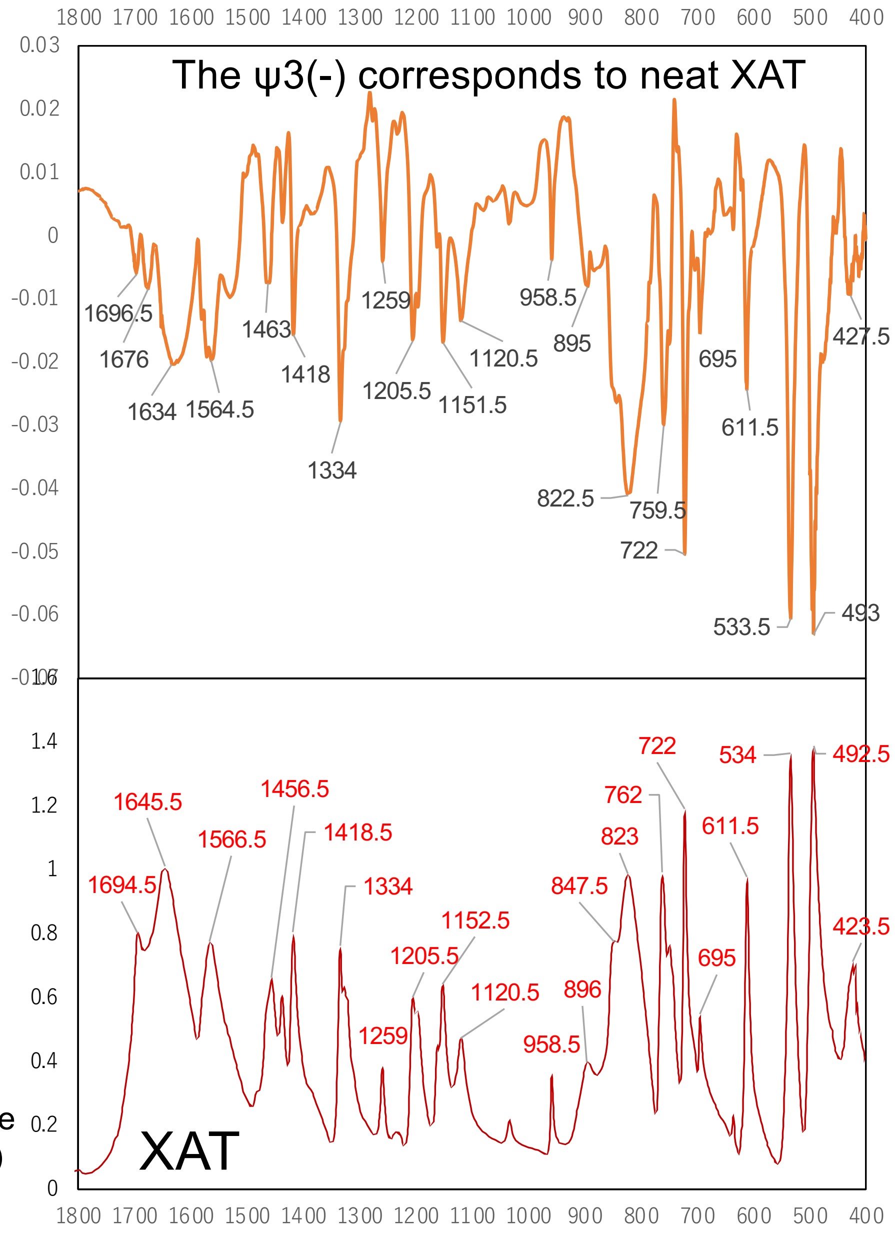

Figure S7(e)

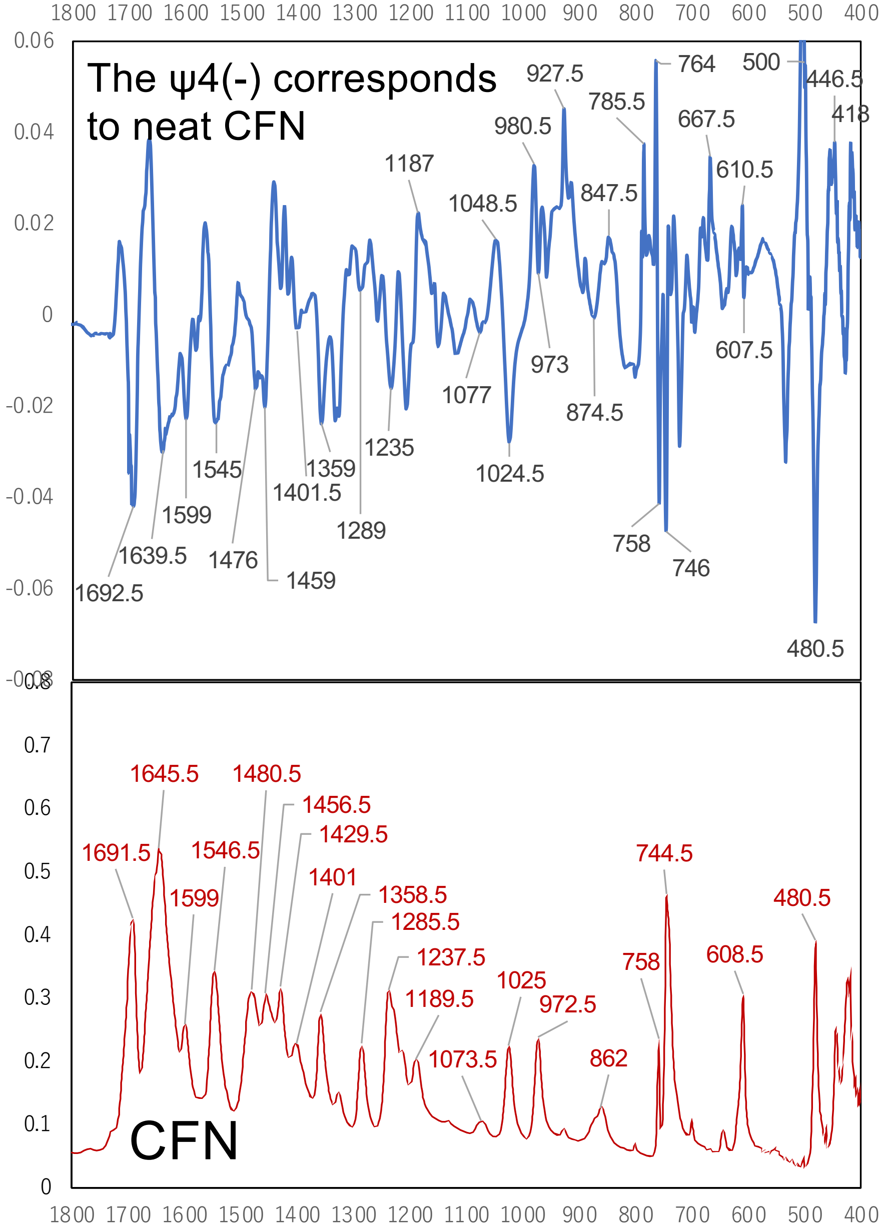

Figure S7(f)

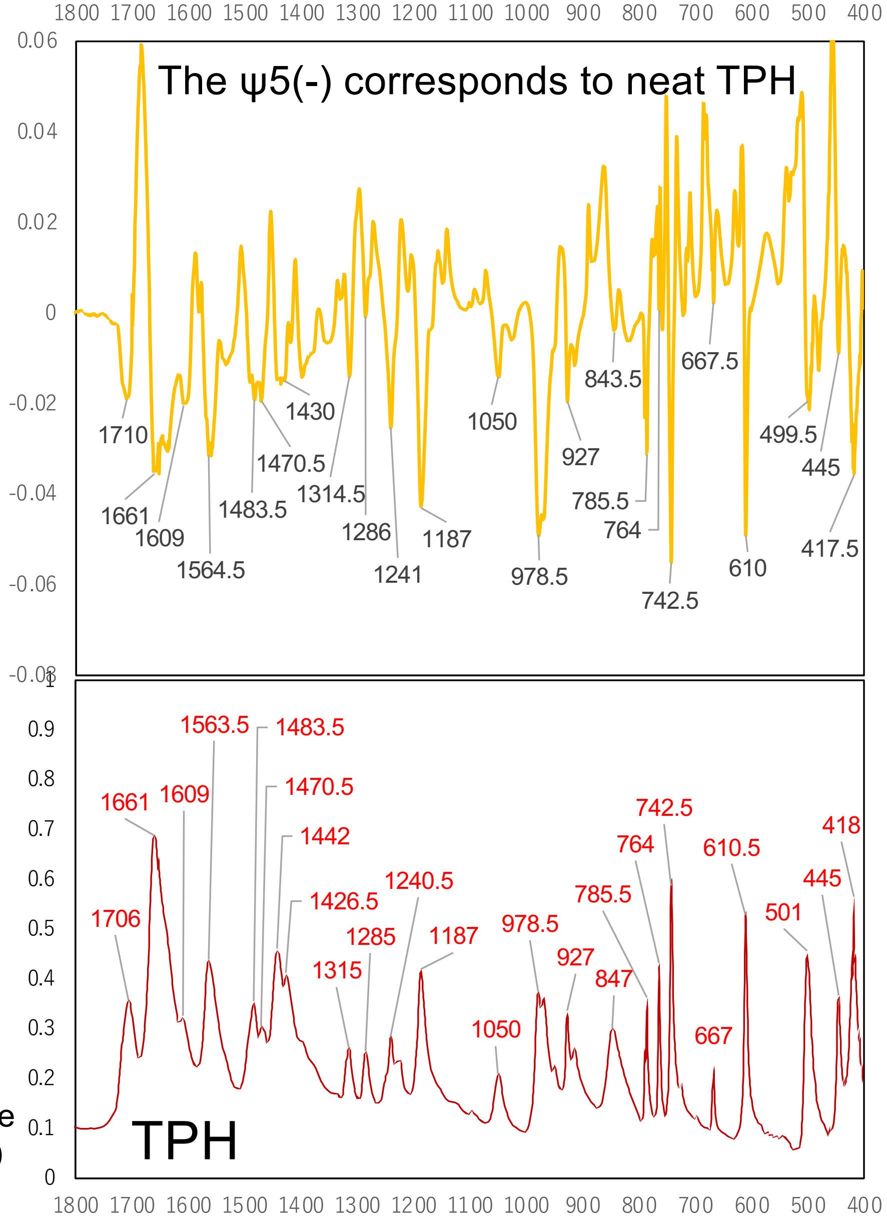

Figure S7(g)

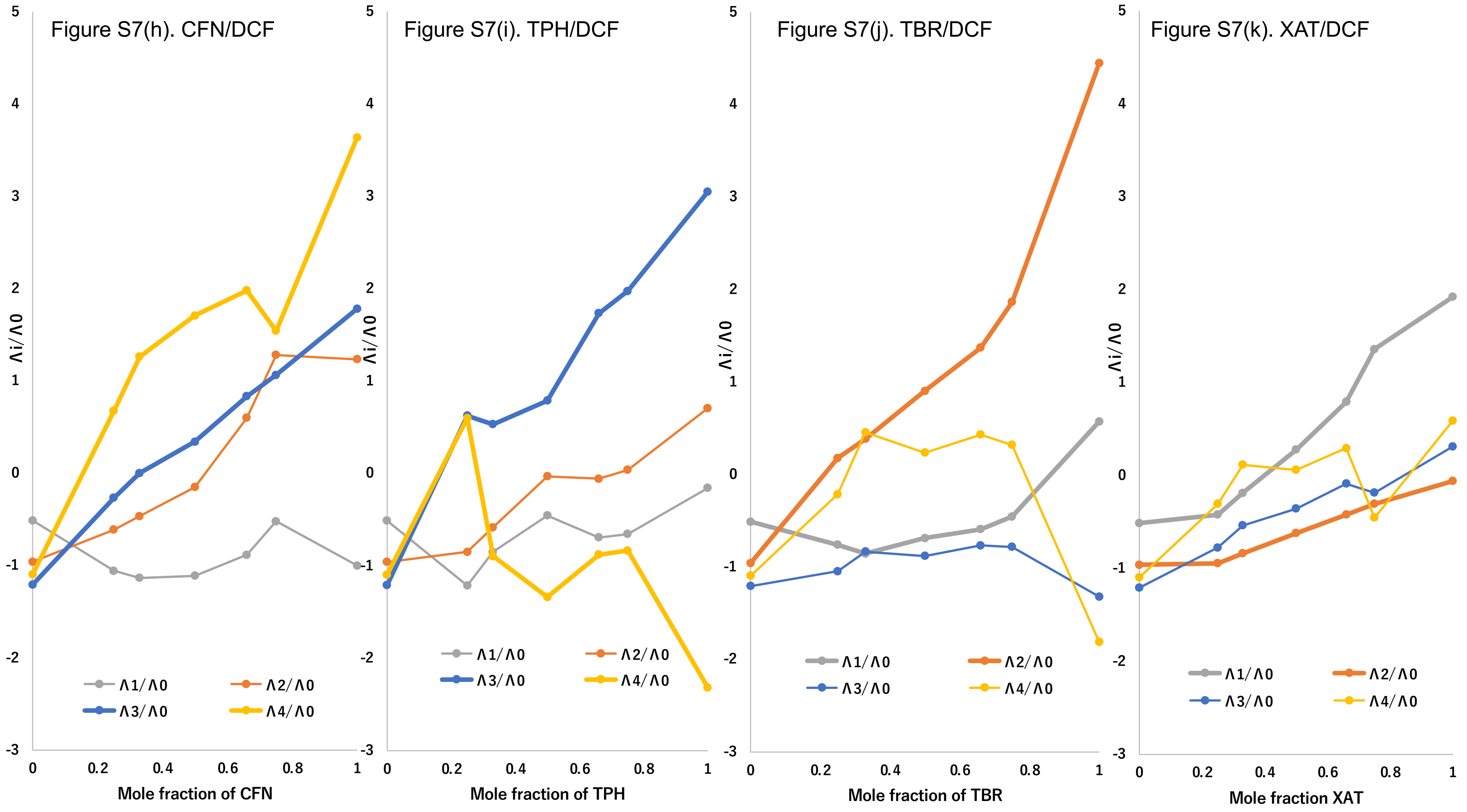
Figure S7(h). CFN/DCF
Λi/Λ0
Mole fraction of CFN
Λ1/Λ0
Λ2/Λ0
Λ3/Λ0
Λ4/Λ0
Figure S7(i). TPH/DCF
Λi/Λ0
Mole fraction of TPH
Λ1/Λ0
Λ2/Λ0
Λ3/Λ0
Λ4/Λ0
Figure S7(j). TBR/DCF
Λi/Λ0
Mole fraction of TBR
Λ1/Λ0
Λ2/Λ0
Λ3/Λ0
Λ4/Λ0
Figure S7(k). XAT/DCF
Λi/Λ0
Mole fraction XAT
Λ1/Λ0
Λ2/Λ0
Λ3/Λ0
Λ4/Λ0

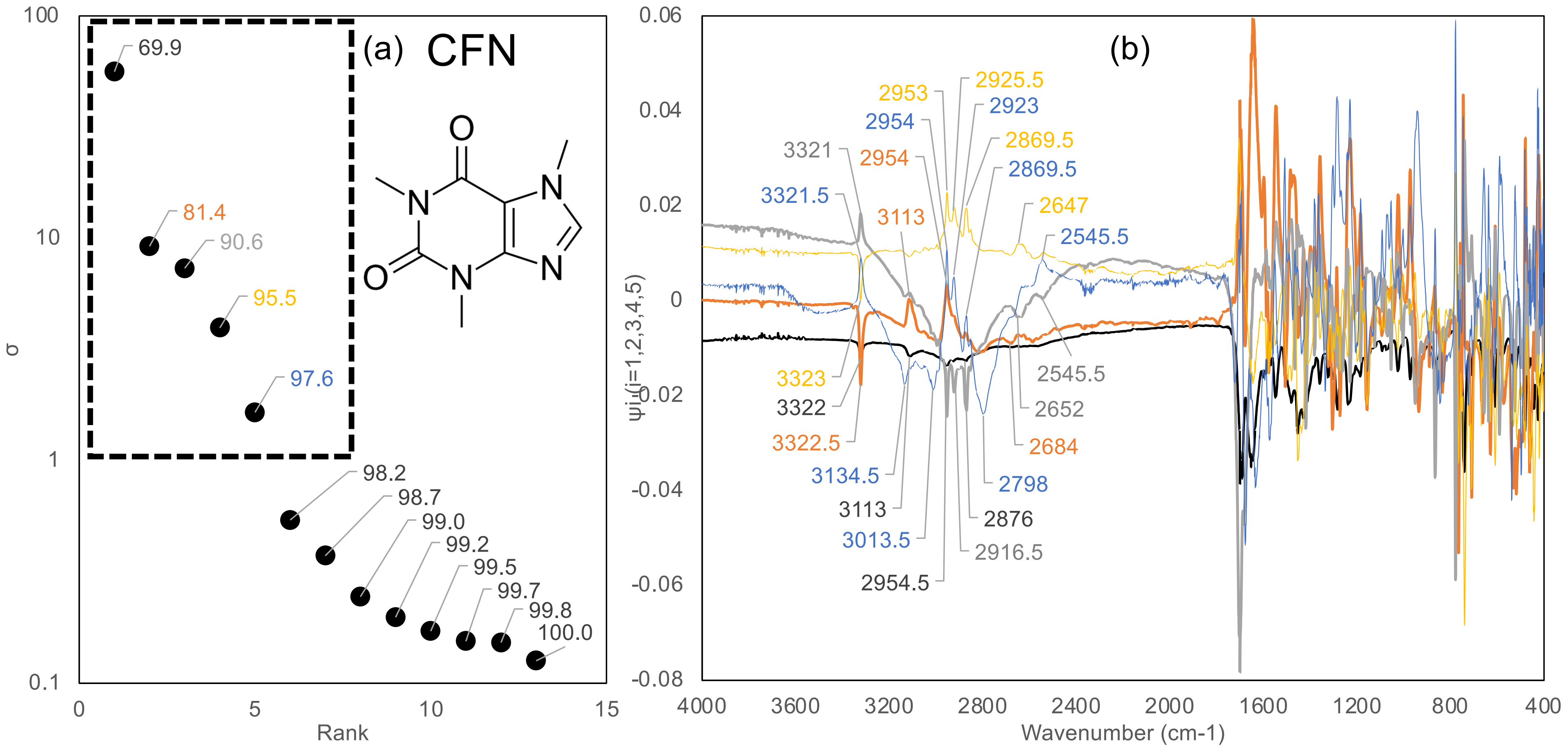

Figure S8. SVD computation for FTIR spectra of CFN/IBP and CFN/DCF mixtures provided singular values σi (a) and Basis spectra ψi(b). Cumulative proportions of σi were appended beside the σ plots, indicating that higher five σ's satisfied 97.6 percent of the variance. The basis spectra decomposed by the SVD computation corresponded to the components ψ1 (black), ψ2 (orange), ψ3 (gray), ψ4 (yellow), and ψ5 (blue). Details of ψ's assignments to the observed spectra in the wavenumber range 1800-400 cm^{-1} are followed (e-o).

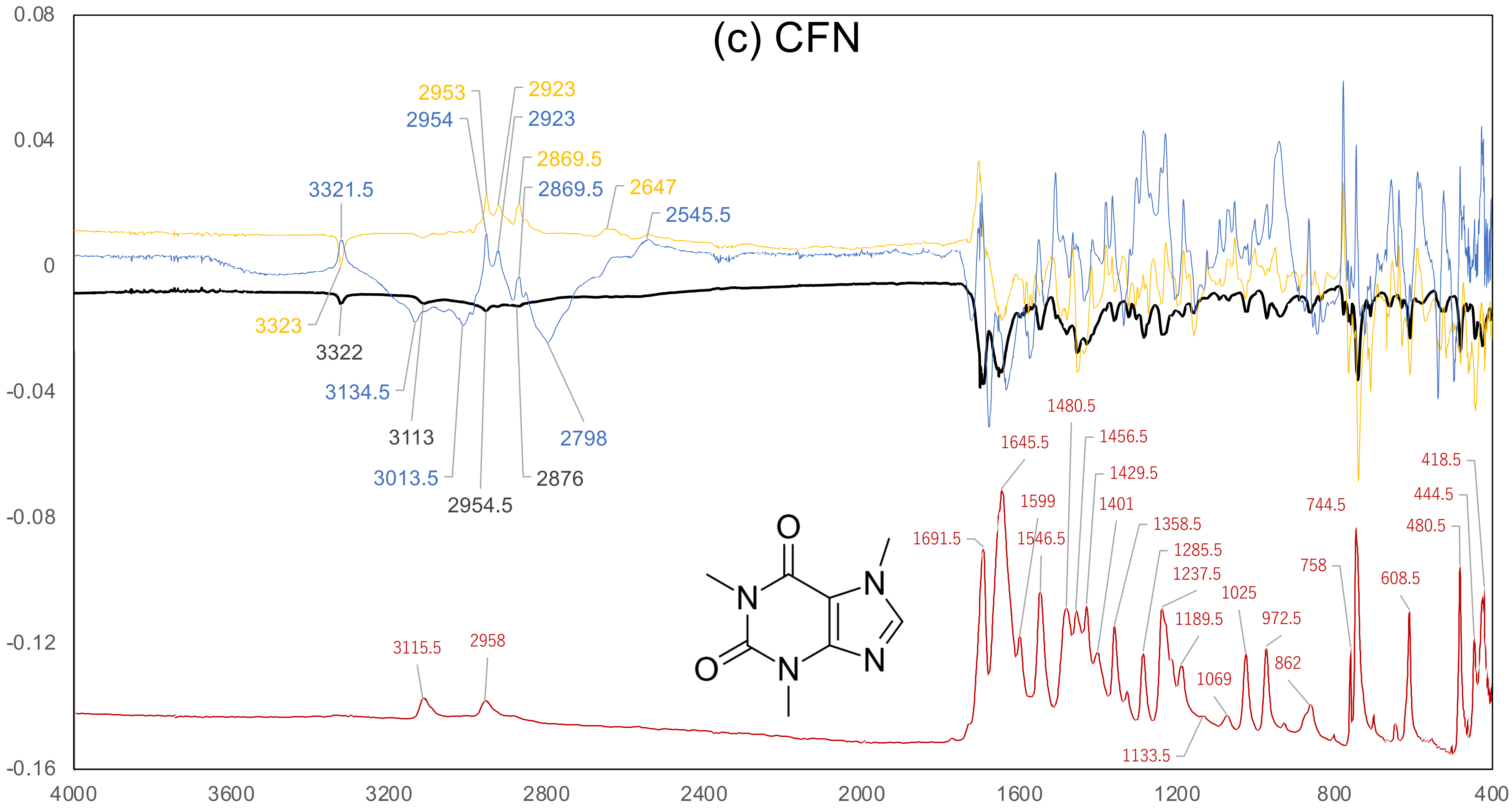

Figure S8. The basis spectra decomposed by the SVD computation corresponded to the components at the rank of 1 (black), 4 (yellow), and 5 (blue).

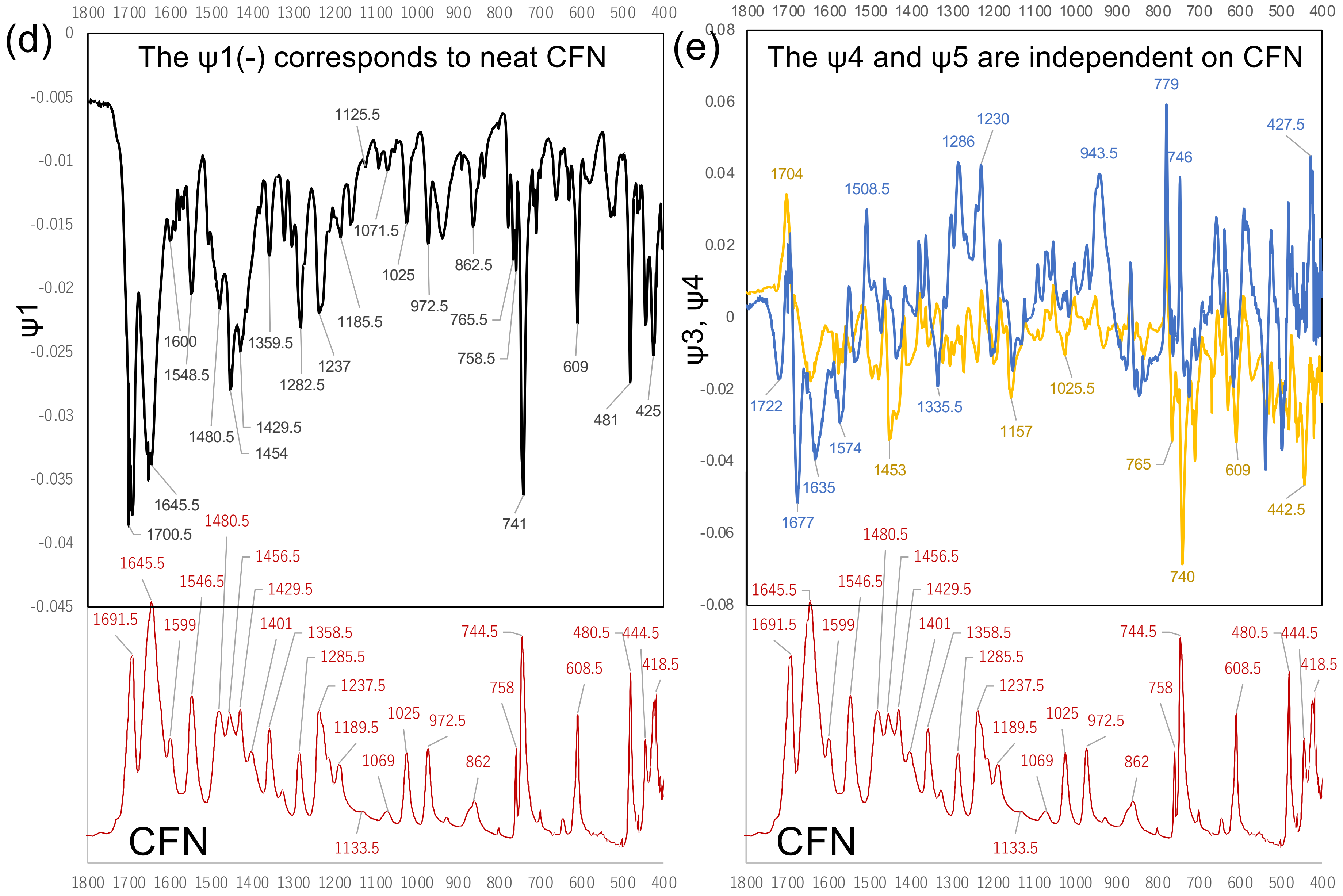

(d)
The ψ1(-) corresponds to neat CFN
ψ1
CFN
(e)
The ψ4 and ψ5 are independent on CFN
ψ3, ψ4
CFN

Figure S8

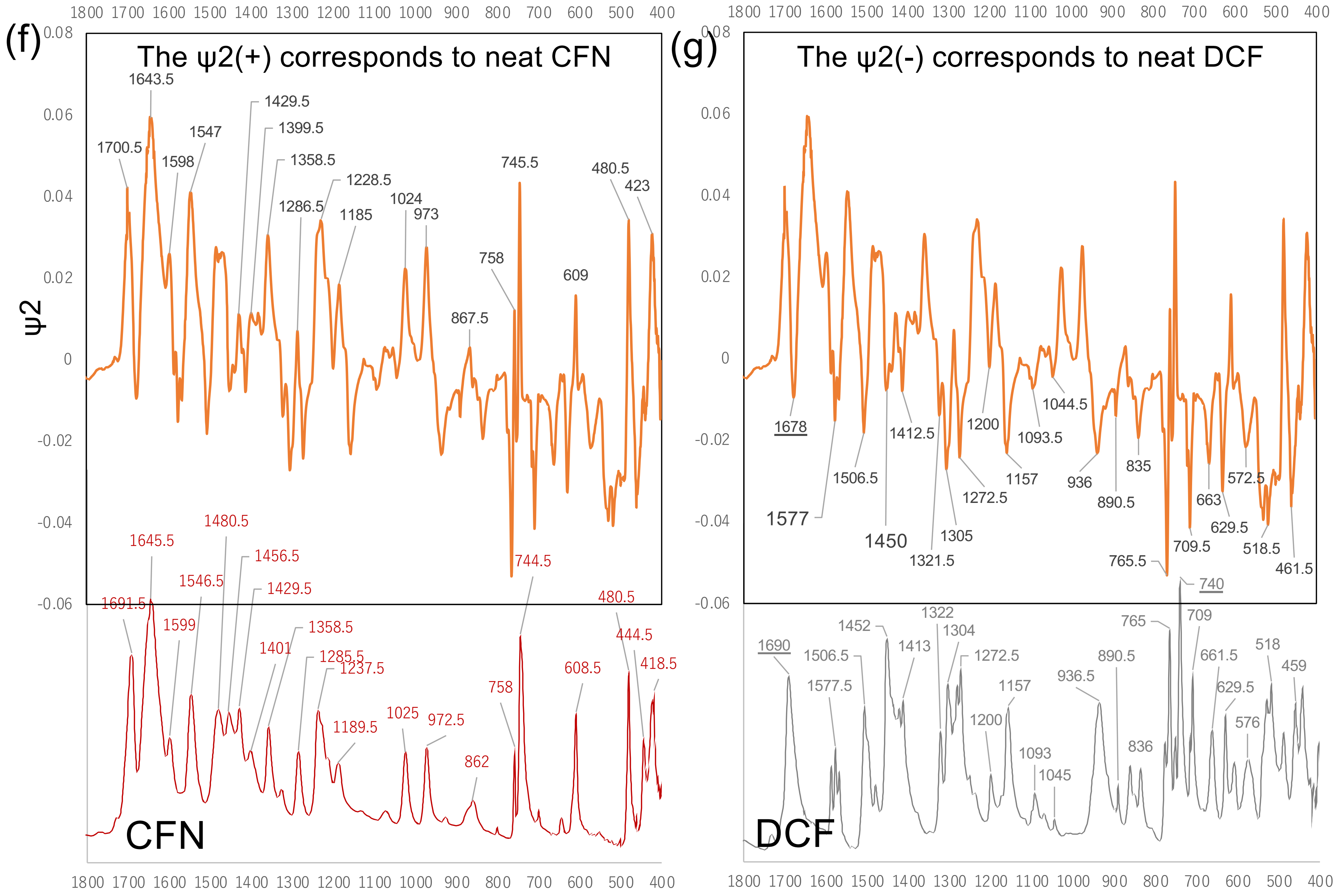
(f)
The ψ2(+) corresponds to neat CFN
ψ2
CFN
(g)
The ψ2(-) corresponds to neat DCF
DCF

Figure S8

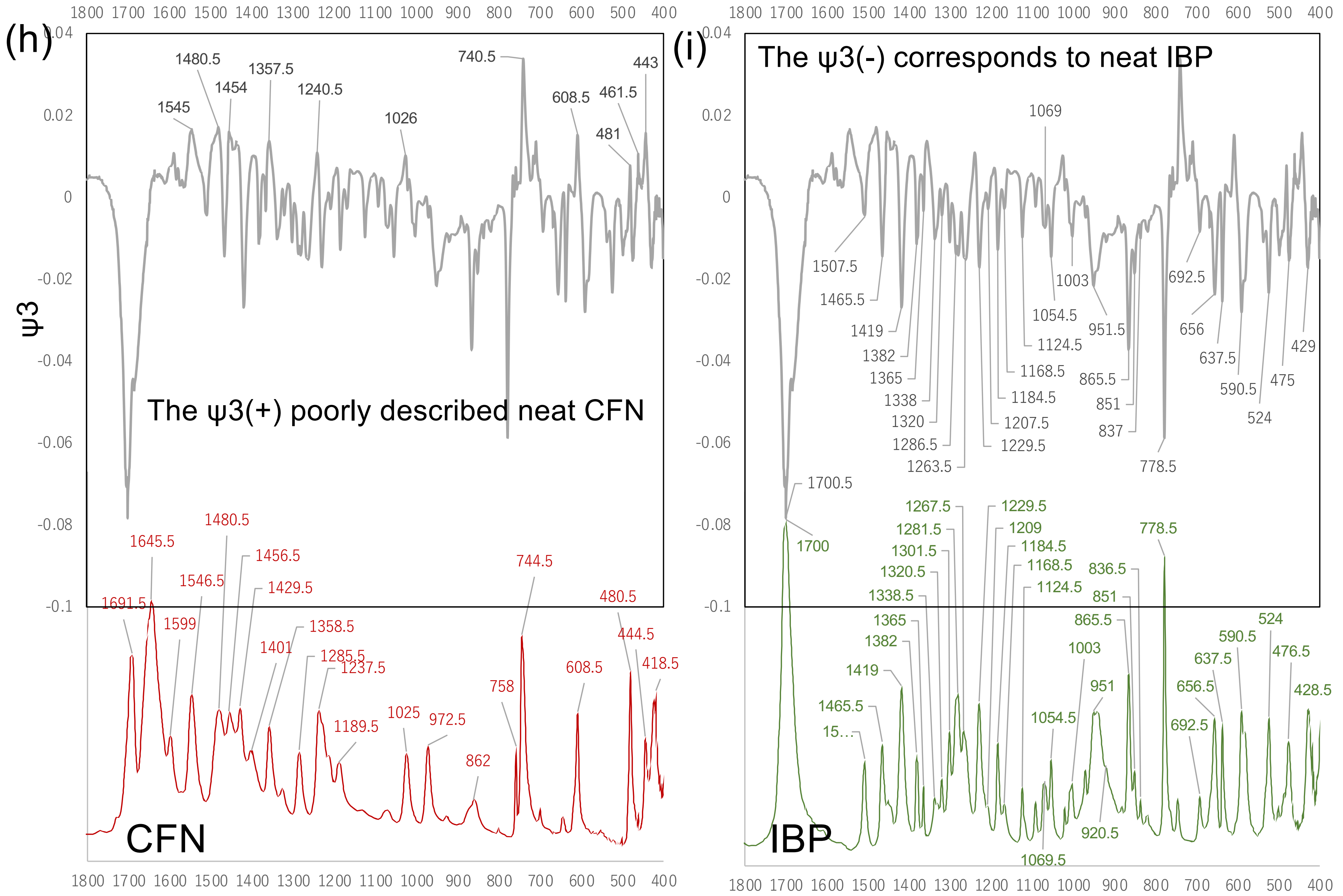

(h)
The ψ3(+) poorly described neat CFN
CFN
(i)
The ψ3(-) corresponds to neat IBP
IBP
ψ3

Figure S8

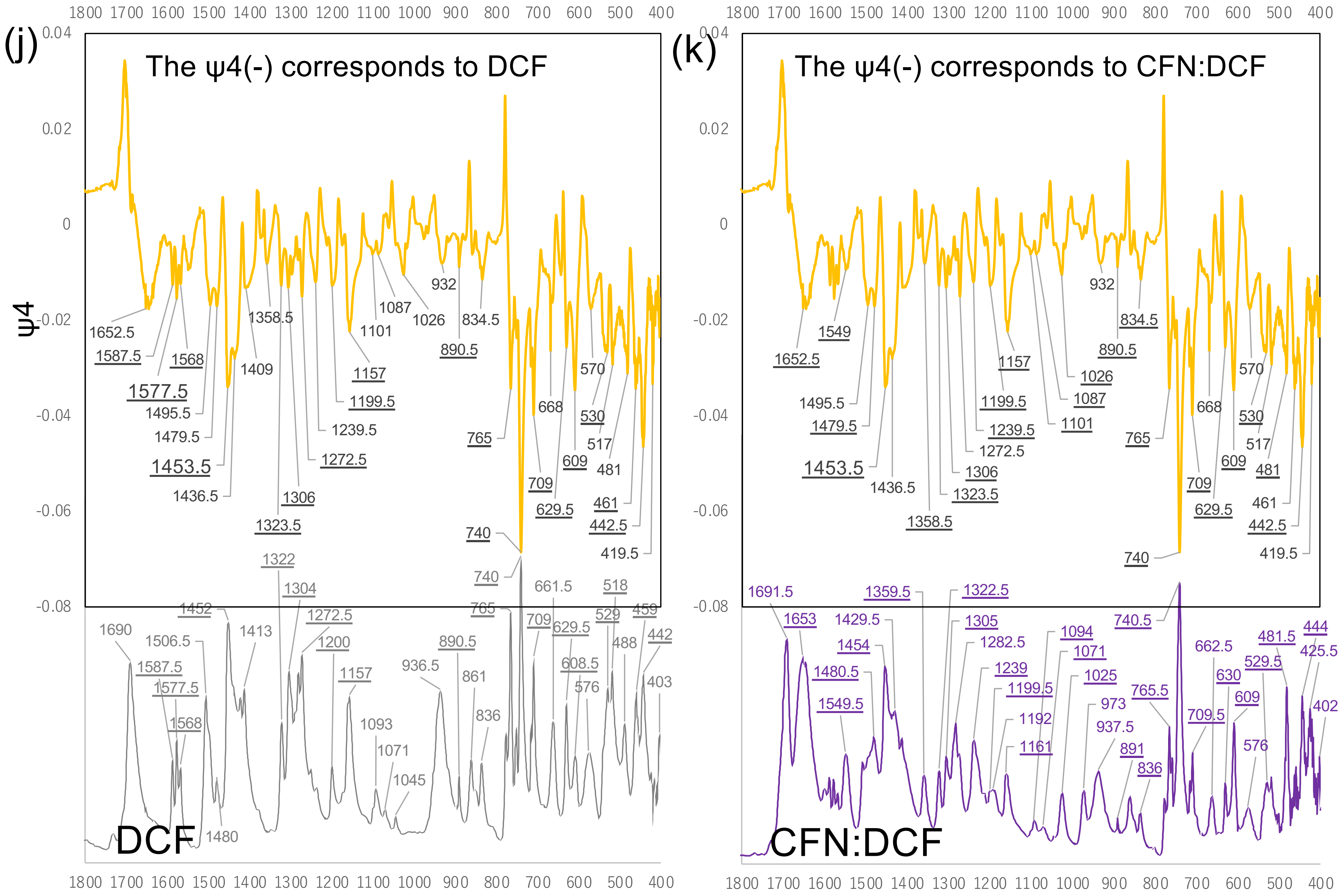
(j)
The ψ4(-) corresponds to DCF
(k)
The ψ4(-) corresponds to CFN:DCF
ψ4
DCF
CFN:DCF

Figure S8

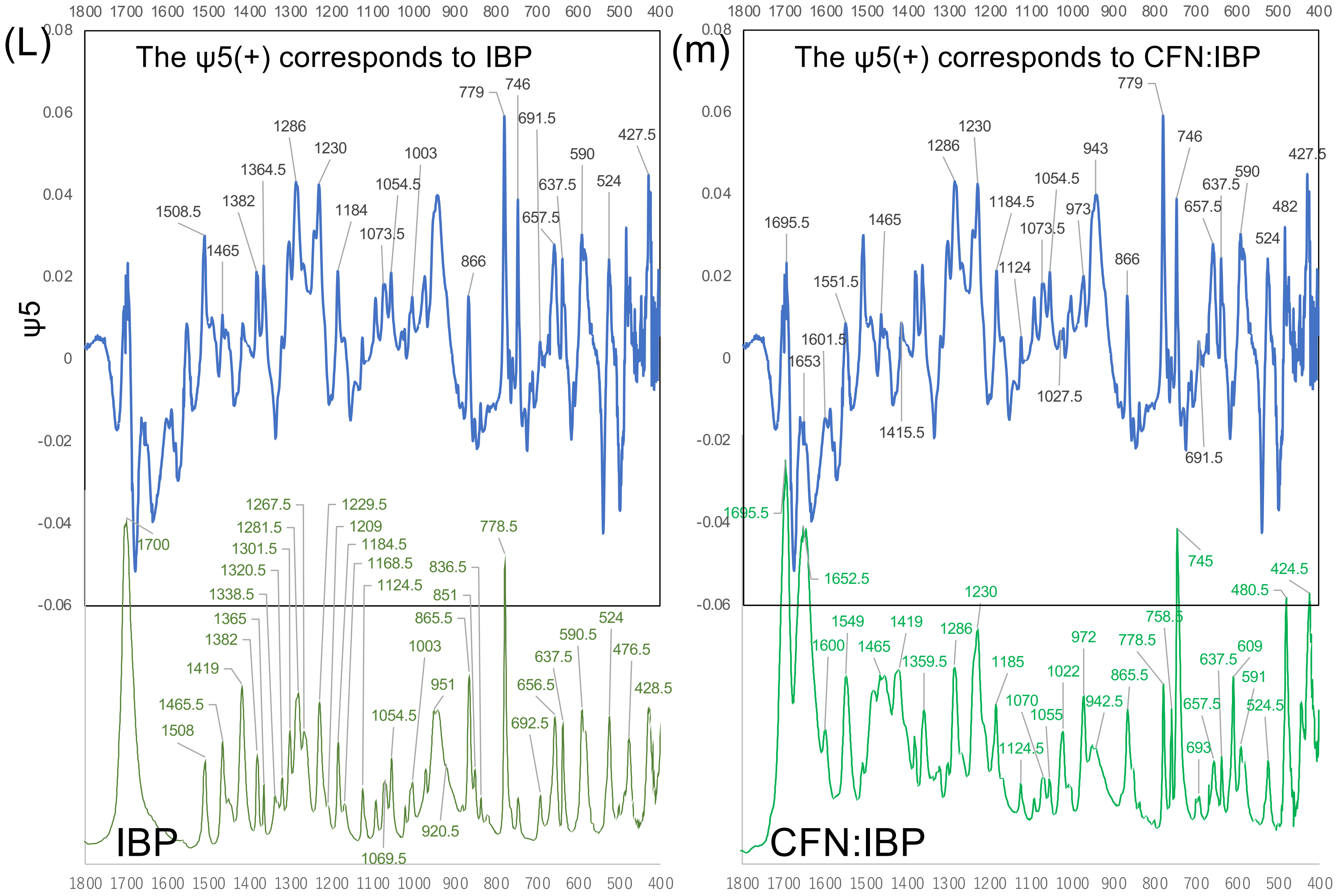
(L)
The ψ5(+) corresponds to IBP
ψ5
IBP
(m)
The ψ5(+) corresponds to CFN:IBP
CFN:IBP
1800 1700 1600 1500 1400 1300 1200 1100 1000 900 800 700 600 500 400
0.08
0.06
0.04
0.02
0
-0.02
-0.04
-0.06
779
746
691.5
1286
1230
1003
427.5
590
1364.5
1054.5
637.5
524
1382
1184
657.5
1508.5
1073.5
1465
866
1700
1267.5
1229.5
1281.5
1209
1301.5
1184.5
778.5
1320.5
1168.5
836.5
1124.5
1338.5
851
1365
865.5
1382
1003
590.5
476.5
1419
637.5
951
428.5
656.5
1465.5
1054.5
692.5
1508
920.5
1069.5
943
482
973
1695.5
1551.5
1124
1601.5
1653
1027.5
1415.5
691.5
745
1652.5
424.5
480.5
1549
1419
758.5
1600
1465
972
778.5
609
1359.5
1185
1022
591
865.5
1070
942.5
657.5
1055
524.5
1124.5
693

Figure S8

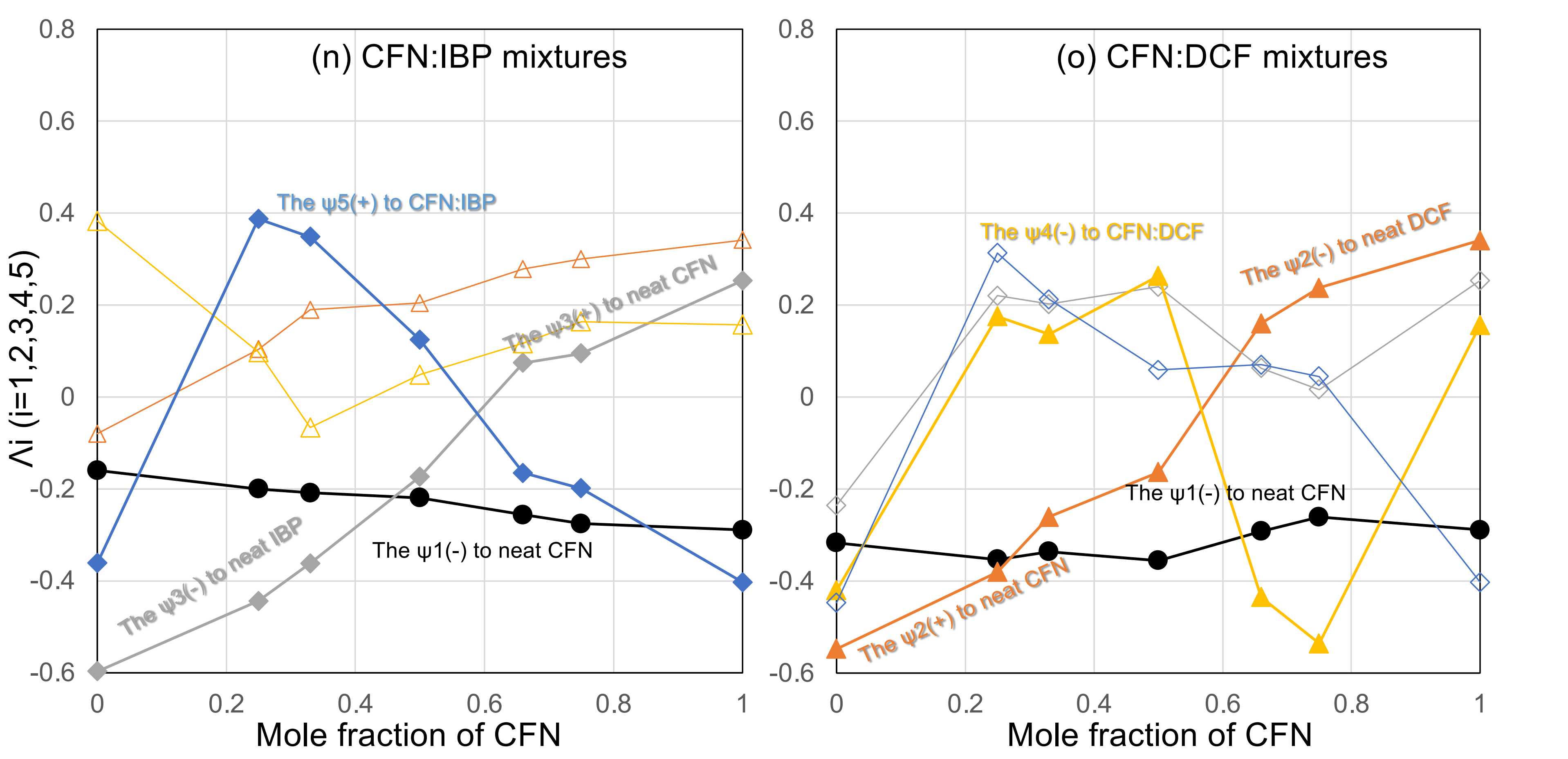

Figure S8 (n and o). The components of the first to fifth singular vectors as a function of mole fraction of CFN. The λ1, λ2, λ3, λ4, and λ5 were represented by closed black circles, orange triangles, gray diamonds, yellow triangles, and blue diamonds. For λ2, λ3, λ4, and λ5, closed and open symbols corresponded to the composition with the basis functions correlated to which the IBP or DCF mixtures, respectively.

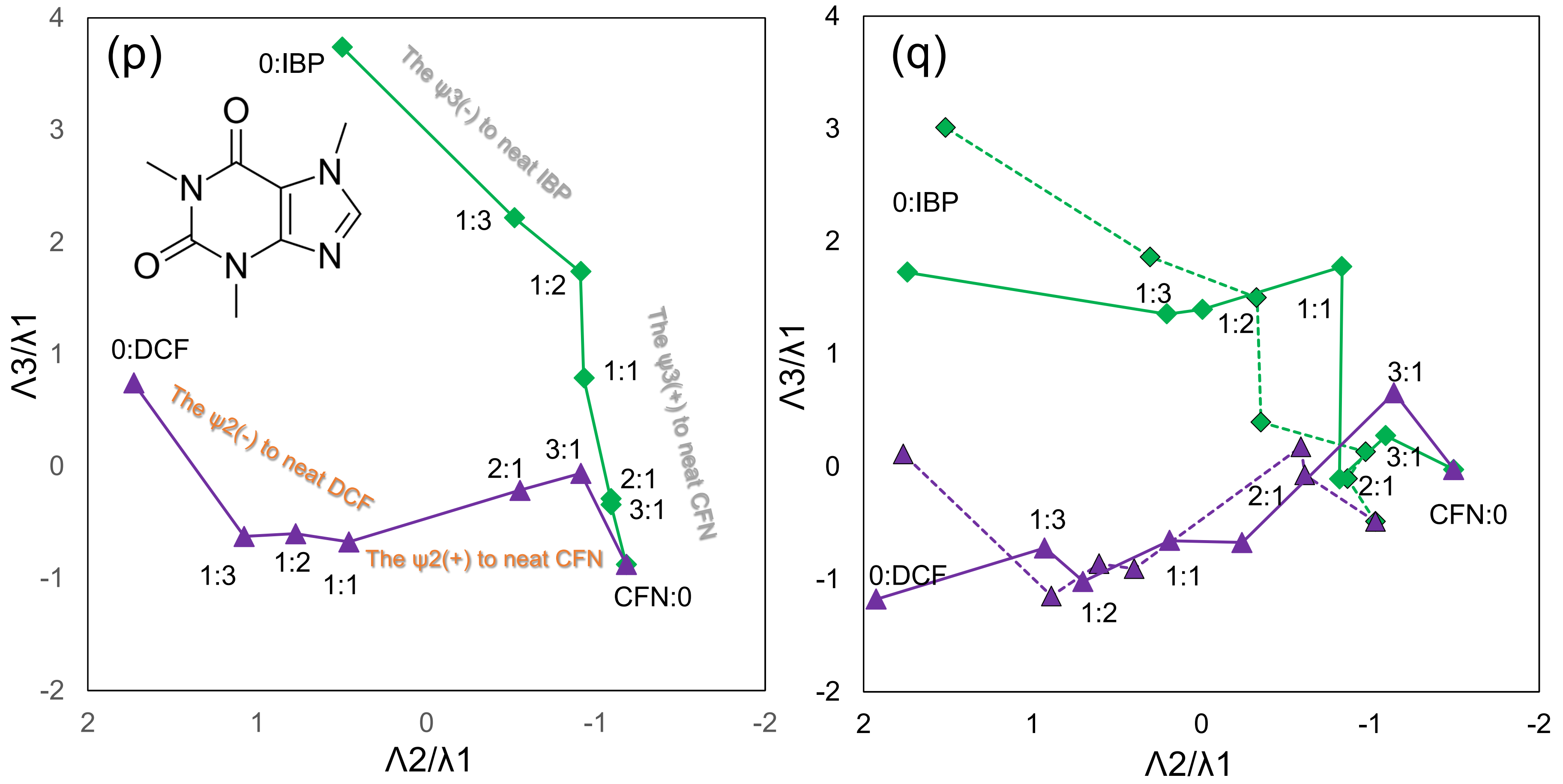

Figure S8 (p and q). The trajectories of the CFN/IBP (green diamonds) and CFN/DCF (violet triangles) mixtures. They shared the neat CFN's vector (CFN:0). The molar ratios were represented along the corresponding plots. The trajectory of the CFN/DCF mixtures traced along the verical (λ2/λ1)-axis (DCF owning dominance in the SVD analysis), whereas that of the CFN/IBP mixtures pursued along the perpendicular (λ2/λ1)-axis. The S13(r) and S13(s) show the trajectories of the average spectra and the individually measured spectra, respectively.

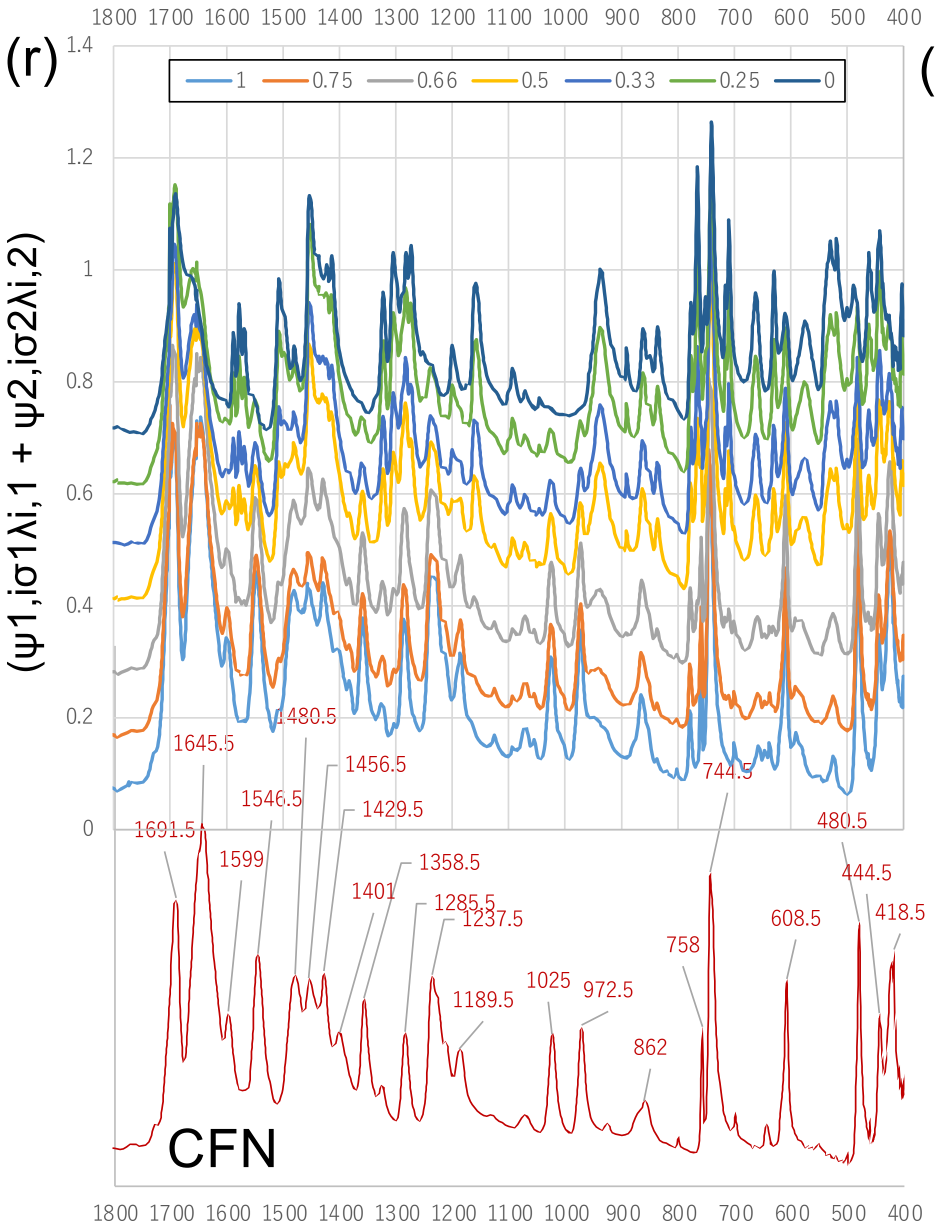
(r)
(ψ1,iσ1λi,1 + ψ2,iσ2λi,2)
1 0.75 0.66 0.5 0.33 0.25 0
1691.5
1645.5
1599
1546.5
1480.5
1456.5
1429.5
1401
1358.5
1285.5
1237.5
1189.5
1025
972.5
862
758
744.5
608.5
480.5
444.5
418.5
CFN

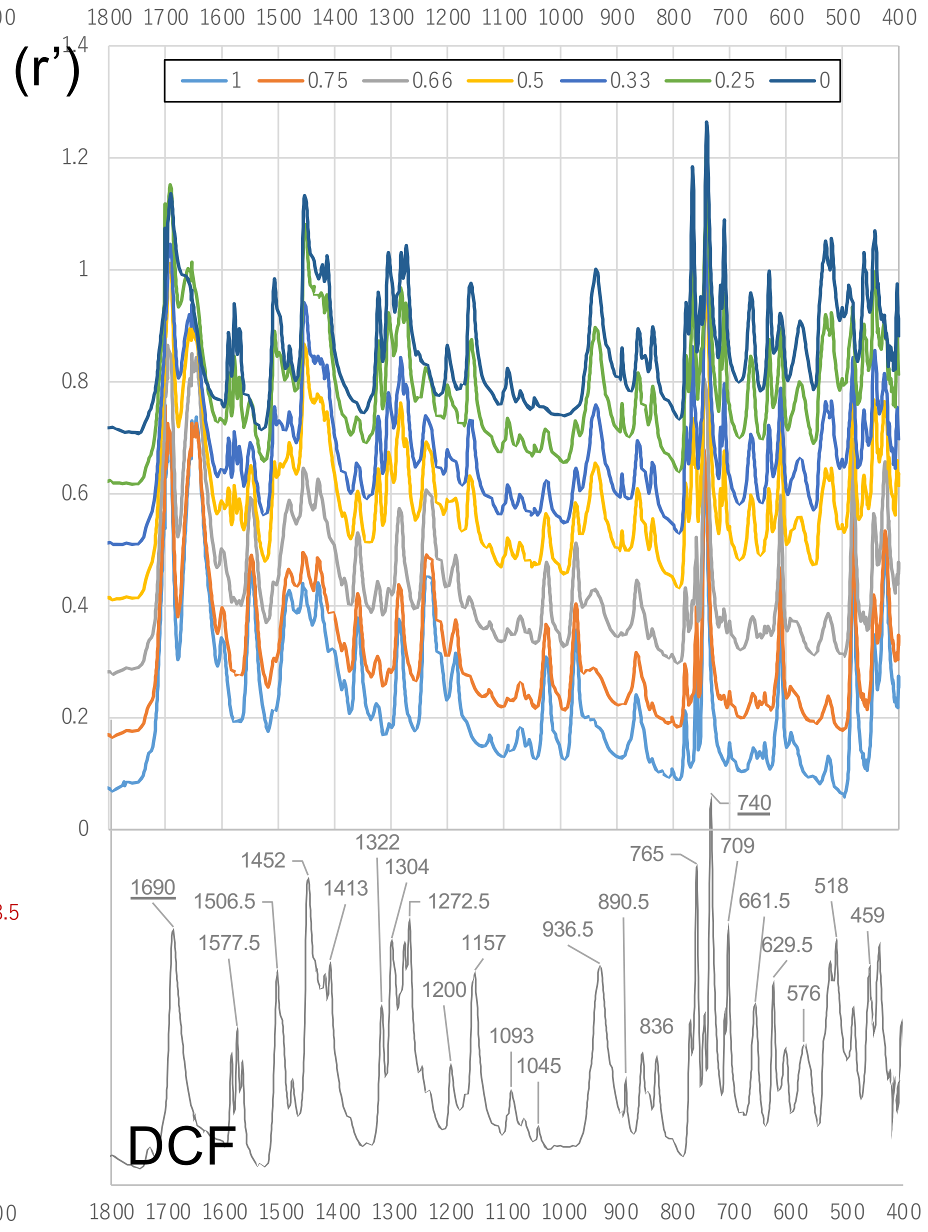
(r')
1 0.75 0.66 0.5 0.33 0.25 0
1690
1577.5
1506.5
1452
1413
1322
1304
1272.5
1200
1157
1093
1045
936.5
890.5
836
765
740
709
661.5
629.5
576
518
459
DCF

Figure S8

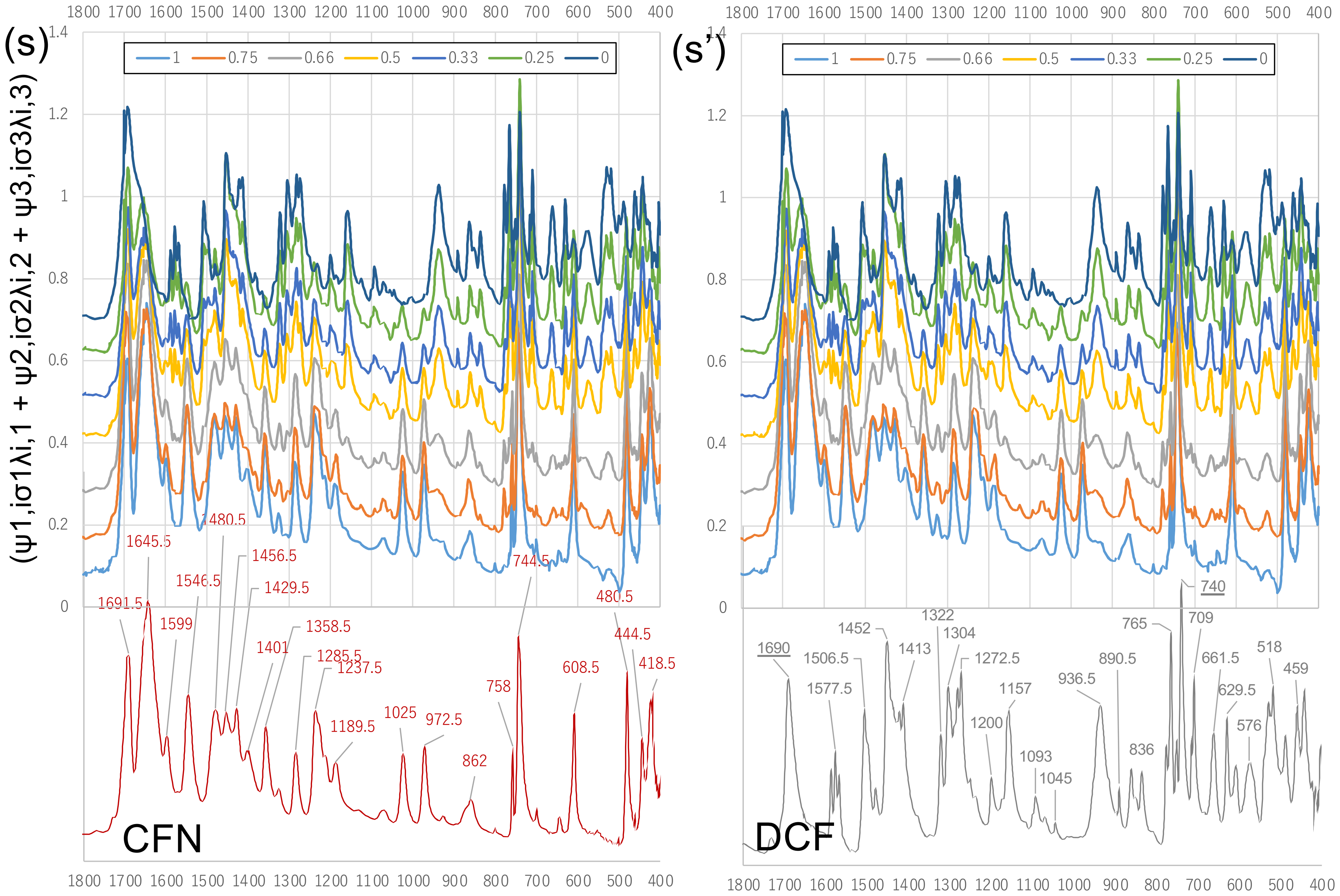
(s)
(s')
(ψ1,iσ1λi,1 + ψ2,iσ2λi,2 + ψ3,iσ3λi,3)
1
0.75
0.66
0.5
0.33
0.25
0
1691.5
1645.5
1599
1546.5
1480.5
1456.5
1429.5
1401
1358.5
1285.5
1237.5
1189.5
1025
972.5
862
758
744.5
608.5
480.5
444.5
418.5
CFN
1690
1577.5
1506.5
1452
1413
1322
1304
1272.5
1200
1157
1093
1045
936.5
890.5
836
765
740
709
661.5
629.5
576
518
459
DCF

Figure S8

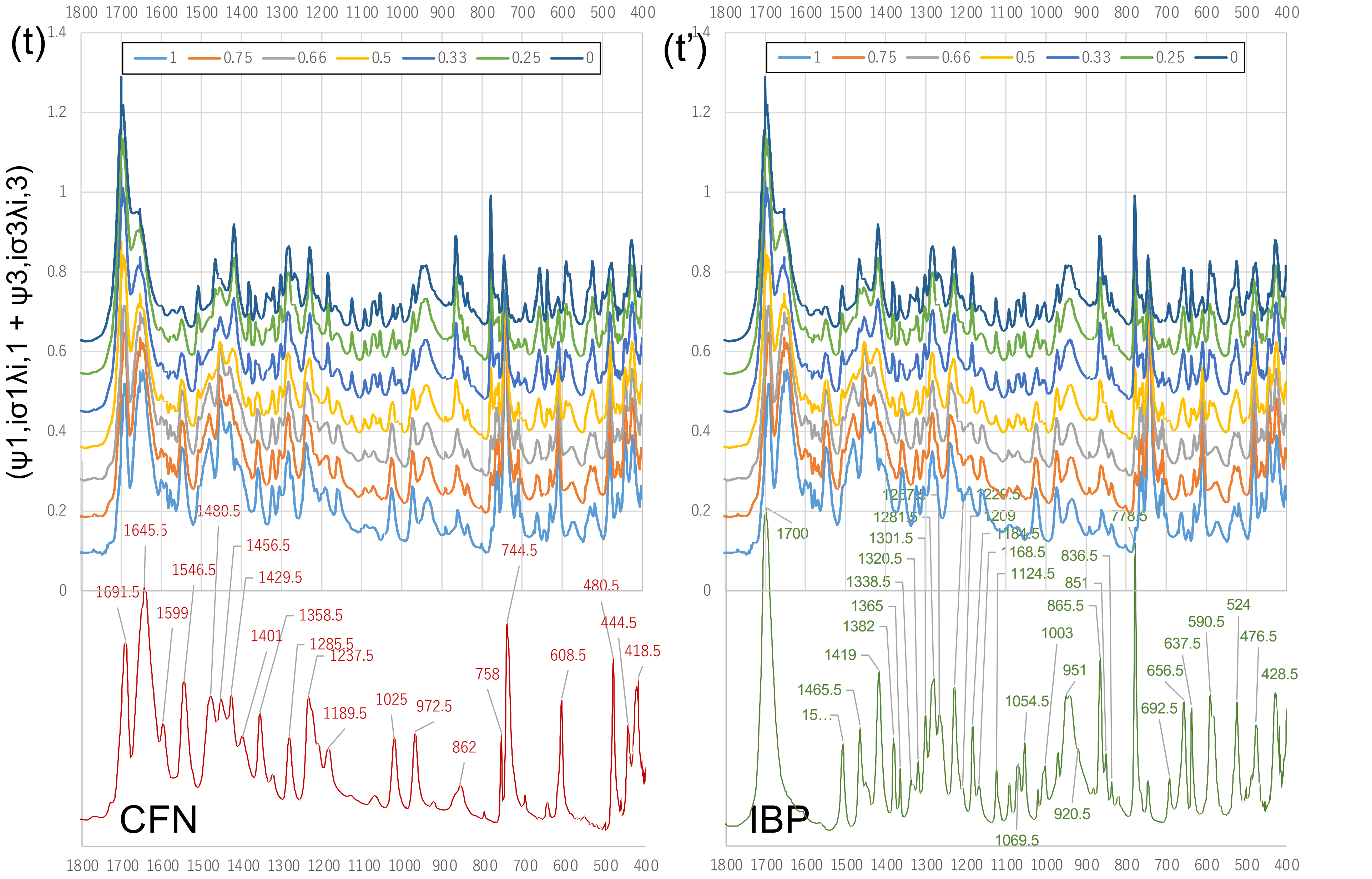

(t)
(t')
(ψ1,iσ1λi,1 + ψ3,iσ3λi,3)
1
0.75
0.66
0.5
0.33
0.25
0
CFN
IBP
1691.5
1645.5
1599
1546.5
1480.5
1456.5
1429.5
1401
1358.5
1285.5
1237.5
1189.5
1025
972.5
862
758
744.5
608.5
480.5
444.5
418.5
1700
1257.5
1229.5
1281.5
1209
1301.5
1184.5
1320.5
1168.5
1338.5
1124.5
1365
1382
1419
1465.5
15…
836.5
851
865.5
1003
951
1054.5
1069.5
920.5
778.5
692.5
656.5
637.5
590.5
524
476.5
428.5

Figure S8

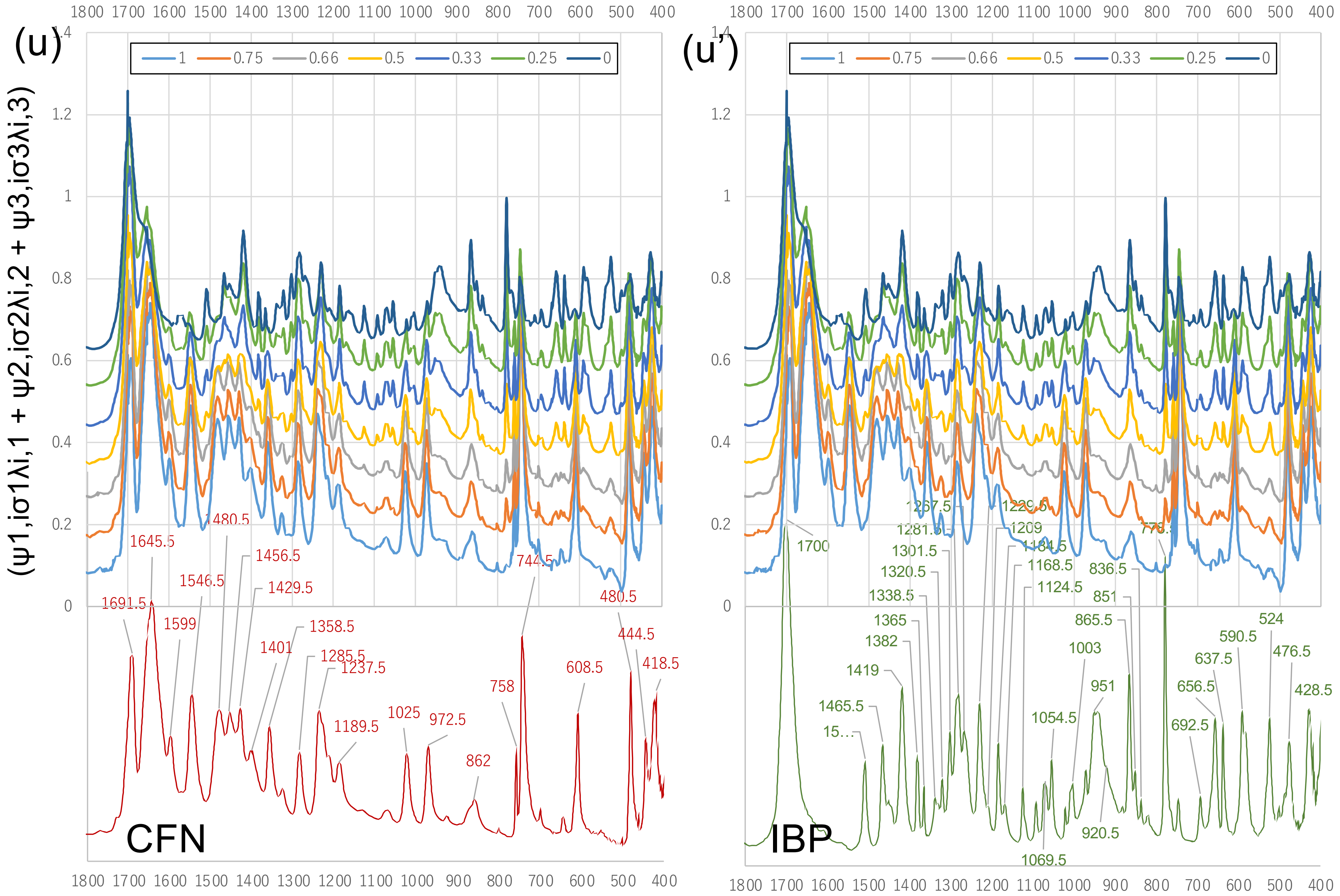
(u)
(u')
(ψ1,iσ1λi,1 + ψ2,iσ2λi,2 + ψ3,iσ3λi,3)
1
0.75
0.66
0.5
0.33
0.25
0
CFN
IBP
1691.5
1645.5
1599
1546.5
1480.5
1456.5
1429.5
1401
1358.5
1285.5
1237.5
1189.5
1025
972.5
862
758
744.5
608.5
480.5
444.5
418.5
1700
1465.5
1419
1382
1365
1338.5
1320.5
1301.5
1281.5
1267.5
1229.5
1209
1184.5
1168.5
1124.5
1069.5
1054.5
1003
951
920.5
865.5
851
836.5
692.5
656.5
637.5
590.5
524
476.5
428.5

Figure S8

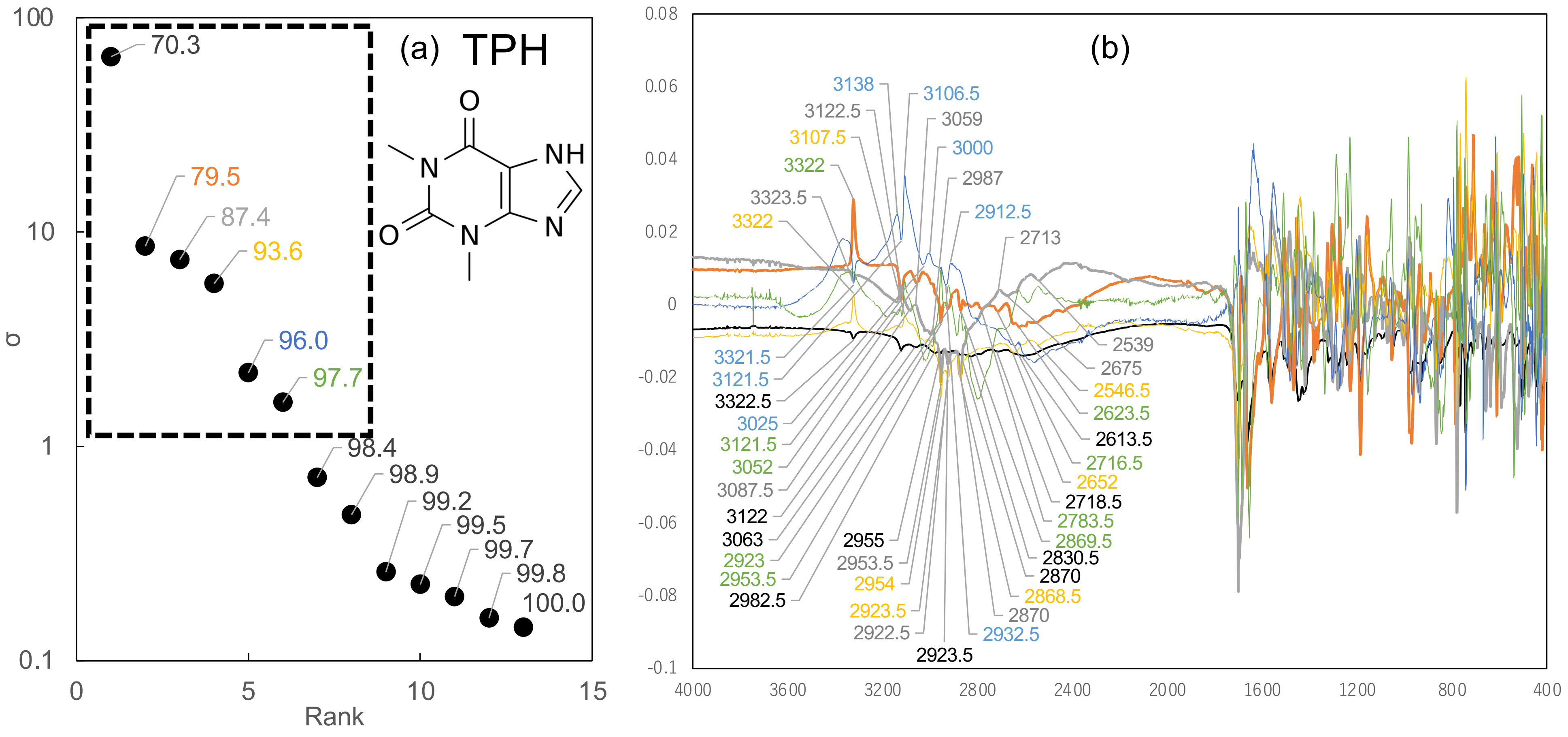

Figure S9. SVD computation for FTIR spectra of TPH/IBP and TPH/DCF mixtures provided singular values σi (a) and Basis spectra ψi (b). Cumulative proportions of σi were appended beside the σ plots, indicating that higher five σ's which were satisfied 97.7 percent of the variance. The basis spectra decomposed by the SVD computation corresponded to the components ψ1 (black), ψ2 (orange), ψ3 (gray), ψ4 (yellow), and ψ5 (blue). Details of ψ's assignments to the observed spectra in the wavenumber range 1800-400 cm^{-1} are followed (e-o).

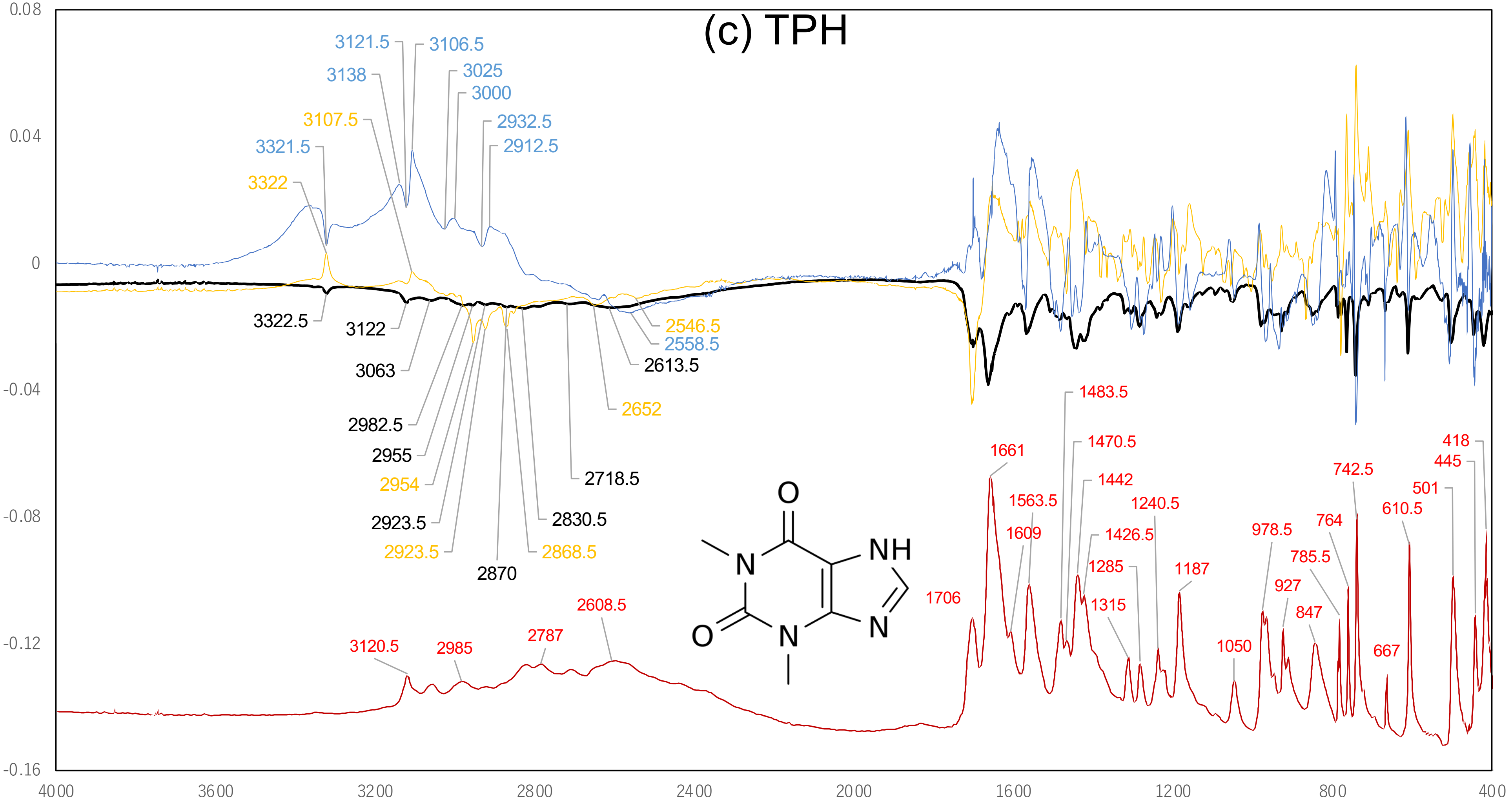

Figure S9. The basis spectra decomposed by the SVD computation corresponded to the components at the rank of 1 (black), 4 (yellow), and 5 (blue).

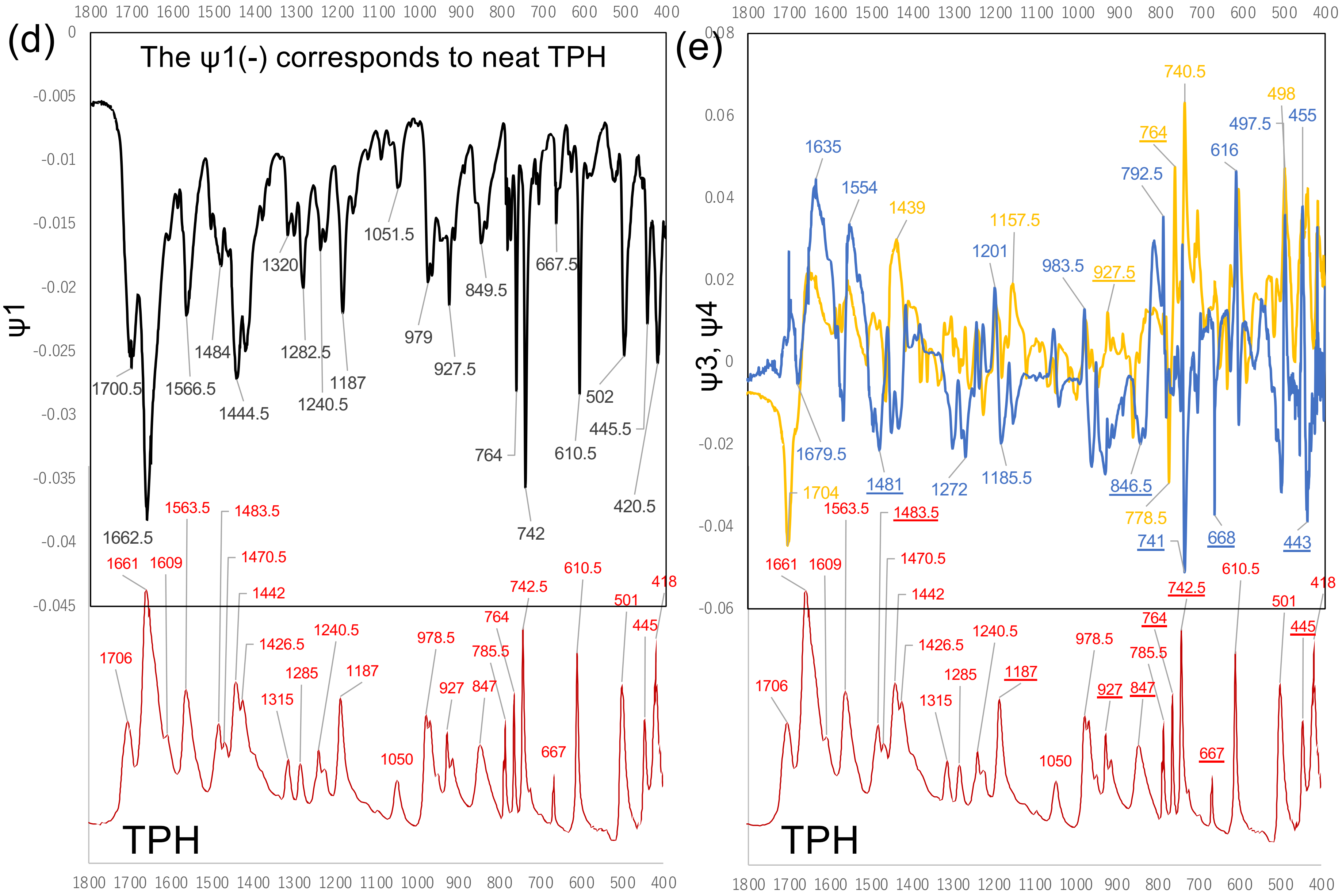

(d)
The ψ1(-) corresponds to neat TPH
ψ1
1800 1700 1600 1500 1400 1300 1200 1100 1000 900 800 700 600 500 400
0
-0.005
-0.01
-0.015
-0.02
-0.025
-0.03
-0.035
-0.04
-0.045
1700.5
1662.5
1566.5
1484
1444.5
1320
1282.5
1240.5
1187
1051.5
979
927.5
849.5
764
742
667.5
610.5
502
445.5
420.5
1661
1609
1563.5
1483.5
1470.5
1442
1426.5
1706
1285
1315
1240.5
1187
1050
978.5
927
847
785.5
764
742.5
667
610.5
501
445
418
TPH
(e)
ψ3, ψ4
0.08
0.06
0.04
0.02
0
-0.02
-0.04
-0.06
1635
1554
1439
1157.5
1201
983.5
927.5
764
740.5
792.5
616
498
497.5
455
1679.5
1704
1481
1272
1185.5
846.5
778.5
741
668
443
1661
1609
1563.5
1483.5
1470.5
1442
1426.5
1706
1285
1315
1240.5
1187
1050
978.5
927
847
785.5
764
742.5
667
610.5
501
445
418
TPH

Figure S9

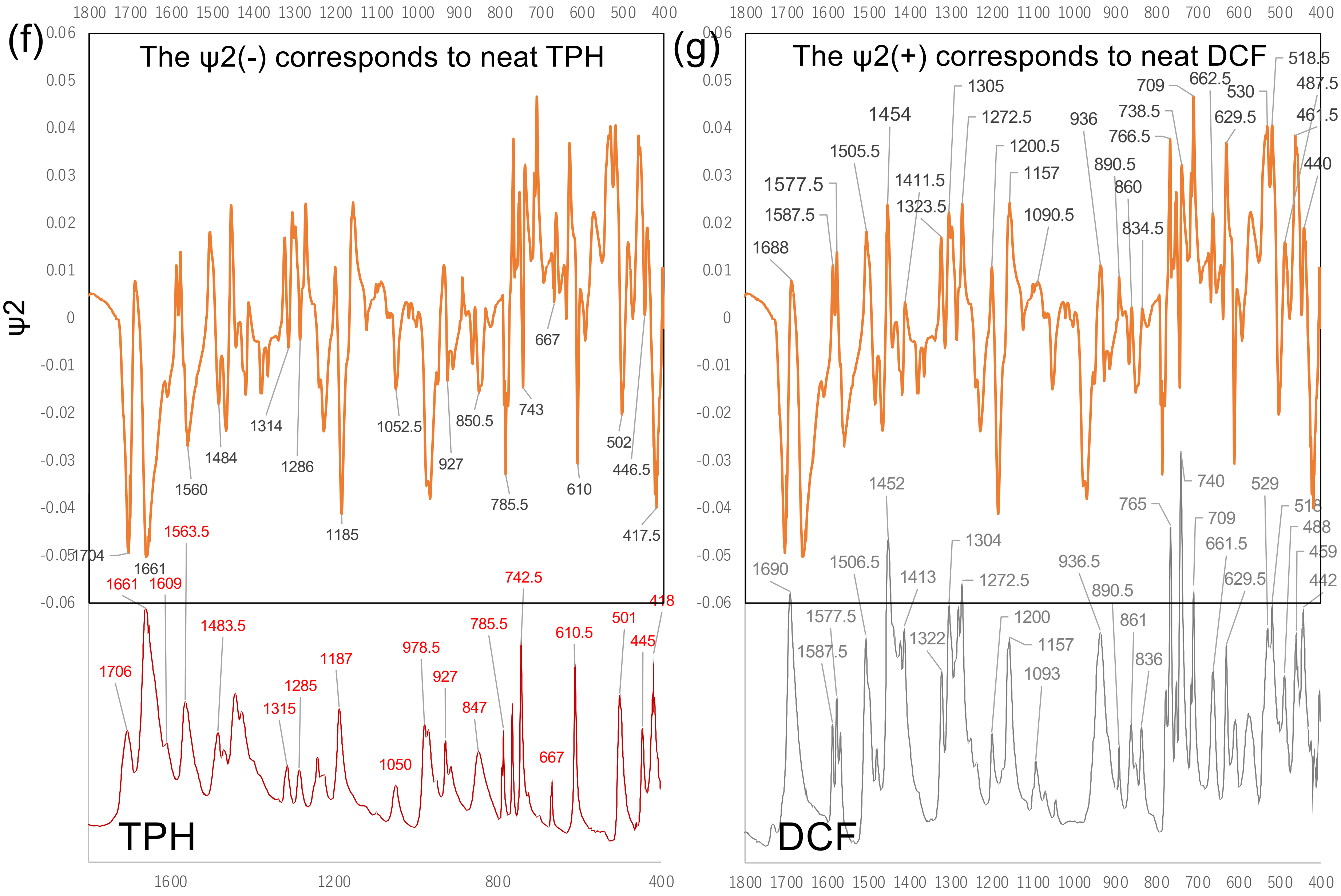

(f)
The ψ2(-) corresponds to neat TPH
ψ2
TPH
(g)
The ψ2(+) corresponds to neat DCF
DCF

Figure S9

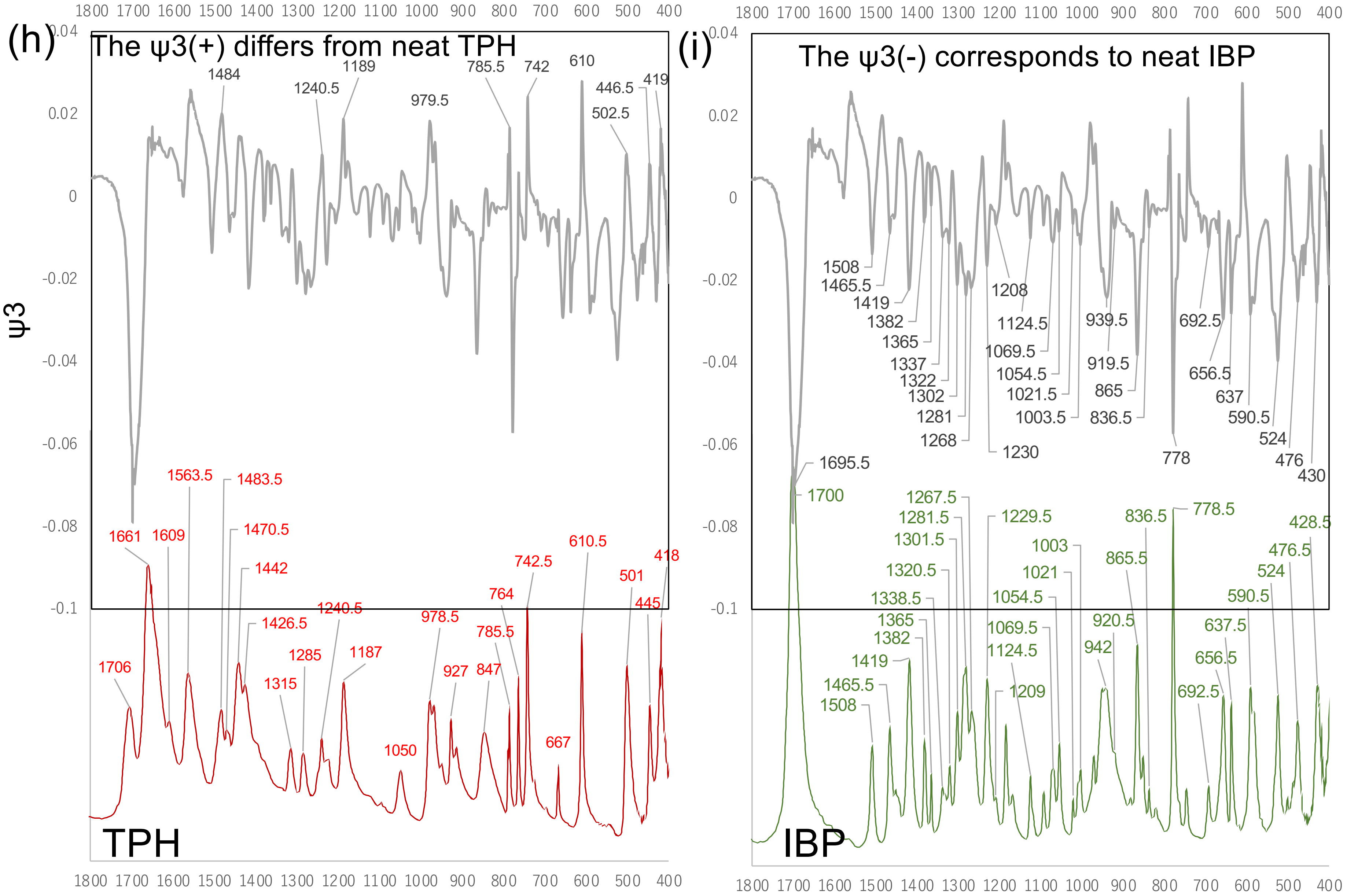
(h)
The ψ3(+) differs from neat TPH
(i)
The ψ3(-) corresponds to neat IBP
ψ3
0.04
0.02
0
-0.02
-0.04
-0.06
-0.08
-0.1
1800 1700 1600 1500 1400 1300 1200 1100 1000 900 800 700 600 500 400
1484
1240.5
1189
979.5
785.5
742
610
446.5
419
502.5
1563.5
1483.5
1661
1609
1470.5
1442
1426.5
1240.5
1706
1315
1285
1187
1050
978.5
927
847
785.5
764
742.5
667
610.5
501
445
418
TPH
1508
1465.5
1419
1382
1365
1337
1322
1302
1281
1268
1208
1124.5
1069.5
1054.5
1021.5
1003.5
1230
939.5
919.5
865
836.5
778
692.5
656.5
637
590.5
524
476
430
1695.5
1700
1267.5
1281.5
1301.5
1320.5
1338.5
1365
1382
1419
1465.5
1508
1229.5
1003
1021
1054.5
1069.5
1124.5
1209
836.5
778.5
865.5
920.5
942
637.5
656.5
692.5
590.5
524
476.5
428.5
IBP

Figure S9

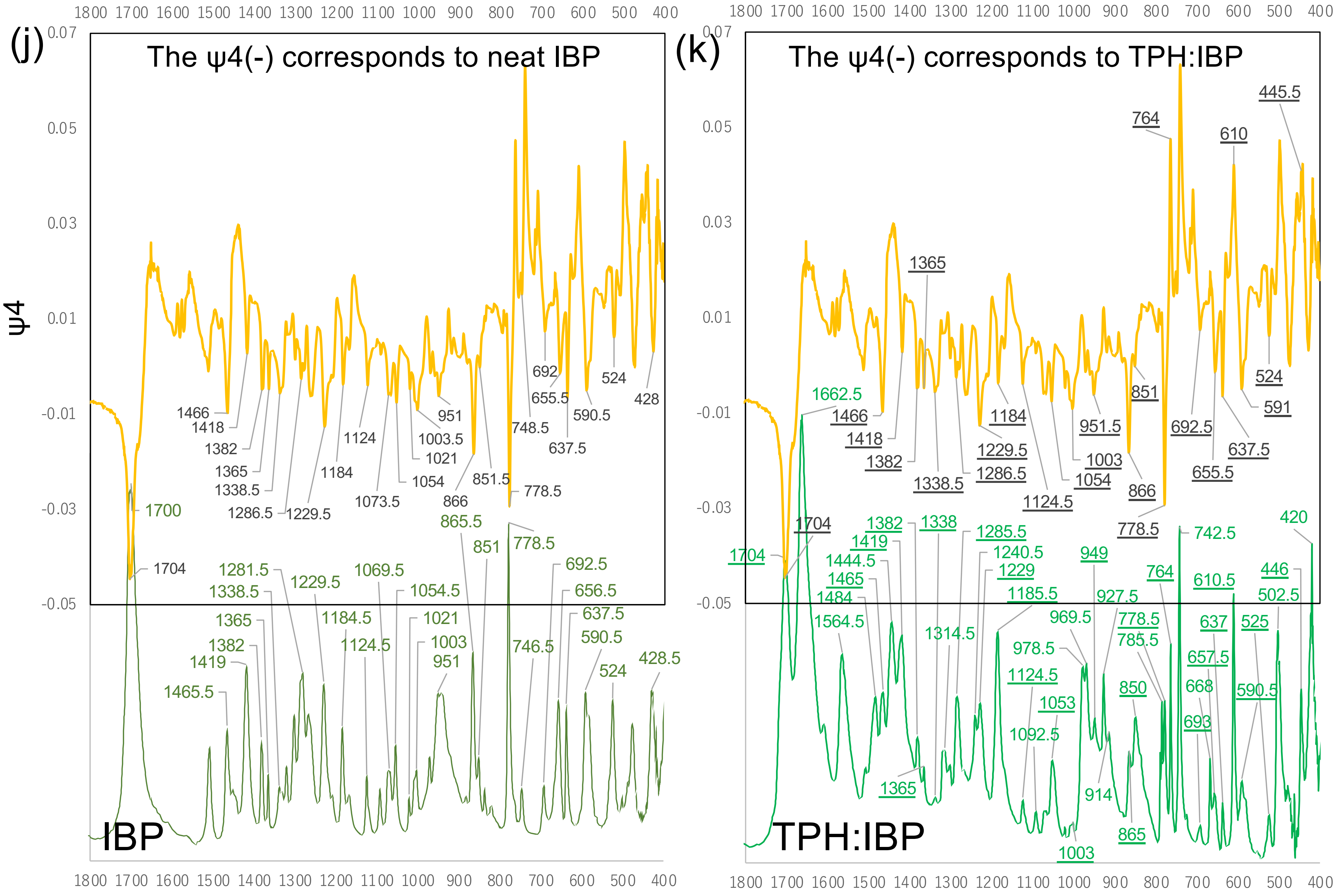

(j)
The ψ4(-) corresponds to neat IBP
(k)
The ψ4(-) corresponds to TPH:IBP
ψ4
IBP
TPH:IBP

Figure S9

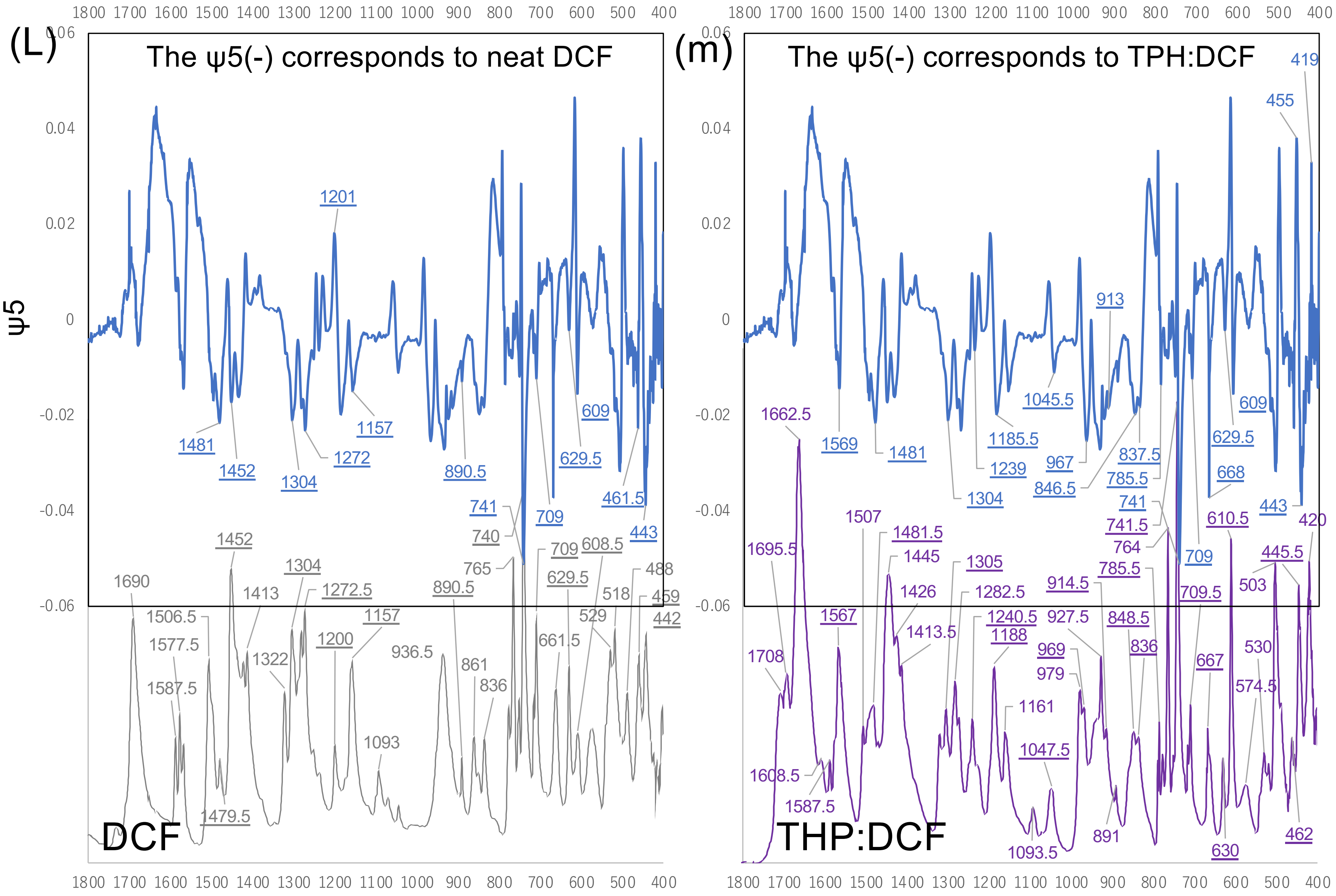
(L)
The ψ5(-) corresponds to neat DCF
(m)
The ψ5(-) corresponds to TPH:DCF
ψ5
DCF
THP:DCF
1800 1700 1600 1500 1400 1300 1200 1100 1000 900 800 700 600 500 400
0.06
0.04
0.02
0
-0.02
-0.04
-0.06
1201
1481
1452
1304
1272
1157
890.5
741
709
629.5
609
461.5
443
1690
1506.5
1577.5
1587.5
1479.5
1413
1322
1272.5
1200
1157
1093
936.5
890.5
861
836
765
740
709
661.5
629.5
608.5
529
518
488
459
442
1662.5
1569
1481
1304
1239
1185.5
1045.5
967
913
846.5
837.5
785.5
741
741.5
764
668
629.5
610.5
709
455
419
420
1695.5
1708
1608.5
1587.5
1567
1507
1481.5
1445
1426
1413.5
1305
1282.5
1240.5
1188
1161
1093.5
1047.5
969
979
927.5
914.5
891
848.5
836
785.5
709.5
667
630
574.5
530
503
445.5
462

Figure S9

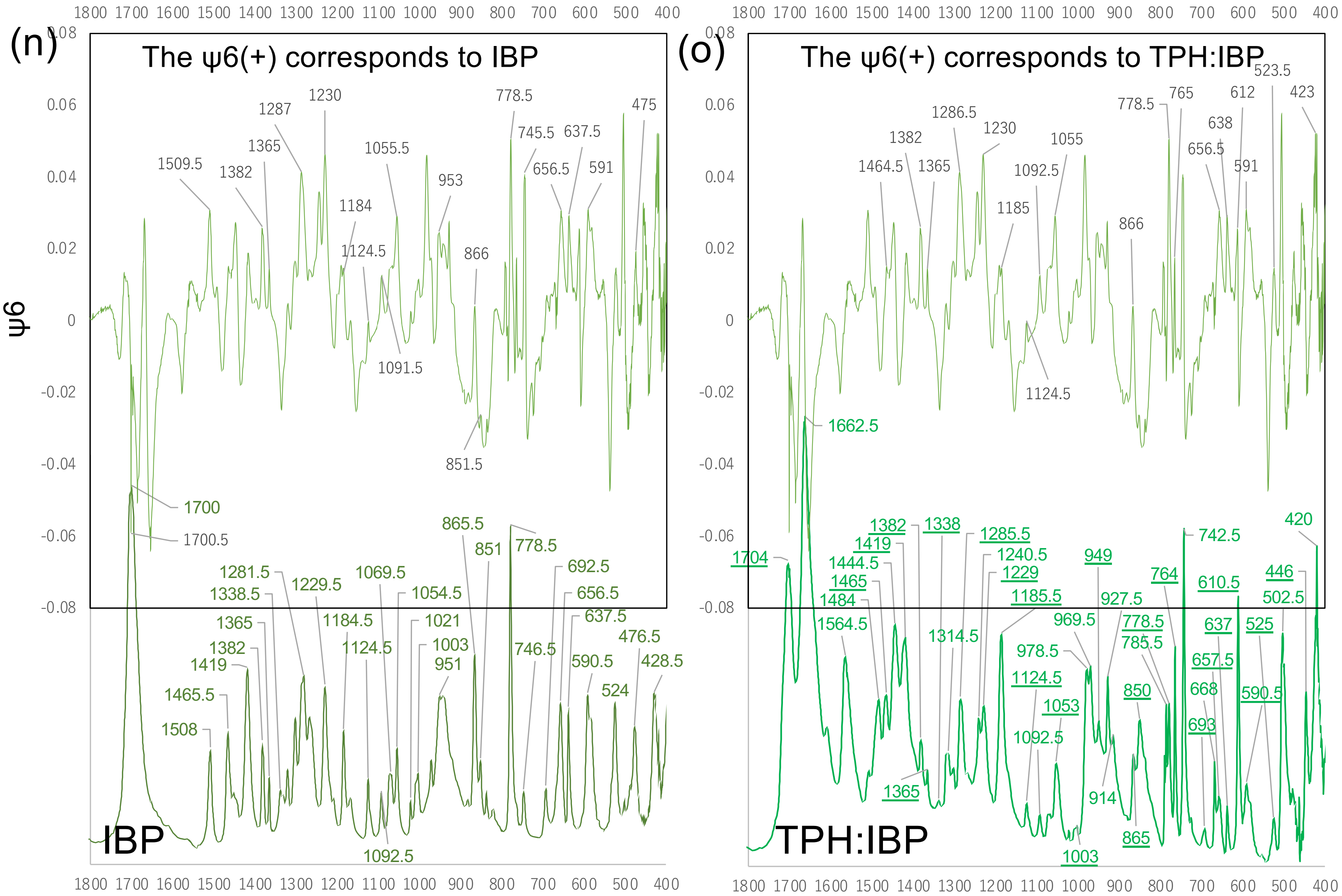
(n)
The ψ6(+) corresponds to IBP
(o)
The ψ6(+) corresponds to TPH:IBP
ψ6
1800 1700 1600 1500 1400 1300 1200 1100 1000 900 800 700 600 500 400
0.08
0.06
0.04
0.02
0
-0.02
-0.04
-0.06
-0.08
IBP
TPH:IBP
1509.5
1382
1365
1287
1230
1184
1124.5
1055.5
1091.5
953
866
851.5
778.5
745.5
656.5
637.5
591
475
1700
1700.5
1508
1465.5
1419
1382
1365
1338.5
1281.5
1229.5
1184.5
1124.5
1092.5
1069.5
1054.5
1021
1003
951
865.5
851
778.5
746.5
692.5
656.5
637.5
590.5
524
476.5
428.5
1464.5
1382
1365
1286.5
1230
1185
1124.5
1092.5
1055
866
778.5
765
656.5
638
612
591
523.5
423
1662.5
1704
1564.5
1484
1465
1444.5
1419
1382
1365
1338
1314.5
1285.5
1240.5
1229
1185.5
1124.5
1092.5
1053
1003
978.5
969.5
949
927.5
914
865
850
785.5
778.5
764
742.5
693
668
657.5
637
610.5
590.5
525
502.5
446
420

Figure S9

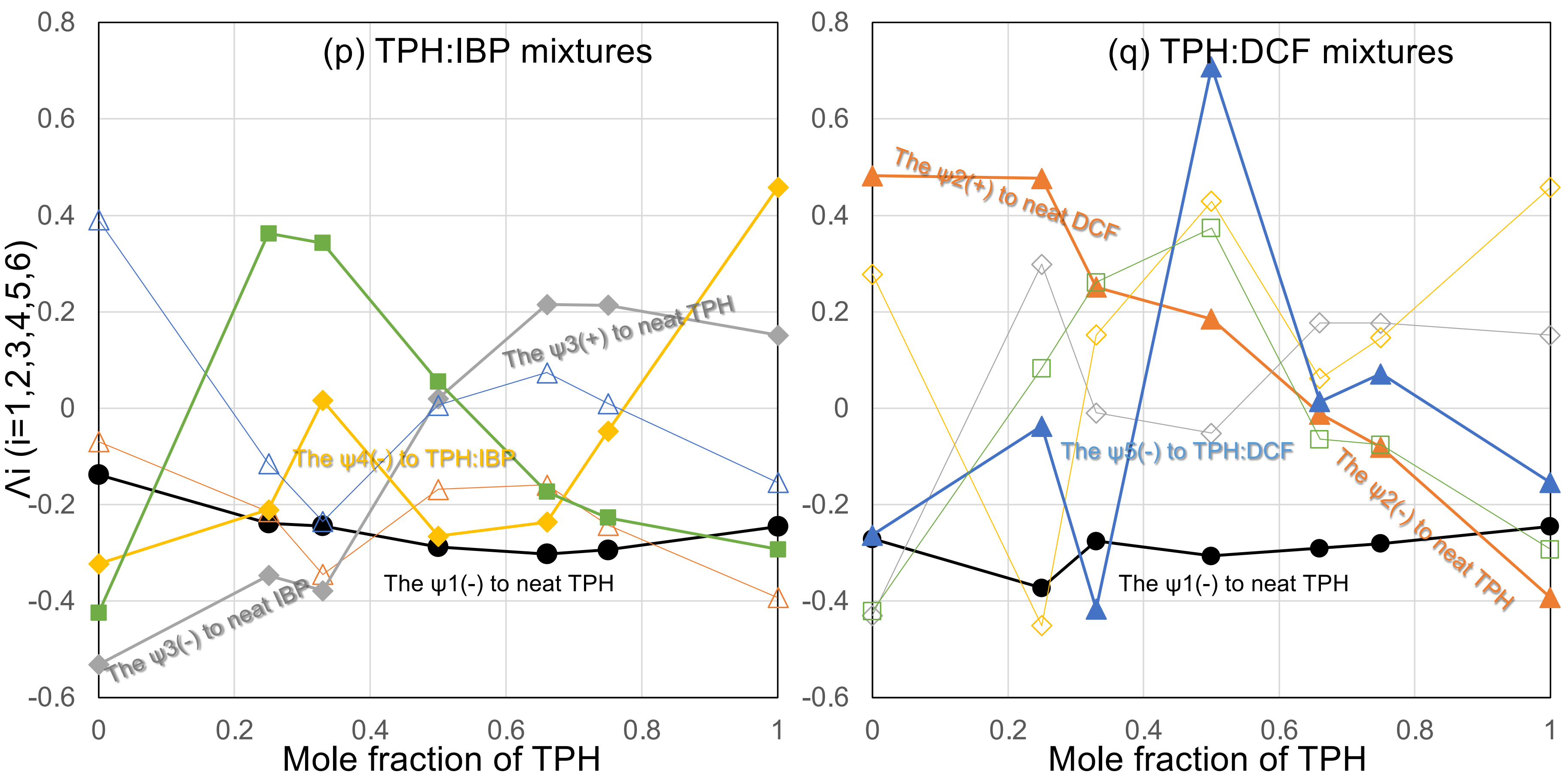

Figure S9 (p and q). The components of the first to fifth singular vectors as a function of mole fraction of TPH. The λ1, λ2, λ3, λ4, λ5, and λ6 were represented by closed black circles, orange triangles, gray diamonds, yellow diamonds, blue triangles, and green squares. For λ2, λ3, λ4, λ5, and λ6, closed and open symbols corresponded to the composition with the basis functions correlated to which the IBP or DCF mixtures, respectively.

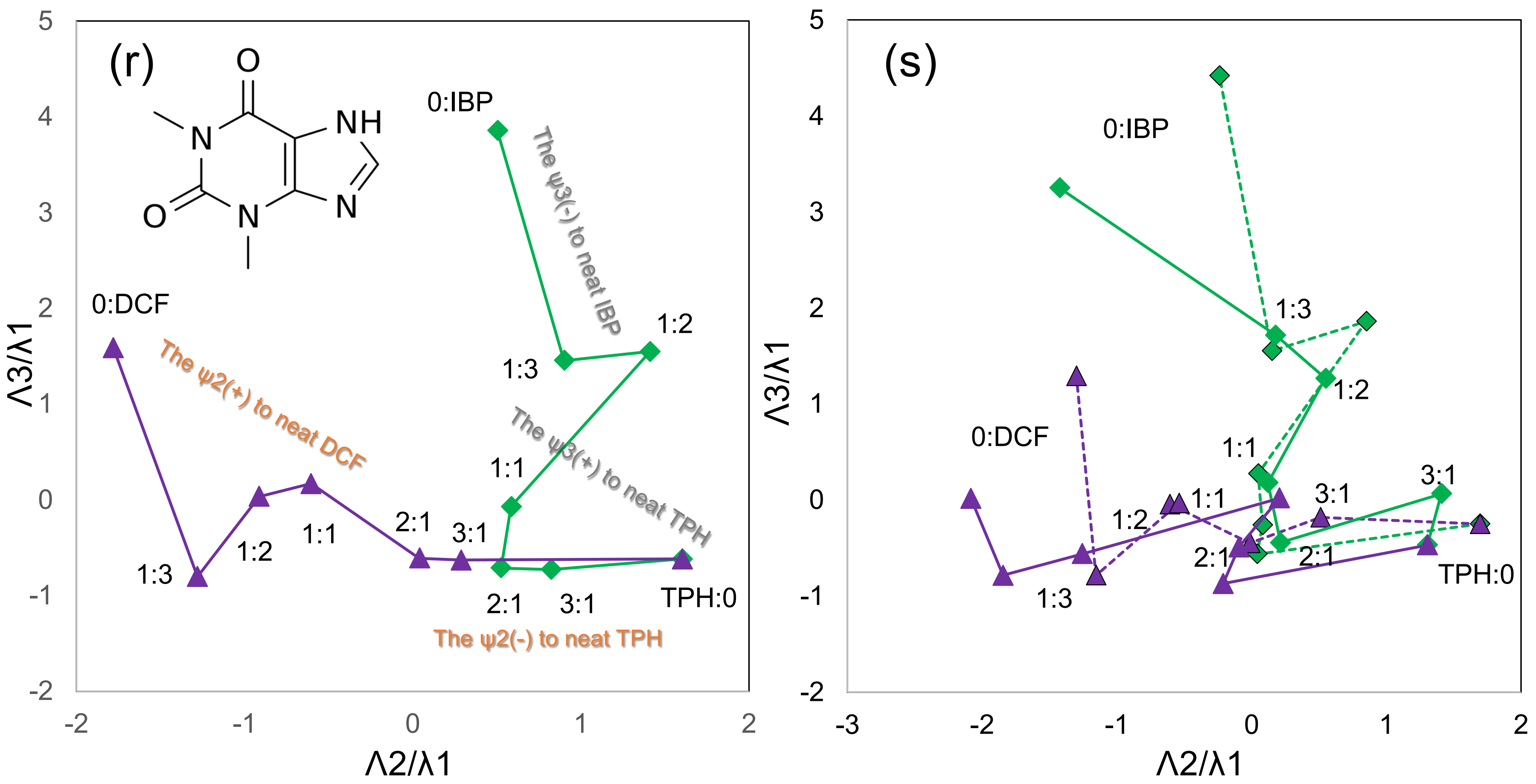

Figure S9 (r and s). The trajectories of the TPH/IBP (green diamonds) and TPH/DCF (violet triangles) mixtures. They shared the neat TPH's vector (TPH:0). The molar ratios were represented along the corresponding plots. The trajectory of the TPH/DCF mixtures traced along the verical (λ2/λ1)-axis (DCF owning dominance in the SVD analysis), whereas that of the TPH/IBP mixtures pursued along the perpendicular (λ2/λ1)-axis, indicating independence of the interactions in these TPH/IBP and TPH/DCF mixtures. The S13(r) and S13(s) show the trajectories of the average spectra and the individually measured spectra, respectively.

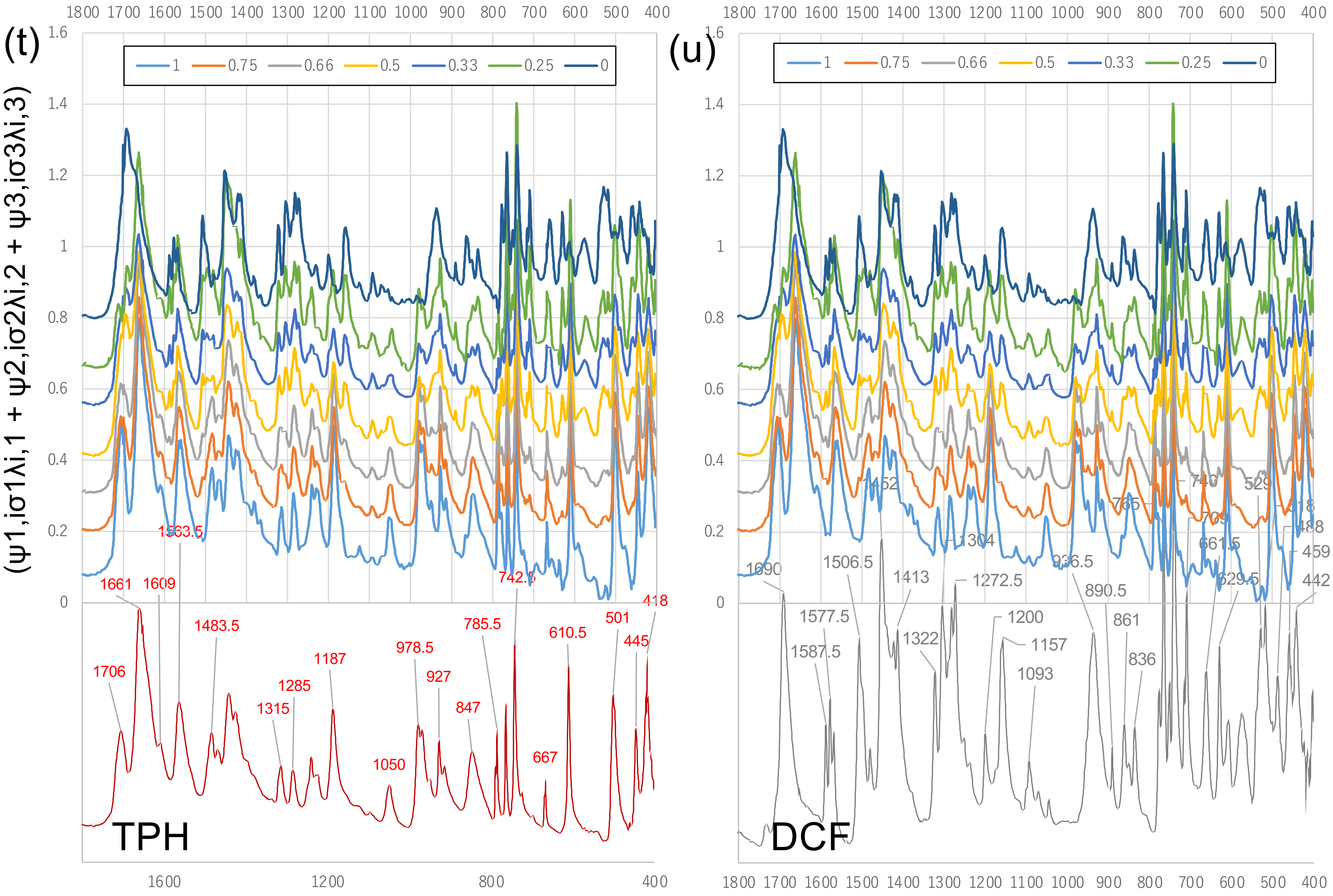
(t)
(u)
(ψ1,iσ1λi,1 + ψ2,iσ2λi,2 + ψ3,iσ3λi,3)
1
0.75
0.66
0.5
0.33
0.25
0
TPH
DCF
1661
1609
1563.5
1706
1483.5
1315
1285
1187
1050
978.5
927
847
785.5
742.5
667
610.5
501
445
418
1690
1577.5
1587.5
1506.5
1452
1413
1322
1304
1272.5
1200
1157
1093
936.5
890.5
861
836
765
740
709
661.5
629.5
529
488
459
442

Figure S9

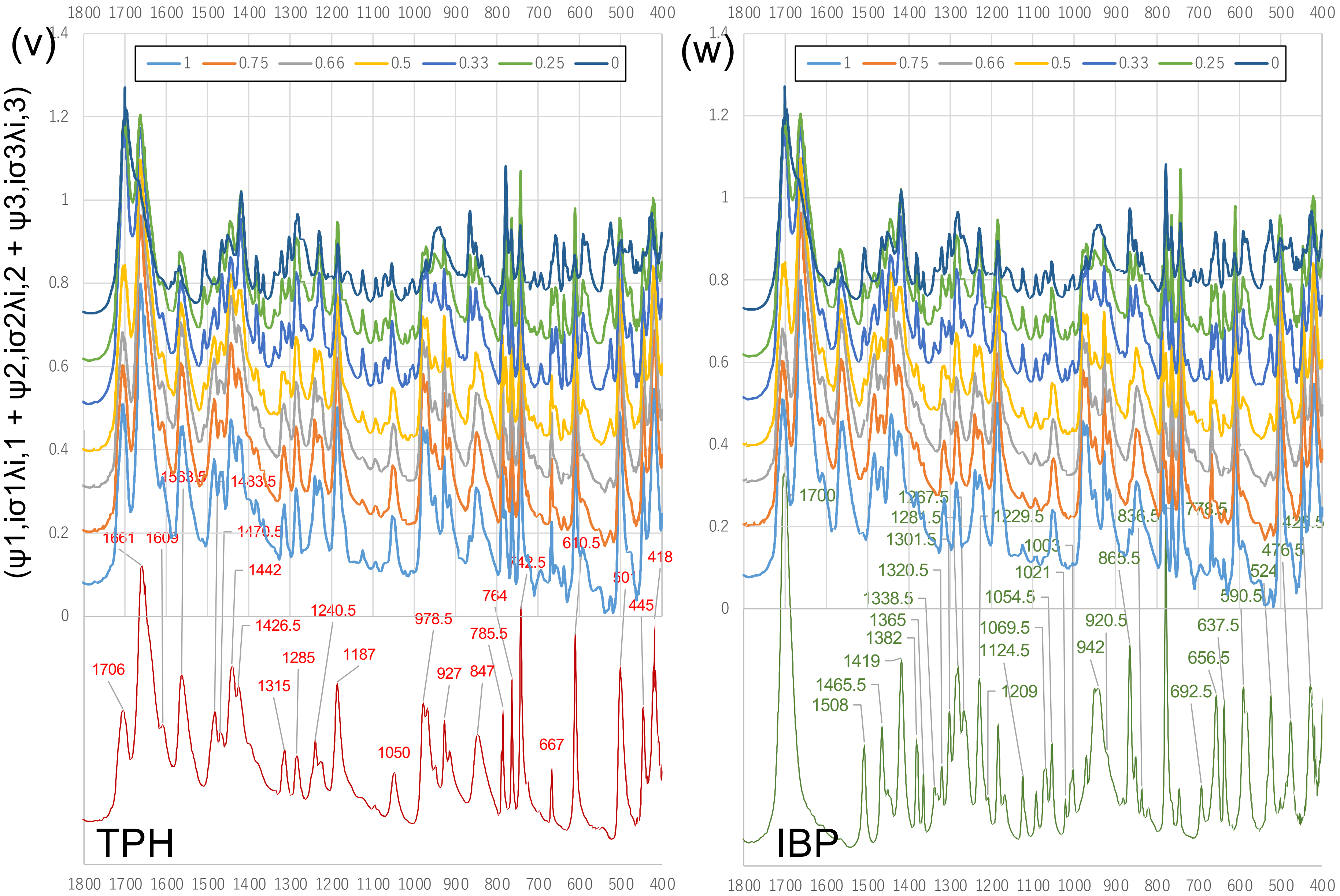
(v)
(w)
1
0.75
0.66
0.5
0.33
0.25
0
(ψ1,iσ1λi,1 + ψ2,iσ2λi,2 + ψ3,iσ3λi,3)
1800 1700 1600 1500 1400 1300 1200 1100 1000 900 800 700 600 500 400
0
0.2
0.4
0.6
0.8
1
1.2
1.4
1661
1609
1563.5
1433.5
1470.5
1442
1426.5
1240.5
1706
1285
1315
1187
1050
978.5
927
847
785.5
764
742.5
667
610.5
501
445
418
TPH
1700
1367.5
1229.5
1301.5
1320.5
1338.5
1365
1382
1419
1465.5
1508
1003
1021
1054.5
1069.5
1124.5
1209
920.5
942
865.5
836.5
778.5
637.5
656.5
692.5
590.5
524
476.5
428.5
IBP

Figure S9

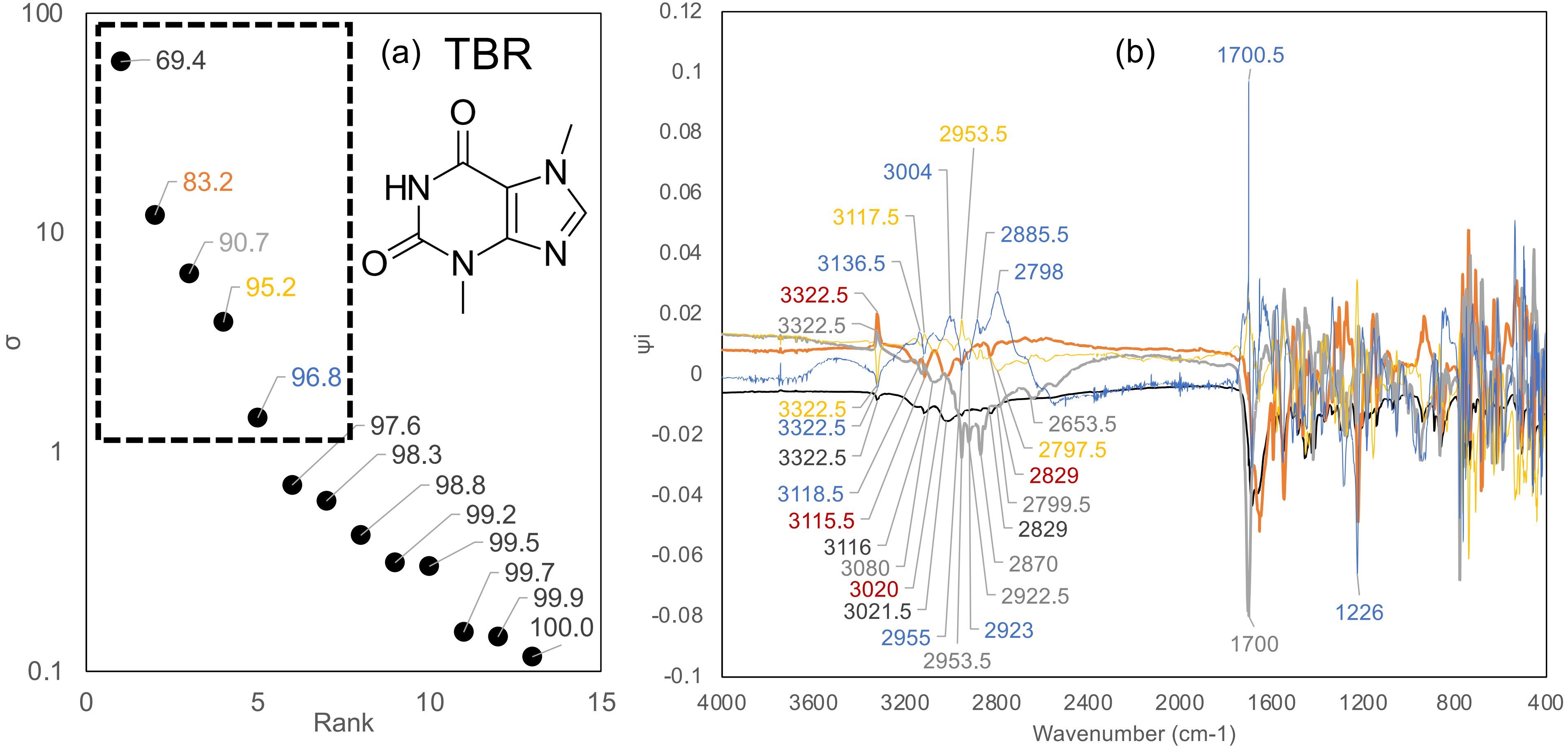

Figure S10. SVD computation for FTIR spectra of TBR/IBP and TBR/DCF mixtures provided singular values σi (a) and Basis spectra ψi(b). Cumulative proportions of σi were appended beside the σ plots, indicating that higher five σ's satisfied 97.6 percent of the variance. The basis spectra decomposed by the SVD computation corresponded to the components at the rank of 1 (black), 2 (orange), 3 (gray), 4 (yellow), and 5 (blue). Details of ψ's assignments to the observed spectra in the wavenumber range 1800-400 cm^{-1} are followed (e-o').

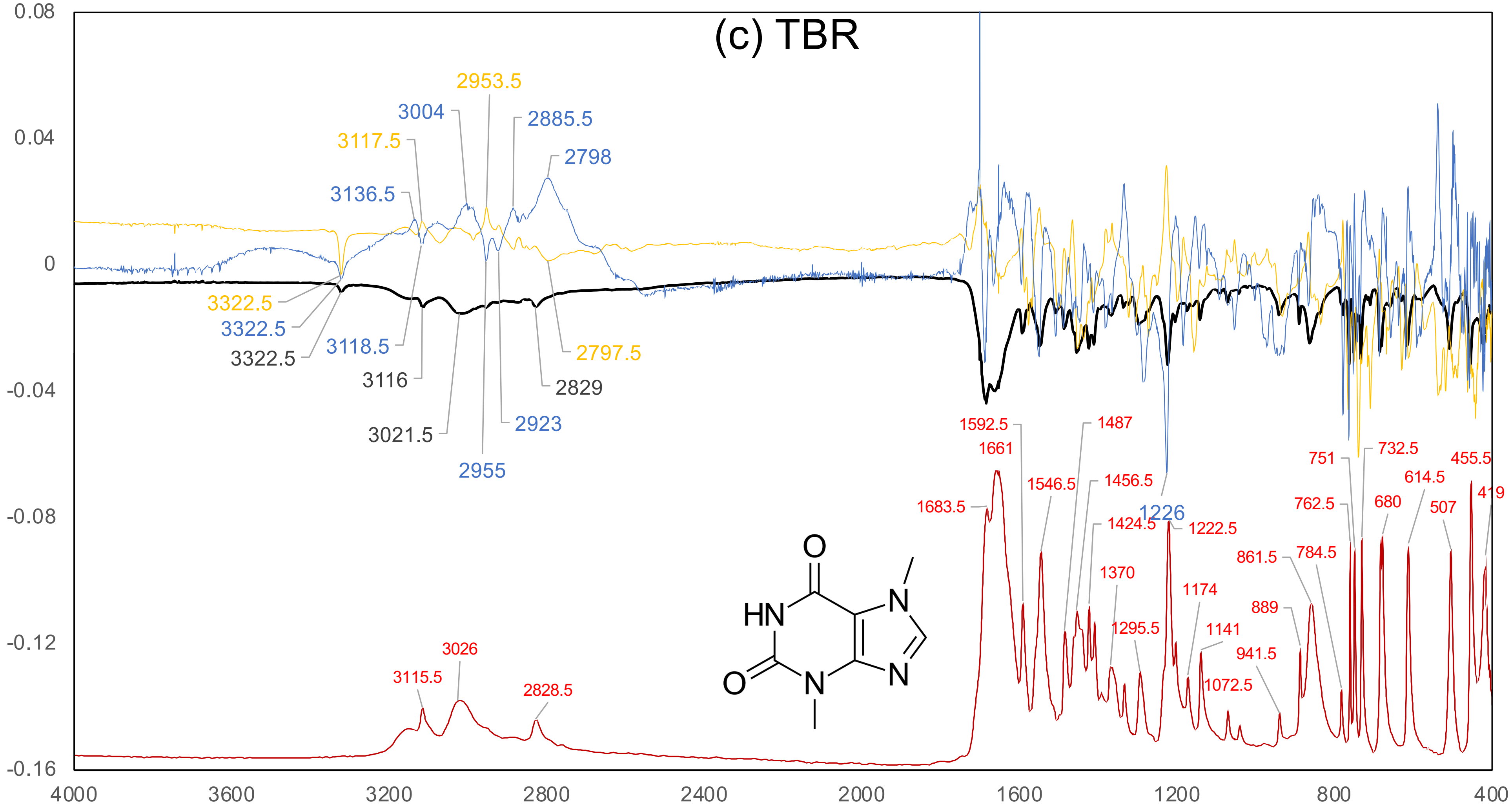

Figure S10. The basis spectra decomposed by the SVD computation corresponded to the components at the rank of 1 (black), 4 (yellow), and 5 (blue).

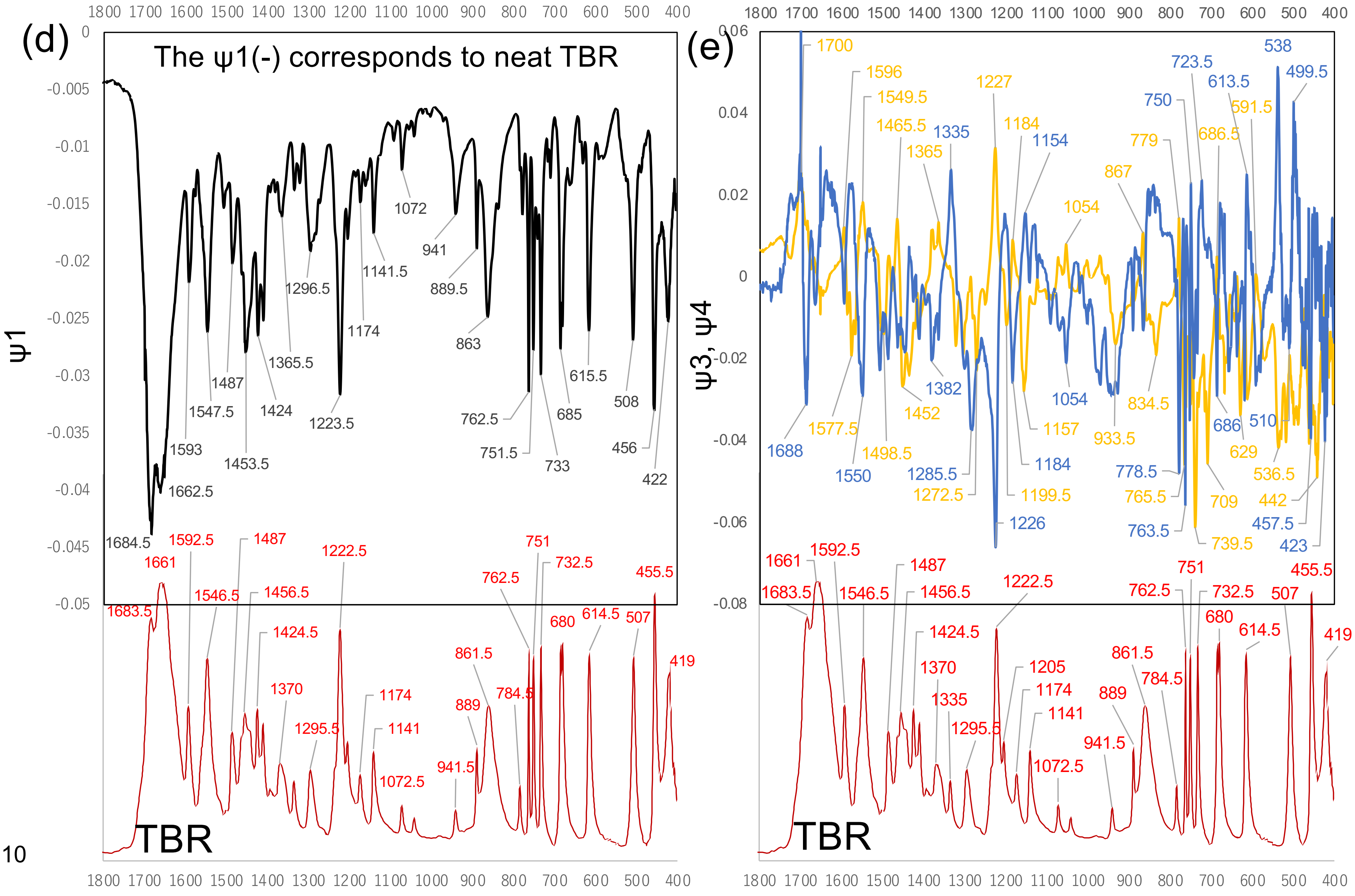
(d)
The ψ1(-) corresponds to neat TBR
ψ1
1684.5
1662.5
1593
1547.5
1487
1453.5
1424
1365.5
1296.5
1223.5
1174
1141.5
1072
941
889.5
863
762.5
751.5
733
685
615.5
508
456
422
1683.5
1661
1592.5
1546.5
1487
1456.5
1424.5
1370
1295.5
1222.5
1174
1141
1072.5
941.5
889
861.5
784.5
762.5
751
732.5
680
614.5
507
455.5
419
TBR
(e)
ψ3, ψ4
1700
1596
1549.5
1465.5
1335
1365
1227
1184
1154
1054
867
779
750
723.5
613.5
591.5
686.5
538
499.5
1688
1577.5
1550
1498.5
1452
1382
1285.5
1272.5
1226
1199.5
1184
1157
1054
933.5
834.5
778.5
765.5
763.5
739.5
709
686
629
510
536.5
442
457.5
423
1661
1683.5
1592.5
1546.5
1487
1456.5
1424.5
1370
1335
1295.5
1222.5
1205
1174
1141
1072.5
941.5
889
861.5
784.5
762.5
751
732.5
680
614.5
507
455.5
419
TBR

Figure S10

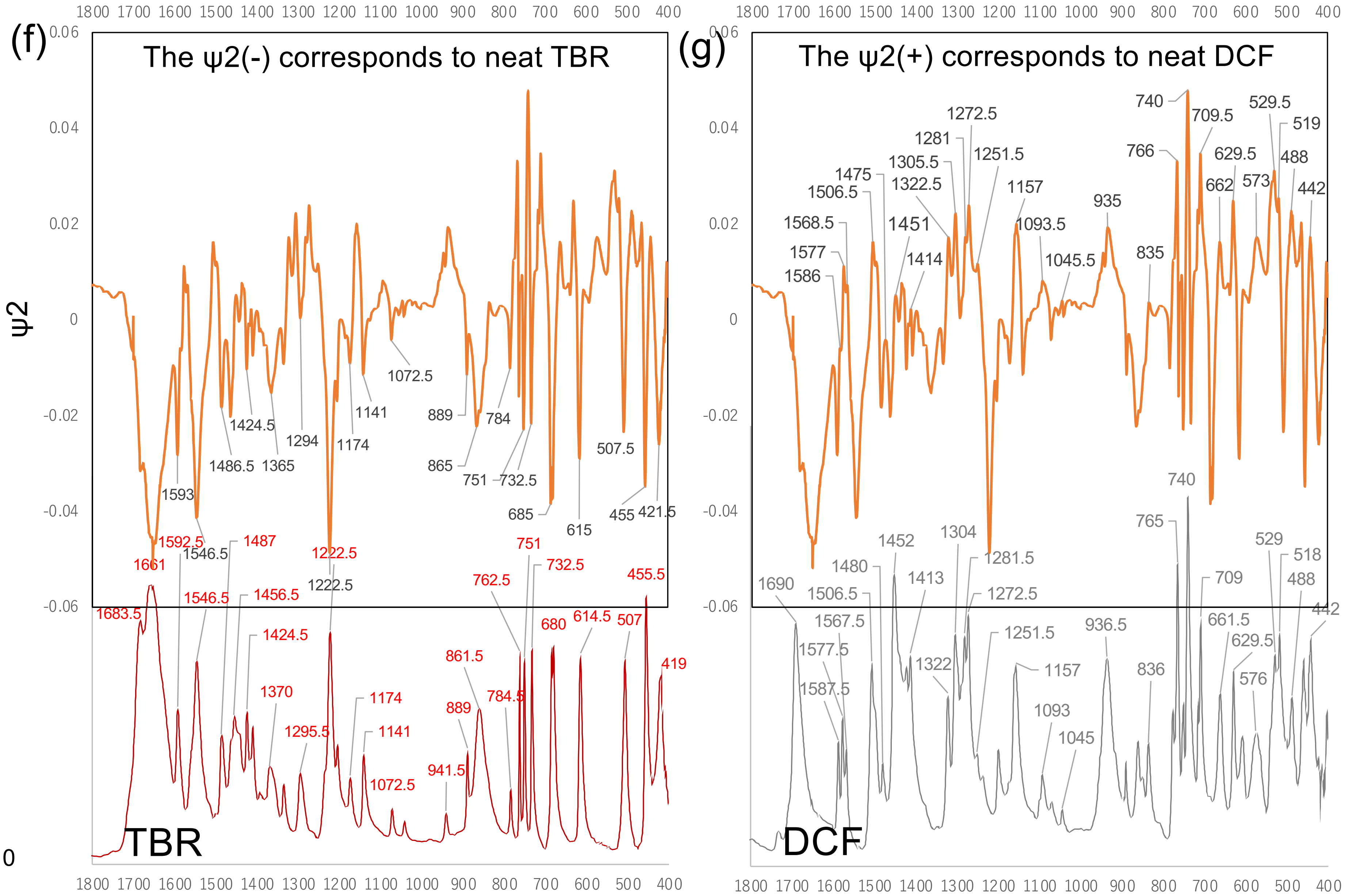
(f)
The ψ2(-) corresponds to neat TBR
ψ2
TBR
(g)
The ψ2(+) corresponds to neat DCF
DCF

Figure S10

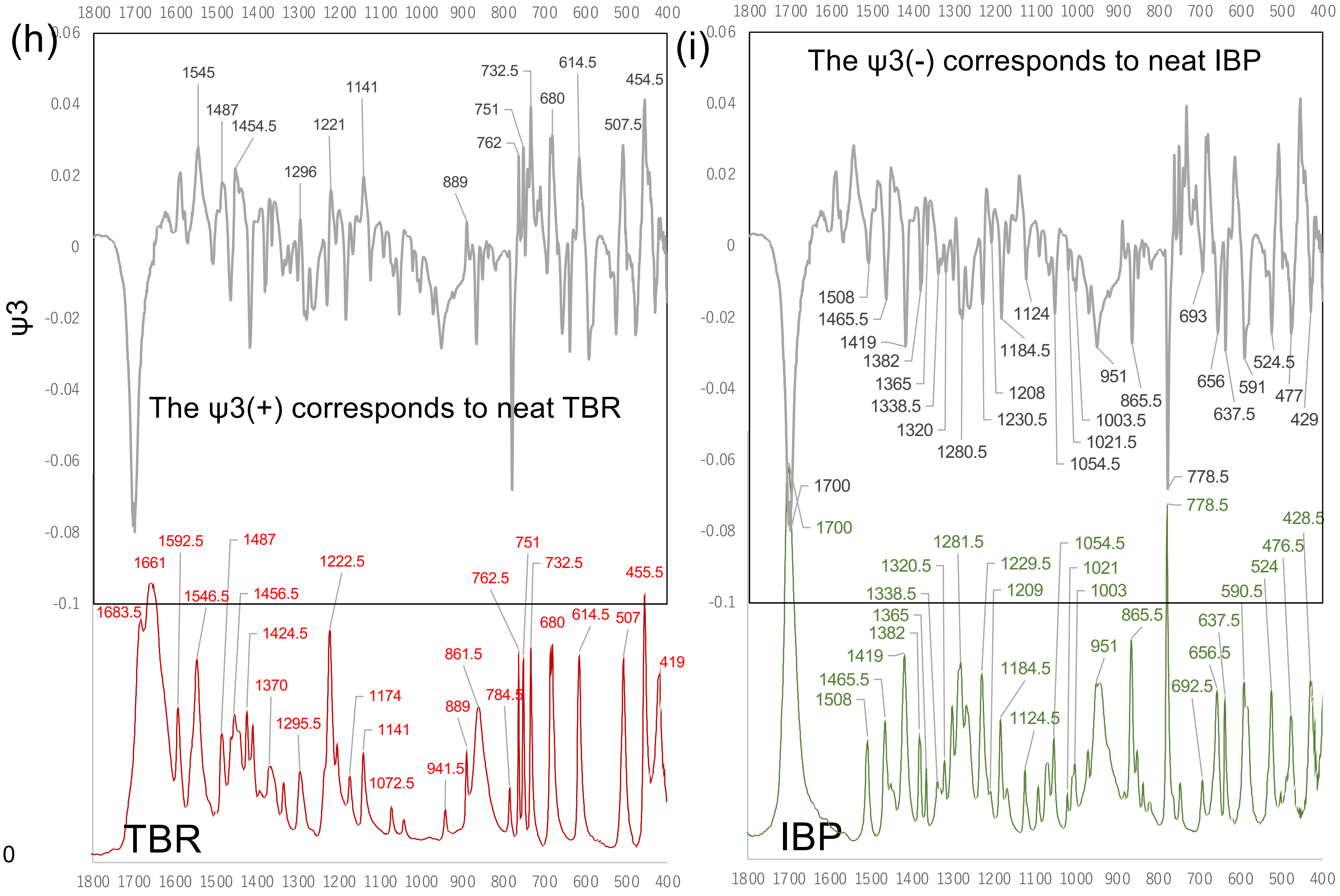
(h)
The ψ3(+) corresponds to neat TBR
TBR
ψ3
1545
1487
1454.5
1296
1221
1141
889
762
751
732.5
680
614.5
507.5
454.5
1683.5
1661
1592.5
1546.5
1487
1456.5
1424.5
1370
1295.5
1222.5
1174
1141
1072.5
941.5
889
861.5
784.5
762.5
751
732.5
680
614.5
507
455.5
419
(i)
The ψ3(-) corresponds to neat IBP
IBP
1508
1465.5
1419
1382
1365
1338.5
1320
1280.5
1230.5
1208
1184.5
1124
1054.5
1021.5
1003.5
951
865.5
778.5
693
656
637.5
591
524.5
477
429
1700
1700
778.5
1281.5
1320.5
1229.5
1209
1054.5
1021
1003
1338.5
1365
1382
1419
1465.5
1508
1184.5
1124.5
951
865.5
692.5
656.5
637.5
590.5
524
476.5
428.5

Figure S10

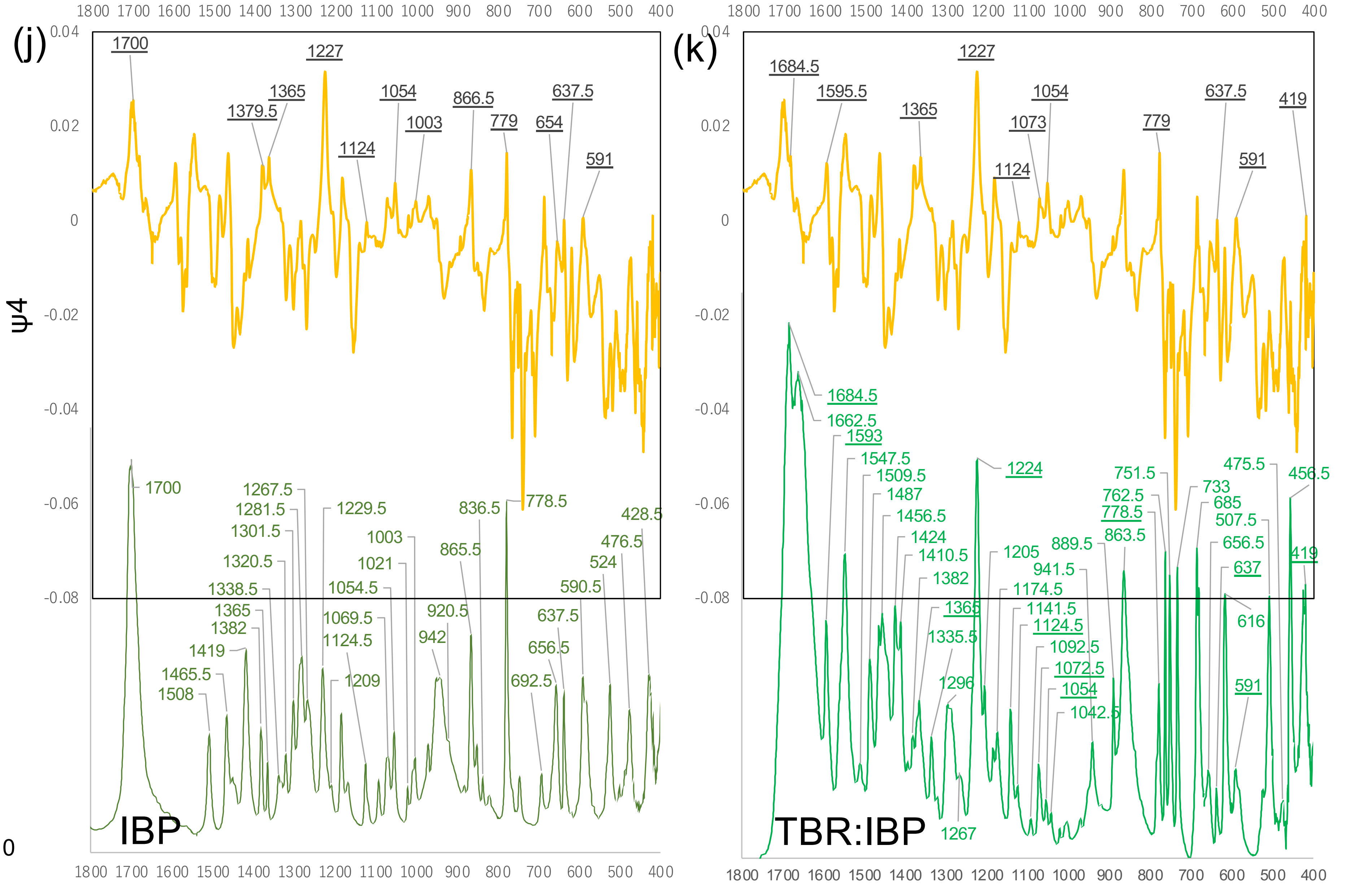

Figure S10

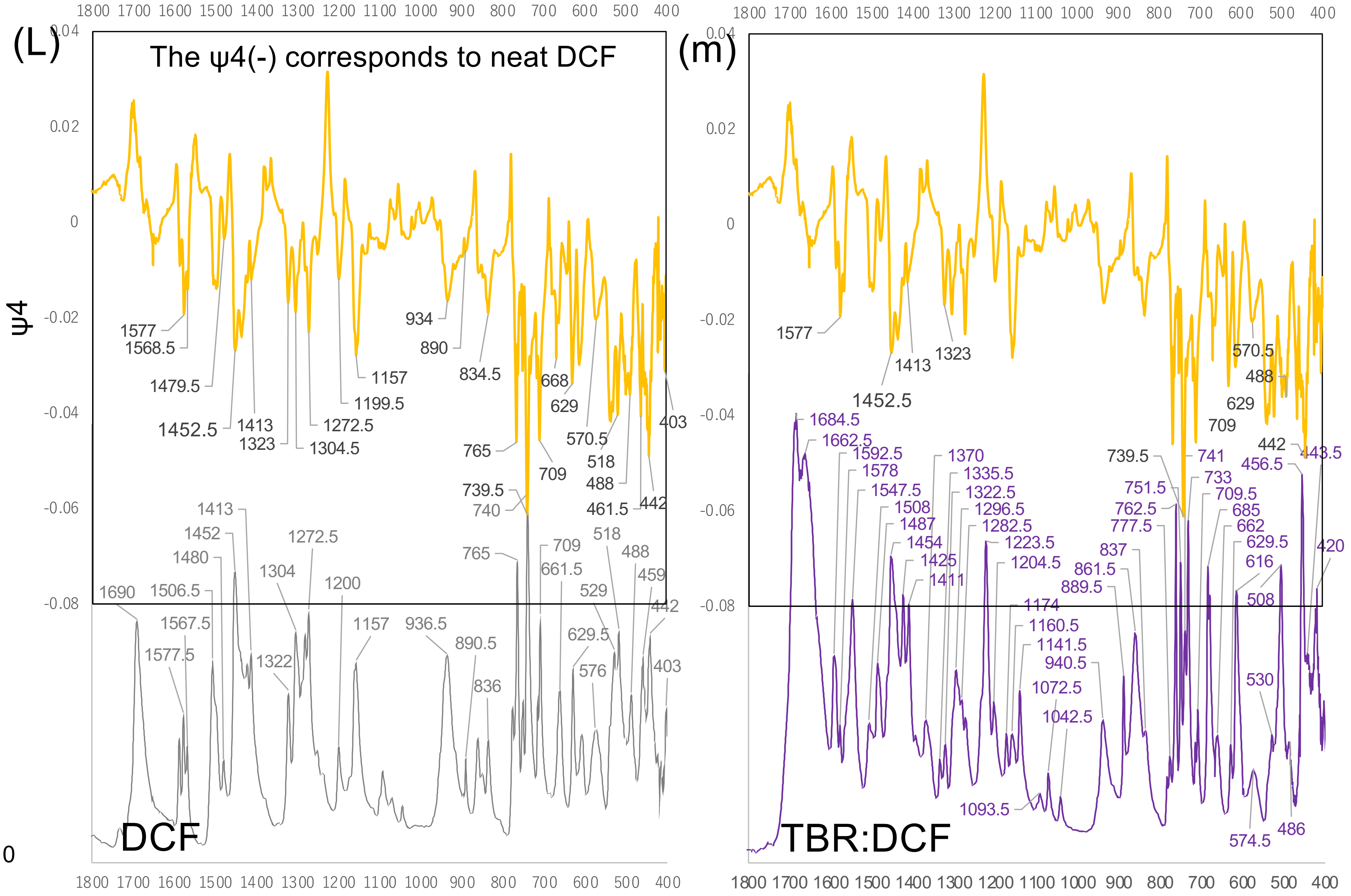

Figure S10

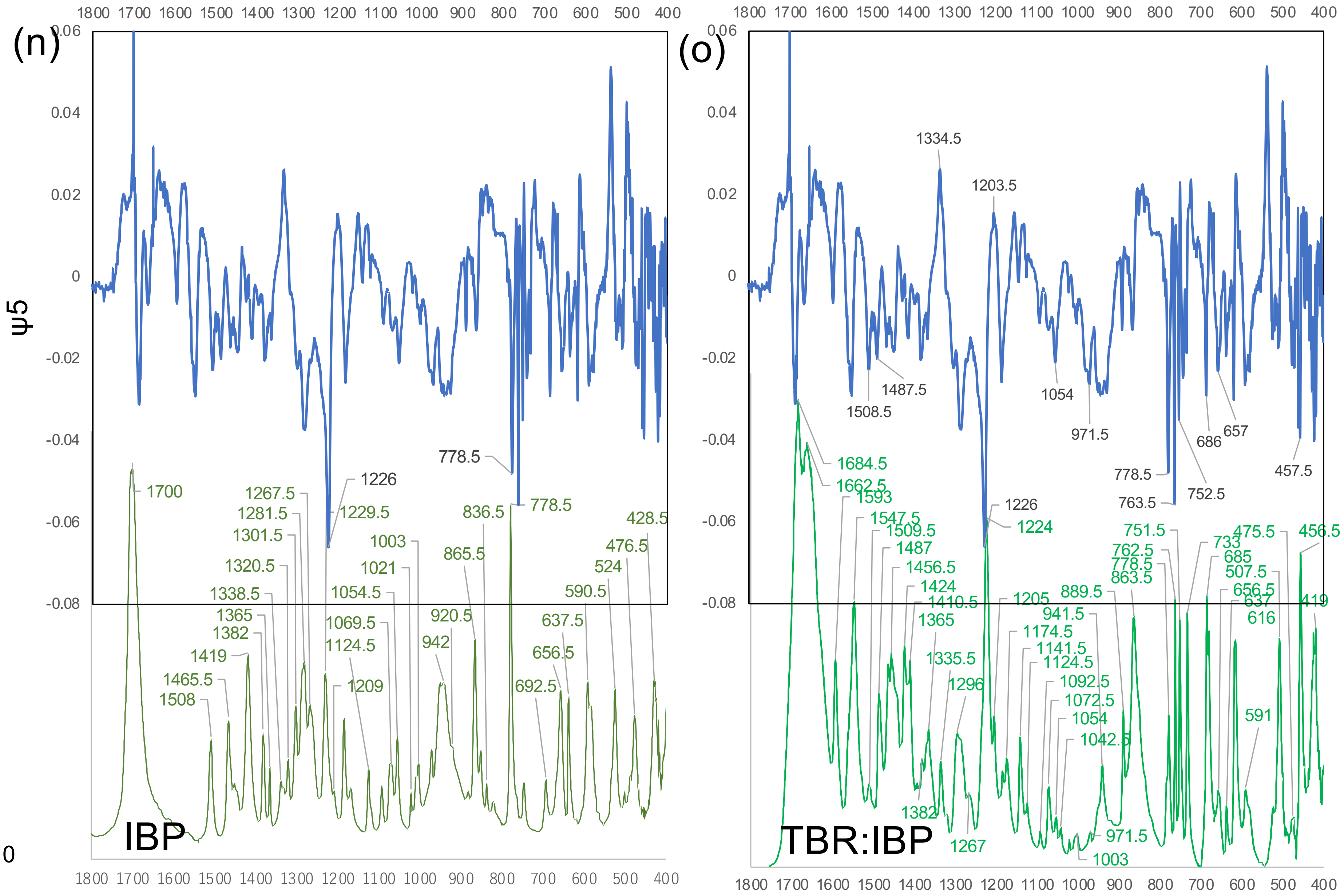
(n)
(o)
ψ5
0.06
0.04
0.02
0
-0.02
-0.04
-0.06
-0.08
1800 1700 1600 1500 1400 1300 1200 1100 1000 900 800 700 600 500 400
IBP
1700
1267.5
1281.5
1301.5
1320.5
1338.5
1365
1382
1419
1465.5
1508
1226
1229.5
1209
1003
1021
1054.5
1069.5
1124.5
778.5
836.5
865.5
920.5
942
778.5
692.5
656.5
637.5
590.5
524
476.5
428.5
TBR:IBP
1334.5
1203.5
1487.5
1508.5
1054
971.5
686
657
457.5
778.5
763.5
752.5
1226
1224
1684.5
1662.5
1593
1547.5
1509.5
1487
1456.5
1424
1410.5
1365
1335.5
1296
1382
1267
1205
1174.5
1141.5
1124.5
1092.5
1072.5
1054
1042.5
1003
971.5
941.5
889.5
863.5
778.5
762.5
751.5
733
685
656.5
637
616
591
507.5
475.5
456.5
419

Figure S10

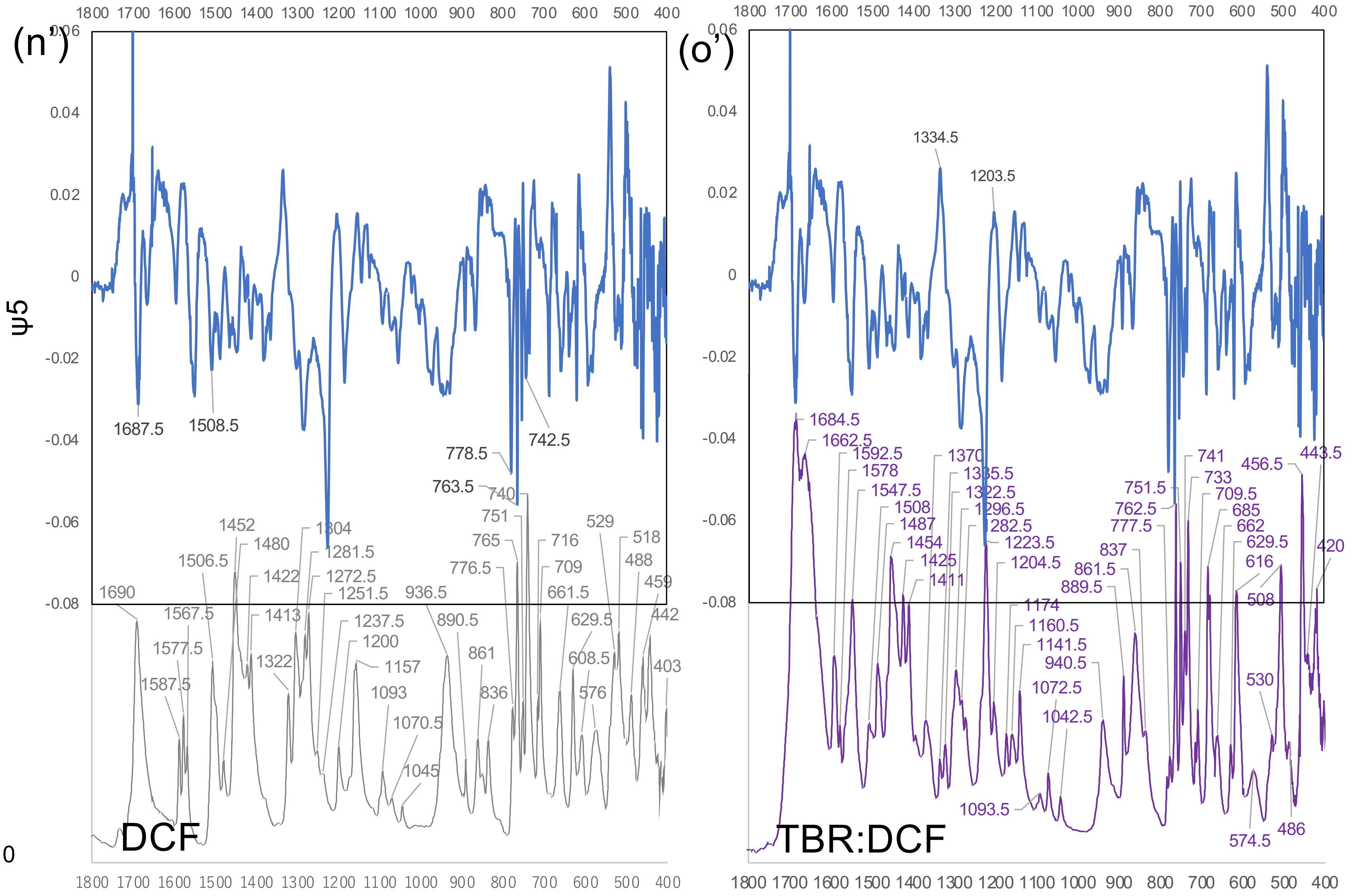
(n')
(o')
ψ5
DCF
TBR:DCF
1687.5
1508.5
778.5
763.5
742.5
740
751
765
776.5
1690
1567.5
1577.5
1587.5
1506.5
1452
1480
1422
1413
1322
1304
1281.5
1272.5
1251.5
1237.5
1200
1157
1093
1070.5
1045
936.5
890.5
861
836
716
709
661.5
629.5
608.5
576
529
518
488
459
442
403
1334.5
1203.5
1684.5
1662.5
1592.5
1578
1547.5
1508
1487
1454
1425
1411
1370
1335.5
1322.5
1296.5
1282.5
1223.5
1204.5
1174
1160.5
1141.5
940.5
1072.5
1042.5
1093.5
837
861.5
889.5
751.5
762.5
777.5
741
733
709.5
685
662
629.5
616
508
530
574.5
486
456.5
443.5
420

Figure S10

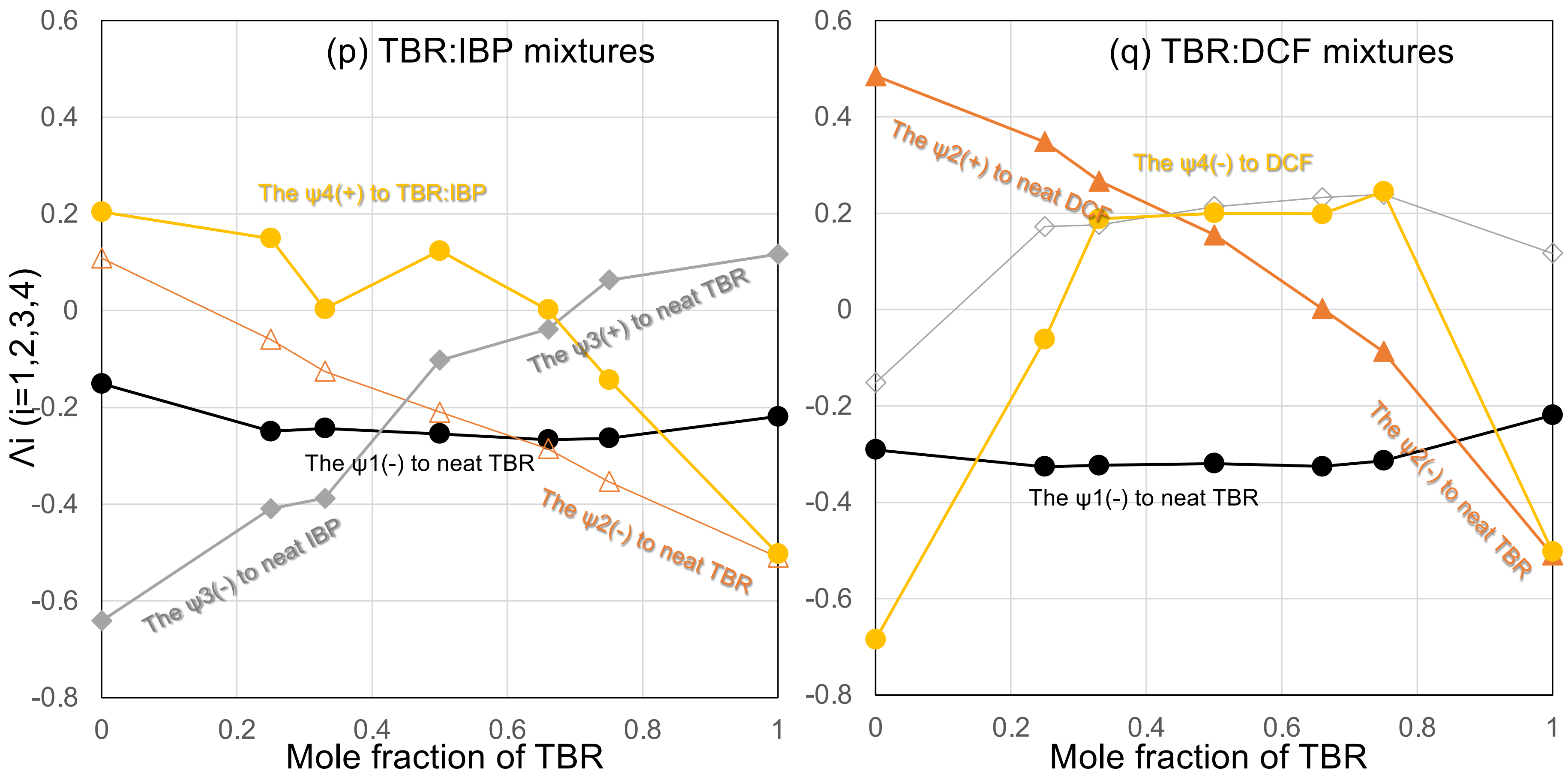

Figure S10 (p and q). The components of the first to fifth singular vectors as a function of mole fraction of TBR. The λ1, λ2, λ3, and λ4 were represented by closed black circles, orange triangles, gray diamonds, and yellow circles. The ψ5 was not assigned to any spectra, as shown in Figure S12 (n-o'). For λ2, λ3, and λ4, closed and open symbols corresponded to the composition with the basis functions correlated to which the IBP or DCF mixtures, respectively.

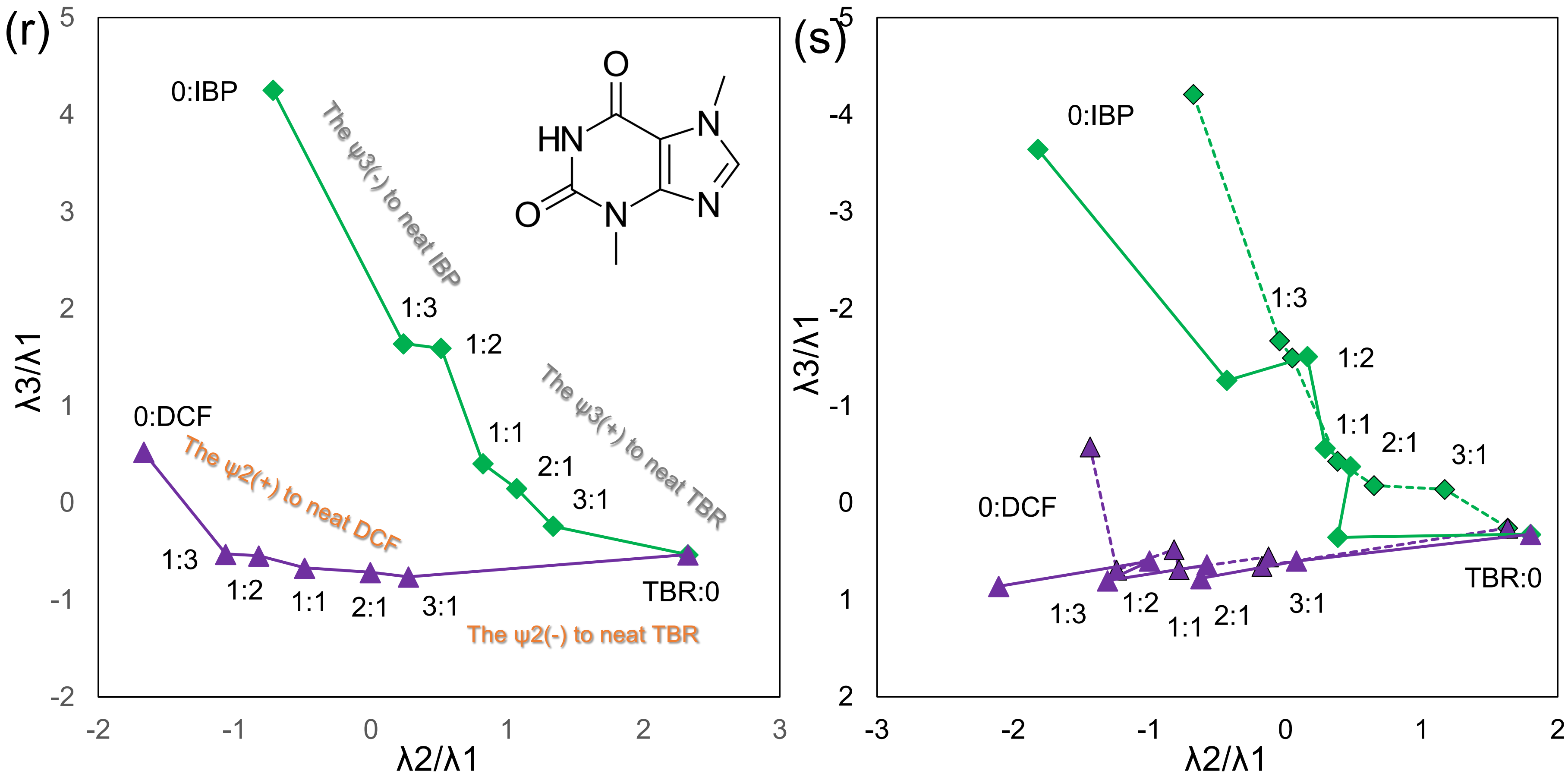

Figure S10 (r and s). The trajectories of the TBR/IBP (green diamonds) and TBR/DCF (violet triangles) mixtures. They shared the neat TBR's vector (TBR:0). The molar ratios were represented along the corresponding plots. The trajectory of the TBR/DCF mixtures traced along the (λ2/λ1)-axis (DCF owning dominance in the SVD analysis), whereas that of the TBR/IBP mixtures pursued in proportion to the diagonal line, suggesting the dependence between the trajectories of the TBR/IBP and TBR/DCF mixtures. The S13(r) and S13(s) show the trajectories of the average spectra and the individually measured spectra, respectively. For latter, the (λ2/λ1)-axis flip-flopped to adjust to the former diagram.

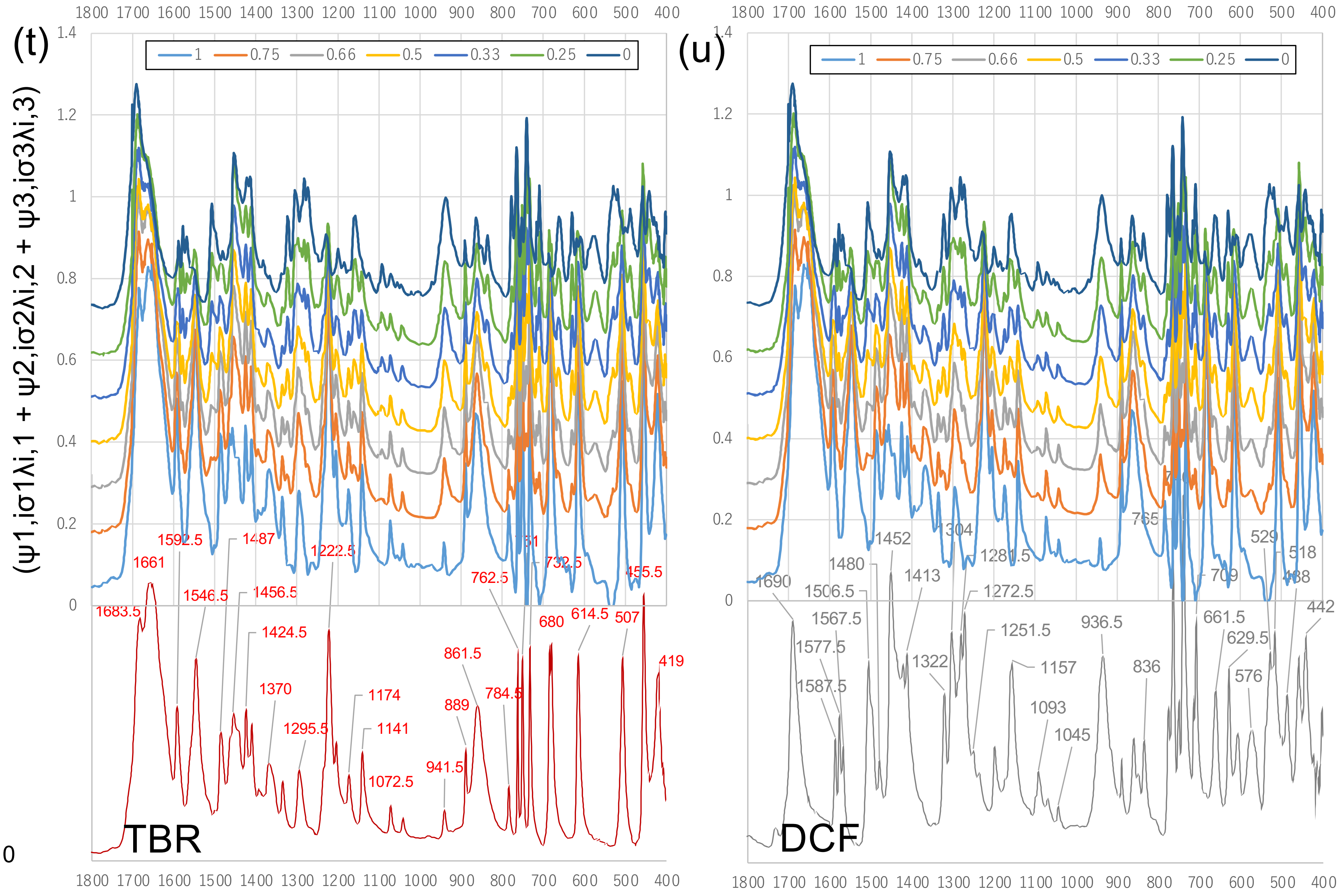
(t)
(u)
(ψ1,iσ1λi,1 + ψ2,iσ2λi,2 + ψ3,iσ3λi,3)
1
0.75
0.66
0.5
0.33
0.25
0
TBR
DCF
1683.5
1661
1592.5
1546.5
1487
1456.5
1424.5
1370
1295.5
1222.5
1174
1141
1072.5
941.5
889
861.5
784.5
762.5
732.5
680
614.5
507
455.5
419
1690
1587.5
1577.5
1567.5
1506.5
1480
1452
1413
1322
1304
1281.5
1272.5
1251.5
1157
1093
1045
936.5
836
765
709
661.5
629.5
576
529
518
488
442

Figure S10

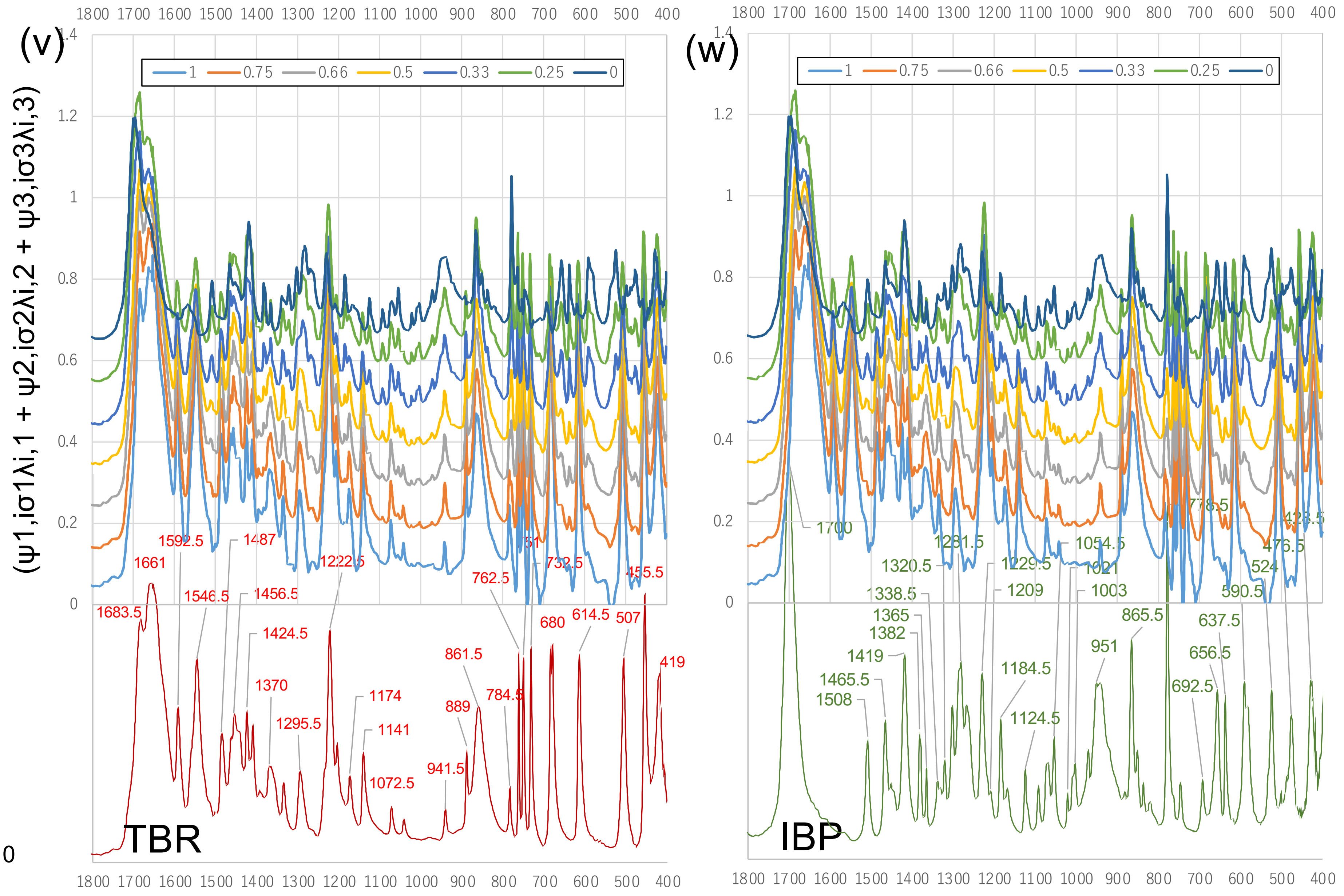

(v)
(w)
1 0.75 0.66 0.5 0.33 0.25 0
(ψ1,iσ1λi,1 + ψ2,iσ2λi,2 + ψ3,iσ3λi,3)
1800 1700 1600 1500 1400 1300 1200 1100 1000 900 800 700 600 500 400
0 0.2 0.4 0.6 0.8 1 1.2 1.4
TBR
1683.5 1661 1592.5 1546.5 1487 1456.5 1424.5 1370 1295.5 1222.5 1174 1141 1072.5 941.5 889 861.5 784.5 762.5 751 732.5 680 614.5 507 455.5 419
IBP
1700 1508 1465.5 1419 1382 1365 1338.5 1320.5 1281.5 1229.5 1209 1184.5 1124.5 1054.5 1021 1003 951 865.5 778.5 692.5 656.5 637.5 590.5 524 476.5 428.5

Figure S10

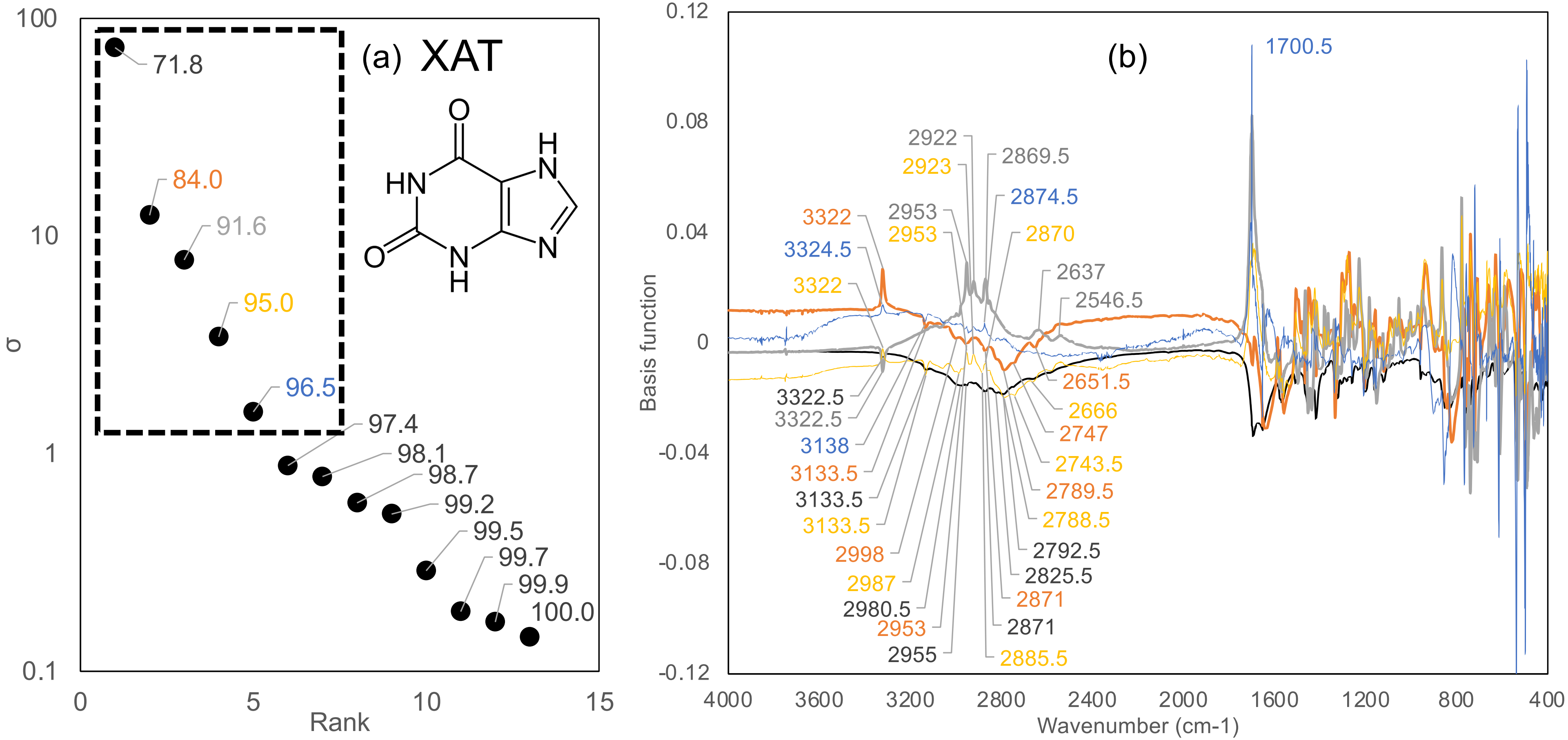

Figure S11. SVD computation for FTIR spectra of XAT/IBP and XAN/DCF mixtures provided singular values σi (a) and Basis spectra ψi(b). Cumulative proportions of σi were appended beside the σ plots, indicating that higher five σ's satisfied 97.6 percent of the variance. The basis spectra decomposed by the SVD computation corresponded to the components at the rank of 1 (black), 2 (orange), 3 (gray), 4 (yellow), and 5 (blue). Details of ψ's assignments to the observed spectra in the wavenumber range 1800-400 cm^{-1} are followed (e-o').

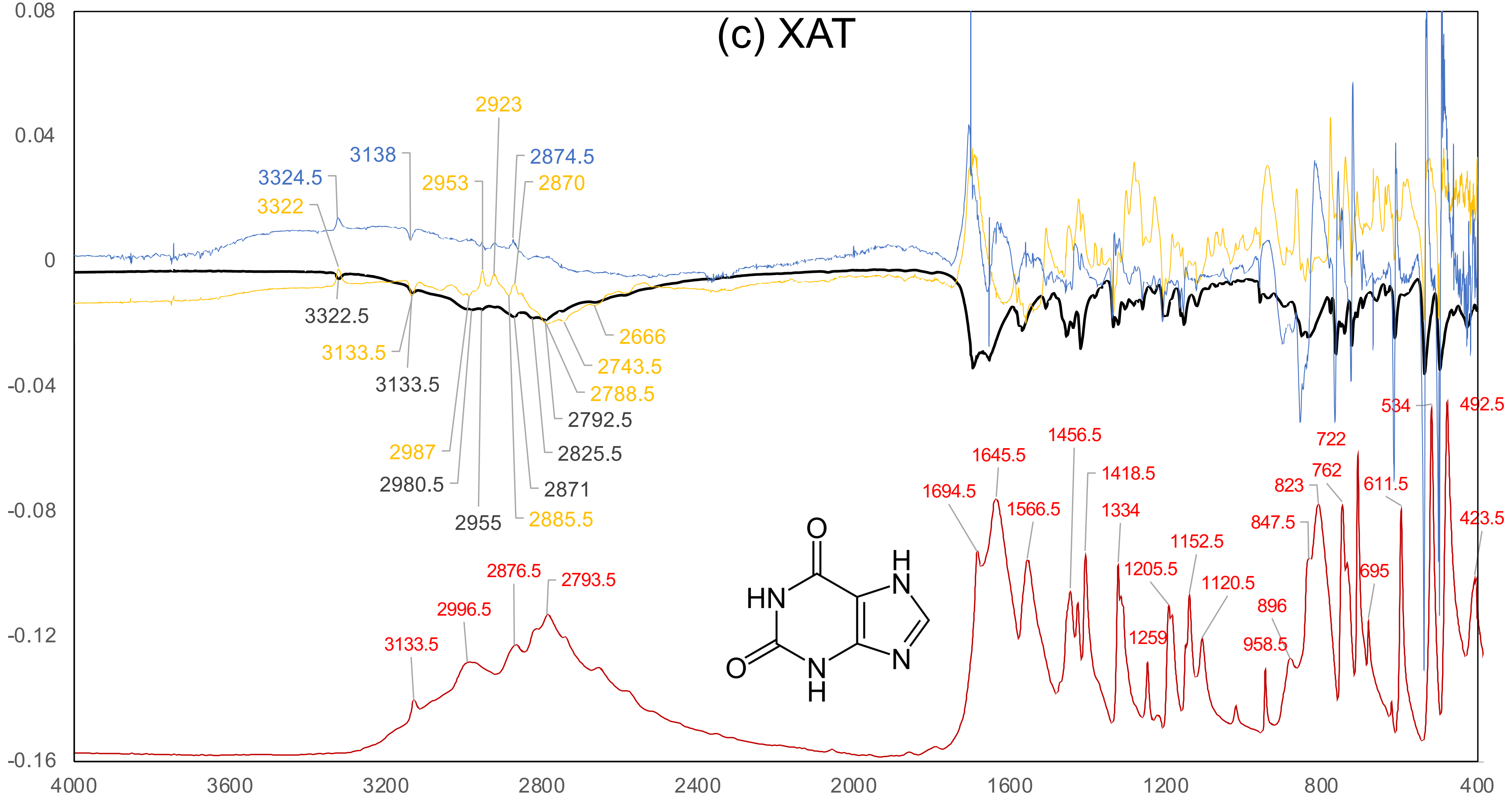

Figure S11. The basis spectra decomposed by the SVD computation corresponded to the components at the rank of 1 (black), 4 (yellow), and 5 (blue).

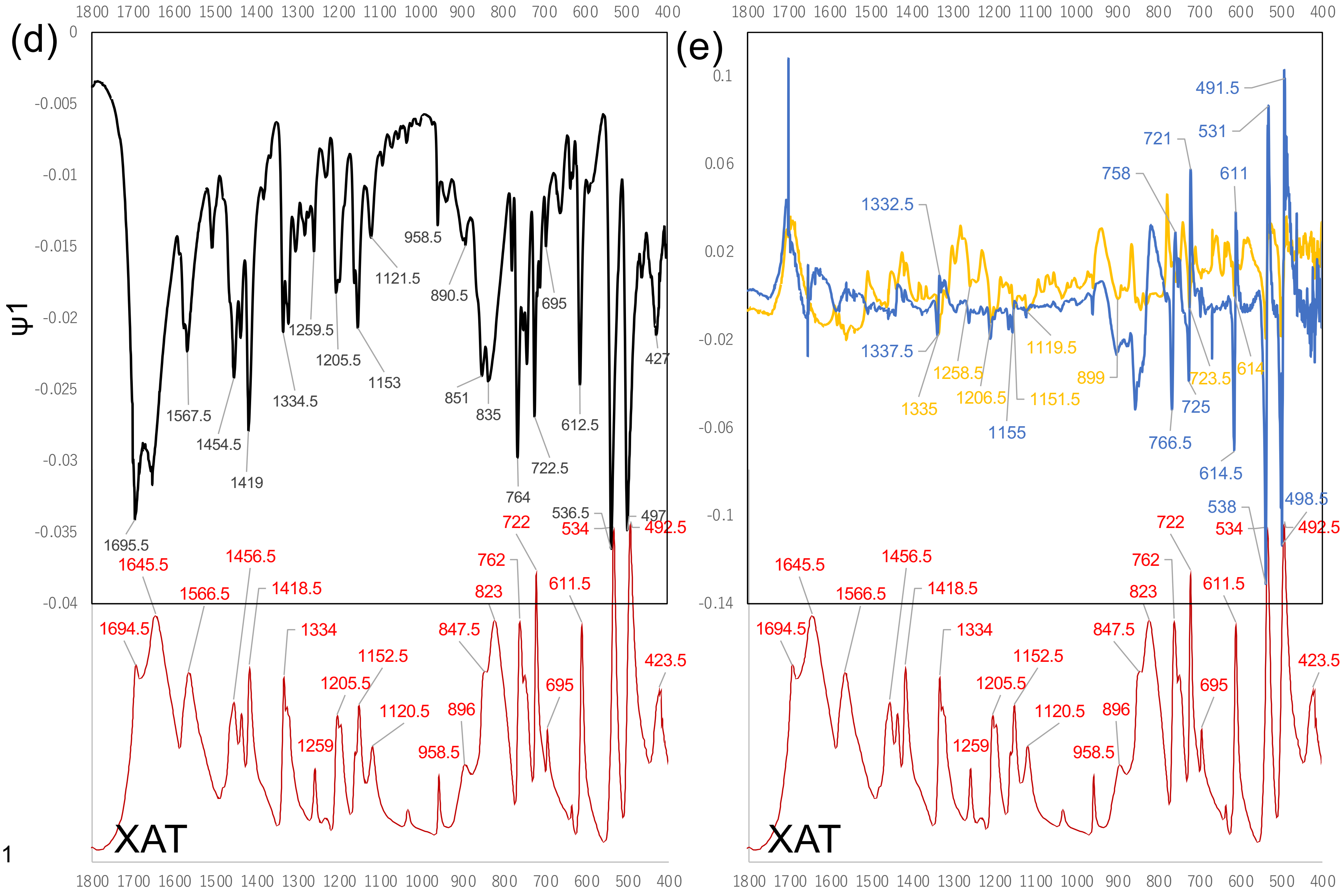
(d)
(e)
ψ1
XAT
XAT
1695.5
1567.5
1454.5
1419
1334.5
1259.5
1205.5
1153
1121.5
958.5
890.5
851
835
764
722.5
695
612.5
536.5
497
427
1332.5
1337.5
1335
1258.5
1206.5
1155
1151.5
1119.5
899
758
766.5
721
723.5
725
611
614
614.5
531
491.5
538
498.5
1694.5
1645.5
1566.5
1456.5
1418.5
1334
1259
1205.5
1152.5
1120.5
958.5
896
847.5
823
762
722
695
611.5
534
492.5
423.5

Figure S11

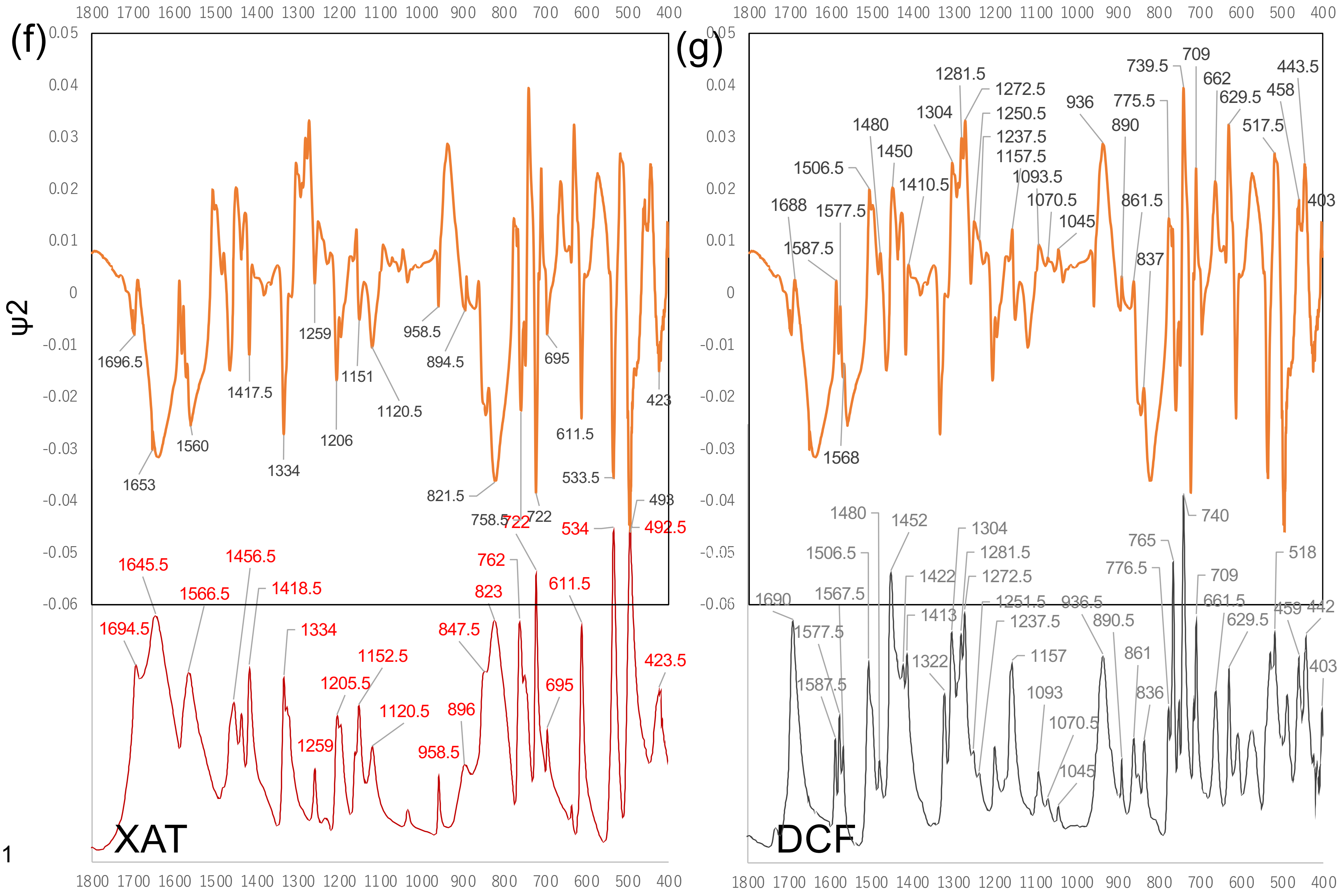

Figure S11

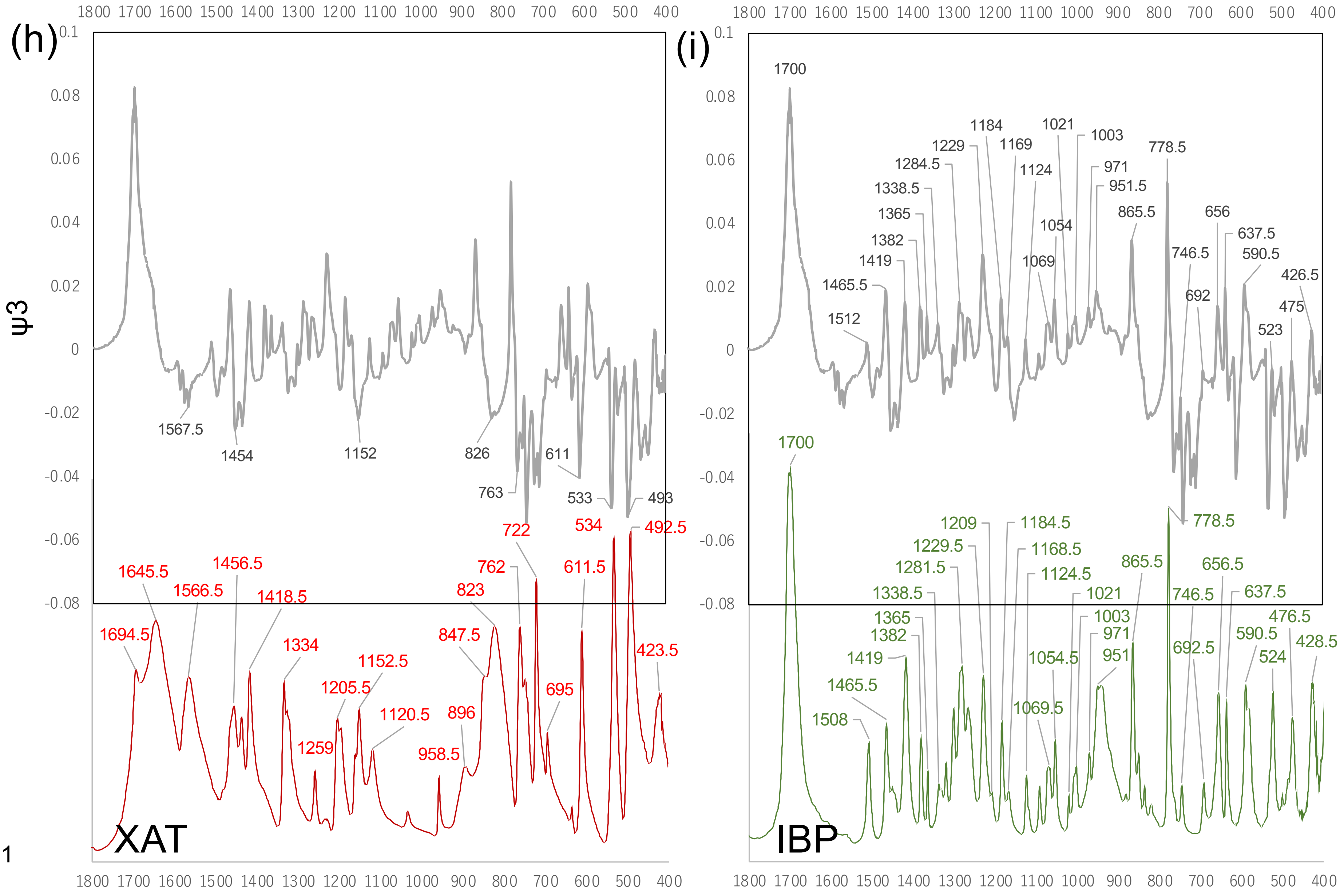
(h)
(i)
ψ3
XAT
IBP
1800 1700 1600 1500 1400 1300 1200 1100 1000 900 800 700 600 500 400
0.1
0.08
0.06
0.04
0.02
0
-0.02
-0.04
-0.06
-0.08
1567.5
1454
1152
826
763
611
533
493
1645.5
1456.5
1566.5
1418.5
1694.5
1334
1152.5
1205.5
1120.5
1259
896
958.5
847.5
823
762
722
534
492.5
611.5
695
423.5
1700
1184
1021
1229
1169
1003
778.5
1284.5
971
1124
951.5
1338.5
1365
1054
865.5
656
1382
637.5
1419
1069
746.5
590.5
1465.5
426.5
692
1512
475
523
1209
1184.5
1229.5
1168.5
1281.5
1124.5
865.5
656.5
1338.5
1021
746.5
637.5
1365
1003
476.5
1382
971
590.5
428.5
1419
1054.5
951
692.5
524
1465.5
1069.5
1508

Figure S11

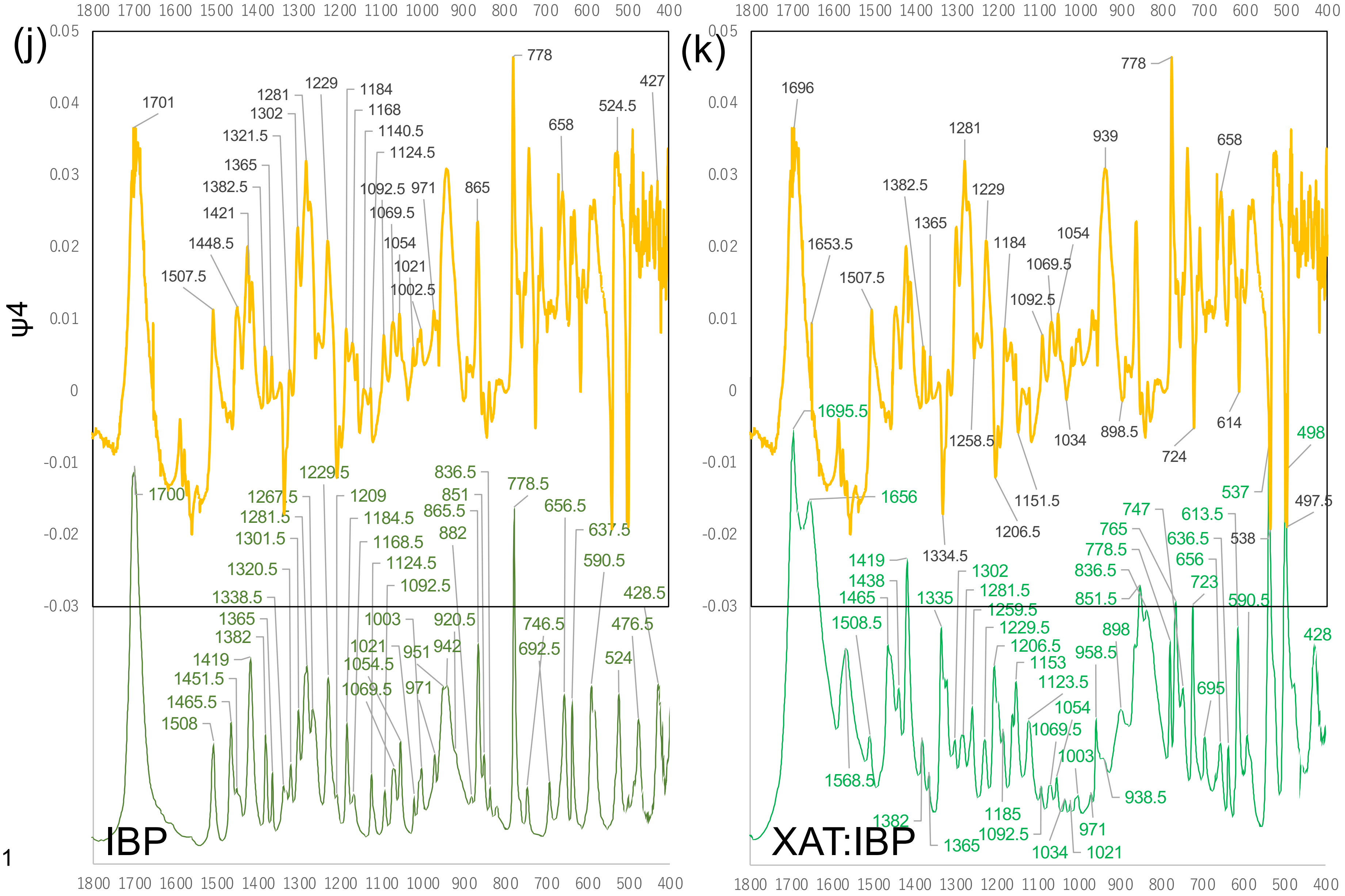

Figure S11

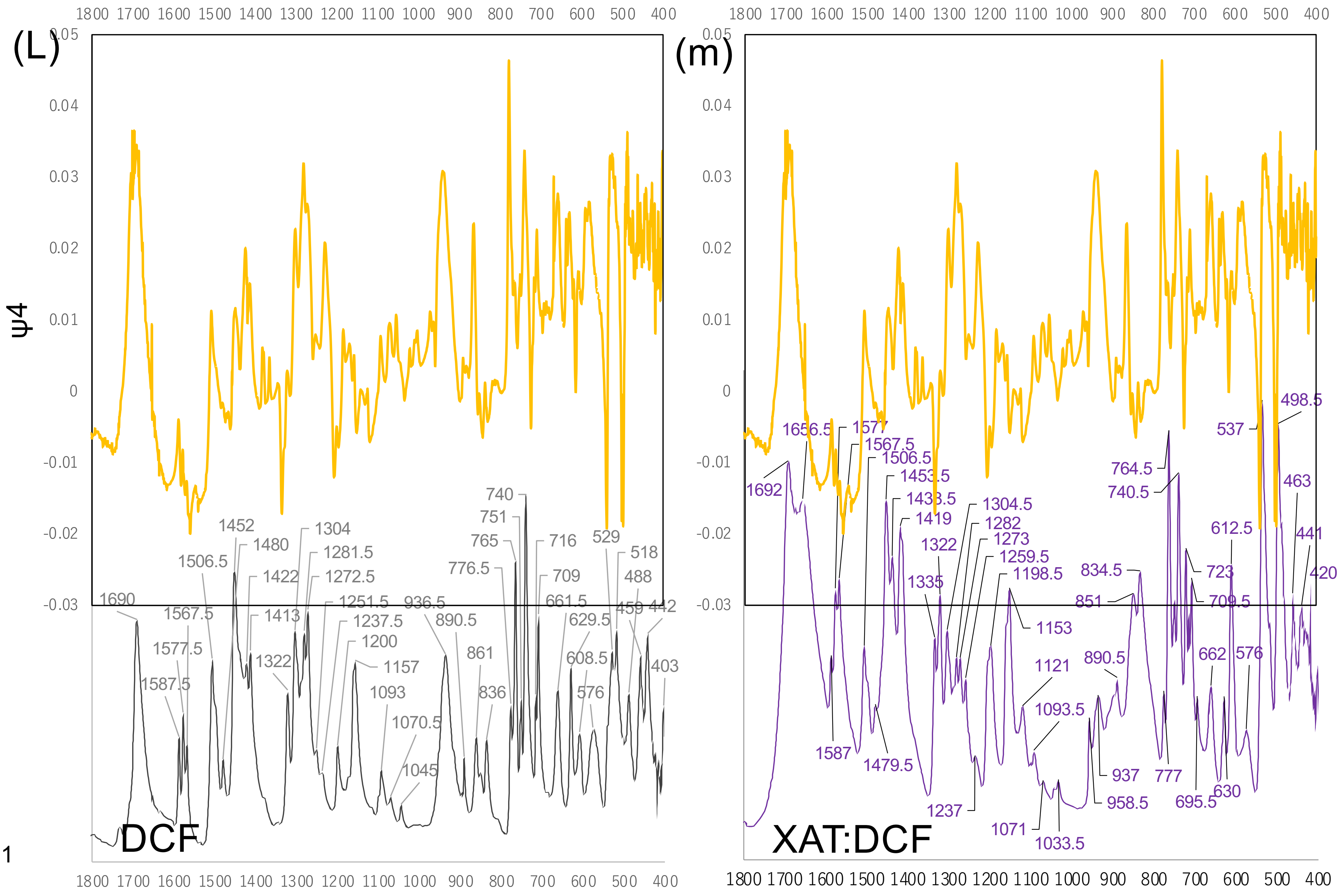
(L)
(m)
ψ4
DCF
XAT:DCF
1800 1700 1600 1500 1400 1300 1200 1100 1000 900 800 700 600 500 400
0.05
0.04
0.03
0.02
0.01
0
-0.01
-0.02
-0.03
1690
1567.5
1577.5
1587.5
1506.5
1452
1480
1422
1413
1322
1304
1281.5
1272.5
1251.5
1237.5
1200
1157
1093
1070.5
1045
936.5
890.5
861
836
776.5
765
751
740
716
709
661.5
629.5
608.5
576
529
518
488
459
442
403
1692
1656.5
1577
1567.5
1587
1506.5
1479.5
1453.5
1433.5
1419
1335
1304.5
1282
1273
1259.5
1237
1198.5
1153
1121
1093.5
1071
1033.5
958.5
937
890.5
851
834.5
777
764.5
740.5
723
709.5
695.5
662
630
612.5
576
537
498.5
463
441
420

Figure S11

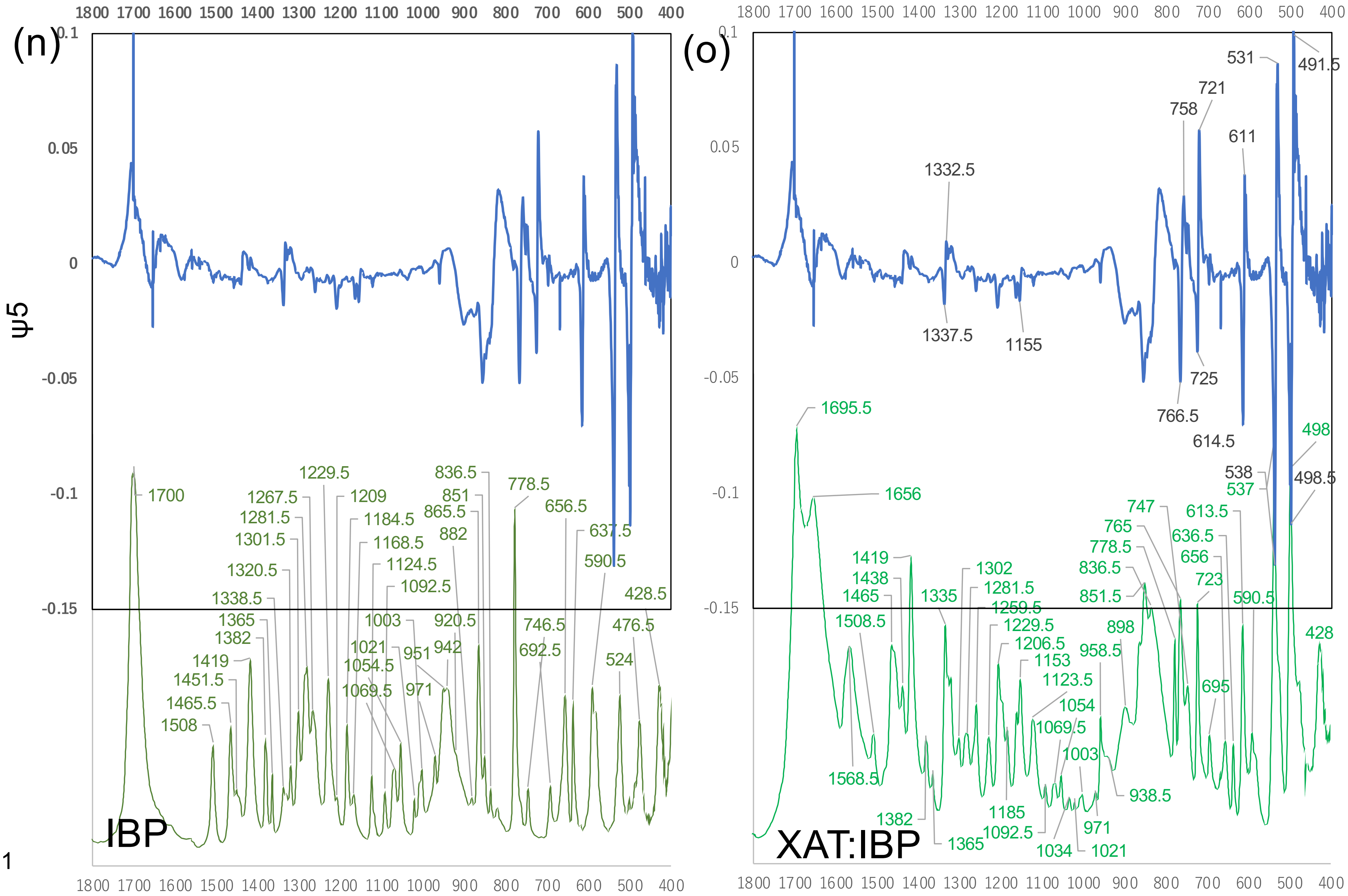
(n)
(o)
ψ5
0.1
0.05
0
-0.05
-0.1
-0.15
1800 1700 1600 1500 1400 1300 1200 1100 1000 900 800 700 600 500 400
IBP
XAT:IBP
1700
1229.5
1267.5
1209
1281.5
1184.5
1301.5
1168.5
1320.5
1124.5
1092.5
1338.5
1365
1003
1382
1021
1419
1054.5
1451.5
1069.5
1465.5
971
951
942
1508
836.5
851
865.5
882
920.5
778.5
656.5
637.5
590.5
746.5
692.5
428.5
476.5
524
531
491.5
721
758
611
1332.5
1337.5
1155
725
766.5
614.5
538
537
498.5
498
1695.5
1656
747
613.5
765
636.5
778.5
656
836.5
723
851.5
590.5
1419
1438
1465
1335
1302
1281.5
1259.5
1229.5
1206.5
1153
1123.5
1054
1069.5
1003
1508.5
1568.5
898
958.5
938.5
695
428
1185
1092.5
971
1382
1365
1034
1021

Figure S11

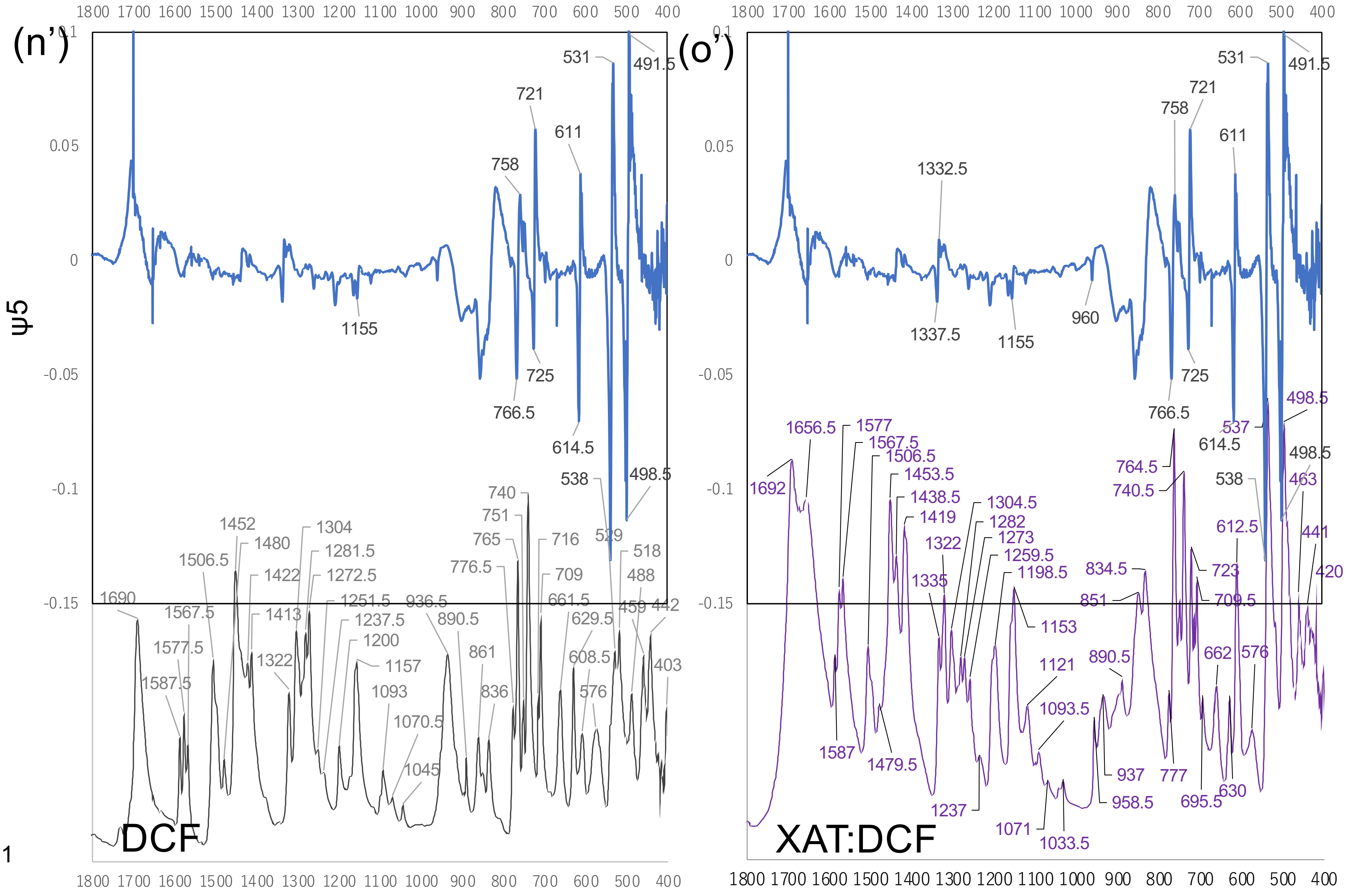

Figure S11

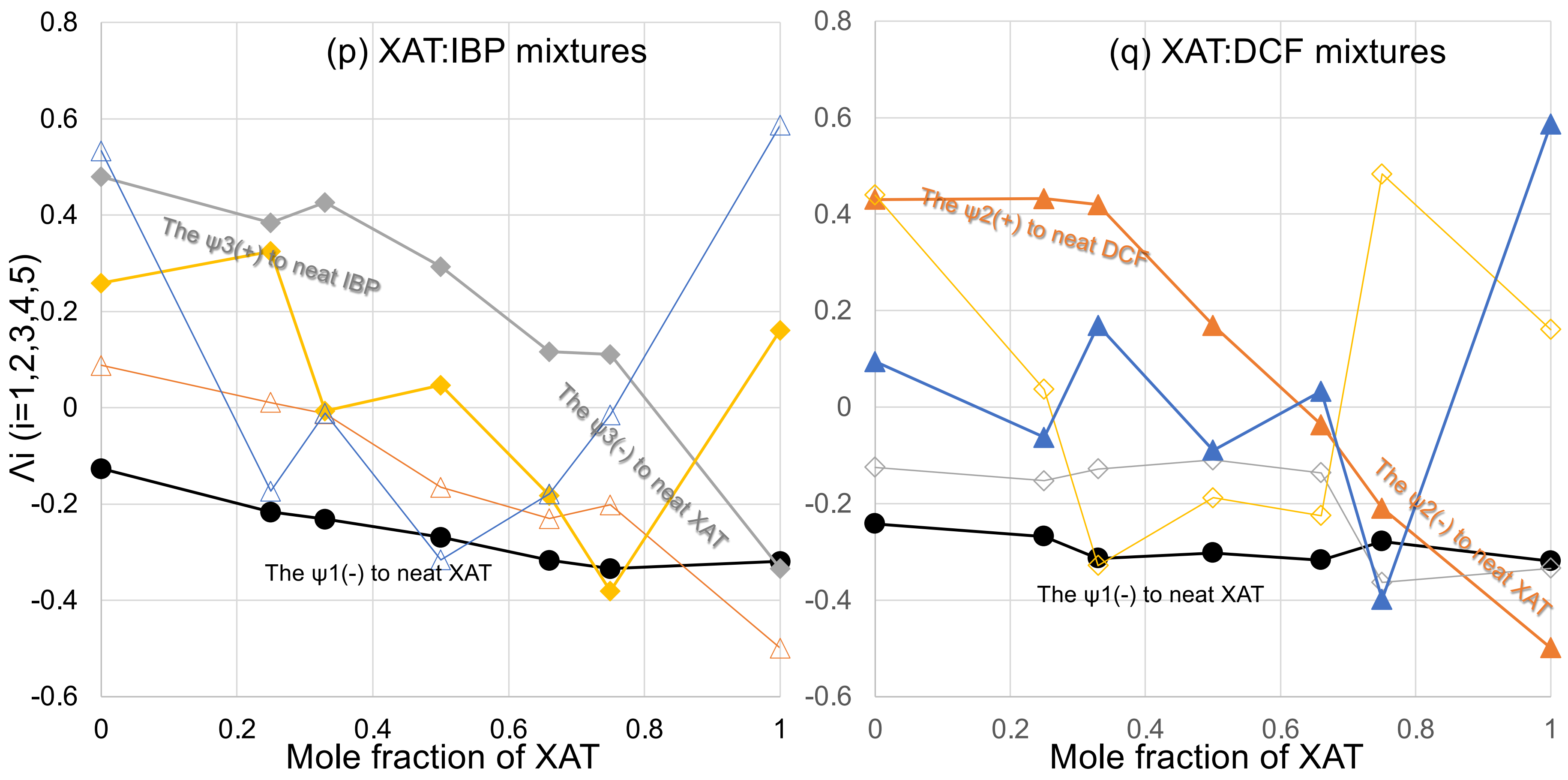

Figure S11 (p and q). The components of the first to fifth singular vectors as a function of mole fraction of XAT. The λ1, λ2, λ3, λ4, and λ5 were represented by closed black circles, orange triangles, gray diamonds, yellow diamonds, and blue triangles. For λ2, λ3, λ4, and λ5, closed and open symbols corresponded to the composition with the basis functions correlated to which the IBP or DCF mixtures, respectively.

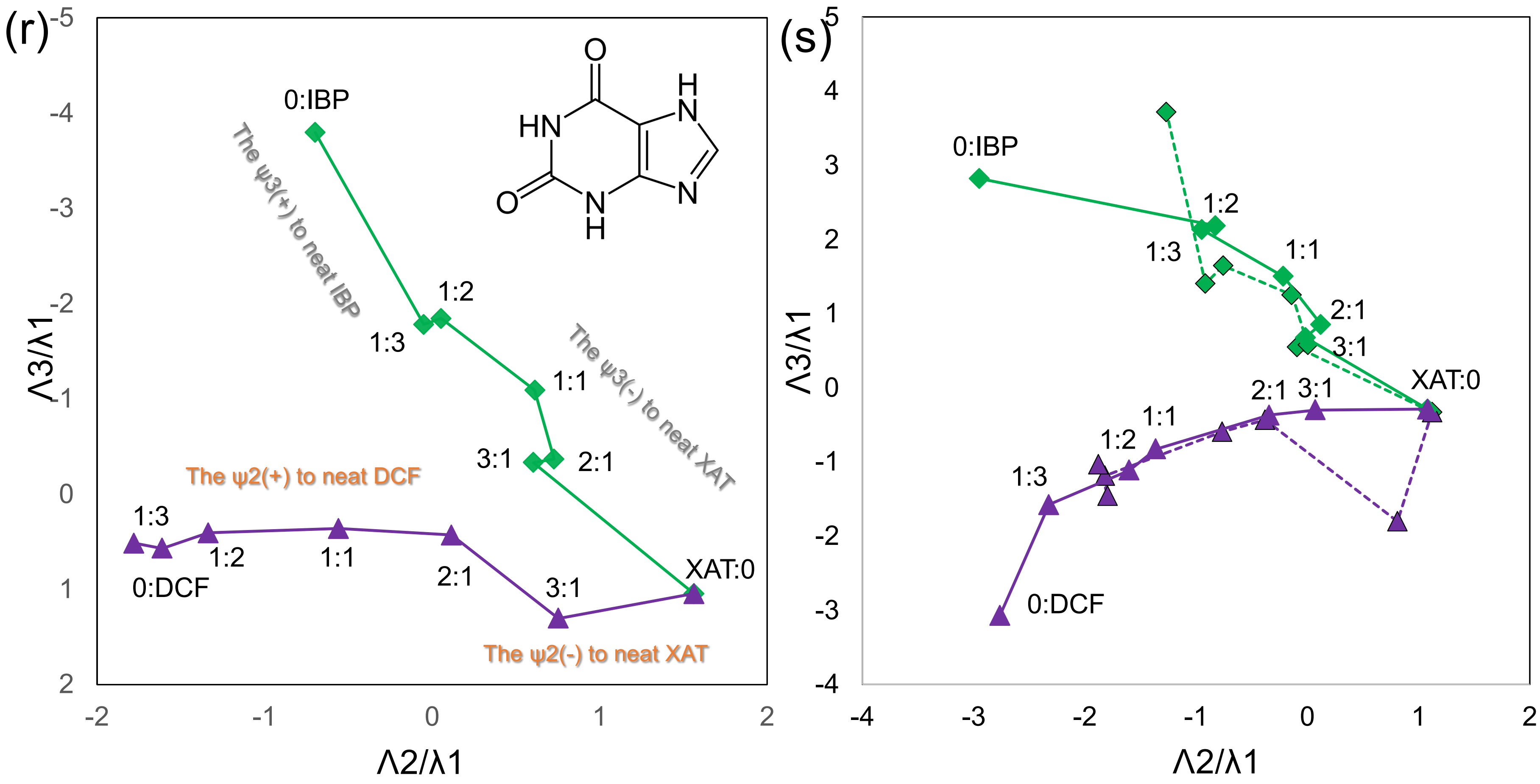

Figure S11 (r and s). The trajectories of the XAT/IBP (green diamonds) and XAT/DCF (violet triangles) mixtures, in which the (λ2/λ1)-axis flip-flopped. They shared the neat XAT's vector (XAT:0). The molar ratios were represented along the corresponding plots. The trajectory of the XAT/DCF mixtures traced along the (λ2/λ1)-axis (DCF owning dominance in the SVD analysis), whereas that of the XAT/IBP mixtures pursued in proportion to the diagonal line, suggesting the dependence between the trajectories of the XAT/IBP and XAT/DCF mixtures. The S13(r) and S13(s) show the trajectories of the average spectra and the individually measured spectra, respectively.

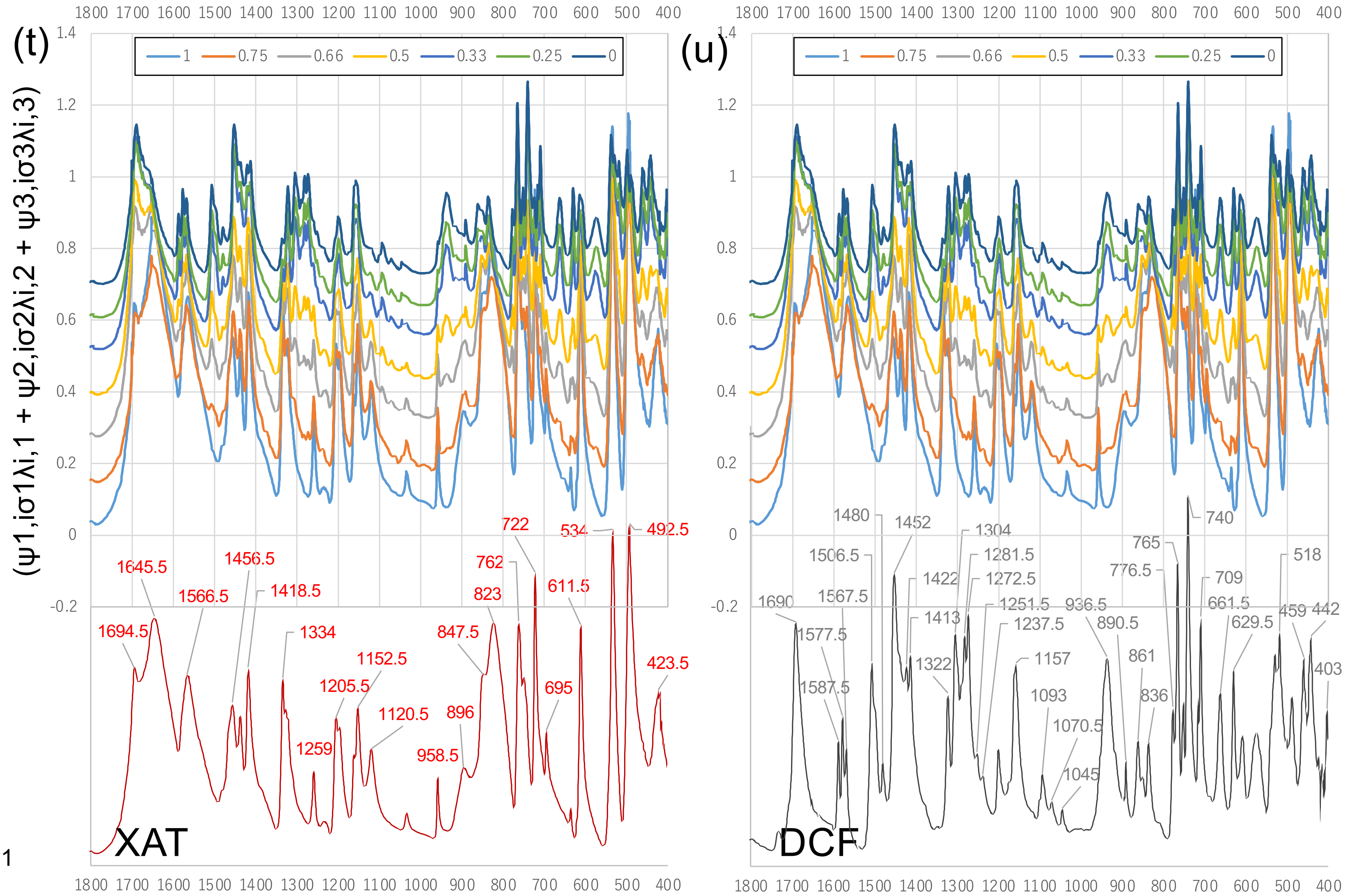

(t)
(u)
1 0.75 0.66 0.5 0.33 0.25 0
(ψ1,iσ1λi,1 + ψ2,iσ2λi,2 + ψ3,iσ3λi,3)
XAT
DCF
1694.5
1645.5
1566.5
1456.5
1418.5
1334
1259
1205.5
1152.5
1120.5
958.5
896
847.5
823
762
722
695
611.5
534
492.5
423.5
1690
1577.5
1587.5
1567.5
1506.5
1480
1452
1422
1413
1322
1304
1281.5
1272.5
1251.5
1237.5
1157
1093
1070.5
1045
936.5
890.5
861
836
776.5
765
740
709
661.5
629.5
518
459
442
403

Figure S11

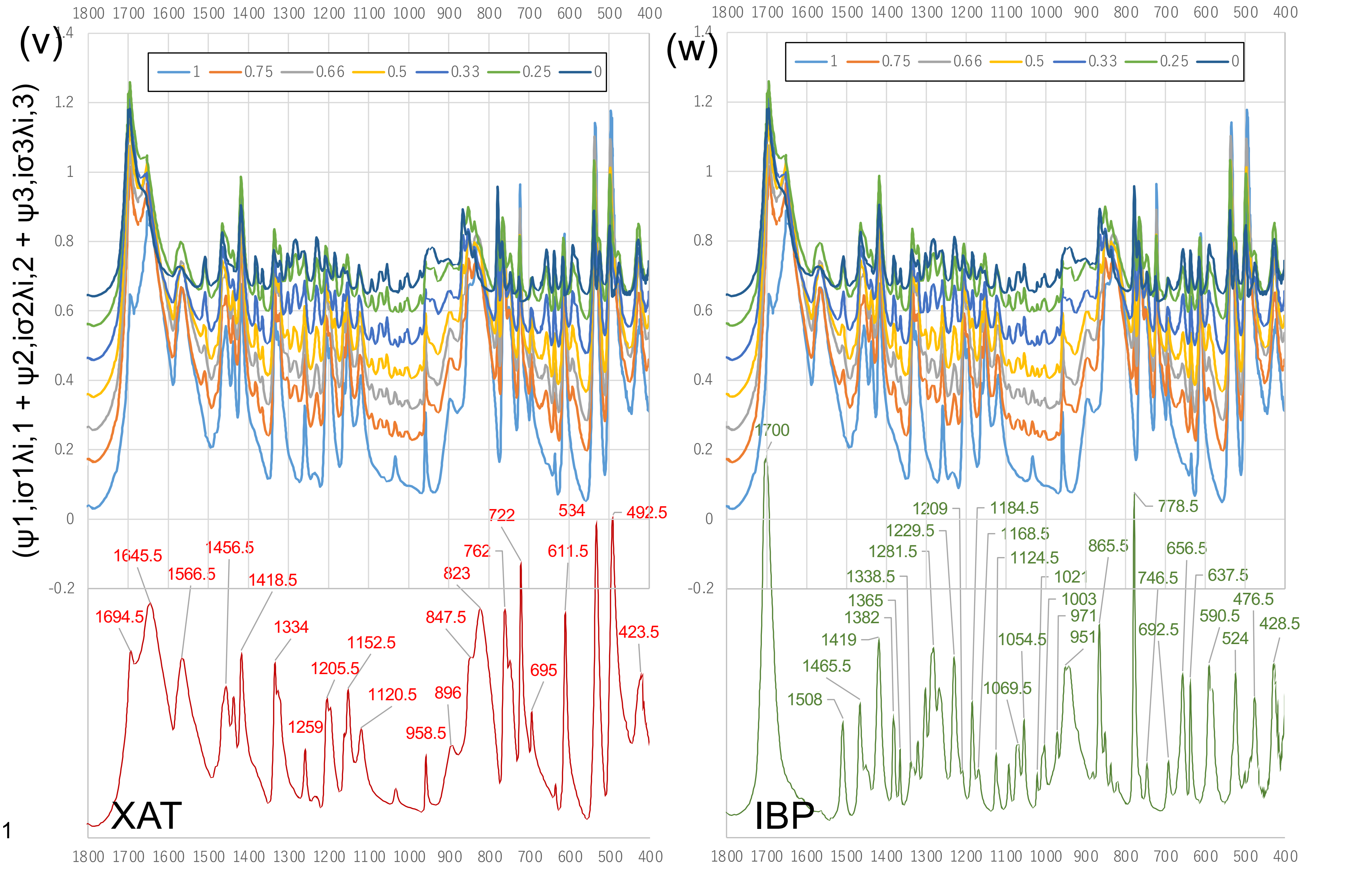
(v)
(w)
(ψ1,iσ1λi,1 + ψ2,iσ2λi,2 + ψ3,iσ3λi,3)
1
0.75
0.66
0.5
0.33
0.25
0
XAT
1694.5
1645.5
1566.5
1456.5
1418.5
1334
1259
1205.5
1152.5
1120.5
958.5
896
847.5
823
762
722
695
611.5
534
492.5
423.5
IBP
1700
1508
1465.5
1419
1382
1365
1338.5
1281.5
1229.5
1209
1184.5
1168.5
1124.5
1069.5
1054.5
1021
1003
971
951
865.5
778.5
746.5
692.5
656.5
637.5
590.5
524
476.5
428.5

Figure S11

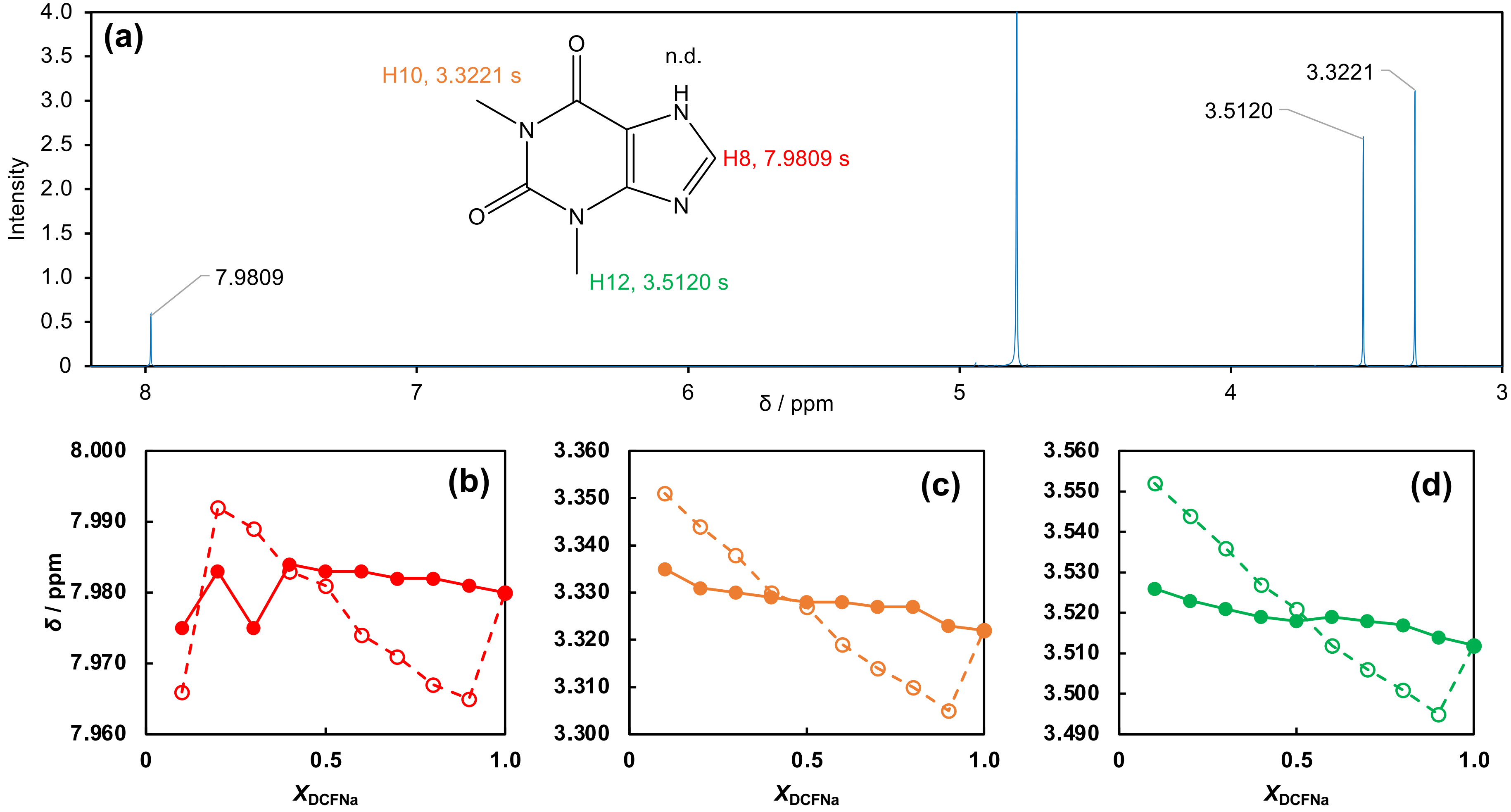

Figure S12. (a) Signal assignment for TPH on 400 MHz ^{1}H-NMR spectrum of 20 mM TPH in D_2O. The obtained chemical shifts in the various mole fractions of DCF sodium salt in the THP/DCF mixtures, for H8 (b), H10 (c), and H12 (d). The closed plots show the chemical shifts in the TPH/DCF mixtures, where the total amount of TPH and DCF sodium salt was adjusted to 20 mM. The open plots demonstrate those obtained by the measurement of TPH alone at the concentration corresponding to each mole fraction.

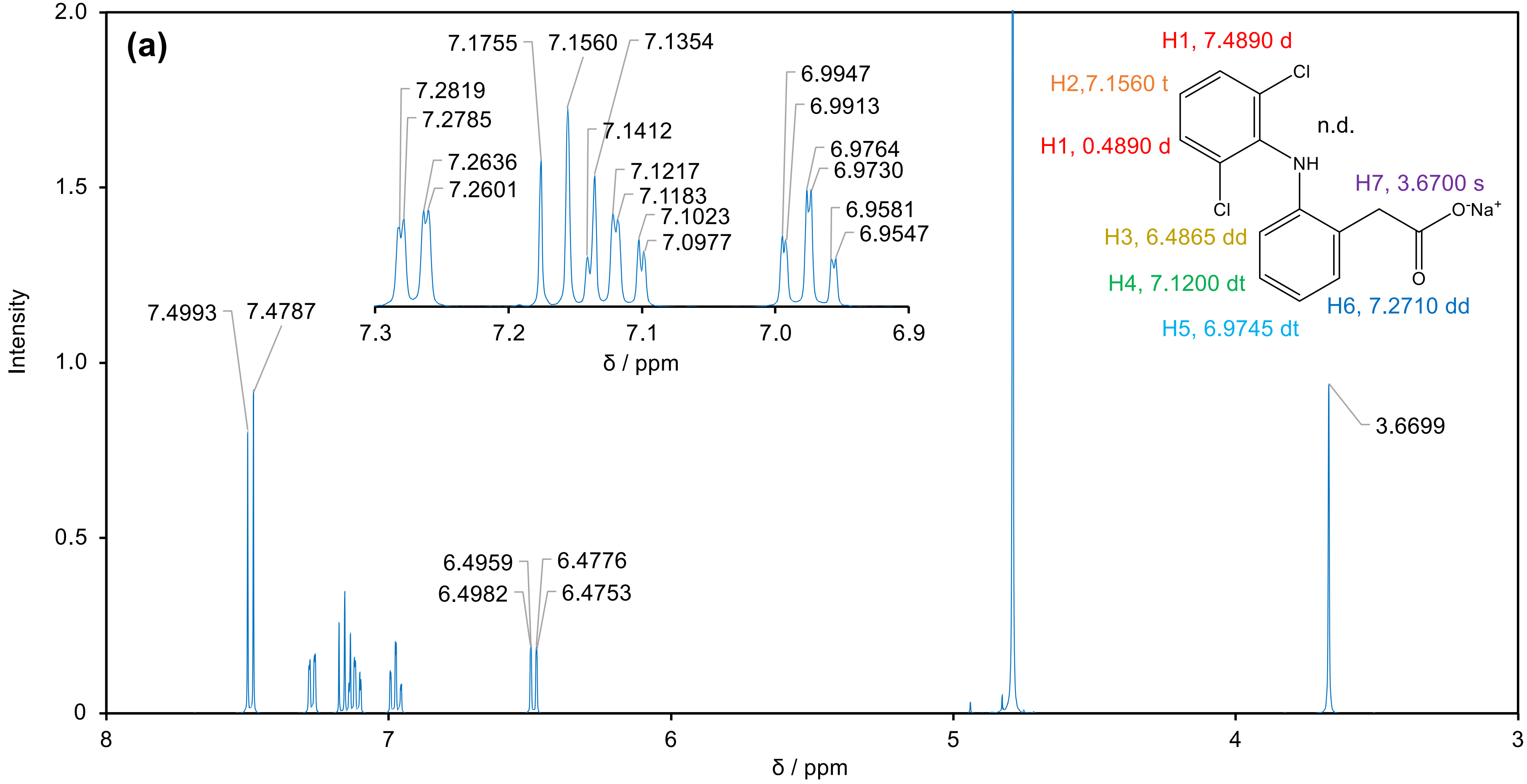

Figure S13. (a) Signal assignments for DCF sodium salt on 400 MHz ^{1}H-NMR spectrum of 20 mM DCF sodium salt in D_2O, assisted with the cross peaks in $^{1}H,^{1}H$-COSY and NOESY experiments. As the 7.1560 ppm triplet signal (H1) partially overlapped with the 7.1200 double triplet signal (H4), signal details were deformed. The 7.4890 ppm doublet signal (H2) was coupled to the 7.1560 ppm triplet signal (H1) with the vicinal *J* value of 8.24 Hz. The vicinal *J* value between H3 and H4 was 8.24 Hz and that between H5 and H6 was observed as 7.32 Hz. The long-range *J* values between H3 and H5 and between H4 and H6 would be determined as 0.92 and 1.32 Hz, respectively.

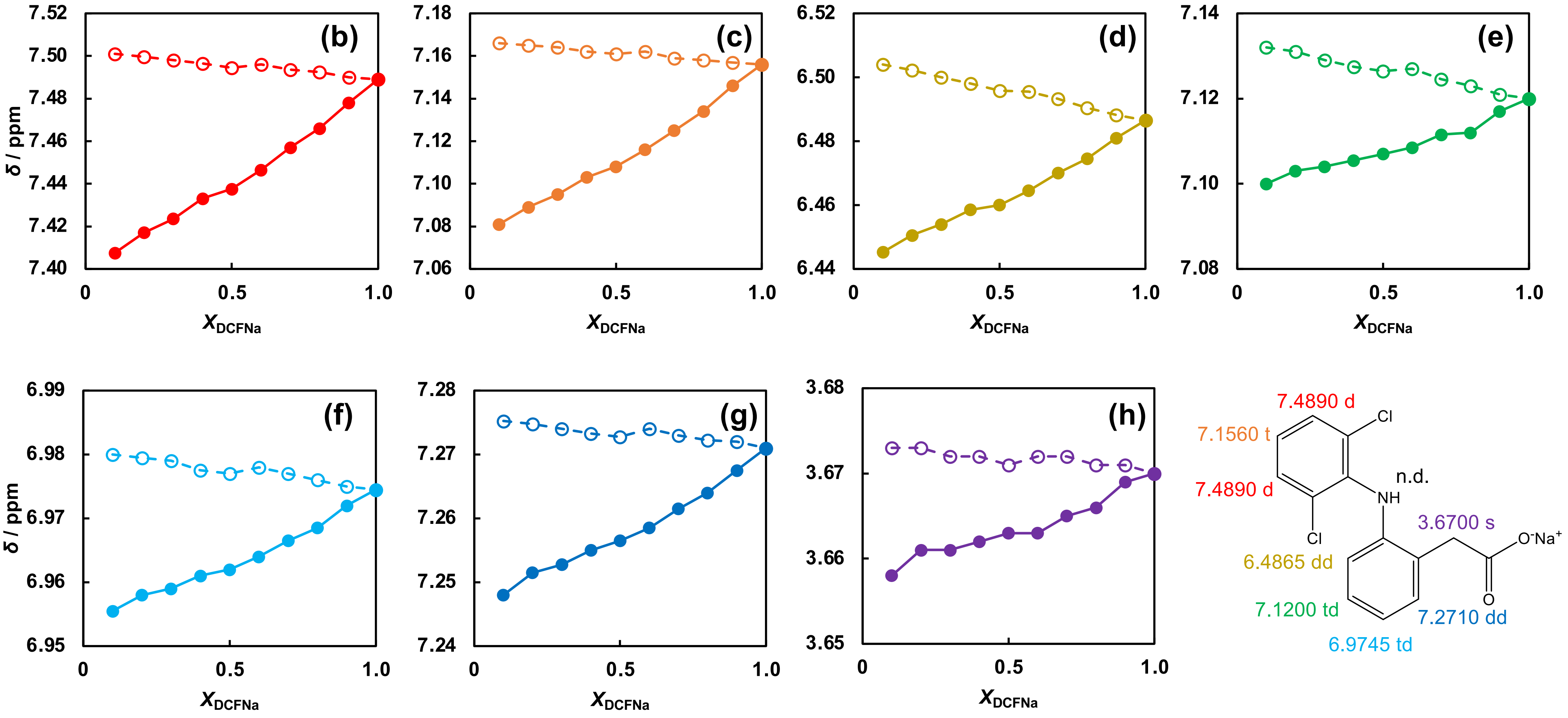

Figure S13. The obtained chemical shifts in the various mole fractions of DCF sodium salt in the THP/DCF mixtures, for H1 (b), H2 (c), H3 (d), H4 (e), H5 (f) H6 (g), and H7 (h). The closed plots show the chemical shifts in the TPH/DCF mixtures, where the total amount of TPH and DCF sodium salt was adjusted to 20 mM. The open plots demonstrate those obtained by the measurement of DCF sodium salt alone at the concentration corresponding to each mole fraction.

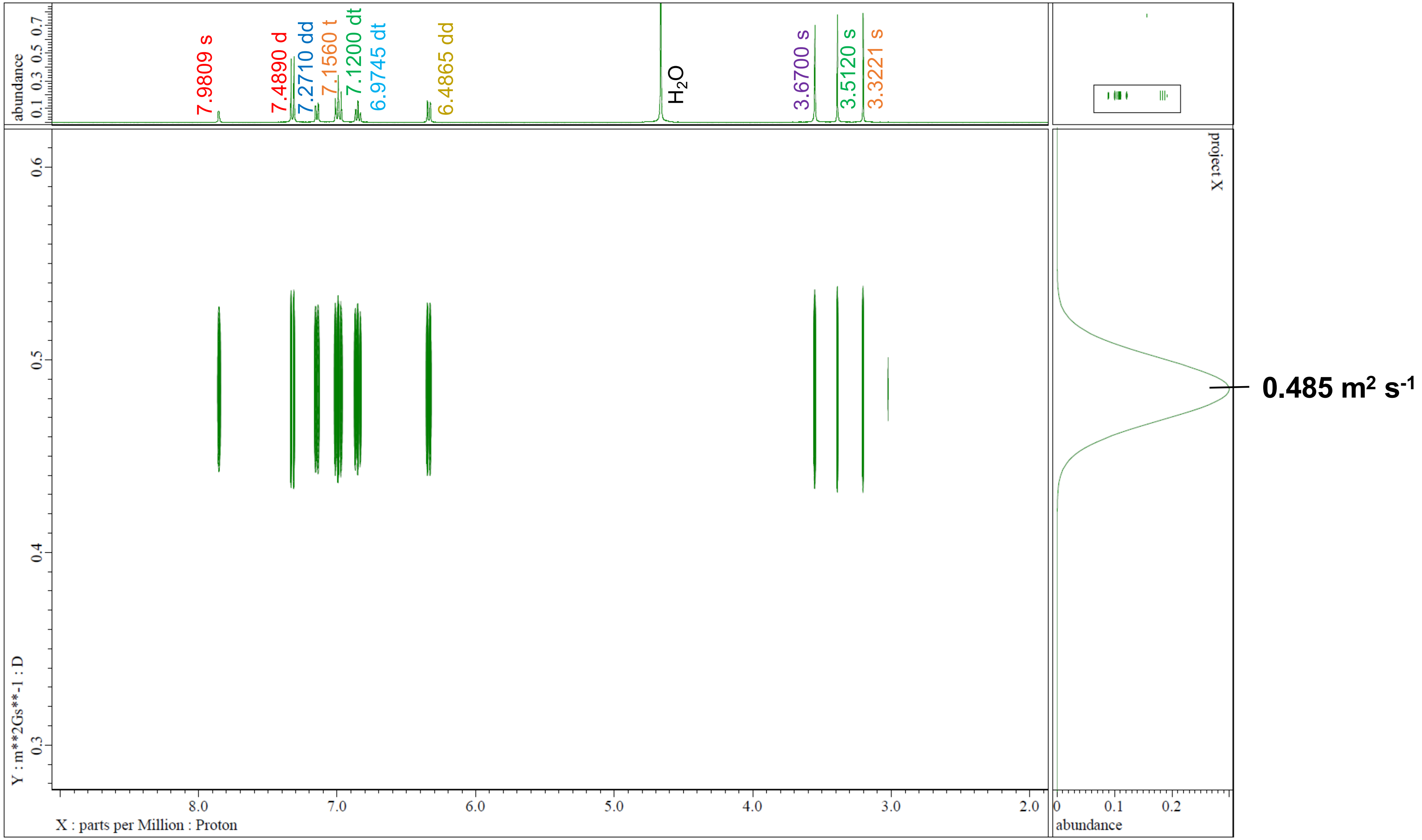

Figure S14. DOSY spectrum of the mixture containing 20 mM DCF sodium salt and 10 mM TPH in D_2O. The signals at 7.9809, 3.5120, and 3.3221 ppm were H8 and the N-Me hydrogens of TPH. The others were assigned to DCF, described in Figure S12(a). The D_{DOSY} value of 0.485 m^2 s^{-1} was observed as the property of the TPH/DCF complex formed in D_2O.

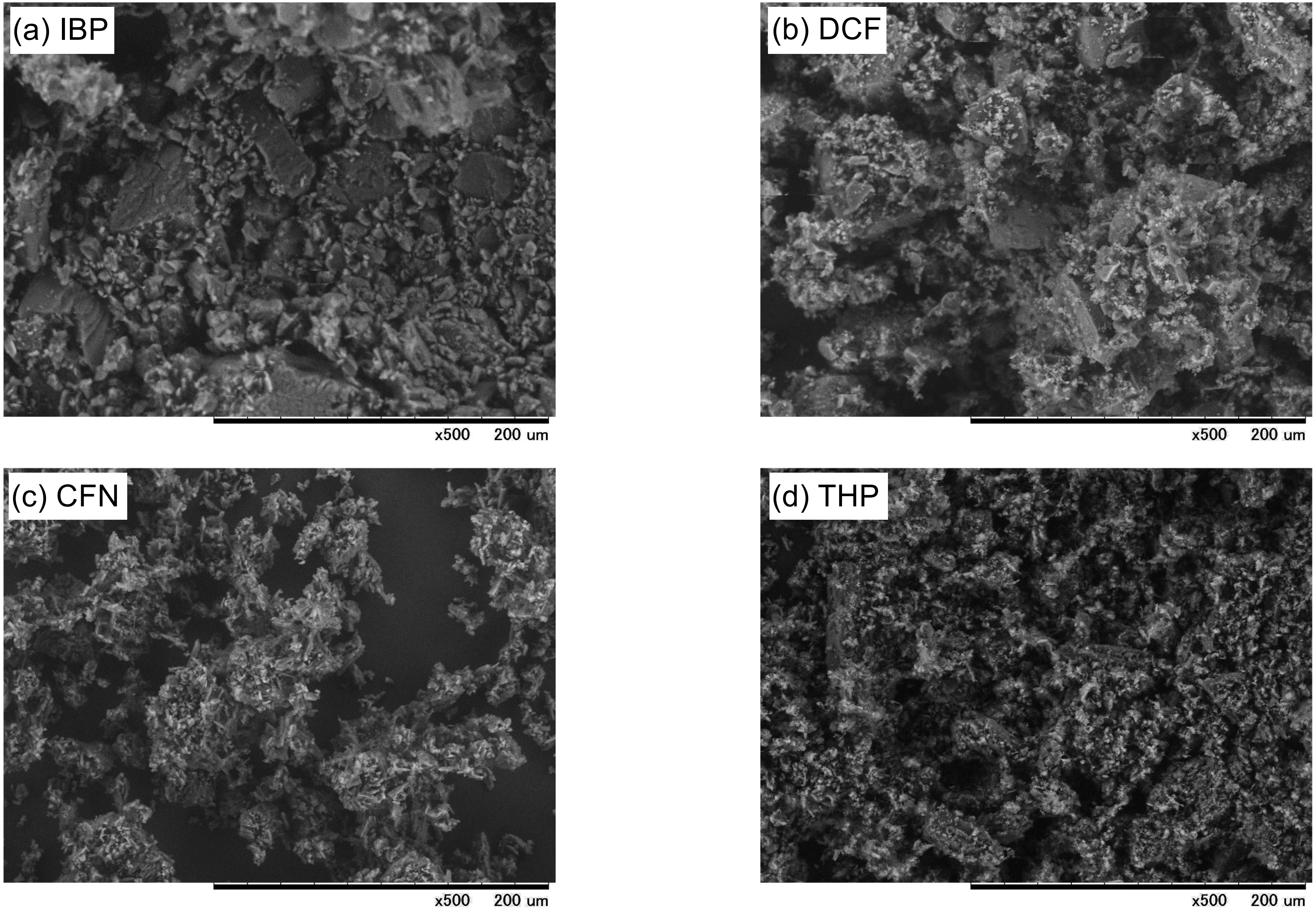
(a) IBP
x500 200 um
(b) DCF
x500 200 um
(c) CFN
x500 200 um
(d) THP
x500 200 um

Figure S15

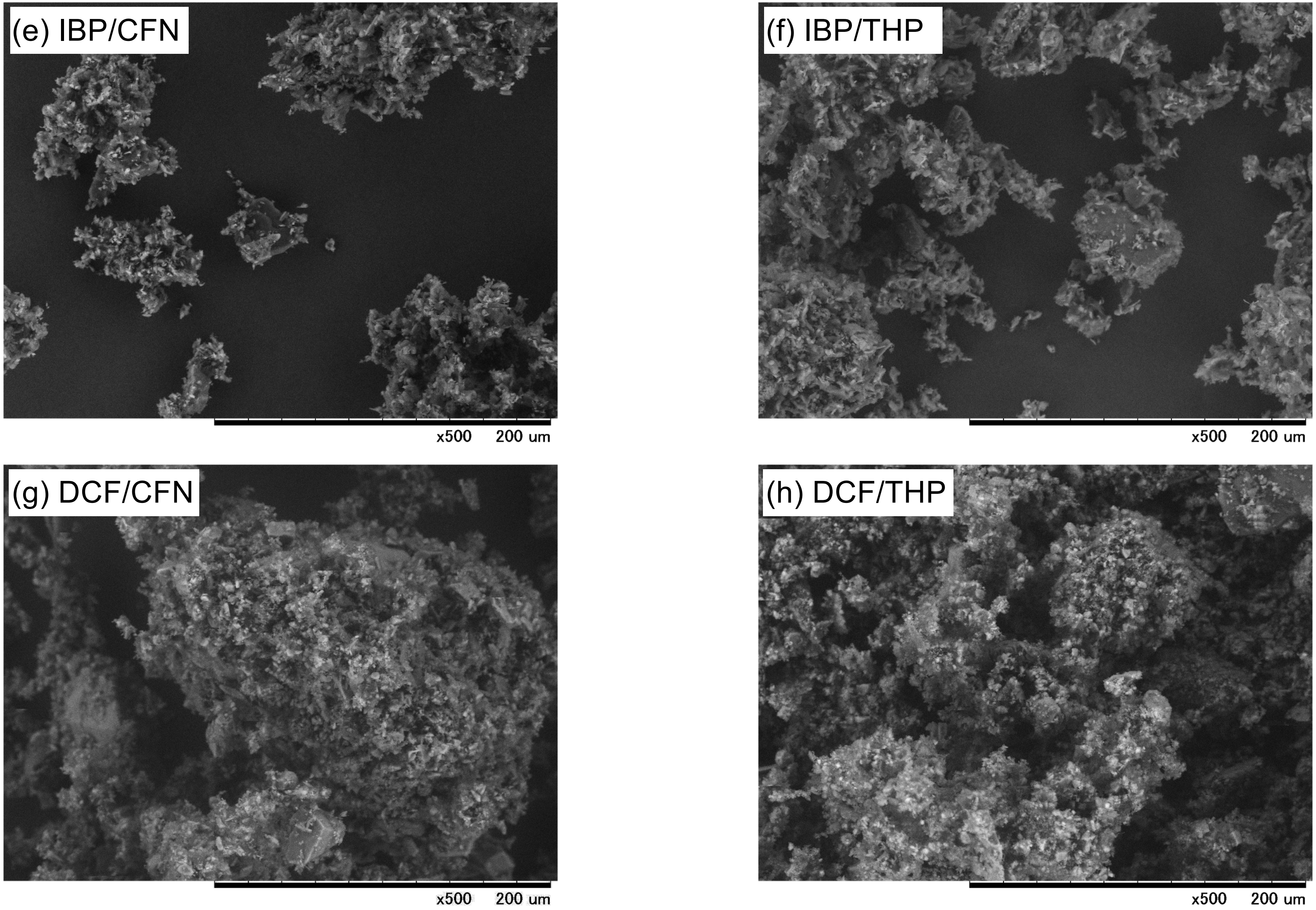
(e) IBP/CFN
x500 200 um
(f) IBP/THP
x500 200 um
(g) DCF/CFN
x500 200 um
(h) DCF/THP
x500 200 um

Figure S15

Figure S15. The SEM images of IBP (a), DCF (b), CFN (c), and THP (d) after the grind. Additionally, images of equimolar mixtures IBP/CFN (e), IBP/THP (f), DCF/CFN (g), and DCF/THP (h) after 7 days from preparation (physical mixture and grind). While the crystals of IBP and DCF were observed clearly despite the grind, those of CFN and THP were observed only in powder. From this, it is shown that CFN and THP crystals were easily crushed to fine crystalline powder. The powder adsorption around the crystals is observed in the SEM image of the mixture. These SEM images will become evidence of a solid-state interaction between IBP and DCF crystals and CFN and THP powders.
